# Terminal Pleistocene epoch human footprints from the Pacific coast of Canada

**DOI:** 10.1371/journal.pone.0193522

**Published:** 2018-03-28

**Authors:** Duncan McLaren, Daryl Fedje, Angela Dyck, Quentin Mackie, Alisha Gauvreau, Jenny Cohen

**Affiliations:** 1 Hakai Institute, Calvert Island, British Columbia, Canada; 2 University of Victoria, Victoria, British Columbia, Canada; Max Planck Institute for the Science of Human History, GERMANY

## Abstract

Little is known about the ice age human occupation of the Pacific Coast of Canada. Here we present the results of a targeted investigation of a late Pleistocene shoreline on Calvert Island, British Columbia. Drawing upon existing geomorphic information that sea level in the area was 2–3 m lower than present between 14,000 and 11,000 years ago, we began a systematic search for archaeological remains dating to this time period beneath intertidal beach sediments. During subsurface testing, we uncovered human footprints impressed into a 13,000-year-old paleosol beneath beach sands at archaeological site EjTa-4. To date, our investigations at this site have revealed a total of 29 footprints of at least three different sizes. The results presented here add to the growing body of information pertaining to the early deglaciation and associated human presence on the west coast of Canada at the end of the Last Glacial Maximum.

## Introduction

Based on modern estimates, an active individual human will make over 224,000,000 steps over a lifespan of 65 years based on an average of 9448 steps per day [1]. Most footprints that result from these steps are ephemeral, poorly defined and disappear quickly [2]. In some cases, a distinct cast of the foot is left in soft sediments, preserving a representation of the foot and motion of the individual. In many societies, specialists, such as trackers and gumshoes, can draw inferences about certain aspects of the individual that left their footprints behind [3,4]. In exceptional circumstances tracks are preserved in the geological record and paleontologists, paleoanthropologists and archaeologists can set about interpreting the tracks [5,6]. For example, the Australopithecine trackways from Laetoli in Tanzania have been intensively studied and reported upon [7–11].

Although found in many different types of sedimentary contexts worldwide (for example in cave floors and volcanic ash), a high proportion of hominin trackways have been discovered in near-shore settings [2,5,12–15]. When it comes to footprints, coastal settings are unique in some respects in that they are places where exposed soft and semi-saturated sediments abound, and they are linear focal regions for human and animal activity. Tidal, wave and aeolian action can provide inputs of additional sediment which may work to fill and cap footprints, adding to the potential of preservation in the geological record [16–19]. In many cases, coastal erosion results in the re-exposure of trackways, although it will most often also result in the destruction of these features [16]. The footprints reported on here were found in a near shore context of the Central Coast of British Columbia (BC), Canada, although they were revealed through excavation as opposed to erosion.

During the Last Glacial Maximum, parts of the western edge of the Cordilleran Ice Sheet terminated at the Pacific coastline [20–22]. Some land between this meeting of ice and sea remained unglaciated, providing refugia for some plants and animals [23–25]. Between 19,000 and 16,000 years ago refugia became larger and more common along the outermost part of the coast [26–28]. These refugia were able to support vegetation as well as large land mammals. It is possible that humans also inhabited these refugia, in particular if they employed the use of watercraft and were able draw their subsistence from maritime and intertidal sources [29]. As such, the western margin of the Cordilleran Ice Sheet is one of the hypothesized means by which human populations moved from Beringia into mid-latitude North America during the last ice age [30]. Archaeological evidence along this proposed coastal migration route is, however, scant. To date, few archaeologists working in the area have attempted focused research projects to test if such evidence exists.

Very few late Pleistocene archaeological sites are known on the Pacific Coast of Canada. Until quite recently, the oldest known site in British Columbia (BC) was the Charlie Lake Cave site. The site is situated at the northern end of the interior “Ice Free Corridor” and the assemblage of artifacts includes a fluted projectile point, dated 12,500–12,100 cal BP [31]. Similar radiocarbon ages were found in association with a lithic assemblage at the Vermillion Lakes site in Alberta, near the southern end of the “Ice Free Corridor” dated between 12,700 and 11,800 cal BP [32]. Early coastal sites in British Columbia and southeast Alaska were, until recently, not known to predate the earliest Holocene [33,34]. To the south, on the Olympic Peninsula in Washington State, the Manis Mastodon site is the best known and oldest documented site from the glaciated coastal region, represented by a mastodon rib with a bone point lodged in it dated to 13,860–13,768 cal BP [35].

In recent years, late Pleistocene aged sites have been found on the coast and as a result the archaeological record of the area has been pushed back in time with the adoption of new strategies. In particular, chipped stone tools including projectile points and fragments found in ancient bear dens on Haida Gwaii have been dated to 12,800–12,600 cal BP [36,37]. Archaeological prospection on relict shorelines of the Central Coast of BC has uncovered a number of Pleistocene/Holocene transition aged sites [38]. The findings from Haida Gwaii and the Central Coast have come about as the result of targeting karst caves with terminal Pleistocene faunal remains [36,37] and relict shorelines of known age for archaeological investigation [39,40]. The project described here focuses on the latter approach. The ages of these Northwest Coast late Pleistocene archaeological sites are not as old as the 14,500 cal BP sites known from mid-latitude North America (e.g., [41,42]), or South America (e.g., [43]). Early archaeological sites on the Northwest Coast do, however, demonstrate terminal Pleistocene human populations in the Americas employed watercraft to reach the islands on which these sites are located. They were very likely able to draw the majority of their subsistence from the ocean and intertidal zone along partially glaciated coastlines [44]. As such, these findings add to the growing body of evidence that early peoples in the Americas inhabited the coastal margin and circumnavigated the western edge of the Cordilleran Ice Sheet to move between Beringia and mid-latitude North America at the end of the last ice age.

Archaeologists working on the Pacific coast of Canada who are interested in investigating the late Pleistocene human occupation of the region are typically faced with significant challenges. Today, much of this region is covered by dense stands of temperate rainforest or thick peat bogs. Overall, deflation and erosional events that can reveal archaeological deposits are rare, and organic soil accumulation tends to be substantial. Most of the current shoreline can only be accessed by boat as the myriad archipelagos, marine channels, waterways and steep-sided fjords preclude road transport for most of the coastline. In addition, the location of the shoreline during the late Pleistocene was highly variable from one region to the next as a result of the complex interplay between glacial isostacy, eustasy, tectonics and other factors [45,46]. For this reason, archaeological investigations into the early peopling of the region often begin with attempts to understand geomorphic processes, in particular charting where relative sea level was in different places at different times [38,40,47–50].

Globally, sea level was much lower during the Last Glacial Maximum as a result of the large amount of the earth’s hydrosphere being trapped on land as ice. In most regions of the globe, sea level was in the order of 120 m lower than today [51]. In some higher latitude areas, however, this eustatic lowering was counteracted by isostasy as glaciers advanced towards continental margins. On the Pacific coast of Canada, the changing volume of ice on the continent during and after the Last Glacial Maximum resulted in a complex array of sea level changes due primarily to eustatic and isostatic factors [45,46]. In parts of the mainland coast, sea level was up to 200 m higher than today [52], while on the outer islands, sea level was as much as 150 m lower at the same time [20,47,53,54].

**Fig 1:**
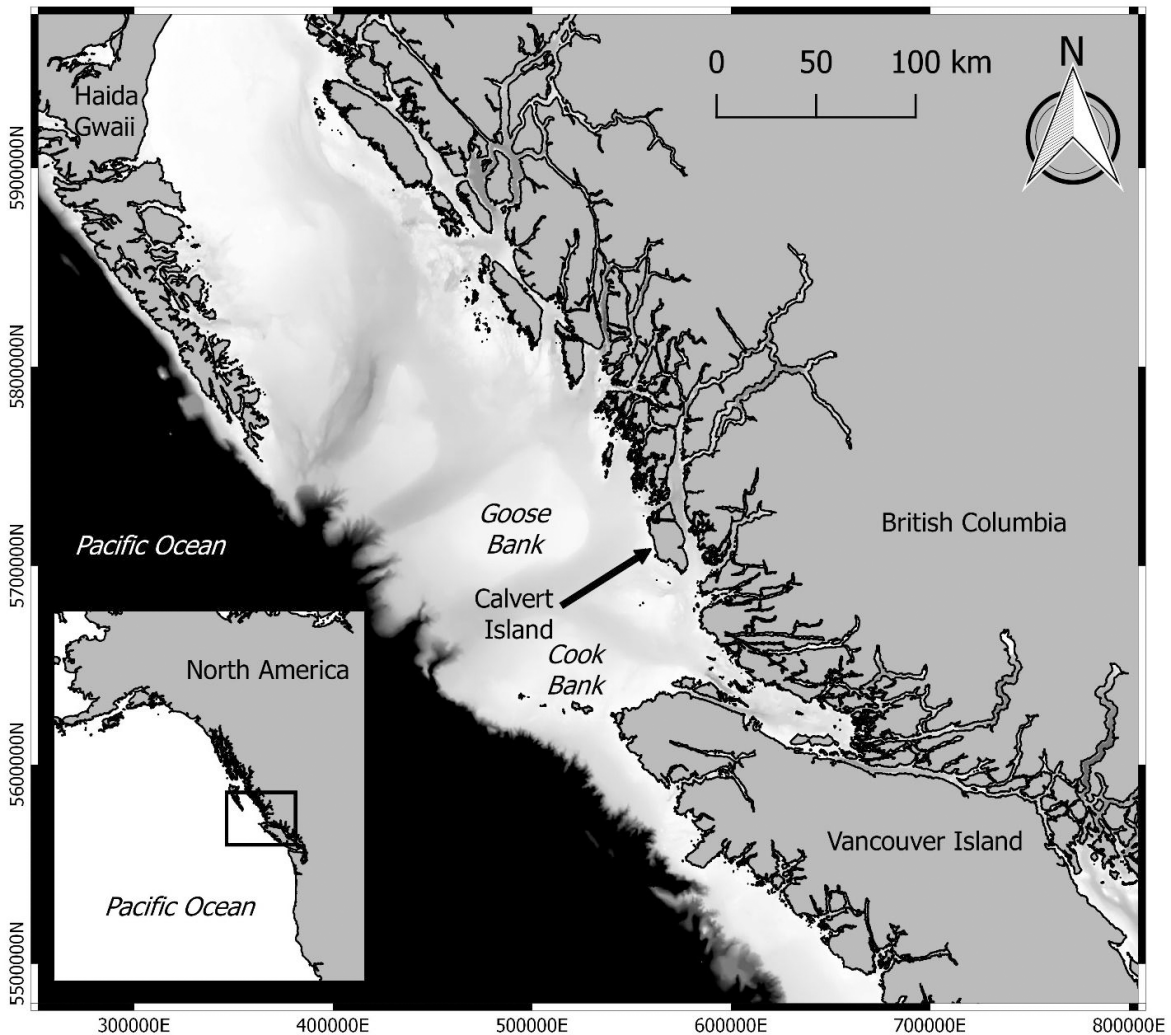
Location of Calvert Island on the west coast of Canada. Bathymetric data is shaded from black, representing -1000 metres and deeper to white, representing 0 metres. Bathymetric and coastline imagery is based on information created by SciTech and the Living Oceans Society.

**Fig 2:**
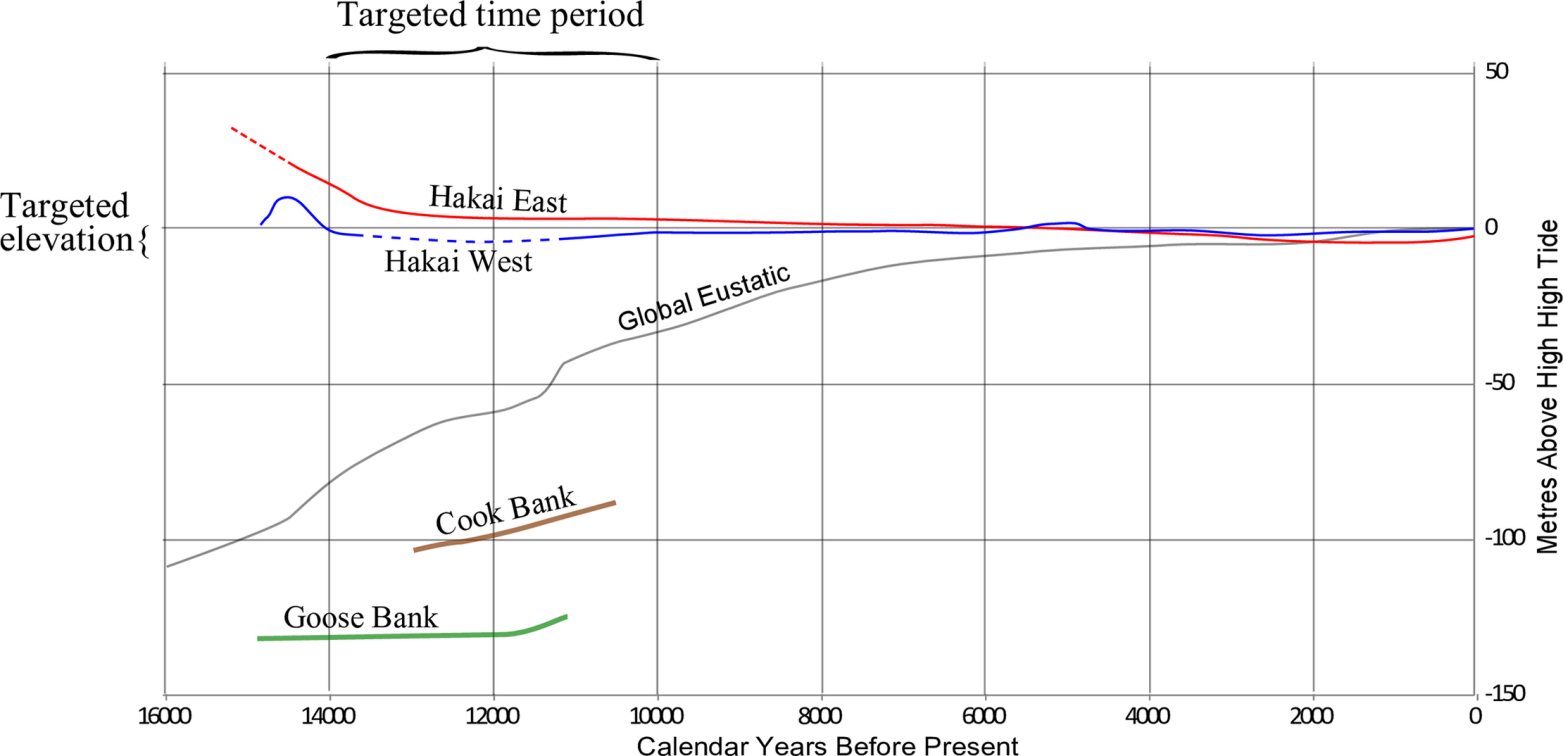
Chart showing sea level curves from the region around Calvert Island. The Hakai West curve is specific to Calvert Island and was used to target specific elevations and related time periods for archaeological prospection. Source for curves include Hakai East and West [40], Global Eustatic [51], Cook Bank [54] and Goose Bank [20], see Fig 1 for locations.

Calvert Island is situated between these two areas of vastly differing sea level history (Fig 1). After 14,500 years ago, sea level dropped rapidly on the mainland and rose rapidly on the outer coast, but remained relatively stable in between these two regions. On Calvert Island sea level was 2 to 3 metres lower than today between 14,000 and 11,000 cal BP [40] (Fig 2). With this knowledge, we devised a program of intertidal testing to search for archaeological deposits dating to this time period. Unexpectedly, a series of human footprints were found while testing deposits under a beach on Calvert Island, the results of which we report here.

### Study area

**Fig 3:**
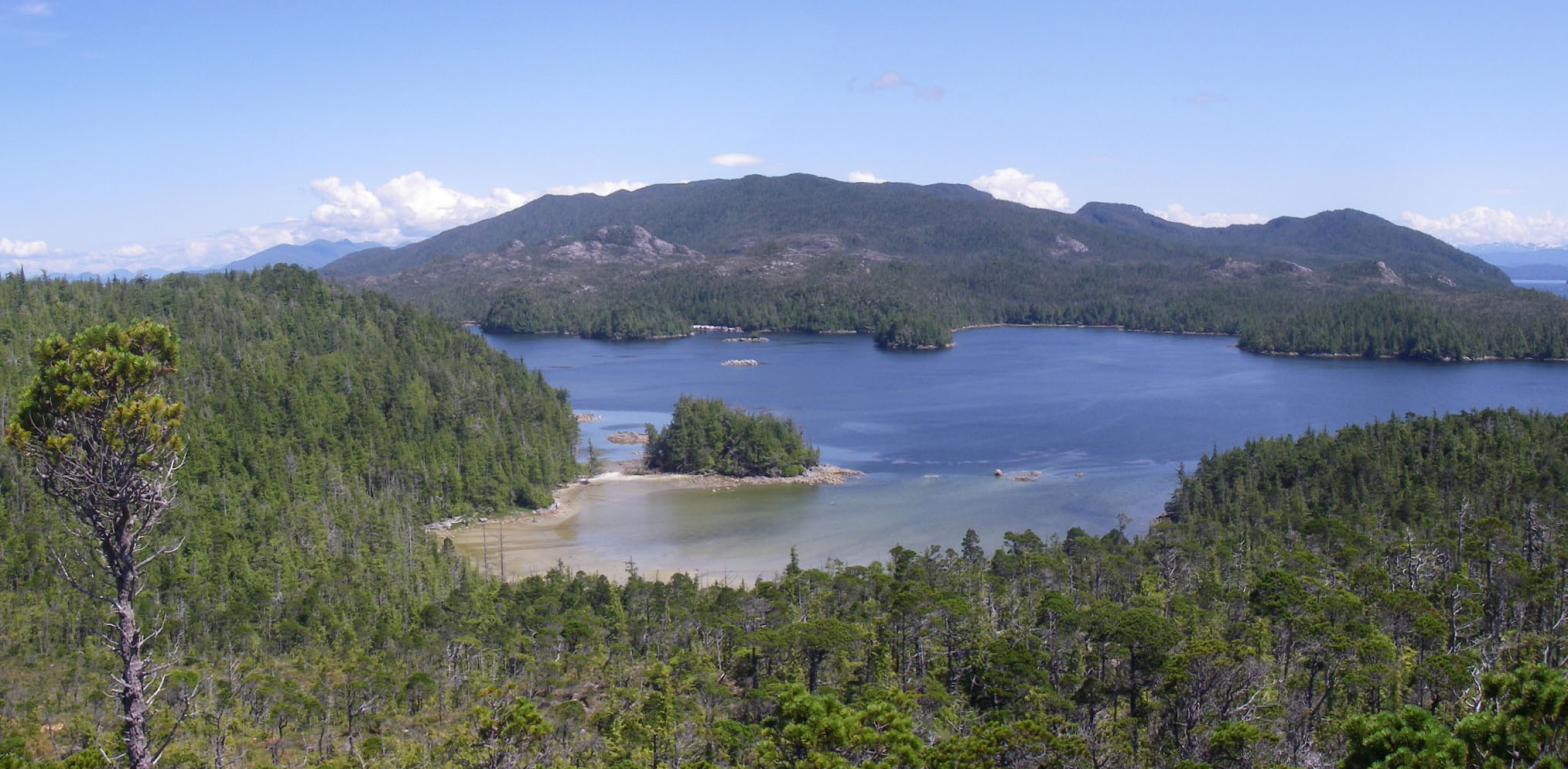
View across the beach at EjTa-4 with Calvert Island in the foreground and Hecate Island in the background. Photo by Jim Stafford.

The outer Central Coast of British Columbia is characterized by thousands of low relief islands, many of which are exposed to the open Pacific Ocean. As one moves from the outer coast to the inner, the elevation of landforms increase until one reaches the crests of the Coast Mountain Ranges. The landscape is typically covered by dense temperate rainforest with climax stands of mixed conifers, mostly western hemlock, Sitka spruce, western redcedar, shore pine and yellow cedar. In flatter areas, sphagnum dominated bogs have developed. Calvert Island is one of the larger outer coastal islands. The highest landform on the island is Mount Buxton with an elevation close to 1000 m. Overall, the shoreline of the region is rocky and sinuous in both protected and exposed areas (Fig 3). Northwestern Calvert Island is somewhat unique on the outer Central Coast of BC as it features large sand beaches in both protected and in exposed locales.

Landforms associated with protected beaches are often found to have associated archaeological deposits [38]. Sea level stability has contributed to the development of very deep shell midden features, some showing over 10,000 years of occupation [34,38].

**Fig 4:**
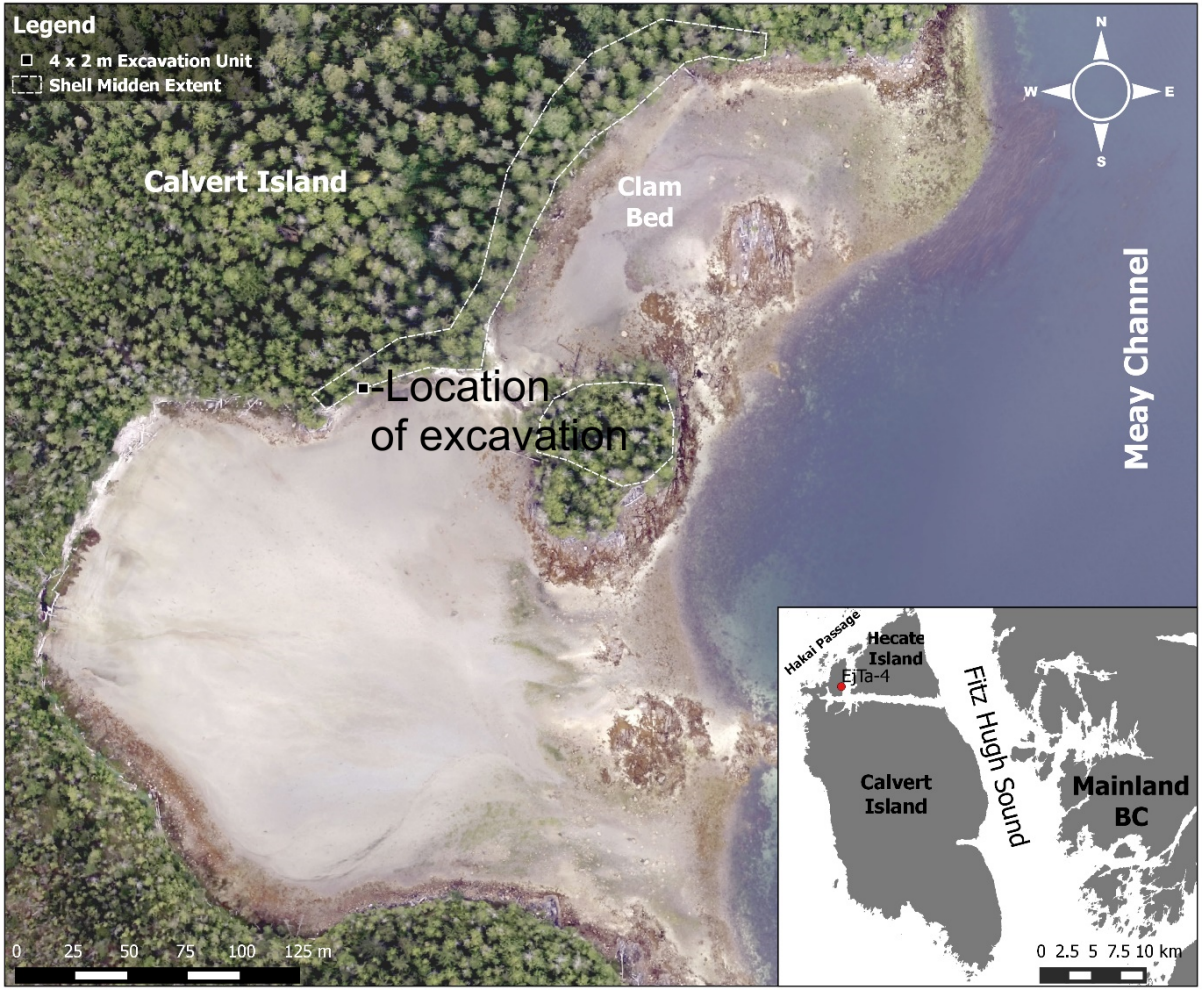
Drone based aerial photograph of EjTa-4 showing the location of the excavation unit with footprints. Imagery courtesy of the Hakai Institute. The shell midden boundary is based on information provided by Farid Rahemtulla.

The Meay Channel I site (EjTa-4) includes substantial Holocene aged shell midden deposits up to 5 m deep (Fig 4), which date from 6100 to 300 cal BP [38,55,56]. Extensive scatters of chipped stone tools were found on the surface of the intertidal zone in front of the midden. These surface assemblages could date to any time period, although chipped stone tools were more common in local cultural historical sequences of the region prior to 2,300 cal BP [57]. Other features include stone petroforms in the intertidal zone and numerous culturally modified trees in the forests surrounding the site.

## Methods

The beach in front of EjTa-4 was accessed by watercraft from the Hakai Institute facilities on Calvert Island. The field crew included academic and professional archaeologists, representatives from the Heiltsuk First Nation and Wuikinuxv First Nation, and students from the University of Victoria. A 7 cm diameter auger was used for initial sediment sampling, otherwise subsurface examinations were conducted using 50 x 50 cm trowel tests. A 4 x 2 m area was excavated by trowel in natural strata where late Pleistocene aged deposits were identified during initial testing. *In situ* artifacts, faunal remains, plant macrofossils, and features were mapped using line levels and tape measures. Excavated sediments were water screened through 3 and 6 mm mesh. Bulk sediment samples were taken in association with individual strata, footprints, other features and *in situ* artifacts.

Measurements of the revealed footprints were undertaken in the field and included length of the central axis (from the heel to the distal edge of the second phalanx) and maximum width of the ball of the foot as outlined in Bennett and Morse [5].

Following Marty et al. [58] we draw on the following terminology to describe the footprints found at EjTa-4. The term ‘track’ refers to the footprint impression in general and here we use it interchangeably with ‘footprint’. ‘True track’ refers to the cast created by sediment displacement caused by the foot. ‘Under track sediments’ are beneath the true track. The upper part of the undertrack sediments may be compressed and have materials (plant macrofossils, shell fragments etc.) from the surface pressed into them from the force of the foot applying pressure downwards. ‘Over track’ sediment refers to sediments that were deposited after the footprint impressions were made. ‘Track surface’ refers to the original paleo-surface that was walked across. The ‘displacement rim’ is created as a result of lateral sediment displacement when the foot presses down on the ground, and by the slight upwards pull around the true track when the foot is lifted out of the print.

All collected materials were transported to the archaeological laboratories at the Hakai Institute on Quadra Island and the University of Victoria for cataloguing and analysis. Samples for radiocarbon dating were sent to the W.M. Keck Carbon Cycle Accelerator Mass Spectrometry Laboratory at the University of California, Irvine for analysis. Faunal remains were analyzed using the comparative collection at the University of Victoria Zooarchaeology lab. Lithics were analyzed drawing on the typology developed for the region by Rahemtulla [59]. Photographs of the tracks were digitally enhanced using the D-stretch plugin for Imagej [60], a freeware image processing and analysis program.

Some sediments were subsampled and prepared for light microscopic identification of sediment sphericity, pollen, spores, diatoms and other micro-organisms. Light microscope transects were conducted on the slides using 400X and 1000X magnification to detect the presence or absence of these and to identify those that were present. Samples of wood were identified to taxon through cellular analysis, using a comparative collection and following standard texts [61–63]. Wood samples were analyzed using high-powered microscopy viewed at 100X-800X magnification.

## Results

Stratified sediments were found beneath the beach surface on the southern side of EjTa-4 during initial subsurface sampling in the intertidal zone in 2014. In one 50 x 50 cm trowel test, a suspected human track was found 60 cm below the beach surface. The feature was impressed into the top of a light brown clay and was filled with dark brown sand and pea gravel. At the base of the true track impression, directly under the track fill, pieces of preserved wood were recovered. Two of these were radiocarbon dated, resulting in age estimates of 13,169–13,095 cal BP (11,295 ± 30 ^14^C BP—UCIAMS142561) and 13,317–13,241 cal BP (11,435 ± 30 ^14^C BP–UCIAMS 149779).

**Fig 5:**
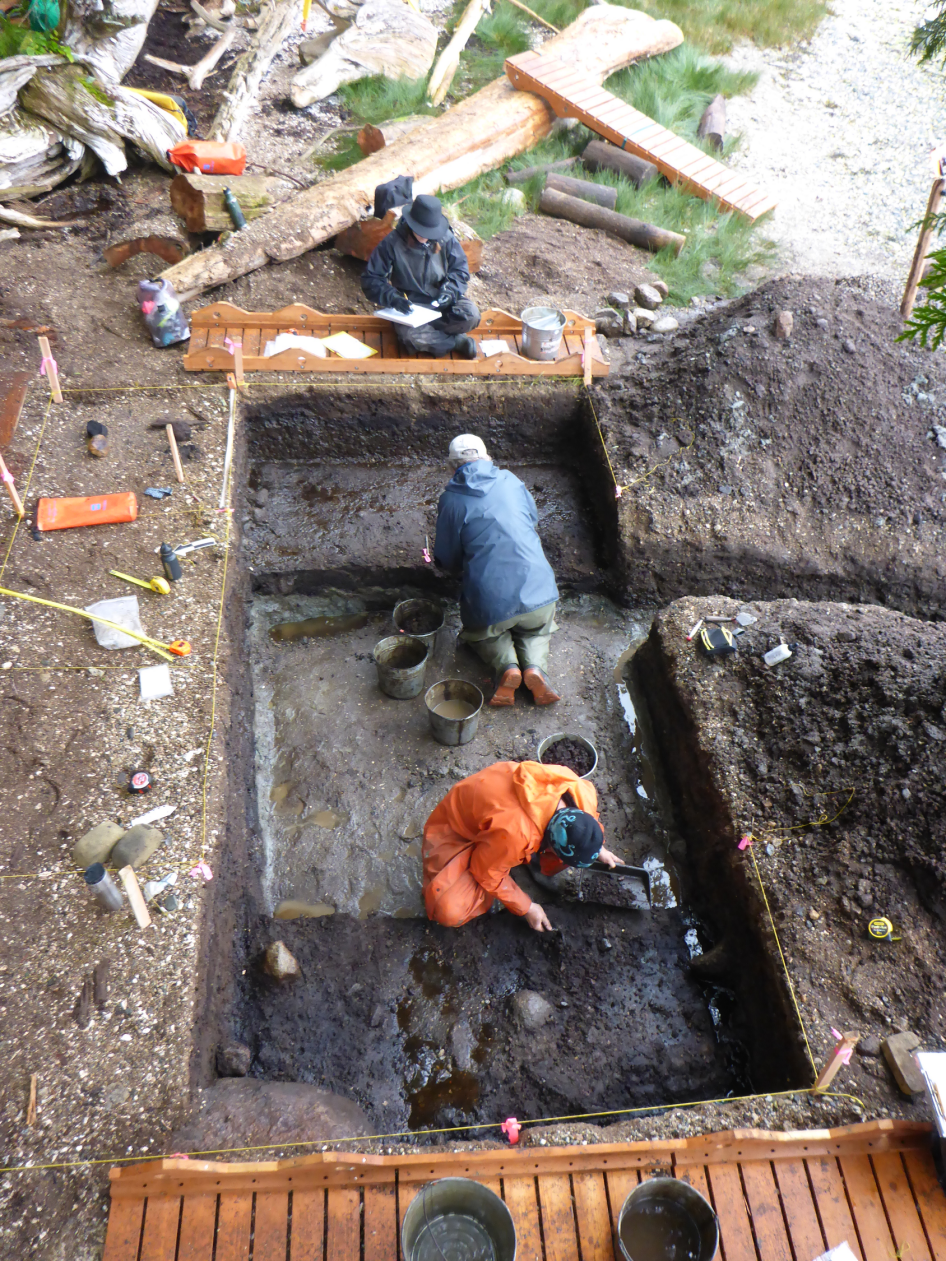
View of the 4 x 2 metre excavation unit. The 2 x 2 metre square at centre was removed in 2015 to Stratum XII. The 2 x 1 metre units on either side of this, under active excavation of Stratum VII, were removed in 2016. Photo by Joanne McSporran.

**Fig 6:**
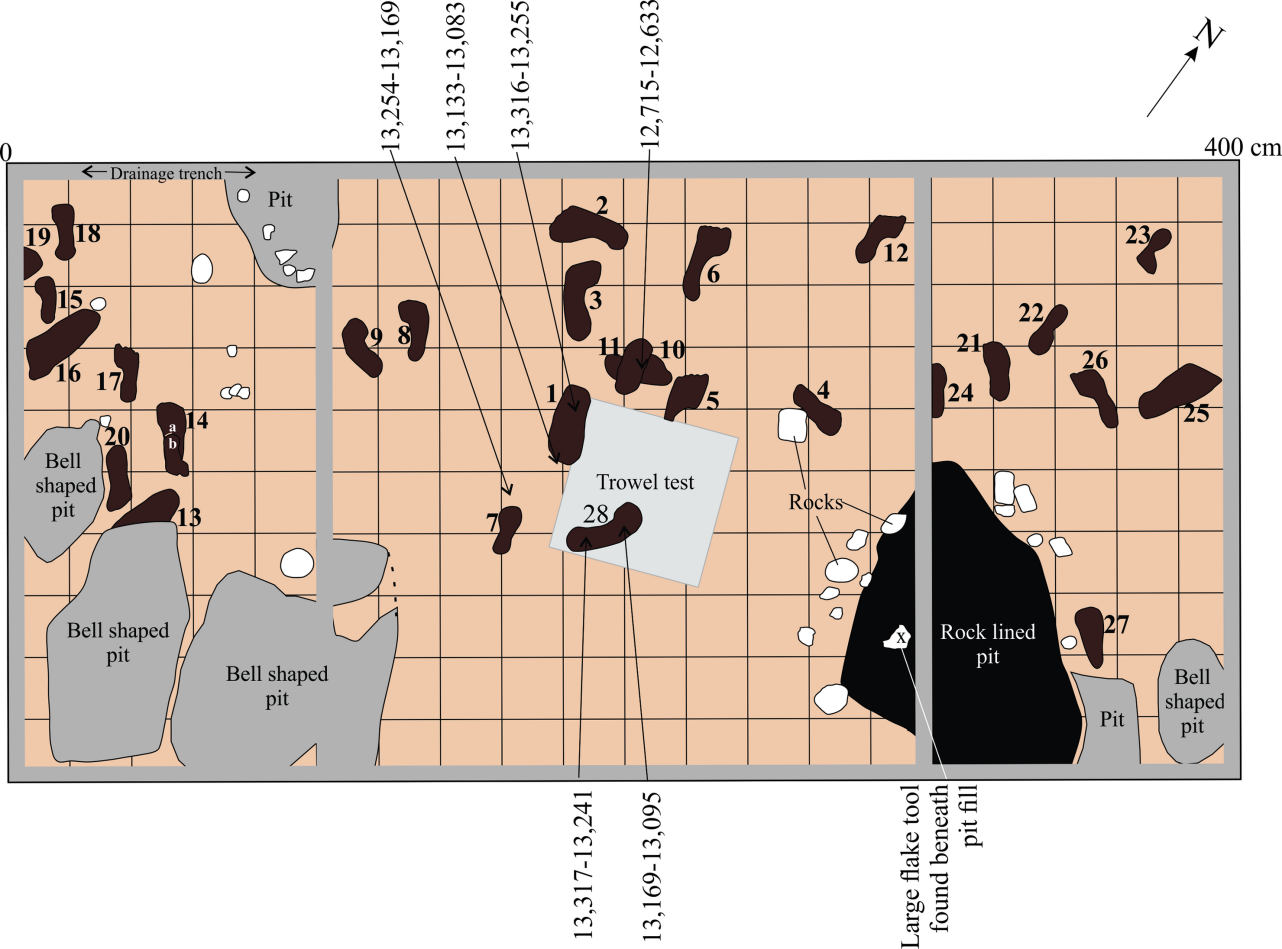
Planview of 4 x 2 m excavation unit undertaken at EjTa-4 showing trackway and track surface. This illustration is based on field notes and photographs. Radiocarbon dates (cal BP) are from the base of footprint impressions and wood found adjacent to these features on the track surface. All of the bell-shaped pits (shaded grey) are intrusive into the track surface from above with the exception of the rock-lined pit (shaded black) which appears to be stratigraphically associated with the track surface.

**Fig 7:**
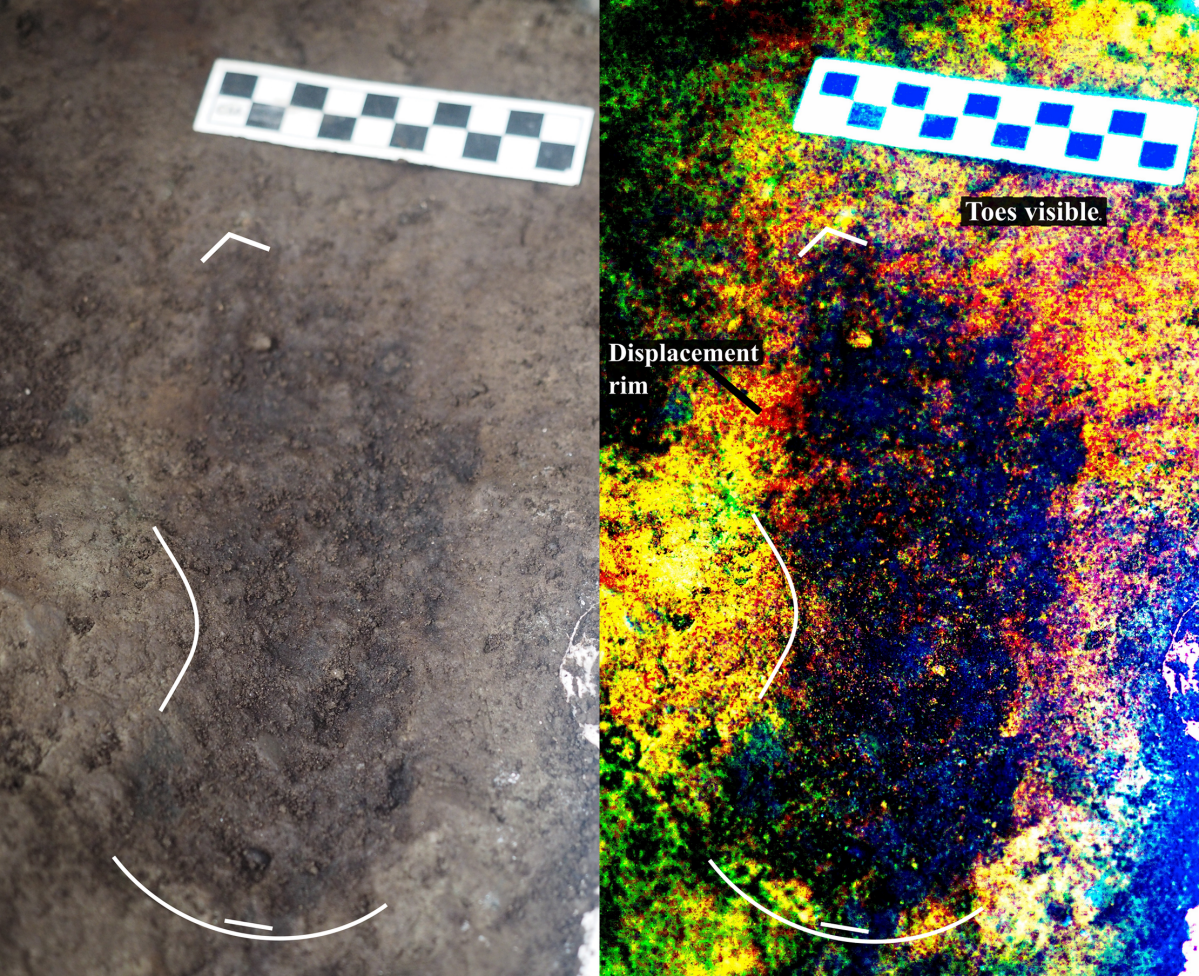
Photograph of track #17 beside digitally-enhanced image of same feature using the DStretch plugin for ImageJ. Note the toe impressions and arch indicating that this is a right footprint. Photo by Duncan McLaren.

**Fig 8:**
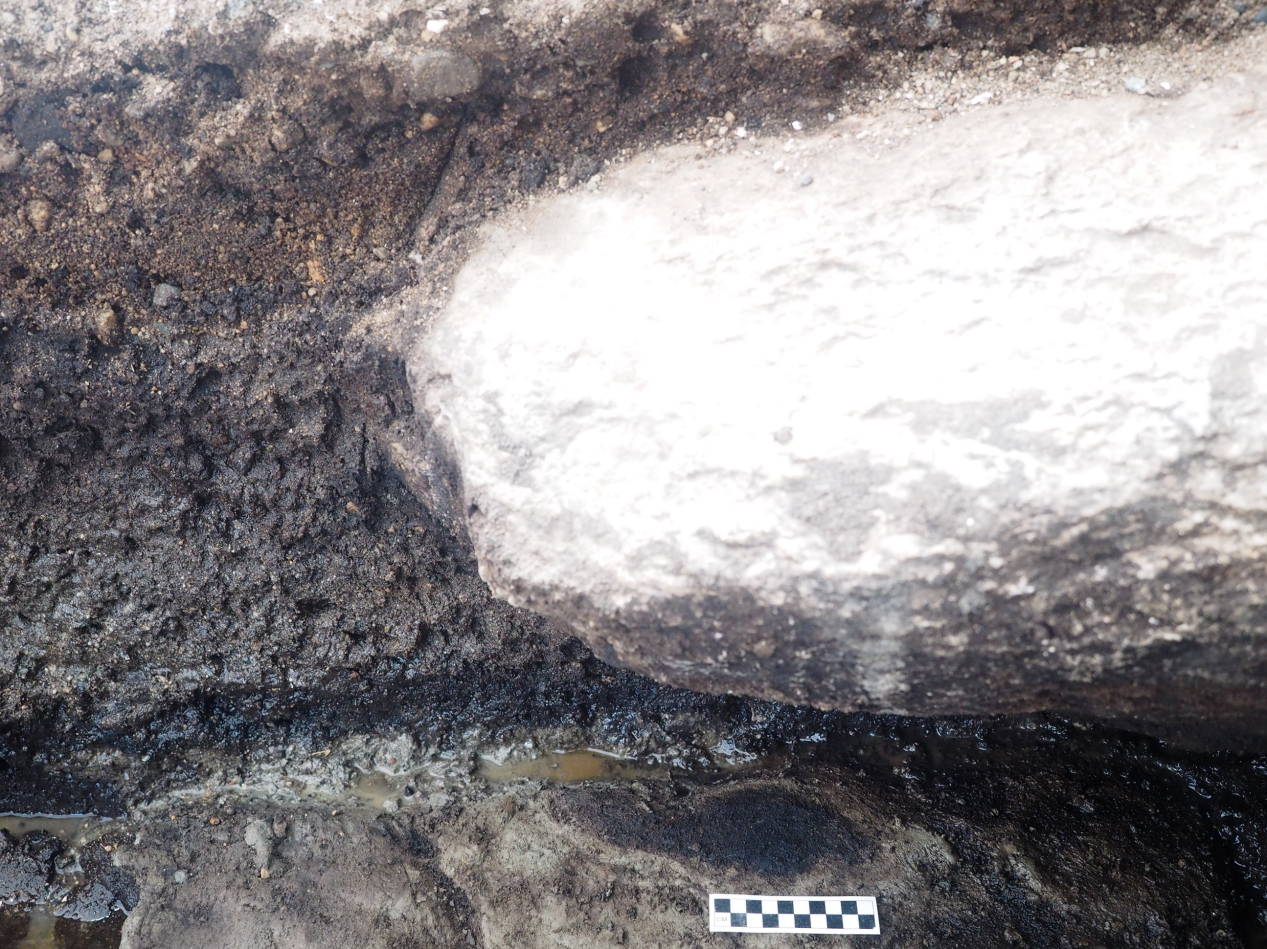
Photograph of track #15 under boulder. Arrow indicates outer end of sediment displacement rim. Photo by Duncan McLaren.

**Fig 9:**
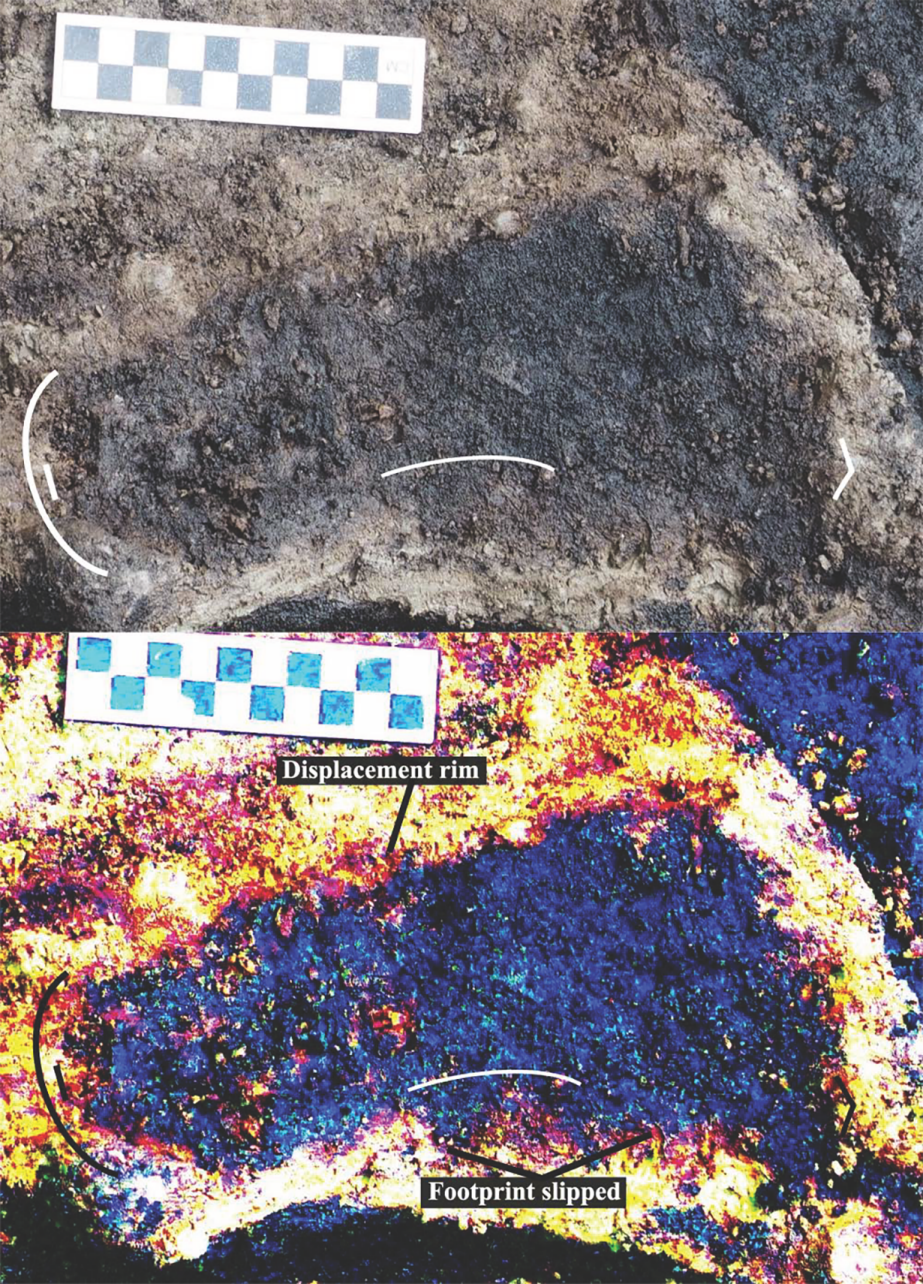
Photograph of track #20 with sediment displacement rim beside digitally enhanced image of same feature. Photo by Duncan McLaren.

**Fig 10:**
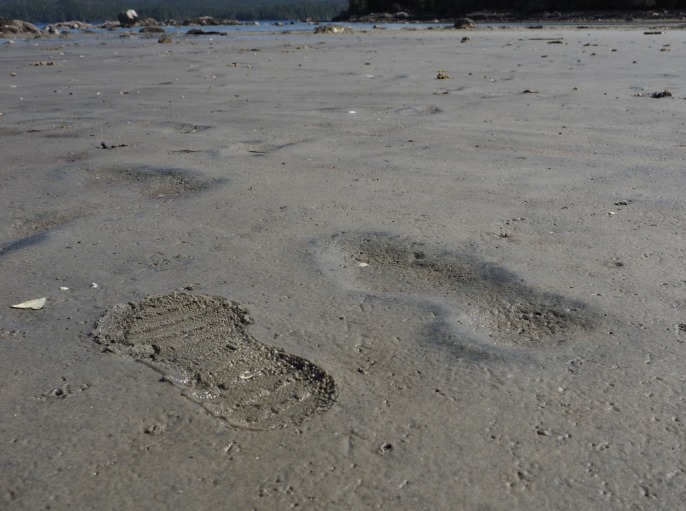
Two modern gumboot footprints in mid intertidal zone at EjTa-4. On right–one tide cycle old, note the displacement rim. On left–fresh track not yet subjected to high tide. Also, note the sandpiper tracks in the foreground. Photo by Joanne McSporran.

**Fig 11:**
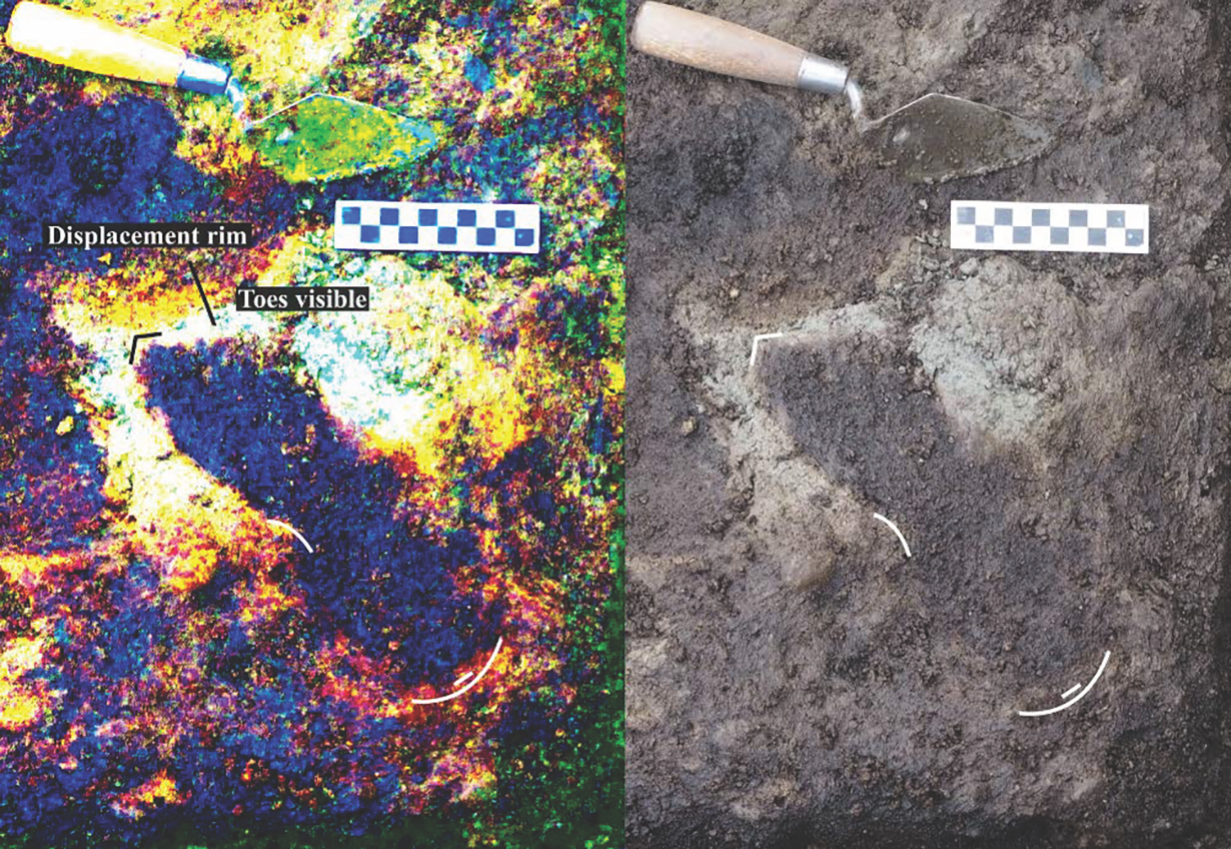
Track number #22 showing sediment displacement rim around distal end of the foot. Digital contrast adjustment (D-stretch) shows the heel and sediment displacement rim parts of the track clearly. Photo by Joanne McSporran.

In the 2015 and 2016 field seasons, we returned to the same location and opened up a 4 x 2 m excavation unit centered on the initial 50 x 50 cm test (Figs 5 and 6), revealing an additional 28 tracks oriented in different directions (Table 1). Some of these features were found to be clearly discernable with visible toe impressions (Fig 7), while others were less so. Several features had clear sediment displacement rims (Figs 8 and 9). Similar displacement rims form around tracks left on the intertidal surface today (Fig 10). Digital enhancement of the photographed features was found to be useful in visually representing the tracks (Figs 7, 9 and 11 and also see S1 File for additional images). In addition to the 29 individual footprints, we found evidence of other partial footprint-like depressions, but over-trampling had rendered them only barely discernible. For this reason, many could not be recorded in detail or with confidence and are not included in the total number of tracks discovered.

**Table 1:** Measurements and comments on footprints found at EjTa-4. Length is along the central axis and width refers to maximum width of the ball of the foot. Depth below datum is indicated as DBD.

**Field#**	**Lab #**	**Length (cm)**	**Width (cm)**	**DBD (cm)**	**Side of Foot**	**Comments**
**2015–1**	1	n/a	n/a	96	?	Footprint slipped obscuring measurement; partial print only
**2015–2**	2	25.5	13	87	Right	Toe marks visible
**2015–3**	3	26	12	88	Left	Toes partially obscured
**2015–4**	4	19	8.5	93	Left?	Very distinct with toe drag marks
**2015–5**	5	n/a	9.5	91	Left	Heel is missing; toe marks visible
**2015–6**	6	25.5	11.5	90	Left	Clear toe impressions
**2015–7**	7	15	7	96	Right	Small footprint
**2015–8**	8	20	9	97	Right	Faint
**2015–9**	9	20	9	98	Left	Faint but some toes visible
**2015–10**	10	25	n/a	89	?	Crossed with footprint #11
**2015–11**	11	20	n/a	89	?	Crossed with footprint #10
**2015–12**	12	25	10	90	Left	Big toe visible
**2016-2/3-1**	13	n/a	n/a	95	?	Only heel present
**2016-2/3-2**	14a	n/a	11.5	94	Right	Partial print; some toe marks
**2016-2/3-2**	14b	15	9	94	Right	Toe marks visible
**2016-2/3-4**	15	20	7	92	Right	Clearly defined rim and arch
**2016-2/3-5**	16	n/a	13	93	Right	Heel slip mark obscures length Some toe marks
**2016-2/3-6**	17	19	10	93	Right	Clear toe marks
**2016-2/3-7**	18	20	7	93	?	Faint
**2016-2/3-8**	19	n/a	n/a	93	?	Only heel visible
**2016-2/3-9**	20	21	10	94	Left	Clearly defined rim
**2016-8/9-1**	21	25	9.5	93	Right	Clear toes marks and arch
**2016-8/9-2**	22	21	8	93	Right	Clear rim, toes obscured by root bioturbation
**2016-8/9-3**	23	16	6	89	Right	Clearly defined rim
**2016-8/9-4**	24	21.5	n/a	90	?	Footprint in poor condition
**2016-8/9-5**	25	n/a	9	93	?	Heel slip mark obscured length, poor condition
**2016-8/9-6**	26	28.5	8.5	93	Left	Partial rim, toes obscured by root bioturbation, likely elongated by slippage
**2016-8/9-7**	27	22	8	98	Right	Clear toe marks
**2014–1**	28	n/a	n/a	n/a	n/a	Footprint noted only

Some bioturbation of these sediments by root penetration was evident in the northeastern most part of the excavation, further obscuring some of the tracks. Large bell-shaped pits were found cutting into the track surface at the southern end (seaward) of the excavation area (Fig 6). These were likely formed as natural upper intertidal paleo-pools that caught run-off from the slope above. Current day analogues to these pits were found in the upper intertidal near the site. As a result of this, most of the footprints found were situated in the northern part of the excavation area.

**Fig 12:**
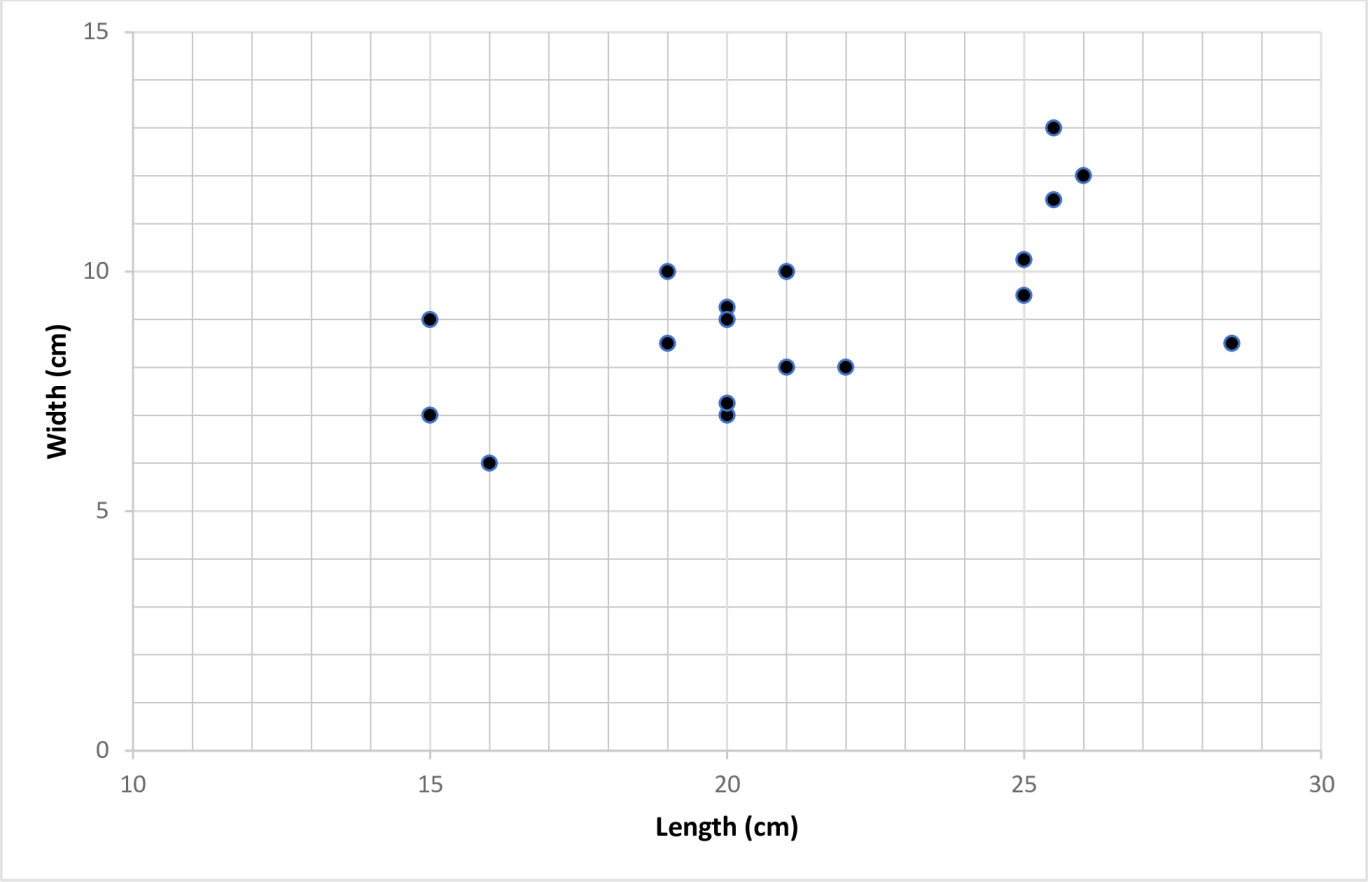
Scattergram comparing length and width for the 18 tracks with full measurements. Three clusters emerge revealing that the prints were left by at least three different individuals. Variation in track size is expected as a result of slippage, foot rotation, taphonomic factors and measurement error.

Measurement were taken to determine size of foot on 18 of the footprints found (Table 1). When plotted in a scattergram, the measurements fall into three distinct size clusters (Fig 12), suggesting that the tracks were left by a minimum of three individuals.

### Stratigraphy and dating

**Fig 13:**
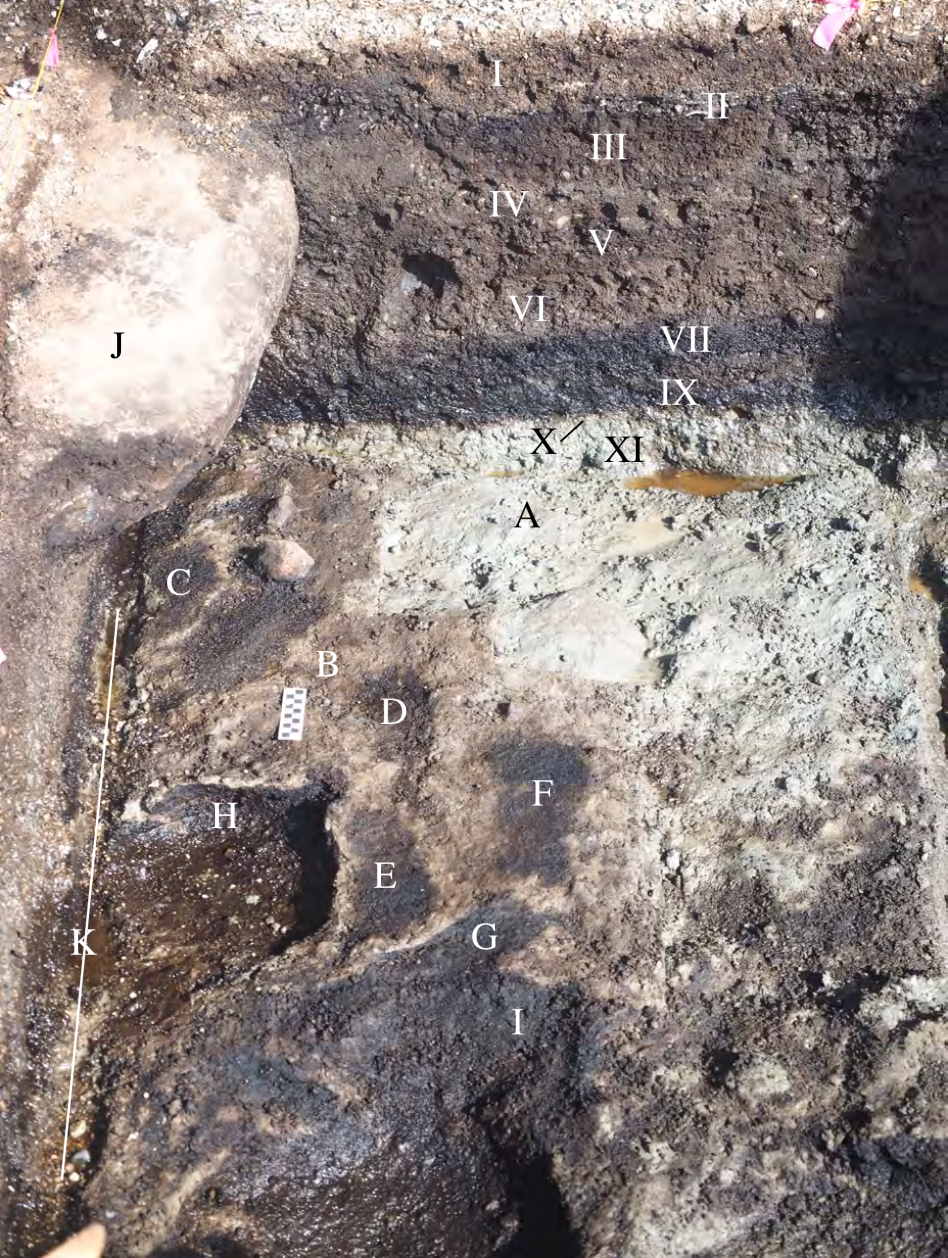
Photograph showing the relationship of site stratigraphy to the track surface. (A) dense grey clay (XI), likely glacial marine–predates 13,400 cal BP, (B) dense brown clay paleosol (X) with preserved wood dating between 13,317 and 12,633 cal BP, (C) track #15, (D) track #17, (E) track #20, (F) superimposed tracks 14a and 14b (G) track #13, (H) bell shaped pit, (I) bell shaped pit, (J) boulder deposited sometime after 3000 years cal BP, (K)–drainage trench to facilitate excavation. Note: Stratum VIII was not found in this part of the excavation. Photo by Duncan McLaren.

**Fig 14:**
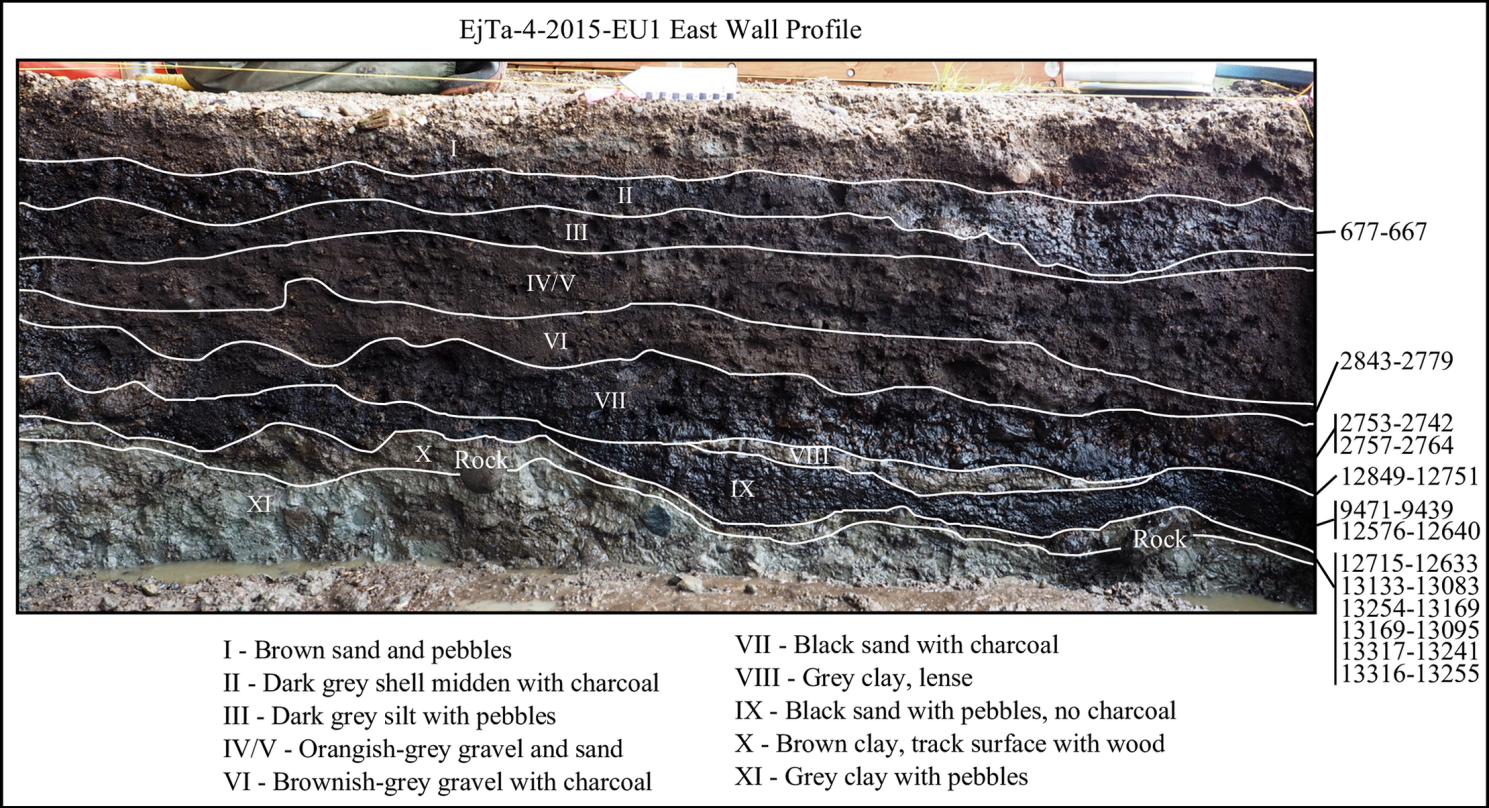
Illustration of strata and associated radiocarbon ages (cal BP). Only radiocarbon dates on identified plant remains and charcoal have been included. Base photo by Joanne McSporran.

Excavations in the intertidal zone at EjTa-4 revealed twelve strata within a metre of the surface of the beach (Figs 13 and 14). From bottom to top, the following strata and associated radiocarbon dates suggest the following depositional sequence.

**Fig 15:**
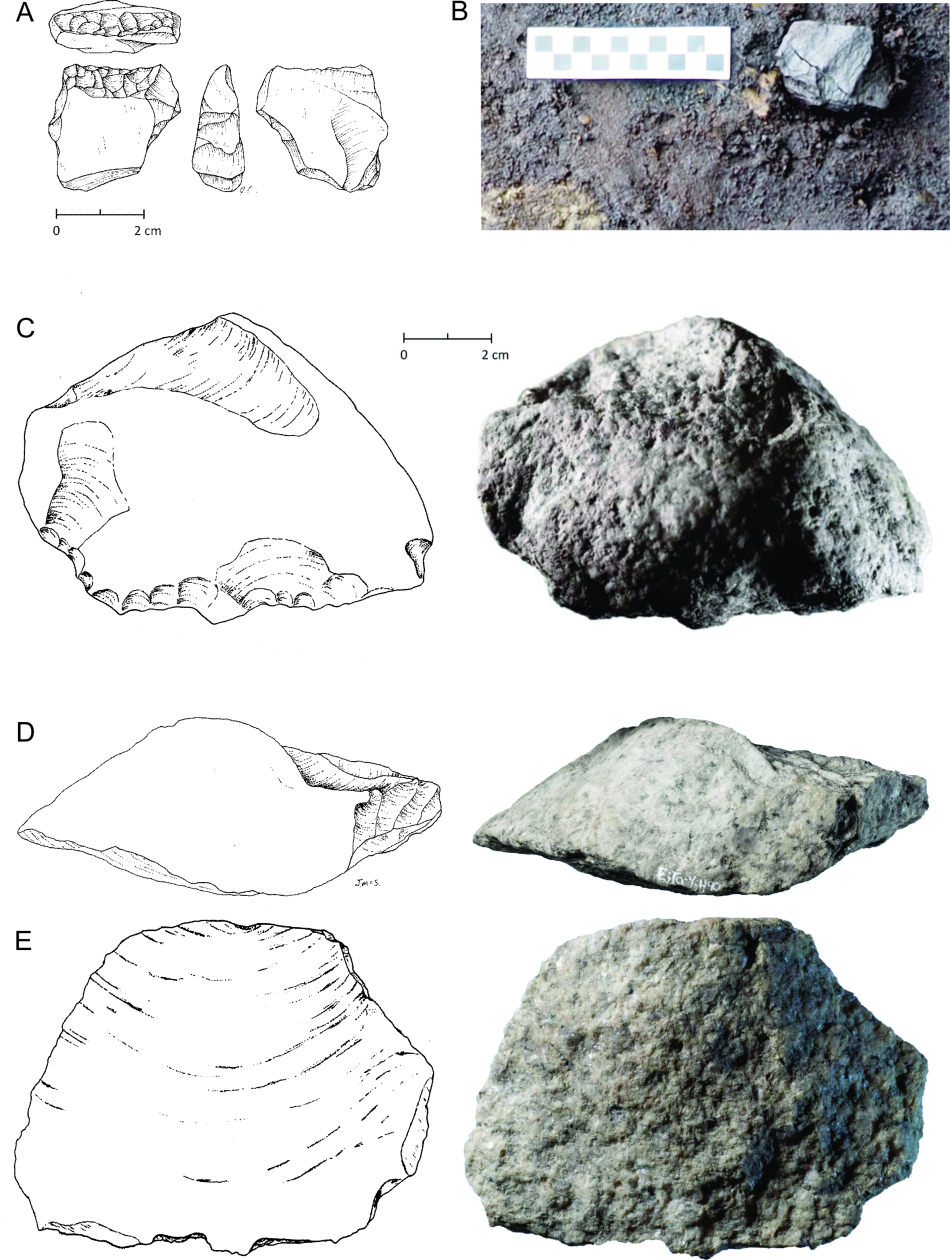
Examples of stone tools associated with Stratum X. (A) illustration of fined-grained quartzite retouched flake tool fragment, (B) photo of in situ medium-grained andesite core, (C) rough-grained quartzite retouched spall tool oblique view, (D) platform view, (E) ventral view. Illustrations by Jenny Cohen (A) and Joanne McSporran (C-E). Photo by Joanne McSporran.

The basal stratum (XII) is blue-grey clay with rounded clasts ranging in size from cobbles to pebbles, mostly locally abundant granito-diorite but with some exotic stones as well. The sediments suggest a glacier-proximal depositional environment, possibly glaciomarine. Above this lies a Stratum XI of grey clay lacking major clasts suggesting a lower energy glacial-proximal depositional environment, possibly the upper intertidal zone. Microscopically the sediment particles were noted to be sharp-edged and fossil micro-organisms were found to be lacking. The topmost part of the Stratum XI clay is uniformly stained light brown (X) and is interpreted as a paleosol. This paleosol exhibits evidence of soil development and contains abundant organic material, primarily small fragments of preserved *Pinus contorta* (shore pine) twigs dated between 13,317 and 12,633 cal BP (Table 2). A microscopic examination of the sediments found that they contained the presence of fossil terrestrial amoebae (*Nebella* and *Quadrulella* spp.), rounded (likely wind-blown) sponge spicules and infrequent and unidentifiable diatom fragments. This paleosol forms the track surface and was used to distinguish true track from over track sediments when excavating the footprint impressions, as the features are impressed into it (Fig 13). During excavation, this paleosol was first encountered at the top of the sediment displacement rims which could be traced out before the entire footprint was revealed. Most of the lithics found in association with Stratum X are fashioned from rough grained andesite and rough to medium grained quartzite. Other materials include fine grained andesite, quartzite and dacite. The stone tools found include twelve platform bearing flakes, fourteen shattered flakes, two cores, three small pebble tools, one retouched core tool, five retouched flake tools and a large retouched spall tool (Fig 15). Five of the rough grained flakes exhibit evidence of being water rolled.

**Table 2:** Radiocarbon ages, calibrations and stratigraphic relationships. All radiocarbon ages were calibrated using Calib 7.0.2 and the IntCal13 curve [65].

Stratum	Lab Code UCIAMS	Sample Code	Material	Description	14c Age	±	Younger Cal Range BP	Older Cal Range BP	Comment
II	186383	EjTa-4-2015-EU1 48–51 cm dbs L2	Charcoal	Column sample	720	15	667	677	Shell midden
VII	163742	EjTa4-2015-EU1 84-87cm L7	Charcoal	Column sample	2605	20	2742	2752	Charcoal rich
VI	163743	EjTa4-2015-EU1 79-85cm L6	Charcoal	Column sample	2715	20	2779	2843	Charcoal rich
VII	179712	EjTa4 EU3 FP9 95-96cm organics .21mgC	Unid plant material	Over track sediments, Track #20	2645	15	2751	2761	Small sample size
VII	179714	EjTa4 EU3 w/ artifact4 93-95cm	Charcoal	Sediments beneath sharp flake	2650	15	2764	2757	Charcoal rich
VII	169400	EjTa4-2015-EU1 94–95 cm dbd FP 1	Unid organic material–root	Over track sediments, Track #1	2855	15	2946	2998	No identifiable organics found in over track sediments
IX	169401	EjTa4-2015-EU1 95–97 cm dbd FP7 .12mgC	Unid organic material–root	Over track sediments, Track #7	4935	25	5608	5706	No identifiable organics found in over track sediments
IX	179713	EjTa4 EU3 FP2 95-96cm wood, seed	Malus seed + wood	Over track sediments Track 14a	8410	20	9439	9471	Pacific crab apple seed and wood in over track sediments
IX	169403	EjTa4-2015-EU1 106–110 cm dbd .056mgC	Unid organic material	Base of natural pit feature	9820	80	11171	11322	Small sample, likely root
IX	184916	EjTa4-2015-EU1 94 cm dbd	*Pinus contorta* wood	Base of stratum	10625	20	12576	12640	Provides upper limiting date for track surface
X	163740	EjTa4-2015-EU1 92-93cm FP10&11	*Pinus contorta* wood	True track #10&11	10720	60	12633	12715	Sampled from paleosol at base of impression
VIII	169399	EjTa4-2015-EU1 87cm dbs Stratum VIII	*Pinus contorta* wood	Interrill	10980	25	12751	12849	Provides upper limiting date for track surface
X	163741	EjTa4-2015-EU1 95-96cm	*Pinus contorta* wood	Track surface 2–6 cm south of Track #1	11260	25	13083	13133	Paleosol and track surface
X	142561	EjTa4-2014-B 134 cm dbd	Wood	True track #28	11295	30	13095	13169	Sampled from paleosol at base of impression
X	169402	EjTa4-2015-EU1 98–99 cm dbd near FP7	*Pinus contorta* wood	Track surface adjacent to Track #7	11365	25	13169	13254	Paleosol and track surface
X	149779	EjTa4 B/Stratum H 134 cm dbd	Wood	True track #28	11435	30	13241	13317	Sampled from paleosol at base of impression
X/XI	163739	EjTa4-2015-EU1 100-102cm L10 FP1	*Pinus contorta* wood	True track #1	11440	25	13255	13316	Sampled from paleosol at base of impression

**Fig 16:**
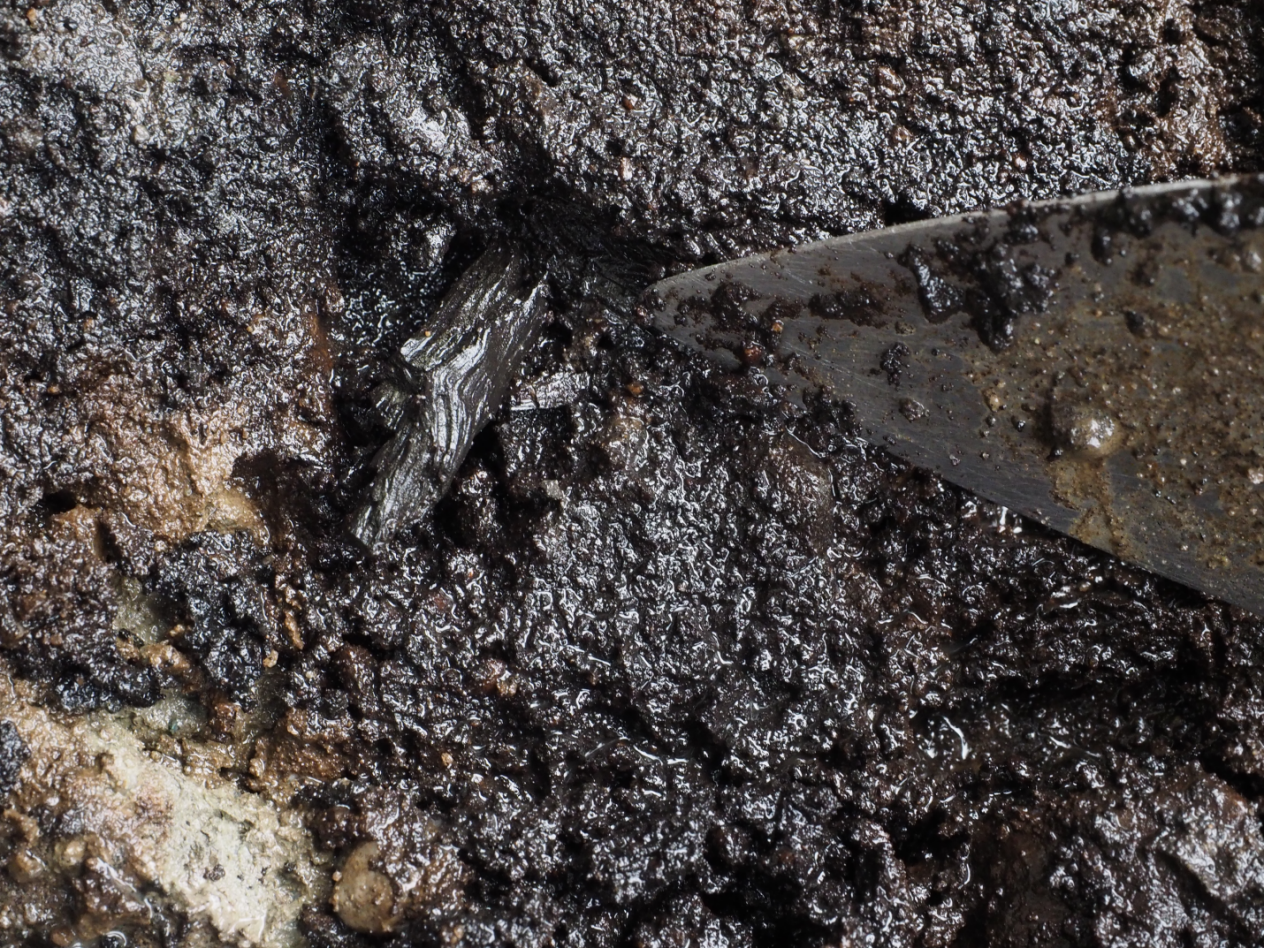
*Pinus contorta* twig sampled from the base of Stratum IX sediment which overlies the track surface. This wood was dated to 12,640–12,576 cal BP. The underlying light brown clay is the track surface (Stratum X) and under that is grey clay (Stratum XI).

A black sand matrix with rounded pea gravel (IX) overlays Stratum X and fills the footprints. The sediments associated with Stratum IX lack charcoal and contain sparse plant macrofossils. Fragments of sponge spicules and shattered diatoms fragments were found during microscopic inspection of sediments from this stratum, including *Coscinodiscus* sp. rim fragments, suggesting marine influence in the depositional processes. Radiocarbon ages from Stratum IX are internally inconsistent ranging from: 12,640–12,576 cal BP (UCIAMS 184916) (Fig 16) to 5706–5608 cal BP (UCIAMS 169401) (Table 2). These dates reveal a lack of congruity, likely as a result of bioturbation, though these ages are more recent than those from Stratum X. Two date samples from this stratum were run on unidentifiable and highly decayed plant material, as little in the way of dateable material was found *in situ* or in the associated sediment samples taken. A feature described as a pit hearth in the field was encountered in the southeastern part of the excavated area. It is outlined by rocks on the western side of the pit and filled with black sand and pea gravel. Despite the characterization of this feature as a hearth, no charcoal or other dateable material was found in the bulk sediment samples taken. The lack of identified charcoal may be related to these sediments having been exposed to alkaline ocean waters for prolonged periods [64]. Three pieces of wood were identified as *Pinus contorta* from the interface between Strata X and IX. Lithics associated with Stratum IX are made from rough grained andesite. Two water rolled flakes, one flake tool and a shattered flake fragment were found in Stratum IX.

Grey clay lenses (Stratum VIII) discontinuously overlay the black sand. Wood sampled from these lenses dates to 12,849–12,751 cal BP (UCIAMS 169399), and eight flakes were found in the stratum. This clay has possibly resulted from redeposition of material associated with Stratum XI. After it was deposited, the discontinuous character of this stratum suggests that at least a part of it was eroded away by rill erosion, with the remaining patches of clay being left as interrills. This stratum clearly overlies the track surface. The age range of 12,849–12,751 cal BP is considered to be a conservative upper date range for the footprints. Wood samples from Strata VIII were identified as *Pinus contorta*.

A stratum of black sand and charcoal (Stratum VII) covers both the rills and interrills. The sediments associated with Stratum VII were deposited after the rill erosion event that effectively created a disconformity. Other than the presence of charcoal in this stratum, it was difficult to distinguish it from Stratum IX during excavation, particularly when not separated by Stratum VIII. Samples of charcoal in Stratum VII sediment have been dated to 2,757–2,764 cal BP (UCIAMS 179714) and 2,752–2,742 cal BP (UCIAMS 163742). Three pieces of wood from this stratum were identified as alder (*Alnus* sp.), Sitka spruce (*Picea sitchensis*), and conifer (likely *Abies* or *Tsuga* sp.), all of which are part of the forest cover found in the region today. One abrader fragment, a core and seven flakes were found in Stratum VII. Additional flakes, two flakes tools, a pebble tool, a core and another abrader fragment came from either Strata VII or IX due to the difficulty in distinguishing the two. With the beginning of the deposition of Stratum VII sediments, a high tide beach berm began to form with inputs of sand and gravel being tossed on it through wave action.

Stratum VI is a grey pea gravel that covers the black sand gravels below. At least some of this stratum is slumped and redeposited shell midden as indicated by abundant periostracum, water rolled lithics and faunal remains. Charcoal from this stratum dates to 2,843–2,779 cal BP (UCIAMS 163743). Associated artifacts include a ground bone bead, 22 flakes, four cores, a core tool, three pebble tools, three flake tools and a hammerstone.

Covering Stratum VI is a series of sand and gravel strata (IV/V) deposited as the beach berm continued to build. No artifacts were found in Stratum V, and only one artifact was found in Stratum IV: a ground and chipped stone tool resembling an awl. The sand and gravel blacken upwards and has a greater organic component (Stratum III) before becoming a shell midden deposit dating to 677–667 cal BP (UCIAMS 186383), possibly redeposited (Stratum II). Stratum III contained three flake tools, while the interface between Strata II and III contained one flake tool and two flakes. Stratum II contained 12 flakes, five flake tools, one shattered flake, and a ground stone celt fragment. Overlying the deposit of shell midden is a stratum of loose beach sand (Stratum I) that makes up the current beach deposits and surface. No artifacts were found in Stratum I, but numerous artifacts have been noted on the surface of the beach.

### Site formation processes

**Fig 17:**
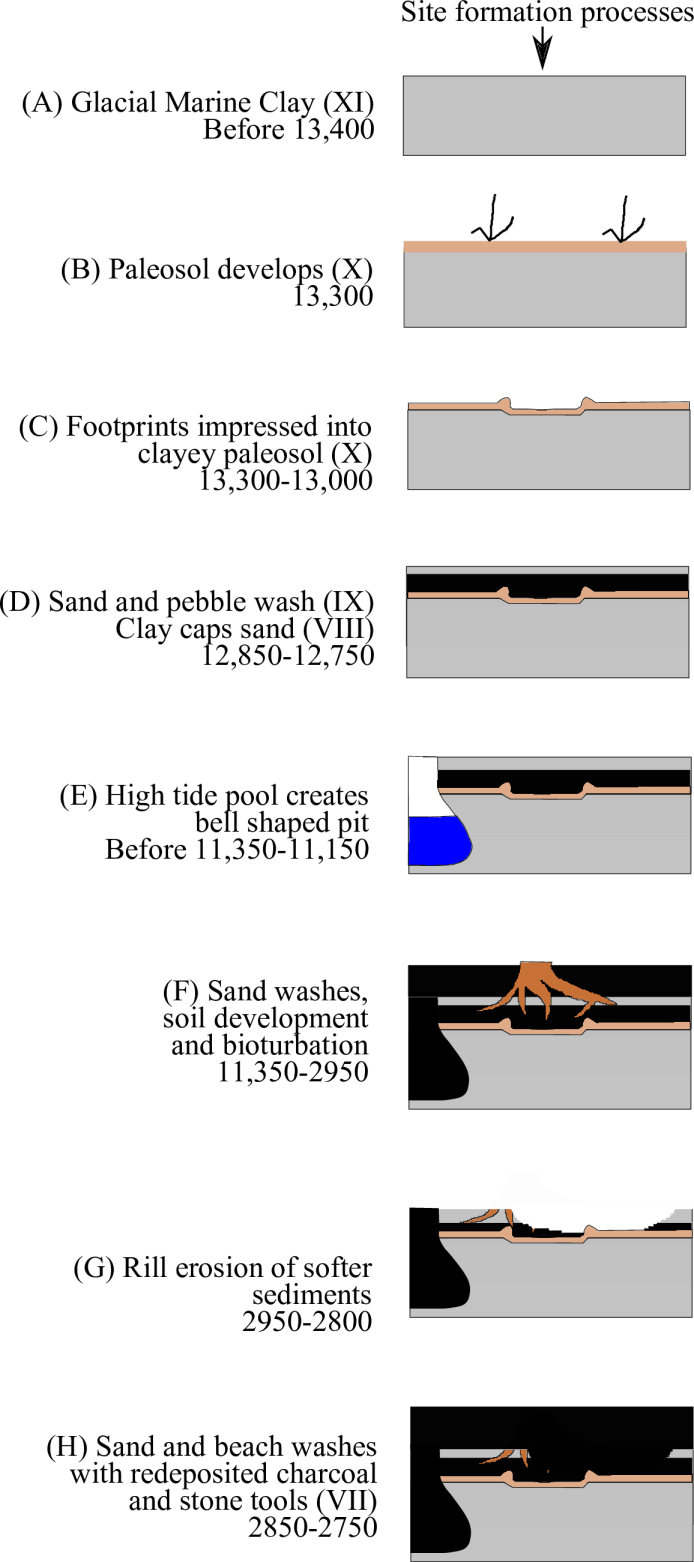
Illustration of site formation processes based on stratigraphy and radiocarbon dates.

**Fig 18:**
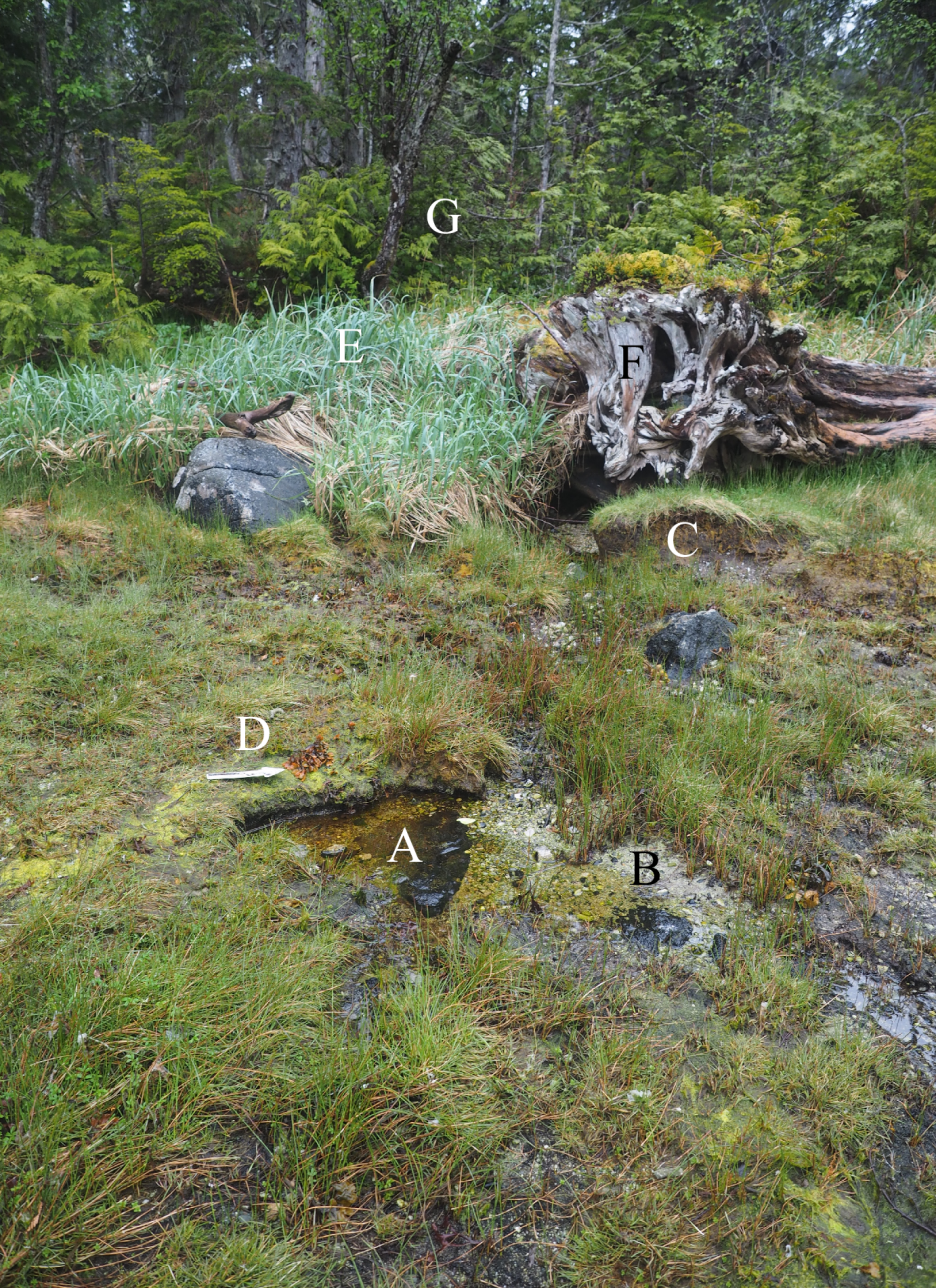
High intertidal pool near EjTa-4 and present-day features of the upper intertidal zone analogous to those described in the site formation processes for Strata IX—VI. (A) upper intertidal bell-shaped pool, (B) rill erosion caused by run-off, (C) steeply eroded interrill, (D) 20 cm scale, (E) dunegrass on top of high tide beach berm, (F) driftwood rootball, (G) western redcedar (*Thuja plicata*) forest. Photo by Duncan McLaren.

Based on observations in the field, the analysis of sediments, and radiocarbon date results, the following site formation processes are interpreted to have occurred (Fig 17). At the end of the last glacial event, a glacial proximal marine clay (Stratum XI, Fig 17A) was deposited between 14,500 and 14,000 cal BP when sea level was higher than today [40]. Sea level dropped below present level after 14,000 cal BP, and by 13,300 cal BP soil had developed on top of the clay (Stratum X, Fig 17B) (Fig 2). This clayey paleosol formed the track surface and wood scattered on the track surface was pressed into the base of the tracks by footsteps (Fig 17C). The track surface was then capped by washes of sand and pebbles (Stratum IX) and then another stratum of clay (VIII) sometime before 12,850–12,750 cal BP (Fig 17D). Between this time and 11,350 cal BP, the track surface was in the upper intertidal zone where pools formed (Fig 17E) resulting in the bell-shaped pits that interrupt the track surface in the southern part of the area excavated (see Fig 18 for a modern analogy). These pits were then filled with washes of sand and gravel. Between 11,350 and 2,950 cal BP, forest soils developed over the site leaving root casts (Fig 17F). Sea level was likely higher than the elevation of the footprints by at least a metre for a part of this time, in particular around 5,000 cal BP (Fig 2)[40], but the deposits were likely protected from erosion by the bulk of sediments that had developed as a berm and forest soils over the track surface. Rill erosion removed some of the sediments overlying the track surface between 2,950 and 2,800 cal BP (Fig 17G). This action created a partial unconformity. Later, the rills were filled and capped with charcoal rich sand and pea gravel (Strata VII–VI, Fig 17H). This erosion does not appear to have affected the Stratum X paleosol; that remains for the most part continuous being clay and much harder than the overlying loose sand and pebble dominated sediments. Deposition after this time resulted in the building up of additional sand, gravel and redeposited shell midden strata in a beach berm that has continued to accumulate up until the present day (Strata V—I).

## Discussion

Based on the clear arch, toe and offset heel attributes of the tracks found during excavations at EjTa-4 we are certain that they were left by human feet. Of the large land mammals that inhabit the area today, only the hind paw of black and grizzly bears is similar to human tracks [66]. Although rare on Calvert Island, bears are common on the coast of BC. Our field crew members are very familiar with bear footprints and keep a look out for them, to know if there is a bear in the area, for making decisions related to worker safety. The tracks excavated on Calvert Island have a clearly defined arch, lack characteristic claw marks, are not triangular in overall shape (rather the heels are offset to either the left or right), lack a long third phalanx (rather the first or second phalanx is longest), and they are overall narrower than bear tracks. In addition to the lack of bear hind tracks, no bear fore tracks were excavated. Overall, non-human tracks of any kind are lacking from the area that was excavated. However, the track surface is a paleosol which extends beyond the periphery of our excavations and it is possible that non-human tracks would be found were excavations to be extended horizontally.

Variation of the footprint length and width measurements may be in part due to the slippery clay in which they were impressed [67]. Regardless of this, three measurement clusters corresponding to three different size classes suggest that there were at least three different individuals who left the footprints (Fig 12). Using a Brannock device measurement chart (a device commonly used to measure feet [68]), the footprint measurements correspond to modern day US shoe sizes of a junior size 8, a junior size one (or a woman’s size 3), and a woman’s size 8–9 (or man’s size 7–8).

**Fig 19:**
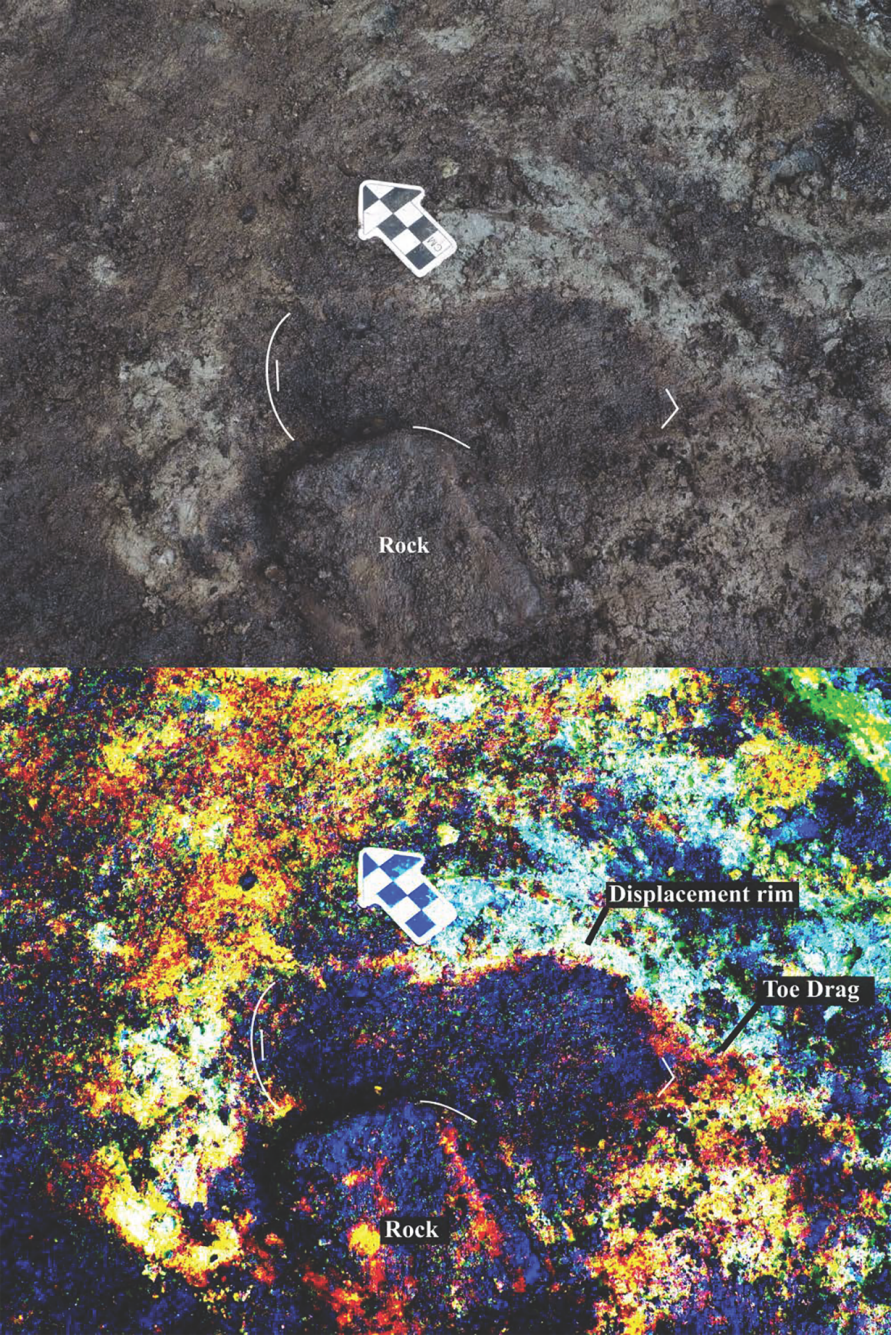
Photograph showing track #4 which has discernable toe drag marks. This track was later pedestalled and removed to the lab. (Photo by Joanne McSporran).

**Fig 20:**
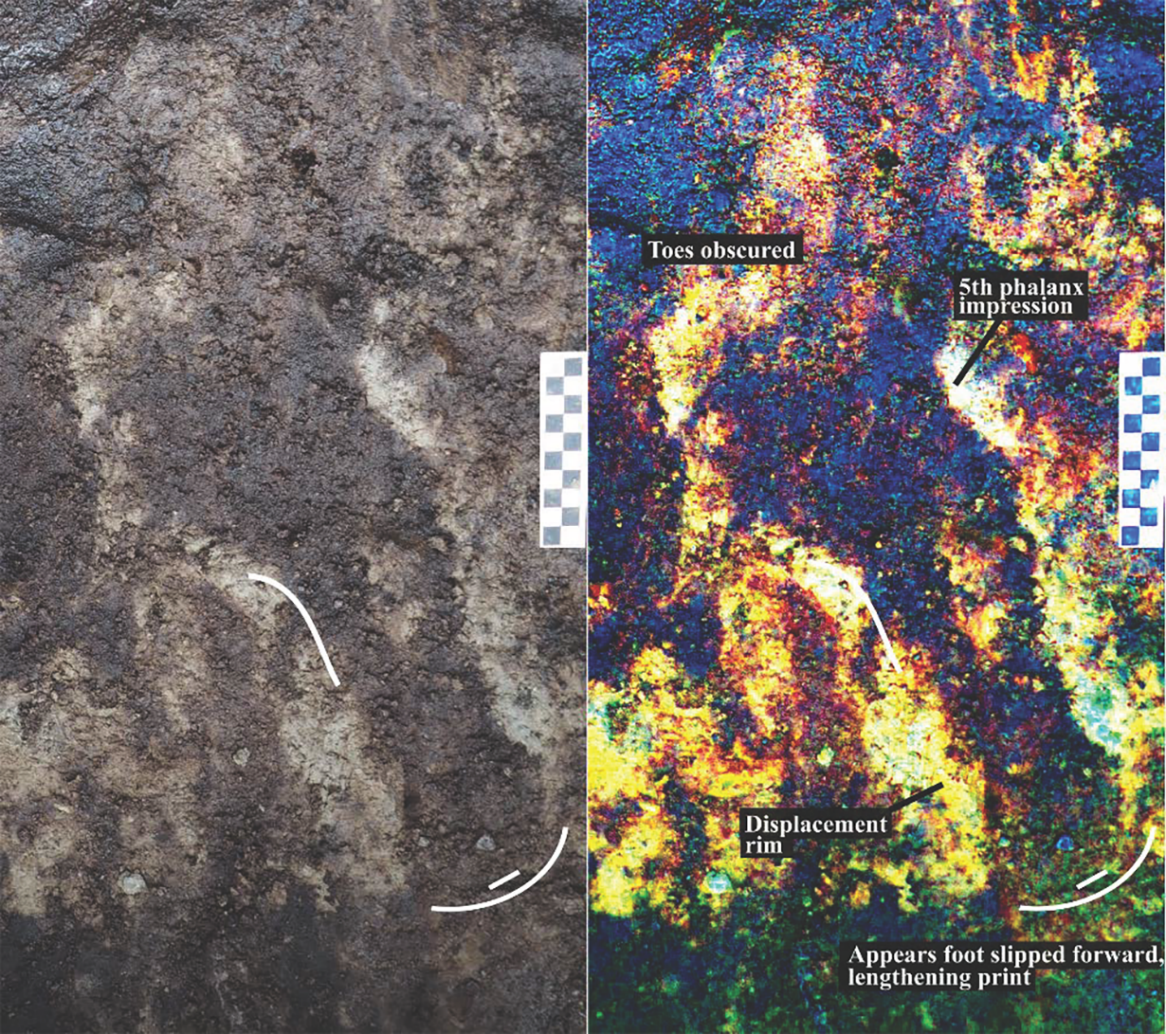
Photograph showing track #26 with an elongated heel as a result of the pedestrian slipping forwards while walking in the clayey substrate (Photo by Joanne McSporran).

In some instances, toe marks were clearly visible in the track impressions. This suggests the likelihood that those who left them were not wearing shoes. In the case of a few of the tracks, no toe prints were visible, and it was speculated during excavation that this may be the result of some type of shoe being worn. This is particularly the case for Footprint #4 which is one of the clearest examples and yet lacked clear toe marks (Fig 19). However, by digitally enhancing photos of this footprint there are clear first and second phalanx forward drag marks and for this reason it is unlikely that the foot that made this impression was shod. In another case (Fig 20) an elongation of the heel of the foot was found, likely caused by the pedestrian slipping forward in the substrate as they walked across it.

**Fig 21:**
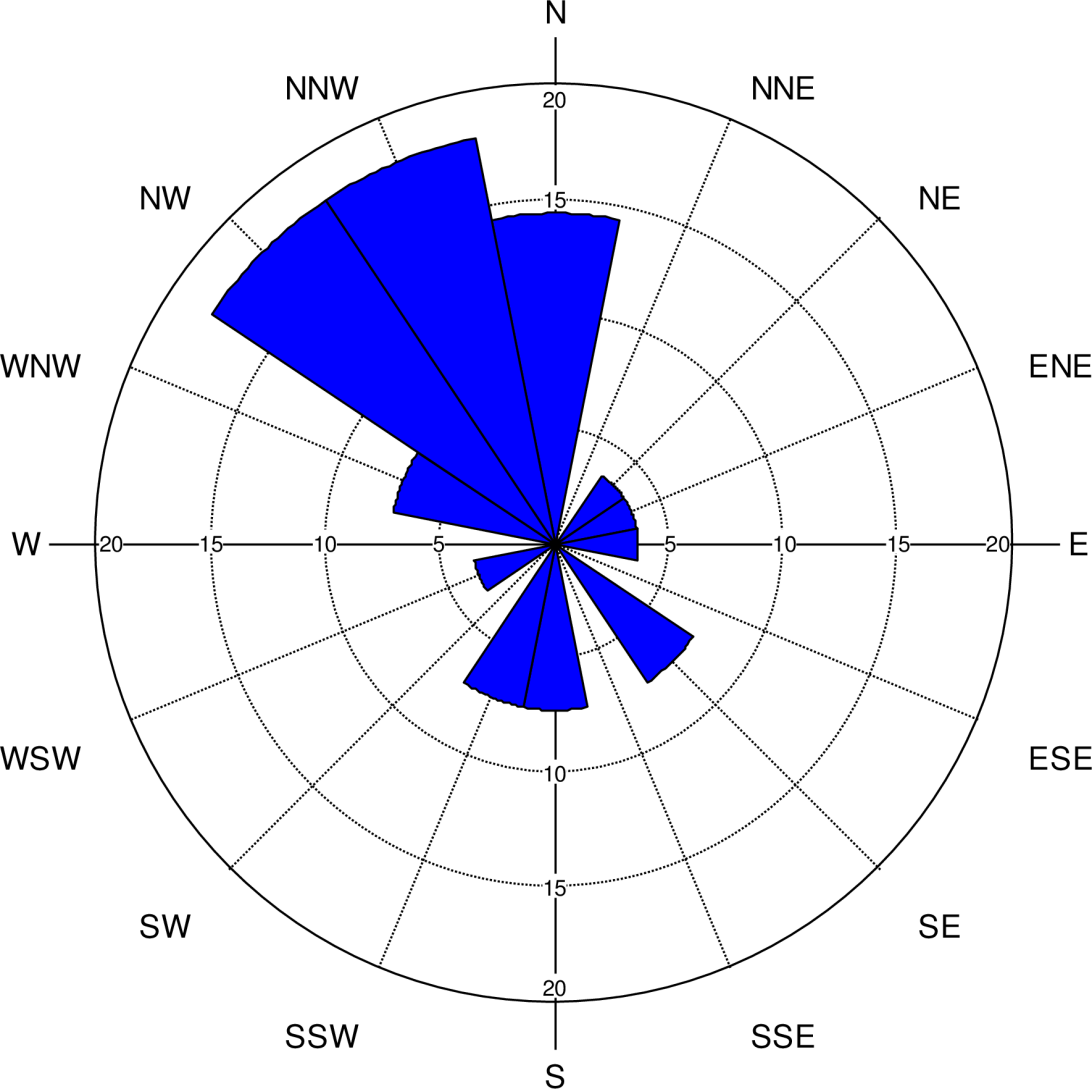
Diagram showing the relative percentage of track azimuth measurements. It is clear from this diagram that those who left the tracks were primarily facing and/or moving in a north to northwesterly direction.

While some famous footprint sites are trackways (linear path of consecutive footprints) [e.g. 7,69], the assemblage from Calvert Island is not linear. The footprints from EjTa-4 more likely represent a congregation site as defined by Morse et al. [70]. This type of pattern results from people concentrating their activities in an area and may be centered around a focal point. Many more partial track-like depressions were discerned during excavations at EjTa-4 than were recorded as definite footprints. These unrecorded features were too obscured by repeated over-trampling to definitely identify as human tracks. For this reason, the footprints reported on here are only those which could be isolated, excavated and clearly demonstrated to be human tracks. By measuring the azimuth of each of the footprints identified, a rose diagram was created which demonstrates that the individuals that left the tracks were predominantly facing in a north to north-westerly direction, up the beach to the vegetation line (Fig 21). Footprints 8 and 9 (Fig 6) are side-by-side left and right footprints suggestive of someone standing with their feet slightly apart and facing northwestwards and inland with their back to the prevailing winds. The footprints were impressed into a soil just above the paleo-shoreline, possibly by a group of people disembarking from watercraft and moving towards a drier central activity area to the north or northwest.

Paleo-environmental studies reveal that glacial refugia capable of supporting large land animals and plants existed in different places on the outer Pacific coast of Canada and southeastern Alaska between 20,000 and 13,000 years ago [23–28]. However, archaeological evidence of human occupation from the same period and geographic region is lacking. The footprints from Calvert Island provide evidence of a human presence on the western margin of the Cordilleran Ice Sheet during the late Pleistocene period, 13,300–13,000 years ago. This time frame is slightly later than of the age of the Manis Mastodon site, situated at the southern end of the area that was influenced by the Cordilleran Ice Sheet [35]. It is anticipated that further targeted research using high-resolution sea level histories on the coast will reveal evidence sufficiently old enough to account for the 14,500 year old deposits found at Paisley Cave [41] or Monte Verde [43]. While the tracks are contemporaneous with the early Clovis complex, which dates between 13,100 and 12,700 cal BP [71], Clovis points have not been found on the coast of BC north of the lower Fraser Valley [72]. It is possible that the late Pleistocene inhabitants of the western margins of the Cordilleran Ice Sheet did not use Clovis technology but rather employed unfluted projectile points related to the Western Stemmed Tradition [41,73].

The paleo-environmental information from northern Calvert Island provides a context that supports our assertion that the footprints date to the late Pleistocene epoch. Evidence from eroding sedimentary exposures on the northwest side of Calvert Island reveal that it was not glaciated 15,000 cal BP [74]. After this time the northern part of the island was subject to a short local glacial advance from the slopes of Mount Buxton that ended 14,500 cal BP. This is consistent with our findings of a glacial clay at the base of our test at EjTa-4. Following this, there is evidence for the start of forest succession. Early post-glacial forest succession is characterized by a dominance of *Pinus contorta* trees [75]. Wood identified and dated from the tracks and track surface is consistently *P*. *contorta* and associated with a paleosol.

Human trackways are rarely discovered by archaeologists. Pleistocene/Holocene transition human tracks have been reported on from three distinct places in the Americas. At the Pehuen Co site in Buenos Aires Province in Argentina, human tracks were found on sandstone platforms in the intertidal zone [76]. These are associated with an impressive array of tracks left by extinct Pleistocene-age mammals dating to 14,000 cal BP. A single footprint was found and recorded at the Monte Verde site in Chile which dates to approximately 14,600 cal BP [77]. Two human trackways left in tuff at Cuatrociénegas site in Mexico have been dated to 10,700 cal BP and 7,200 cal BP [78]. A set of 40,000-year-old human tracks from Central Mexico has been reported on [79], but it has since been shown that these features were probably not created by ancient humans but by recent quarrying activity [70]. There are several mid to late Holocene archaeological sites with human tracks reported on in North America north of Mexico [80]. The Holocene track sites come from a variety of contexts including caves, riparian areas, and a pithouse floor. It is likely tracks are a component of many archaeological sites but are missed or not recognized as a result of their overall ephemeral character.

In the practice of archaeology there are multiple levels of evidence indicative of past human activity. Late Pleistocene sites in the Americas have often undergone intense scrutiny, in particular where the highest levels of evidence, for example human skeletal remains or diagnostic bifacial projectile points, are not present [81]. Alternatively, others have presented lower qualities of evidence, such as scratched or flaked animal bones, as bona fide evidence for the early human occupation of the Americas [82,83]. In this context, it becomes important to at least acknowledge and reflect on the level and quality of evidence being put forth [84,85]. The features discovered in association with Stratum X at EjTa-4 are clearly human tracks. Features which allow for the identification of these tracks include sediment displacement rims surrounding human-track shaped depressions with aspects of foot morphology including heels, arches, balls and, in some cases, individual toes. *Pinus contorta* wood is associated with both the base of the track impression and the track surface. Bracketing dates for the twig wood found in Stratum X and associated with the human tracks range from 13,317 to 12,633 cal BP. According to palynological studies from a nearby pond, *Pinus contorta* pollen accounts for 50% of the assemblage between 14,000 and 12,000 years ago, although it is also found in lesser abundance in later time periods [75]. Stratum IX, which overlies the track surface, is less securely dated, ranging from 12,640 to 5608 cal BP. This particular stratum has been compromised by root bioturbation and rill erosion. Stratum VIII which overlies uneroded sections of Stratum IX dates between 12,849–12,751 cal BP. The oldest date from Stratum IX and the date from the overlying Stratum VIII are similar and provide the upper constraining age for the footprints. Combined, the stratigraphic, paleo-environmental and sea level-based evidence supports the conservative date range of 13,317–12,633 cal BP for the footprints. A limitation of the evidence presented here is that outliers exist, in particular in Stratum IX which covers the track surface and which has undergone bioturbation and erosion in places. Based on our observations of the tracks and the results presented here we maintain that the quality of evidence for late Pleistocene occupation at EjTa-4 is robust and adds to the growing body of information on the early human occupation of the formerly glaciated coastal areas of northern North America [35,36,44]. It is very likely that additional tracks exist in areas surrounding the excavations conducted to date. We have elected to stop excavations at the site so that any remaining tracks remain undisturbed so that future researchers may be able to attempt duplication of the results presented here with more advanced methods.

## Conclusion

During subsurface testing directed toward uncovering late Pleistocene archaeological deposits, human footprints were discovered beneath active beach deposits in front of the Meay Channel I archaeology site (EjTa-4). Further investigation, through careful excavation, revealed a total of 29 human tracks in an area measuring 4 x 2 metres. It is likely that many more tracks exist in the surrounding and unexcavated sediments. Based on the length and width of these features, they appear to have been left by a minimum of three people, including one juvenile. Many more footprints were present but could not be accurately discerned or measured as a result of over-trampling. Preserved wood found on the track surface and pressed into the bottom of the tracks is identified as *Pinus contorta*. Radiocarbon ages on the wood sampled from the track surface and the bottom of the track impressions consistently date between 13,317 and 12,633 cal BP. The footprints found at EjTa-4 add to the growing body of evidence that humans inhabited the Pacific coast of Canada during late Pleistocene times.

## Supporting information

S1 File Supporting information–additional footprint photographs from EjTa-4. McLaren et al. 2018 S1 File.pdf.
(PDF)
