# Supplementary material for: Terminal Pleistocene epoch human footprints from the Pacific coast of Canada
Source: PLoS One. 2018 Mar 28;13(3):e0193522. doi: 10.1371/journal.pone.0193522 (PMC5873988; doi:10.1371/journal.pone.0193522)

S1 File for Terminal Pleistocene epoch human footprints from the Pacific coast of Canada

Duncan McLaren[1,2,*], Daryl Fedje[1,2], Angela Dyck[1,2], Quentin Mackie,[2] Alisha Gauvreau[1,2] and Jenny Cohen[2]

[1]Hakai Institute, Calvert Island, British Columbia, Canada

[2]University of Victoria, Victoria, British Columbia, Canada

Supporting Information – Additional Footprint Photographs From EjTa-4

The photos in this collection were taken by Joanne McSporran, Duncan McLaren and Iain McKechnie.

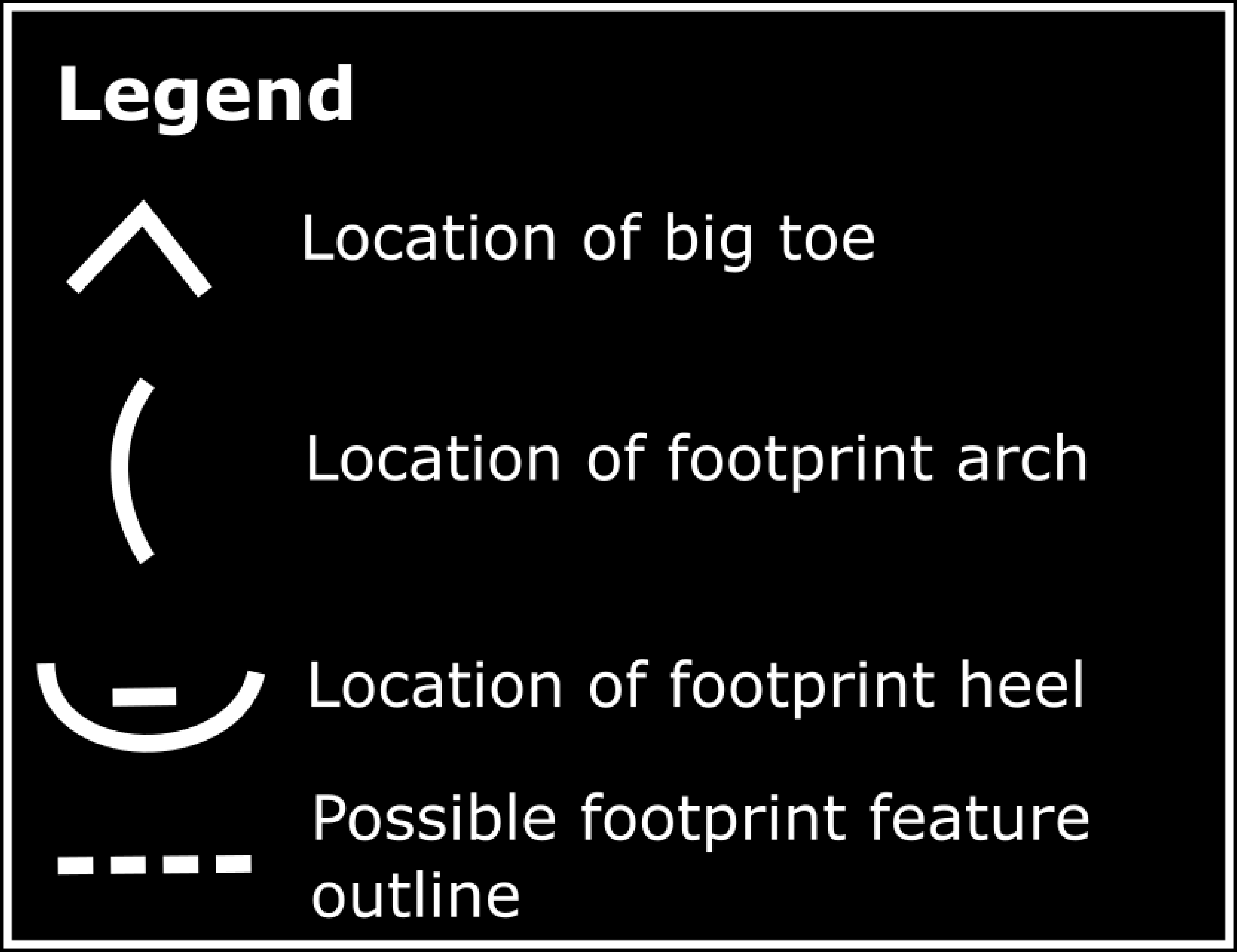
Legend
Location of big toe
Location of footprint arch
Location of footprint heel
Possible footprint feature outline

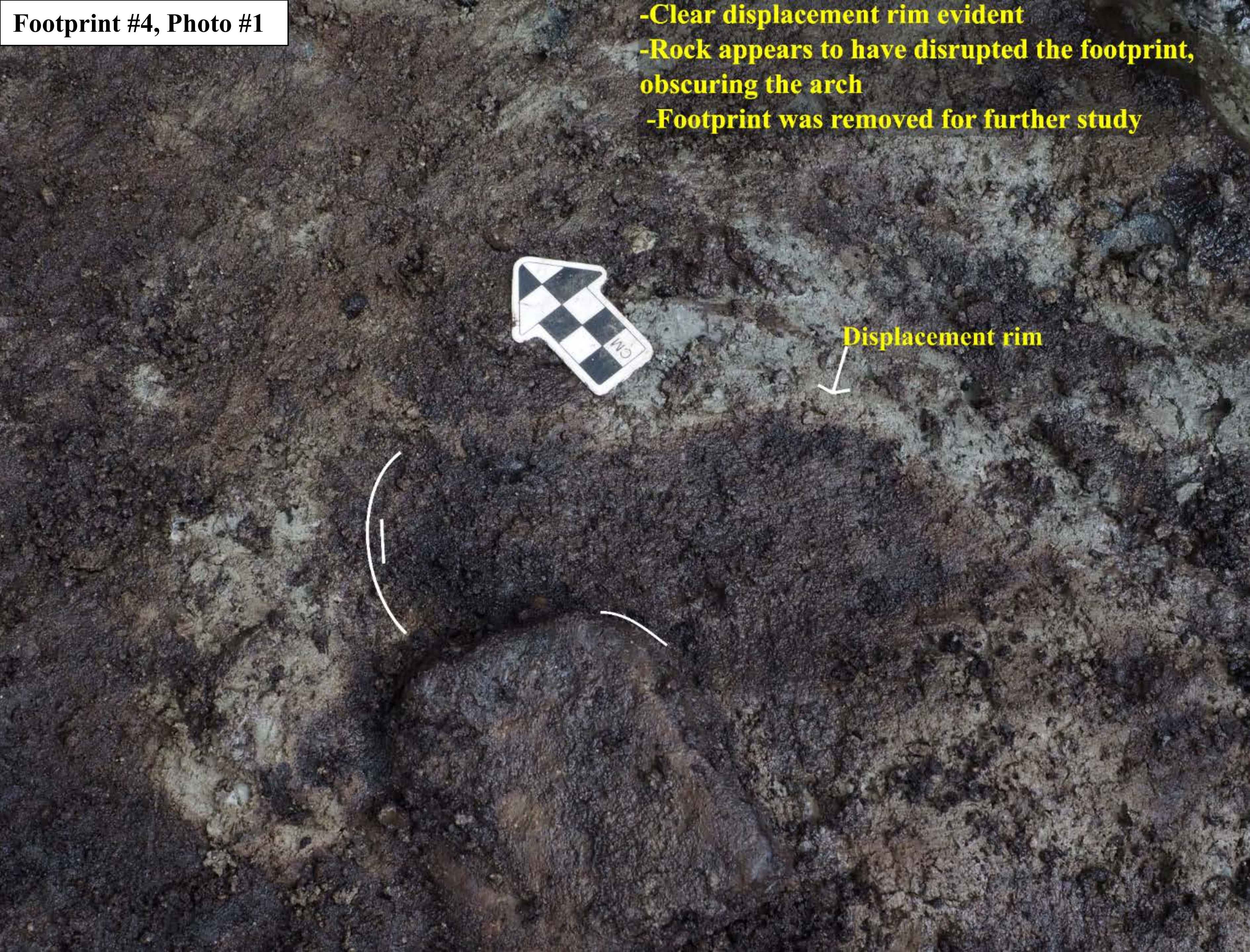
Footprint #4, Photo #1
-Clear displacement rim evident
-Rock appears to have disrupted the footprint, obscuring the arch
-Footprint was removed for further study
Displacement rim

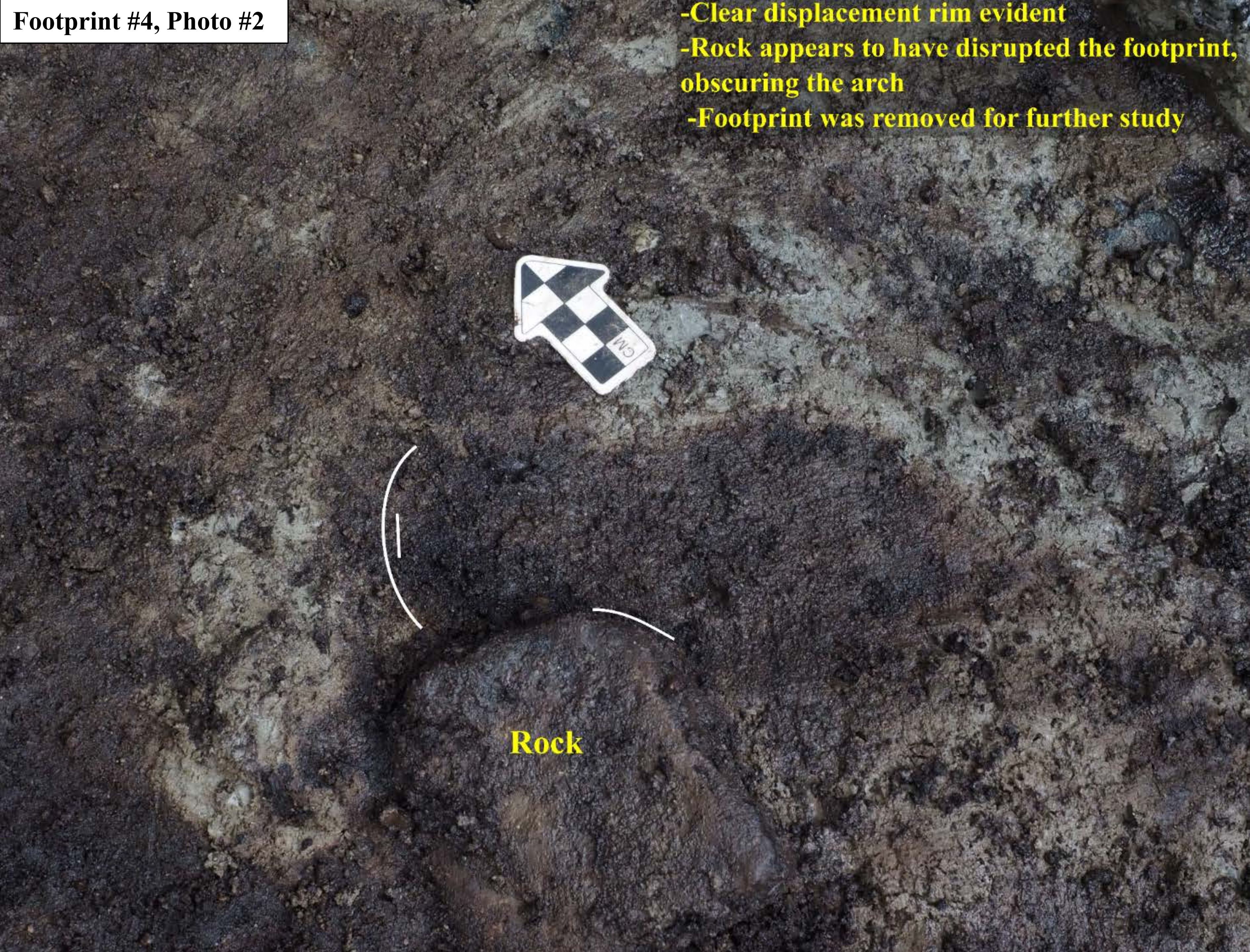

Footprint #4, Photo #2

Footprint #4, Photo #3

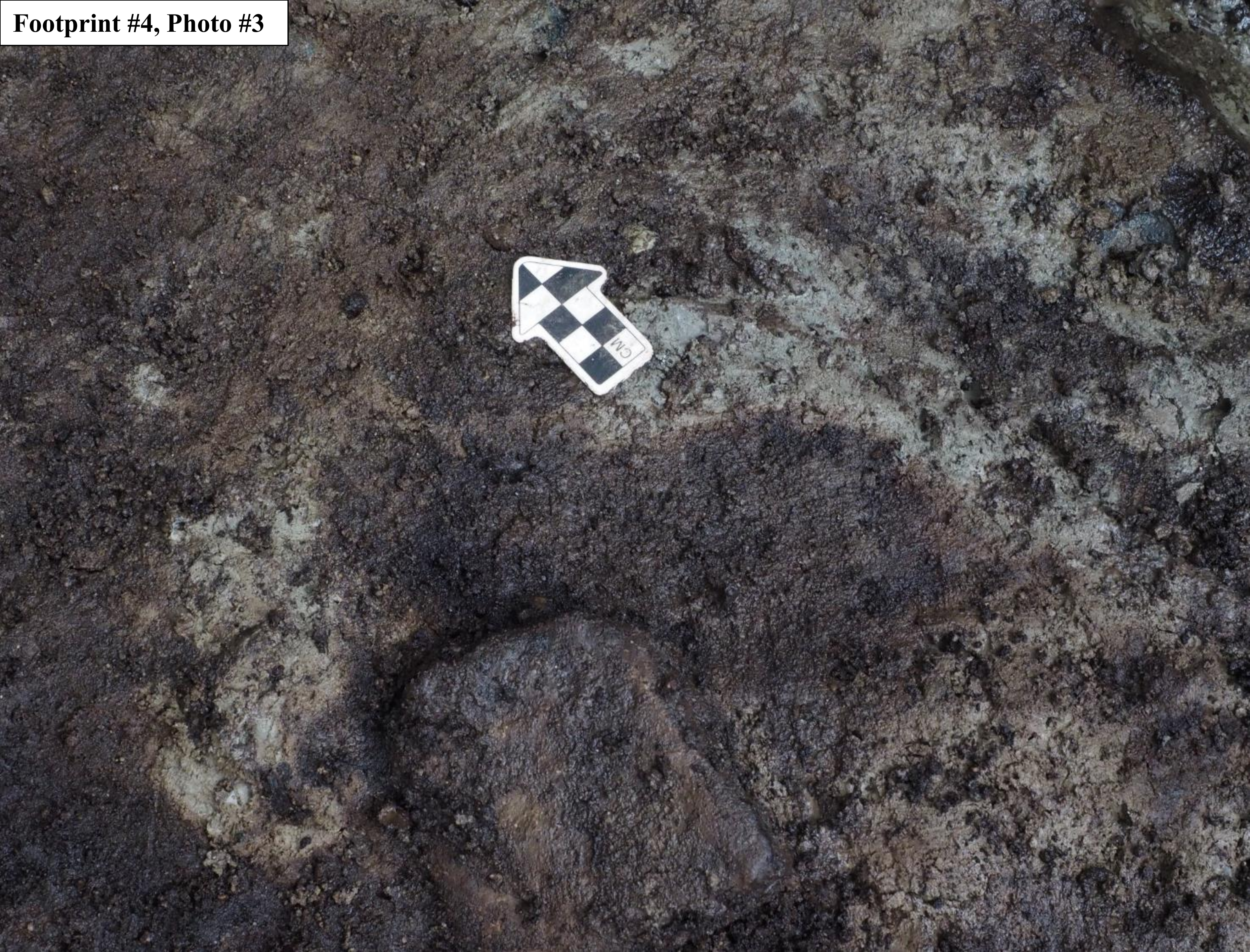

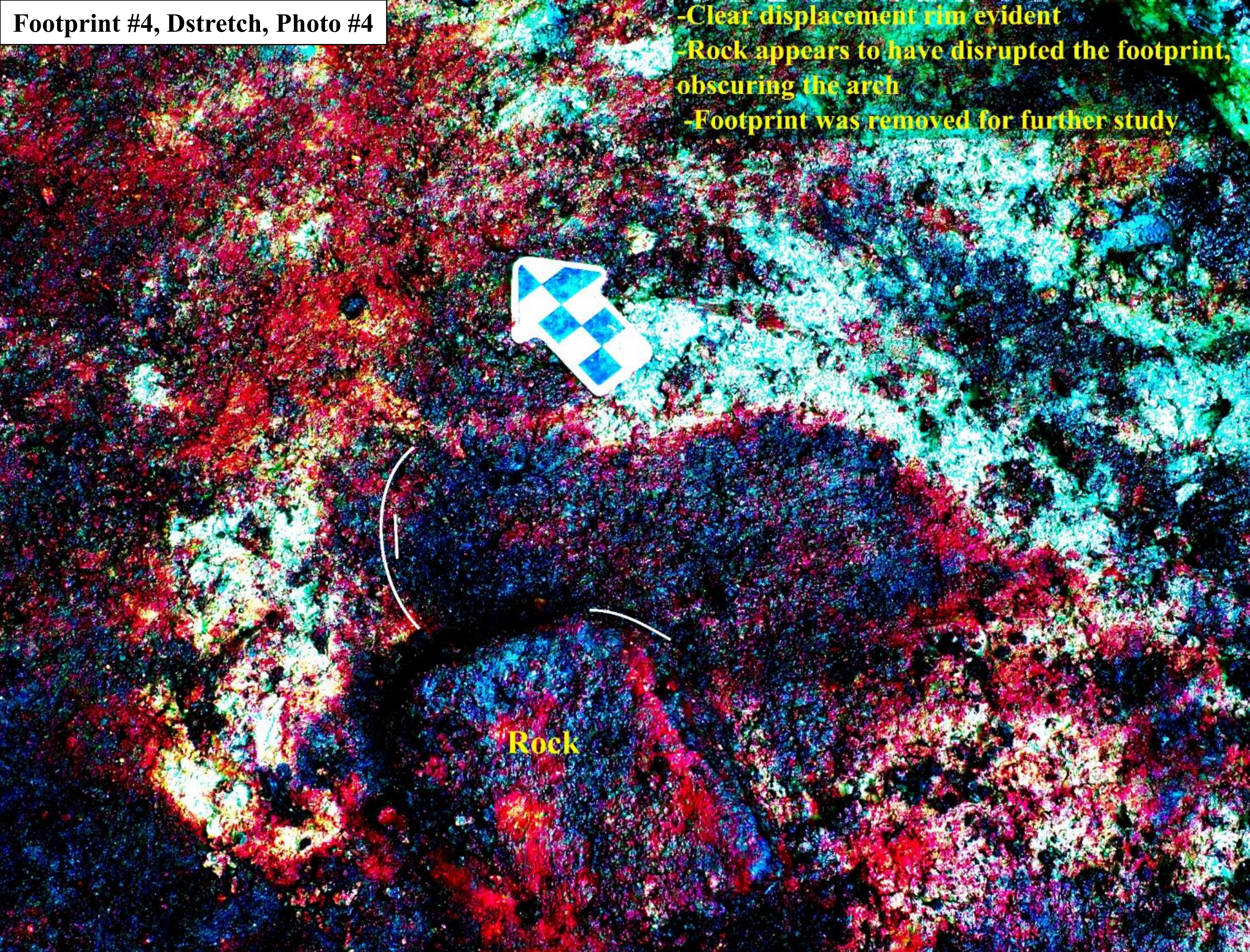
Footprint #4, Dstretch, Photo #4
-Clear displacement rim evident
-Rock appears to have disrupted the footprint, obscuring the arch
-Footprint was removed for further study
Rock

Footprint #4, Photo #5

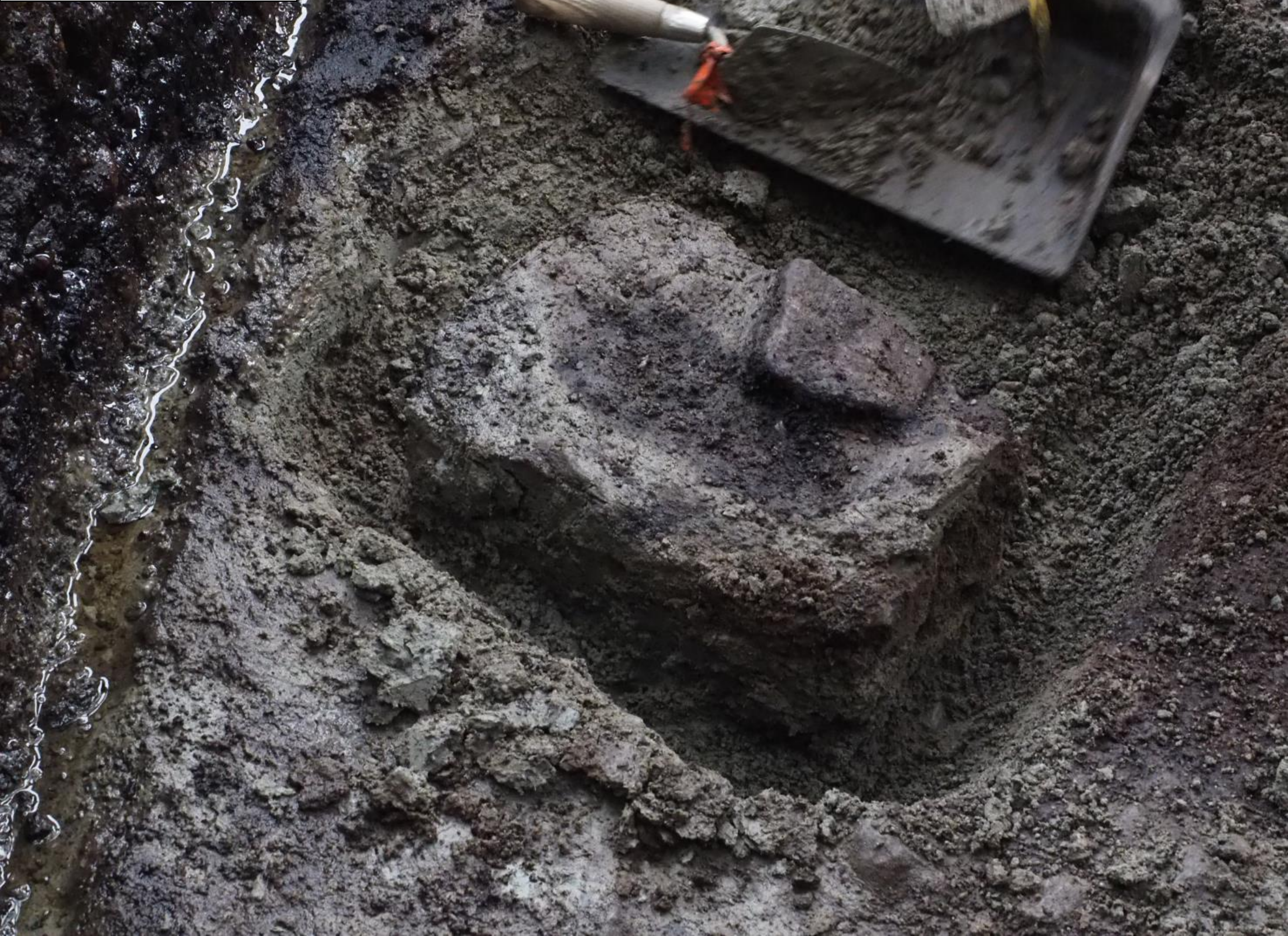

Footprint #4, Dstretch, Photo #6

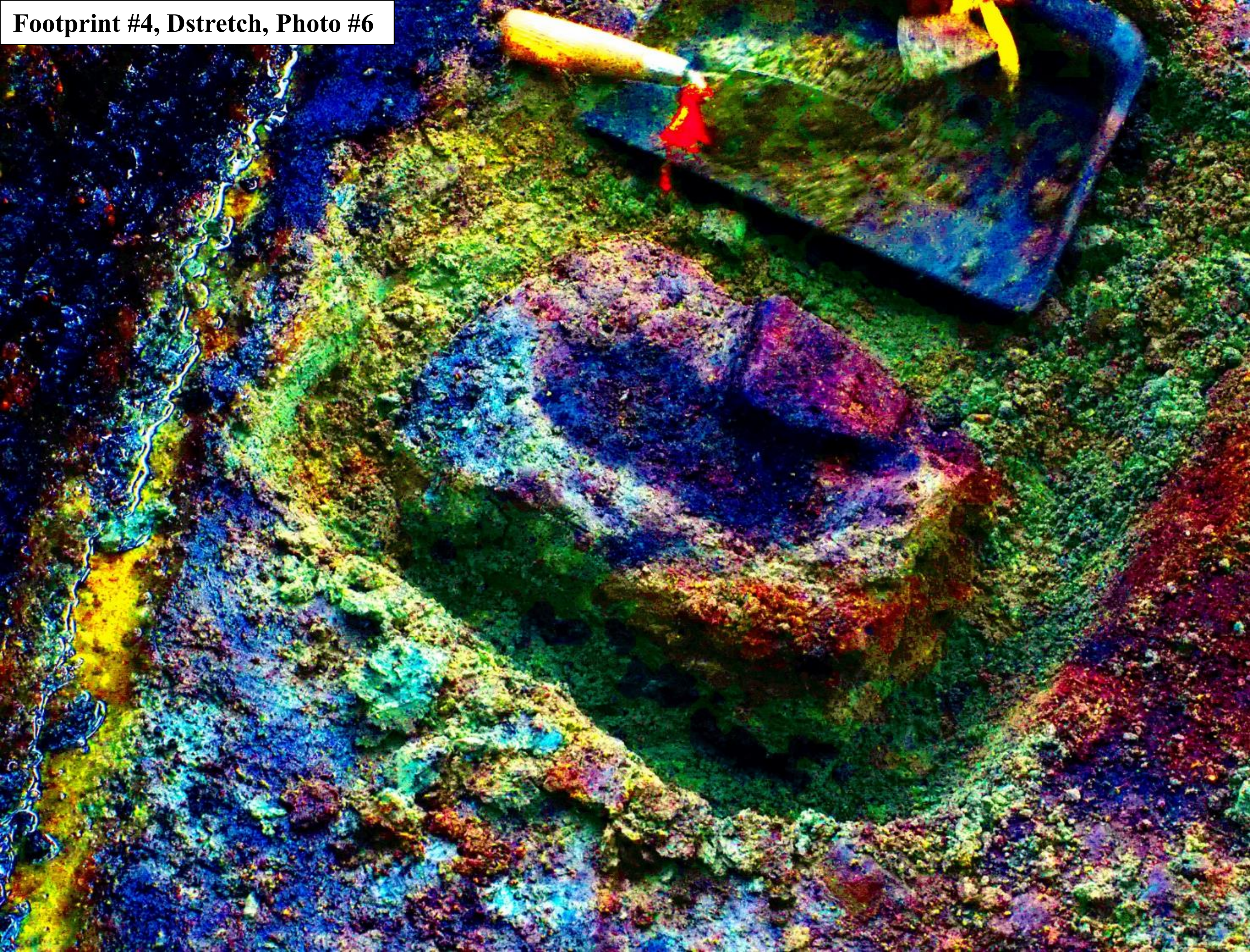

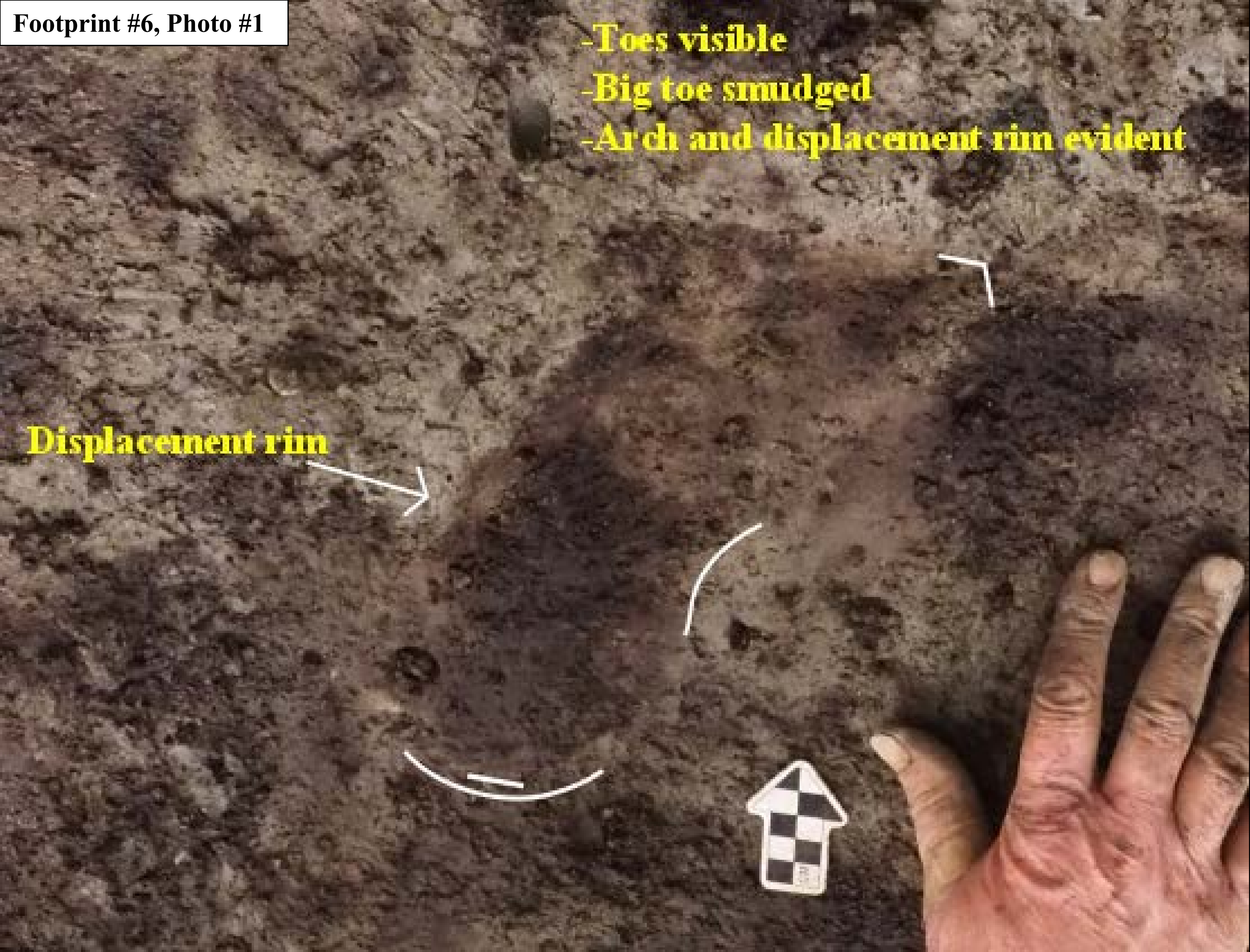
Footprint #6, Photo #1
-Toes visible
-Big toe smudged
-Arch and displacement rim evident
Displacement rim

Footprint #6, Photo #2

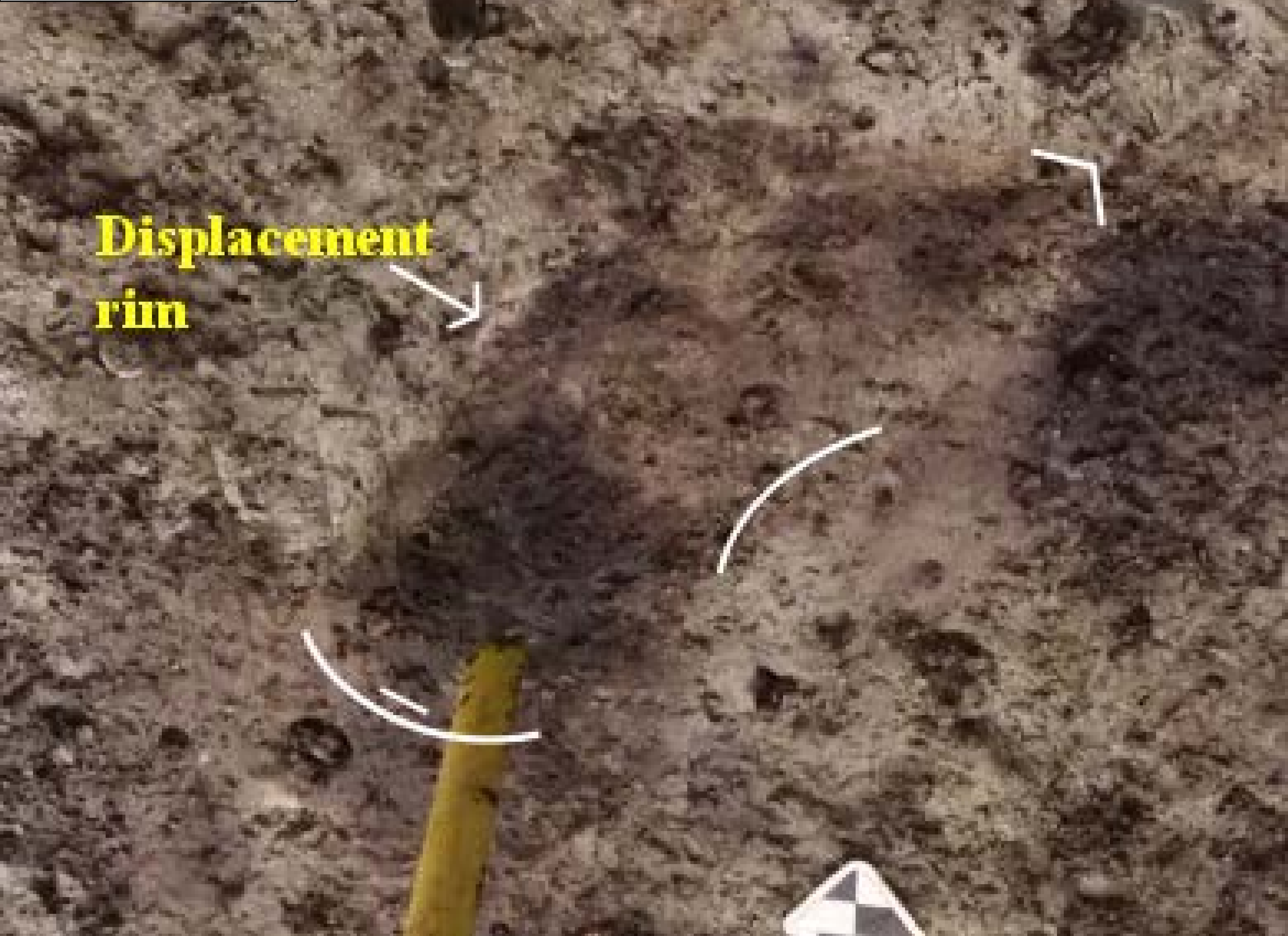

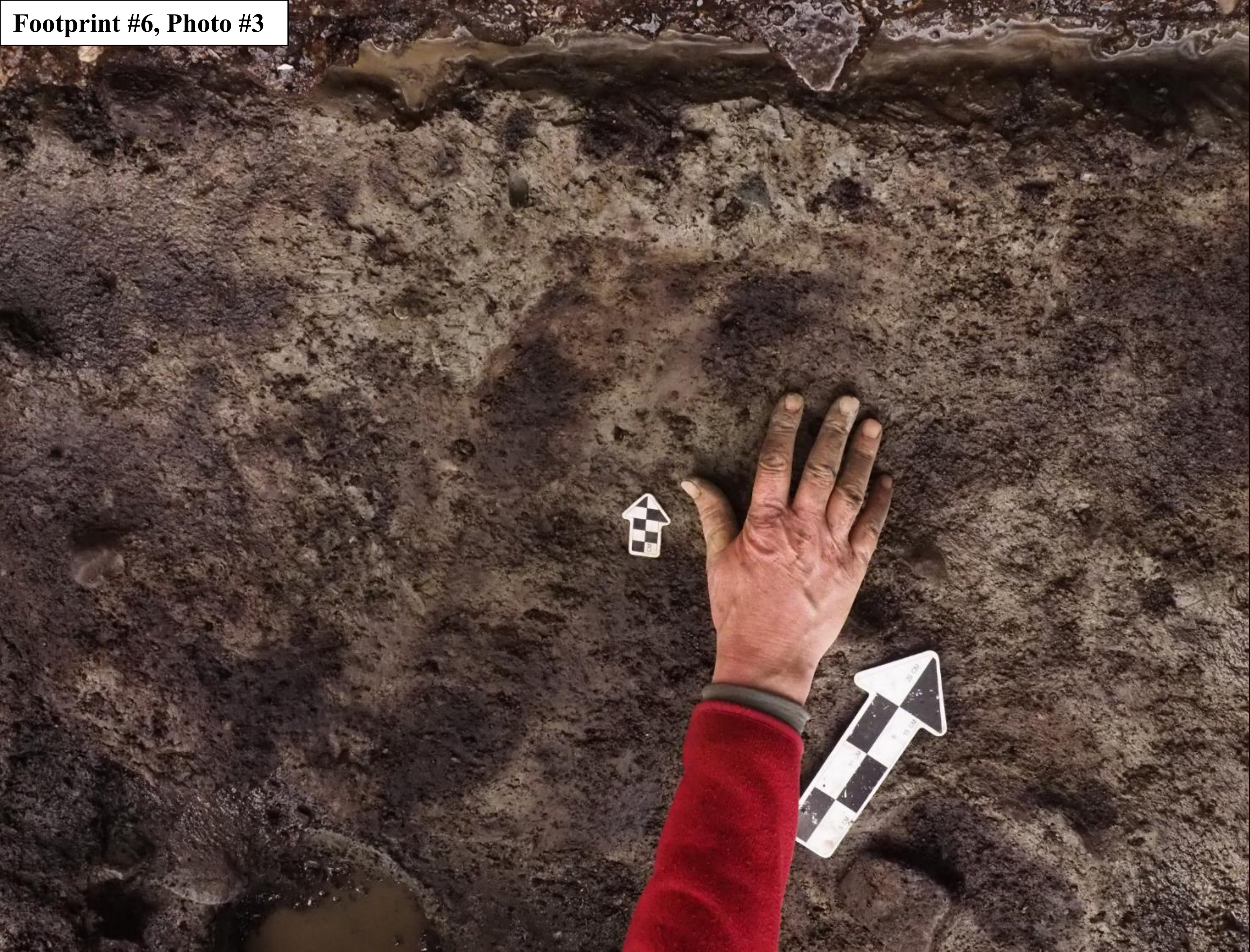

Footprint #6, Photo #3

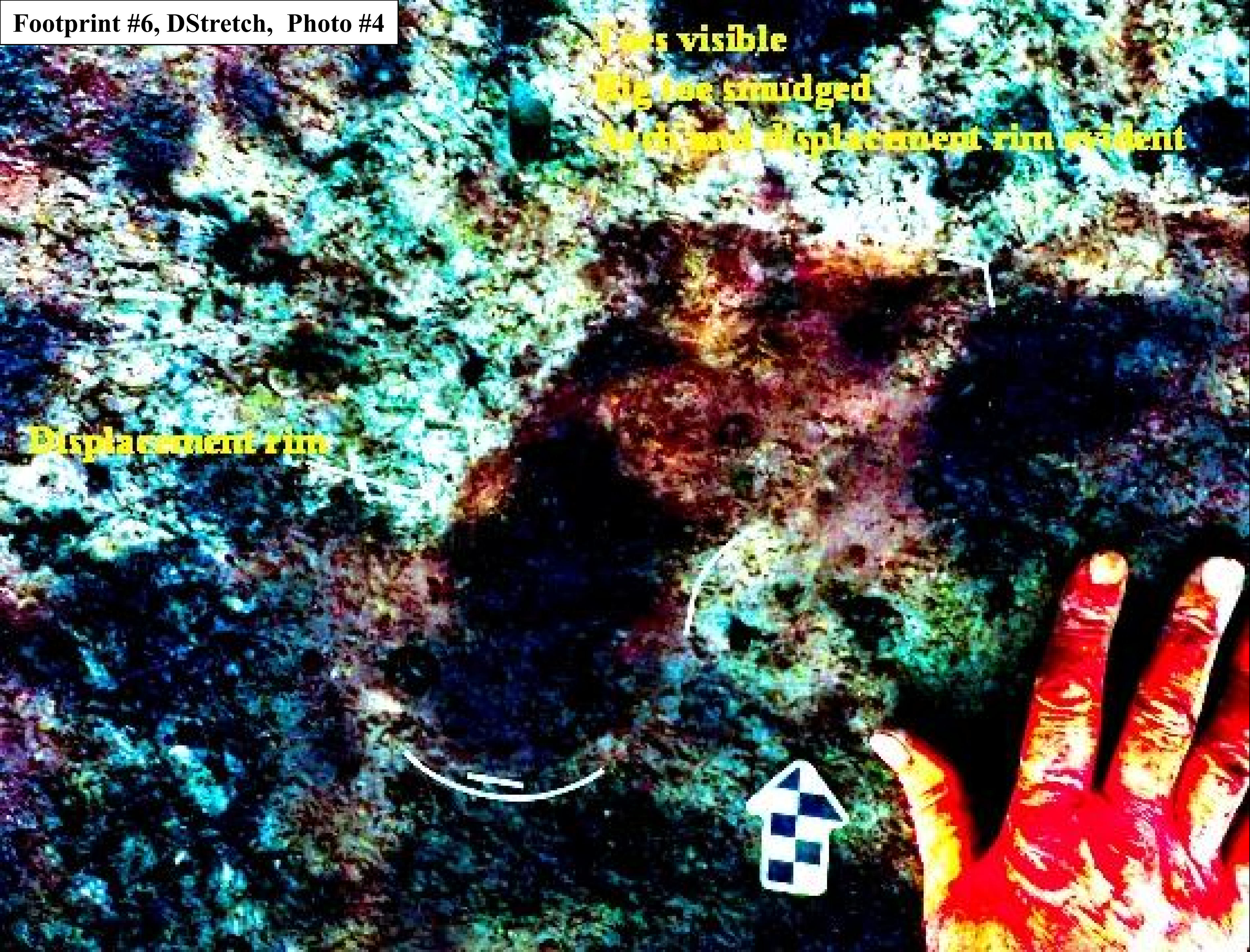

Footprint #6, DStretch, Photo #4

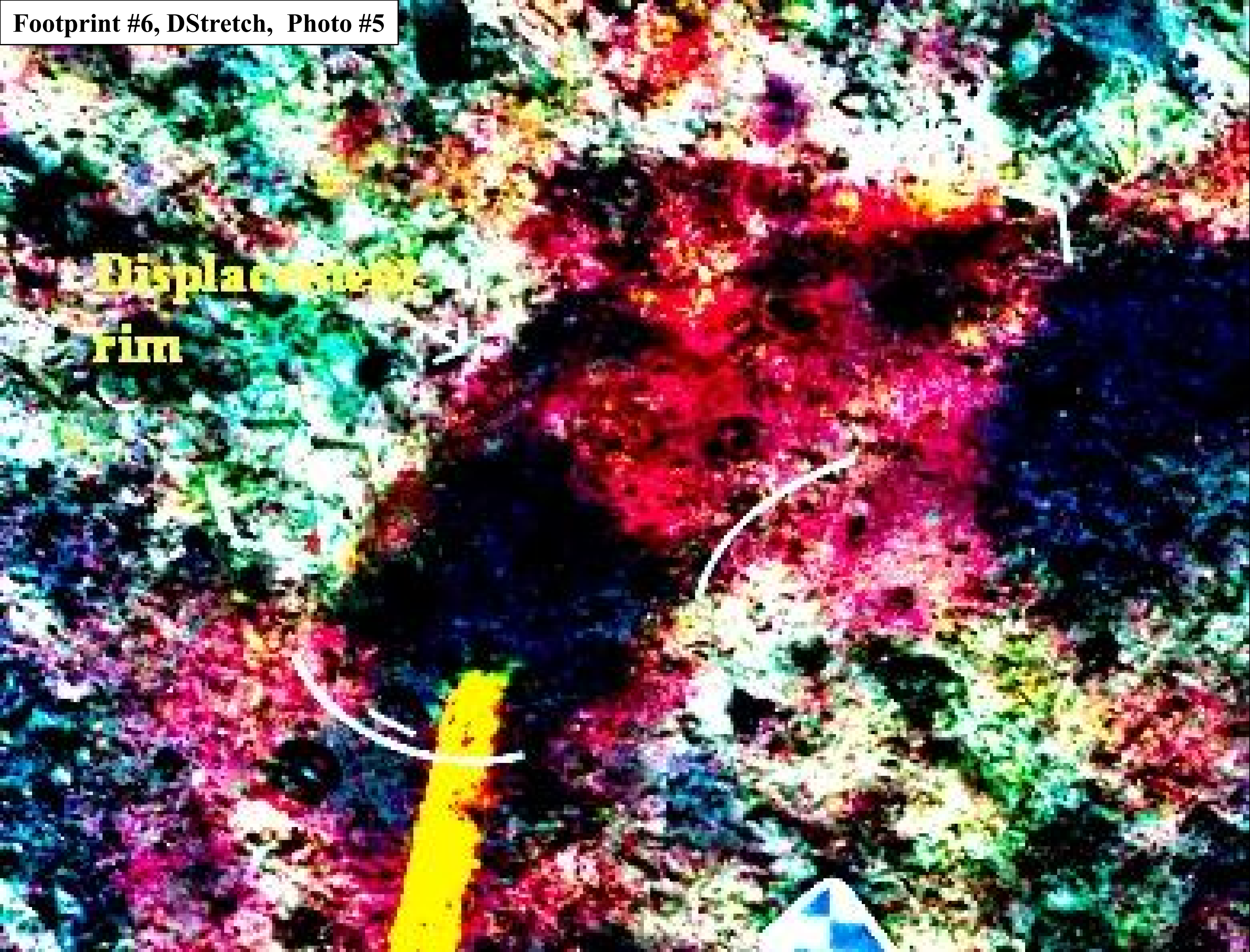

Footprint #6, DStretch, Photo #5

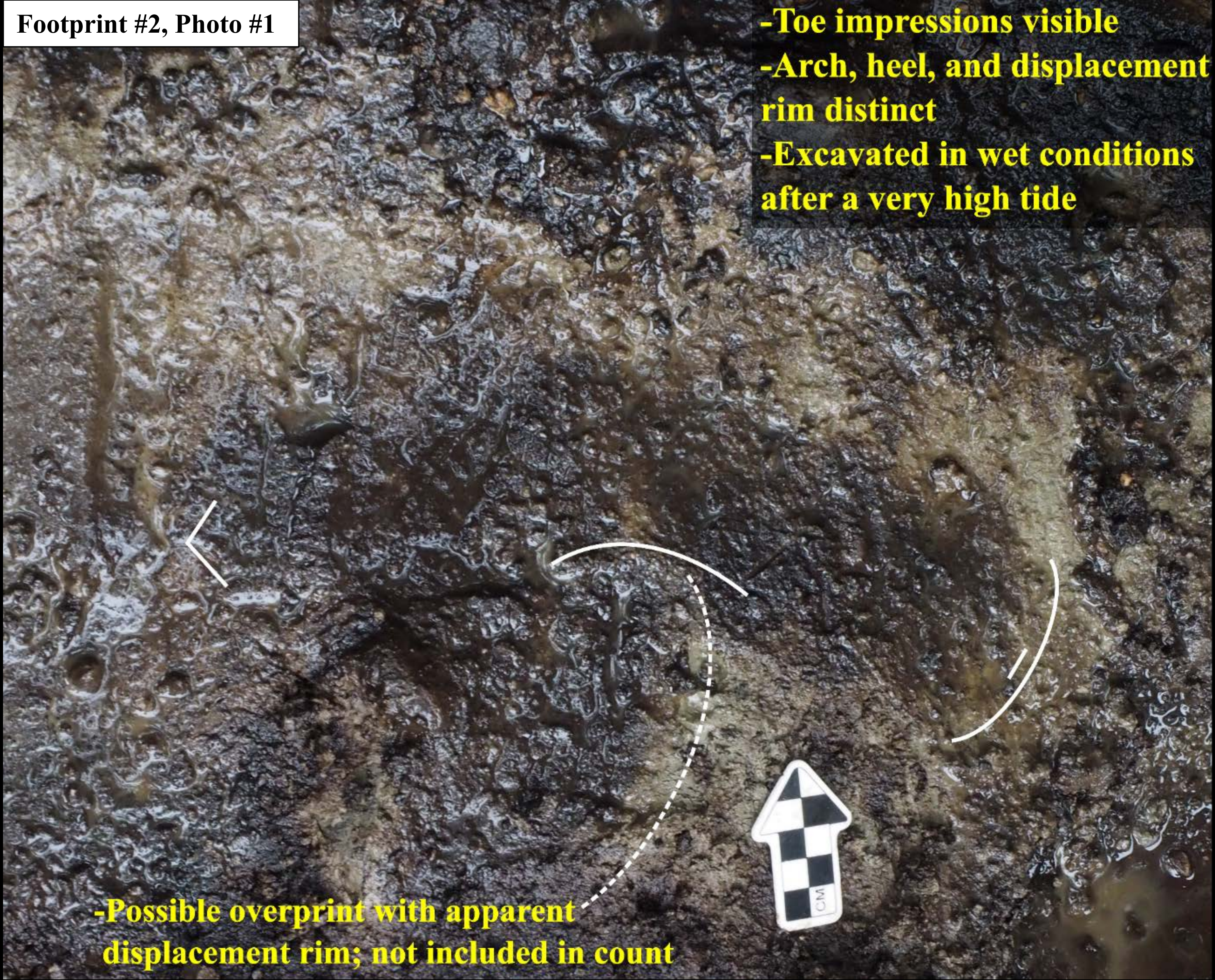
Footprint #2, Photo #1
-Toe impressions visible
-Arch, heel, and displacement rim distinct
-Excavated in wet conditions after a very high tide
-Possible overprint with apparent displacement rim; not included in count
CM

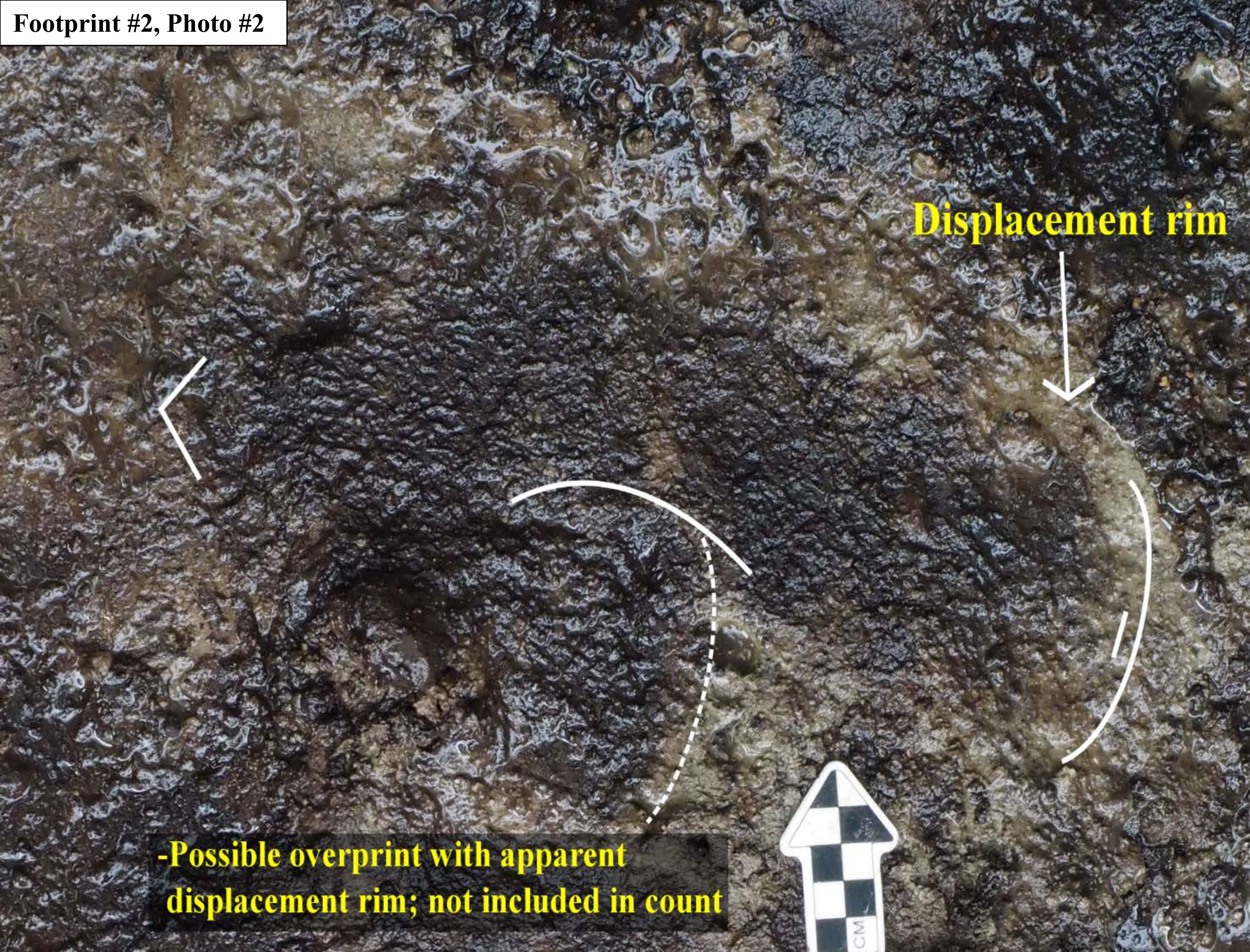
Footprint #2, Photo #2
Displacement rim
-Possible overprint with apparent
displacement rim; not included in count
CM

Footprint #2, Photo #3

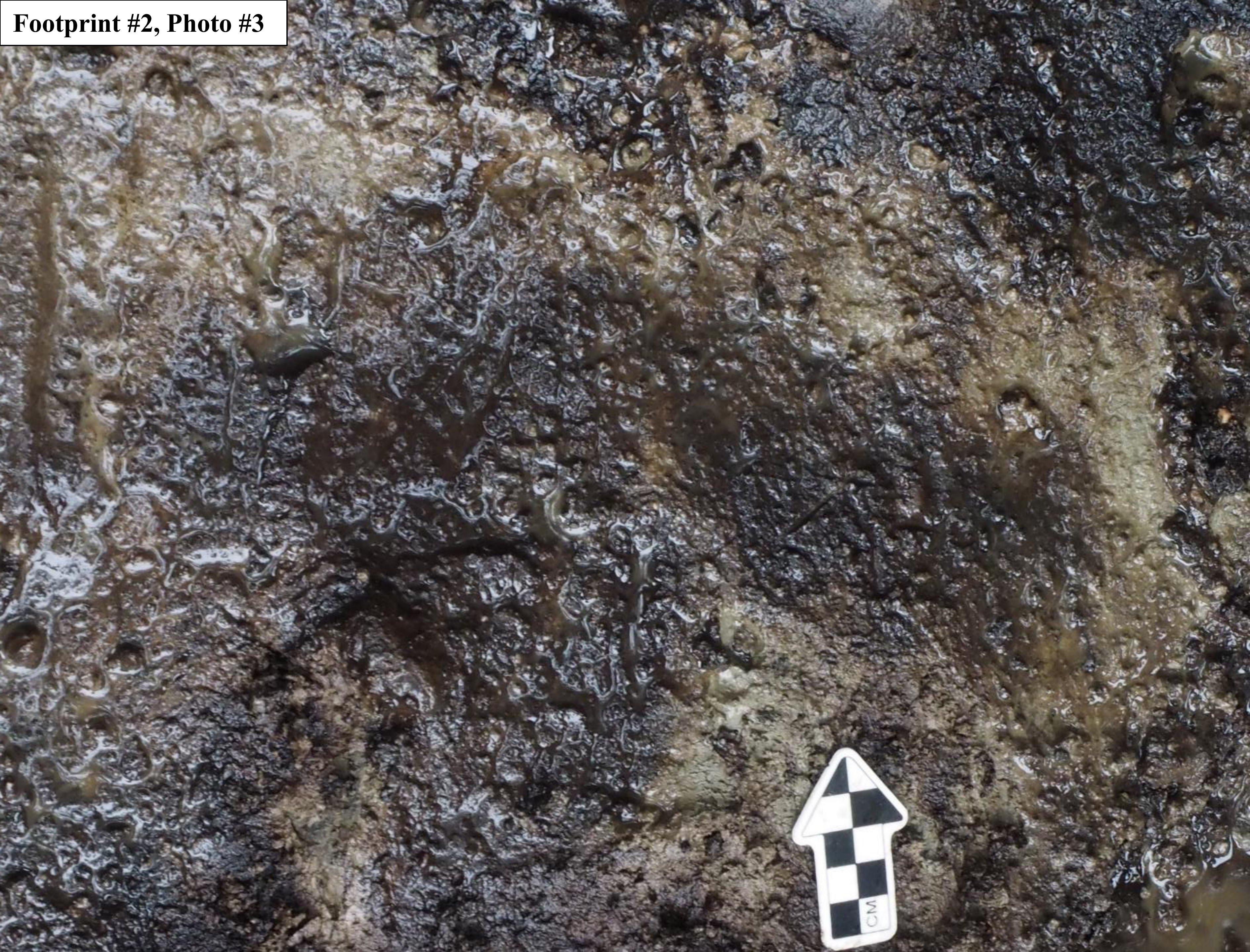

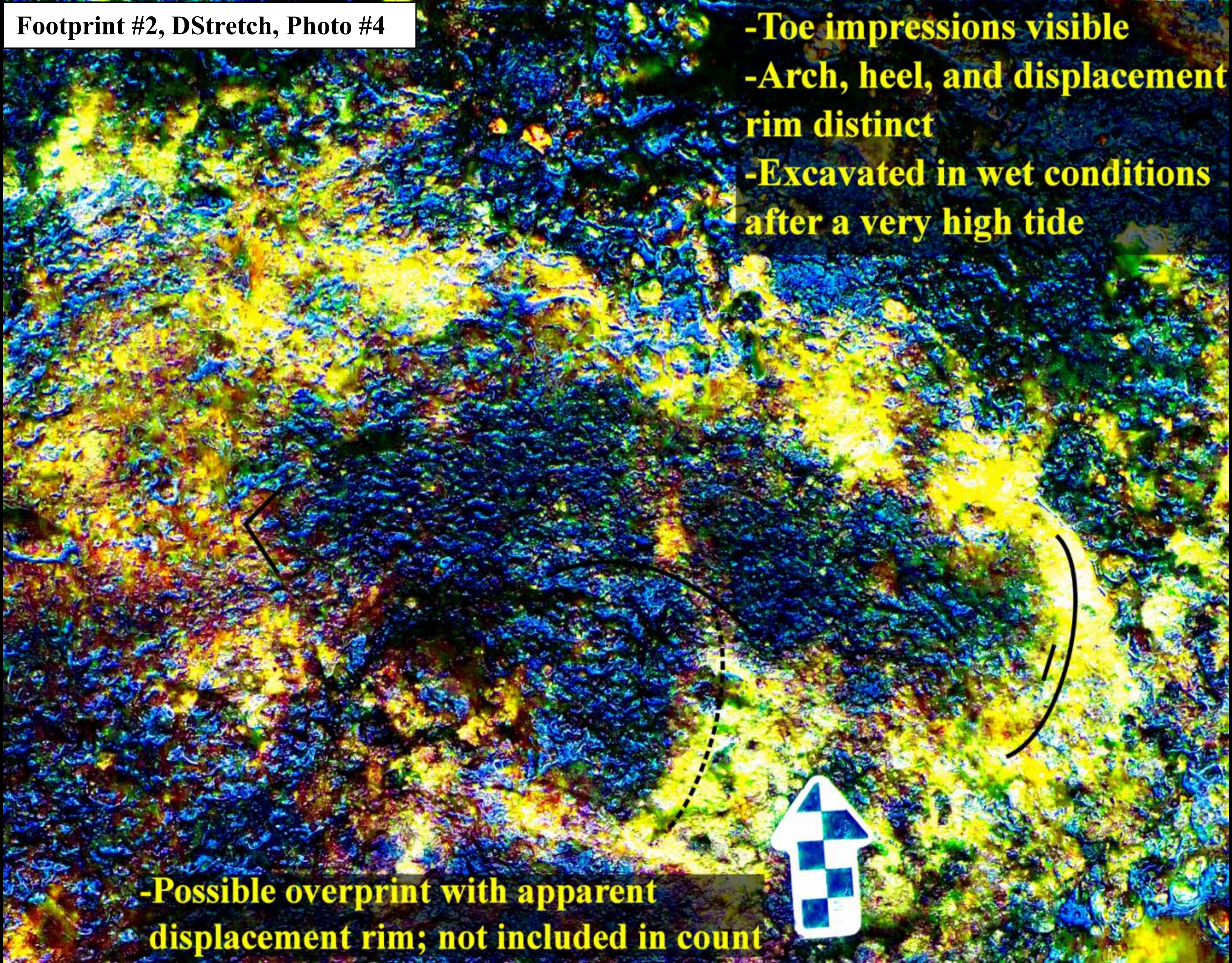
Footprint #2, DStretch, Photo #4
-Toe impressions visible
-Arch, heel, and displacement rim distinct
-Excavated in wet conditions after a very high tide
-Possible overprint with apparent displacement rim; not included in count

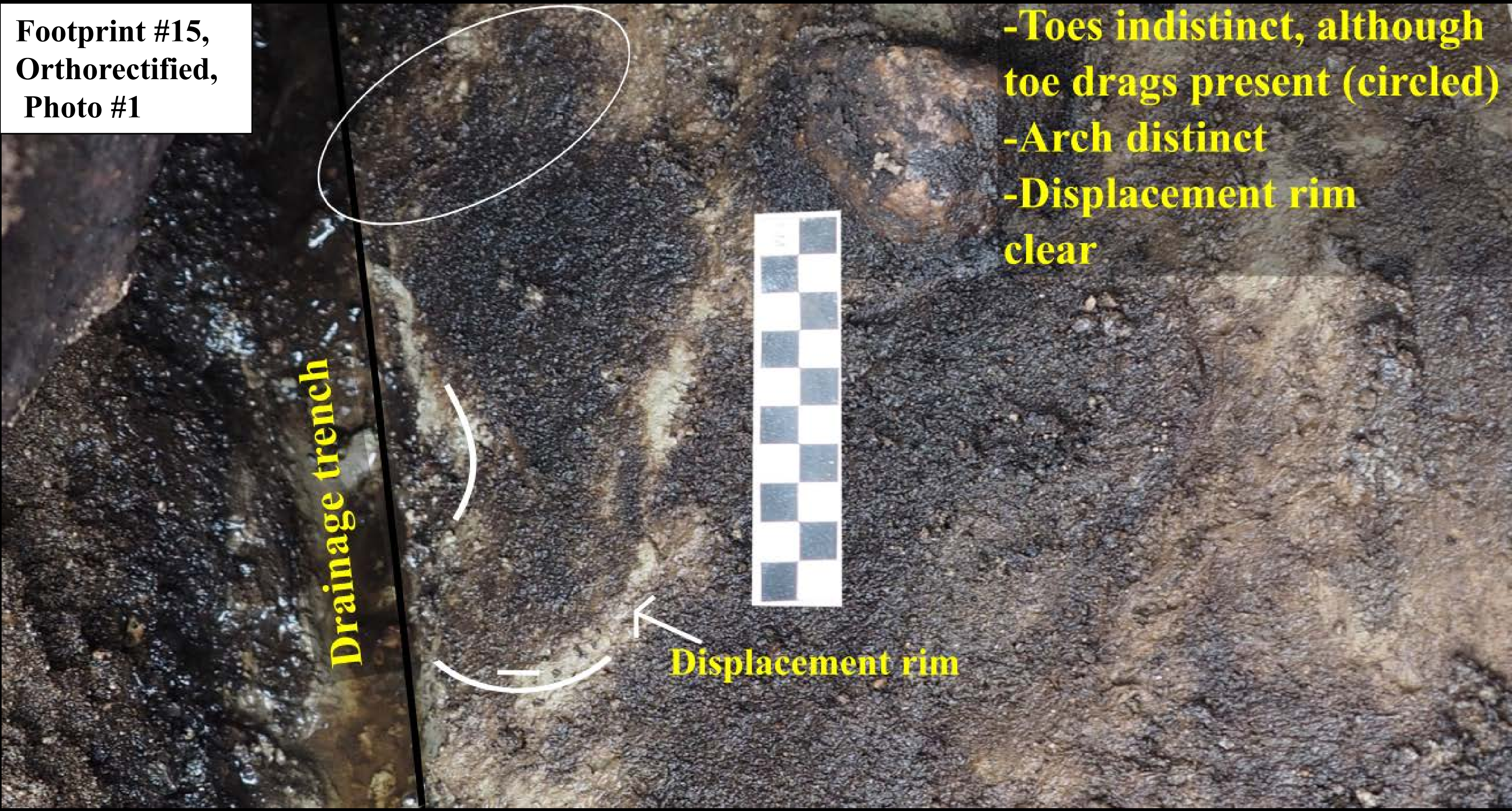
Footprint #15,
Orthorectified,
Photo #1
-Toes indistinct, although toe drags present (circled)
-Arch distinct
-Displacement rim clear
Drainage trench
Displacement rim

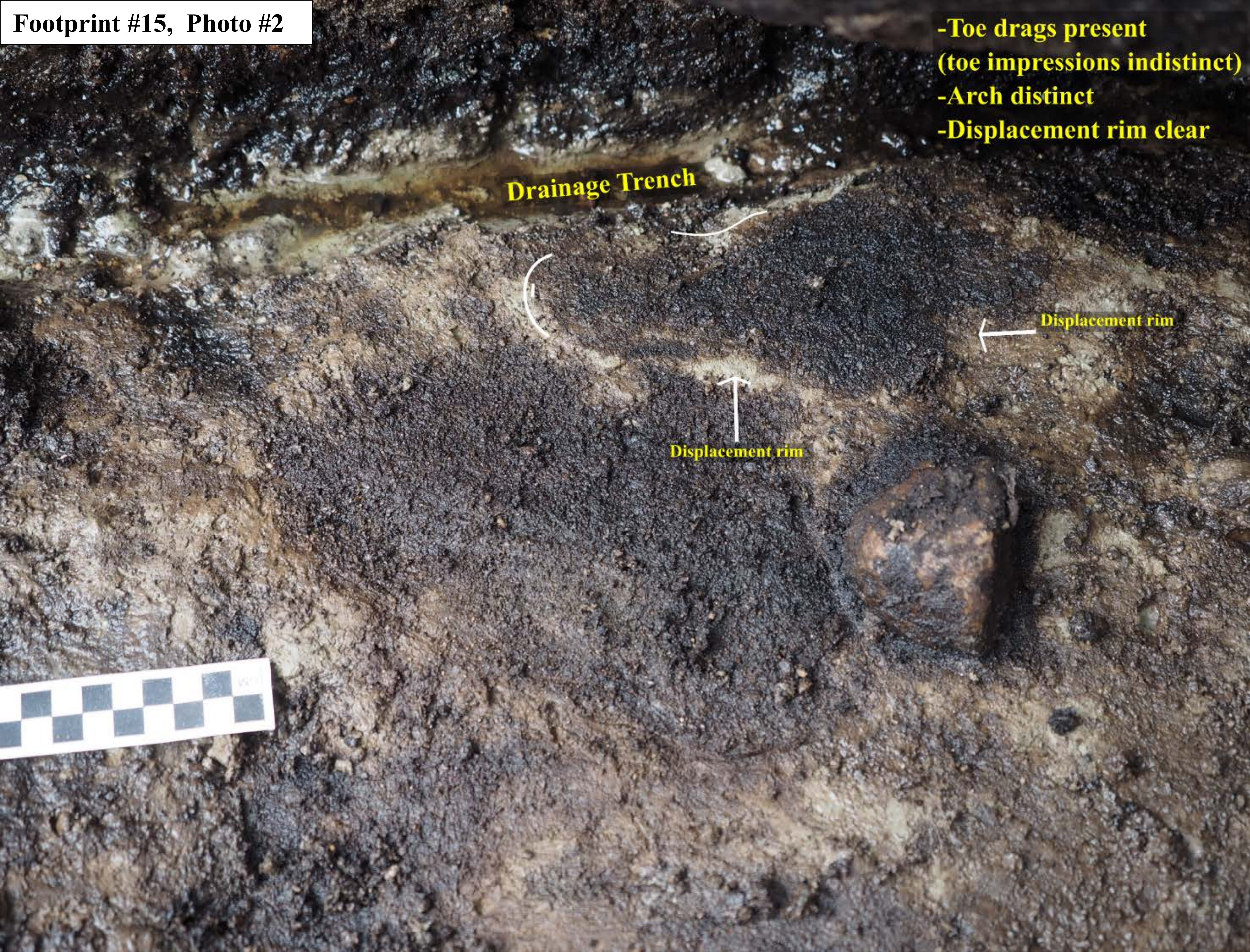
Footprint #15, Photo #2
-Toe drags present
(toe impressions indistinct)
-Arch distinct
-Displacement rim clear
Drainage Trench
Displacement rim
Displacement rim

Footprint #15, Tracing F.P. Outline, Photo #3

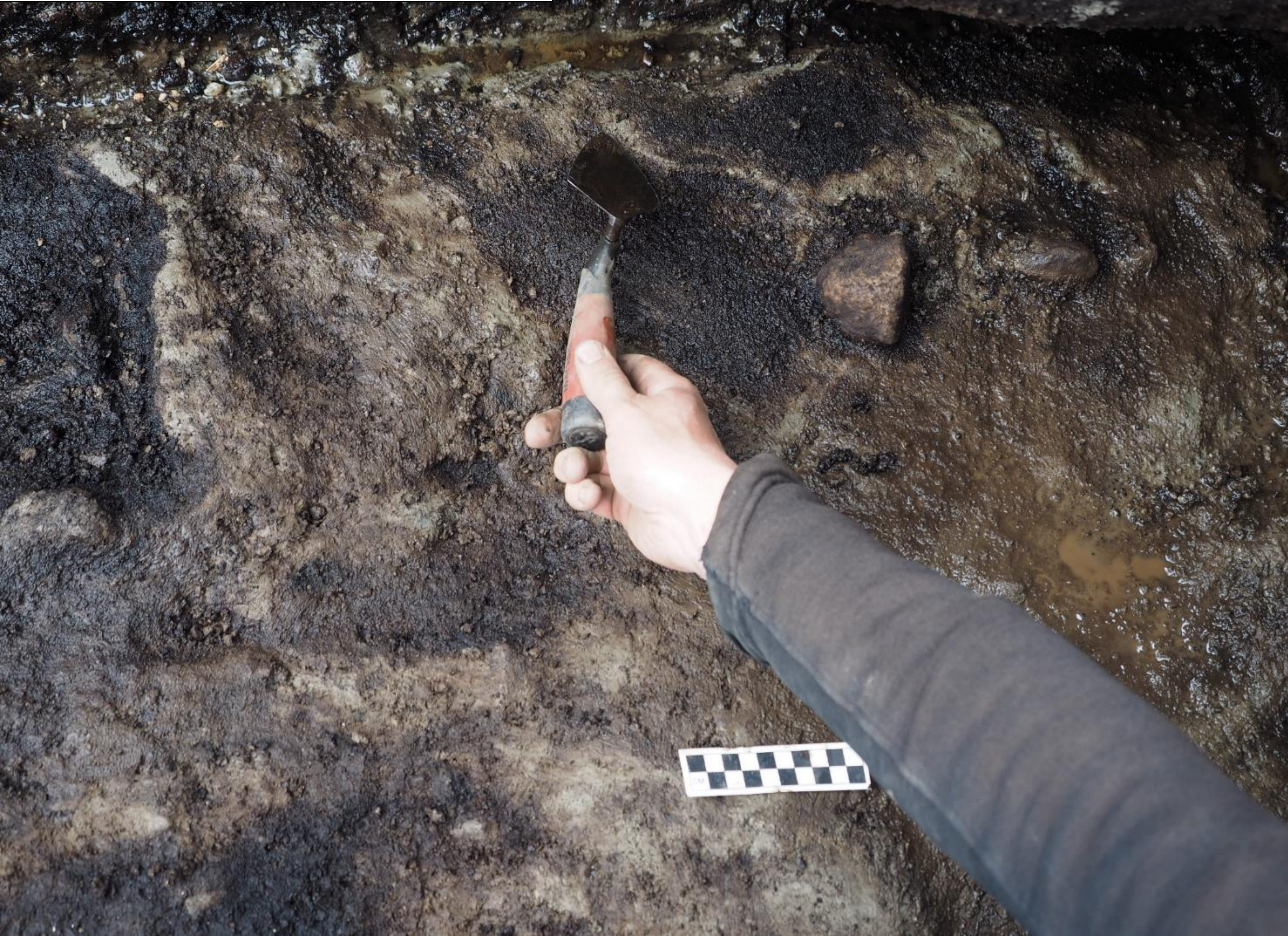

Footprint #15, Tracing F.P. Outline, Photo #4

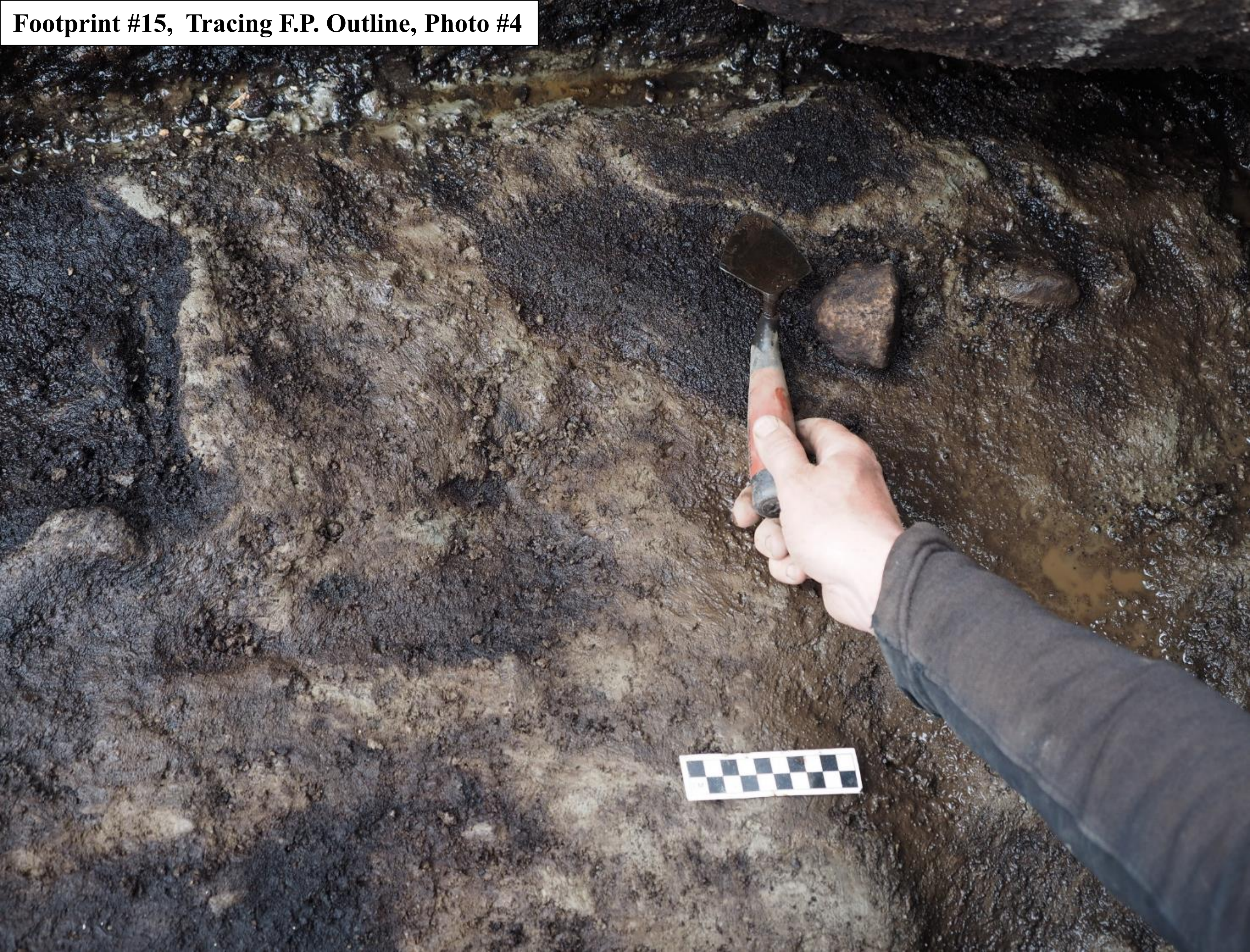

Footprint #15, Tracing F.P. Outline, Photo #5

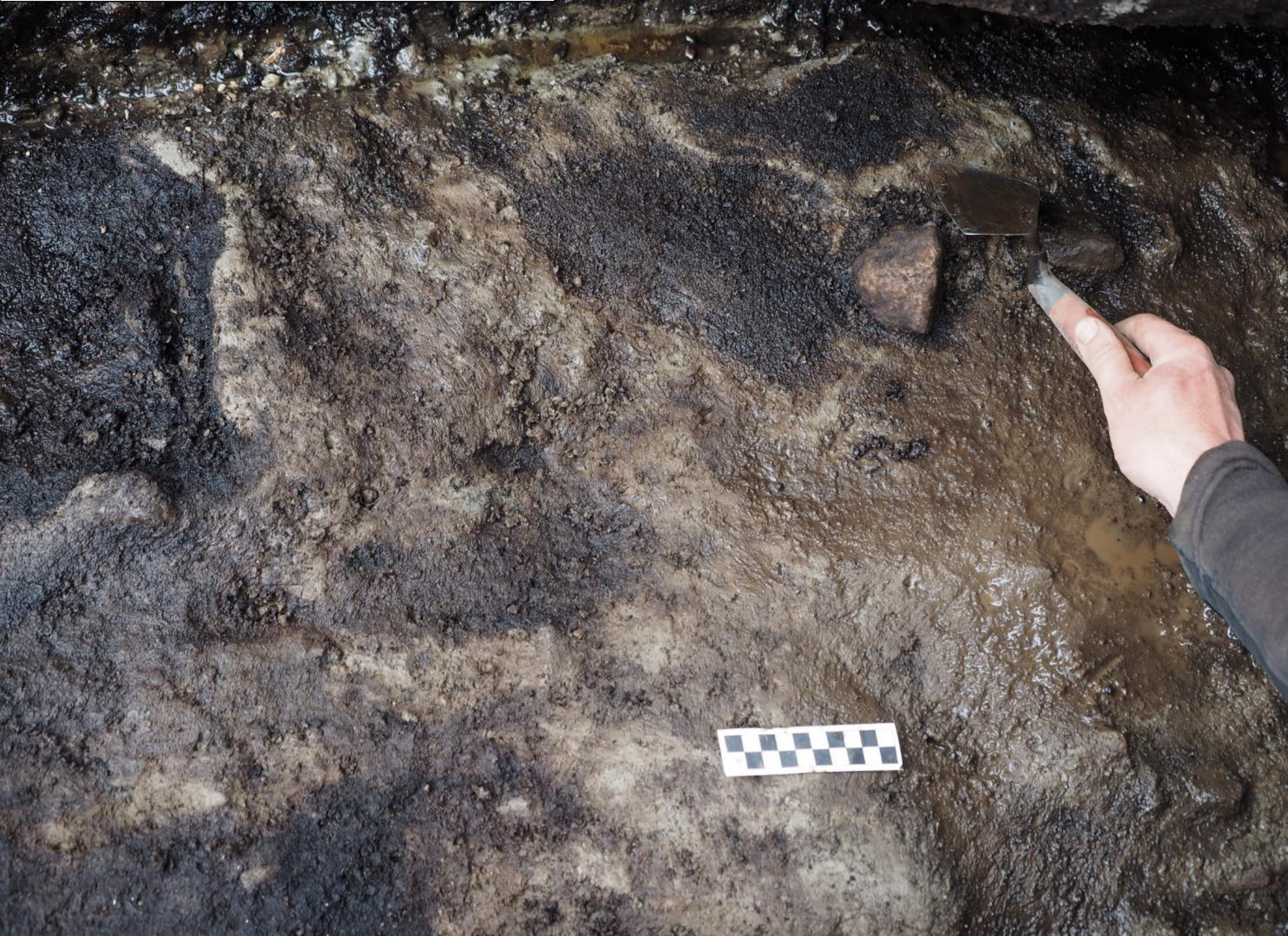

Footprint #15, Tracing F.P. Outline, Photo #6

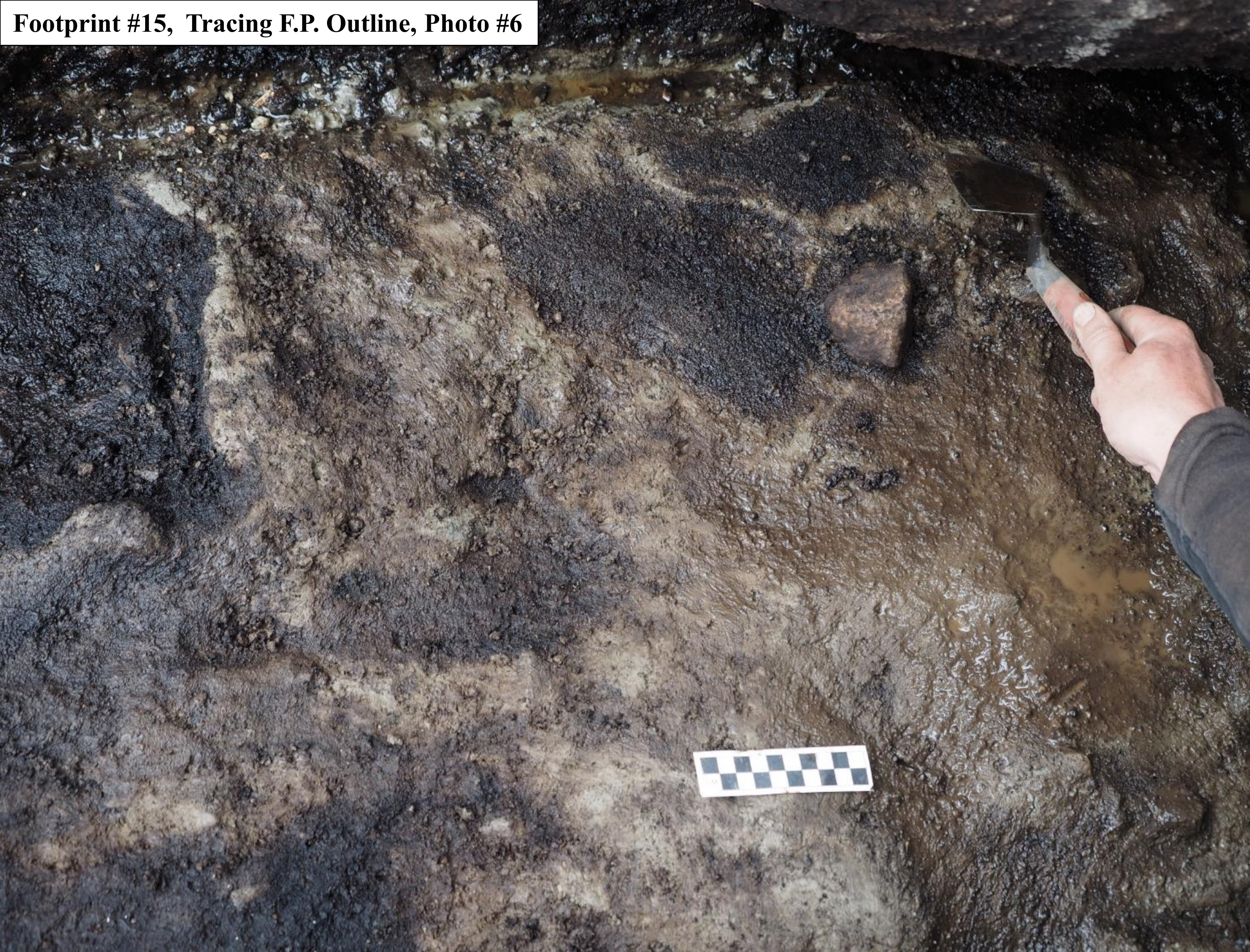

Footprint #15, Tracing F.P. Outline, Photo #7

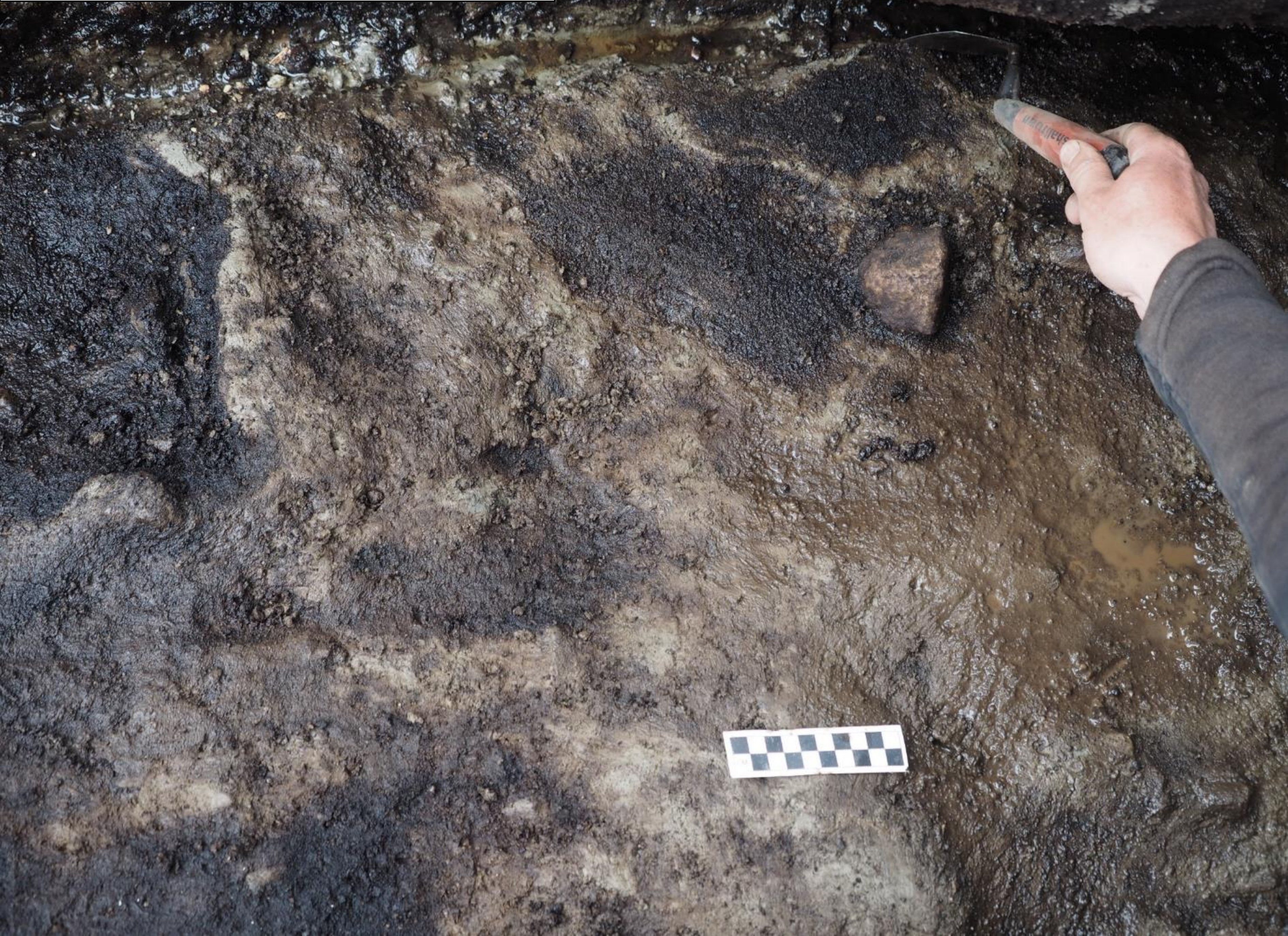

Footprint #15, Tracing F.P. Outline, Photo #8

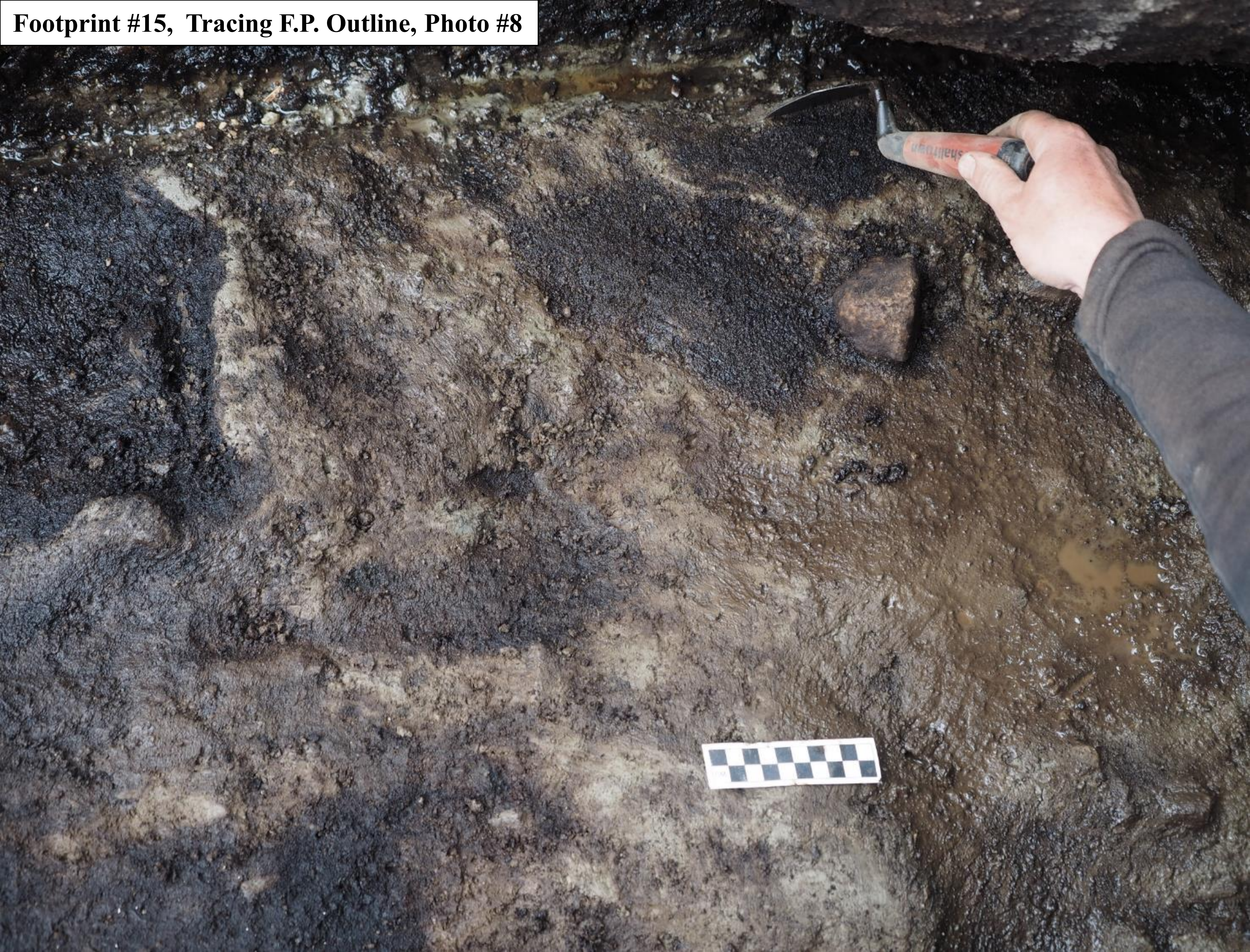

Footprint #17, Photo #1

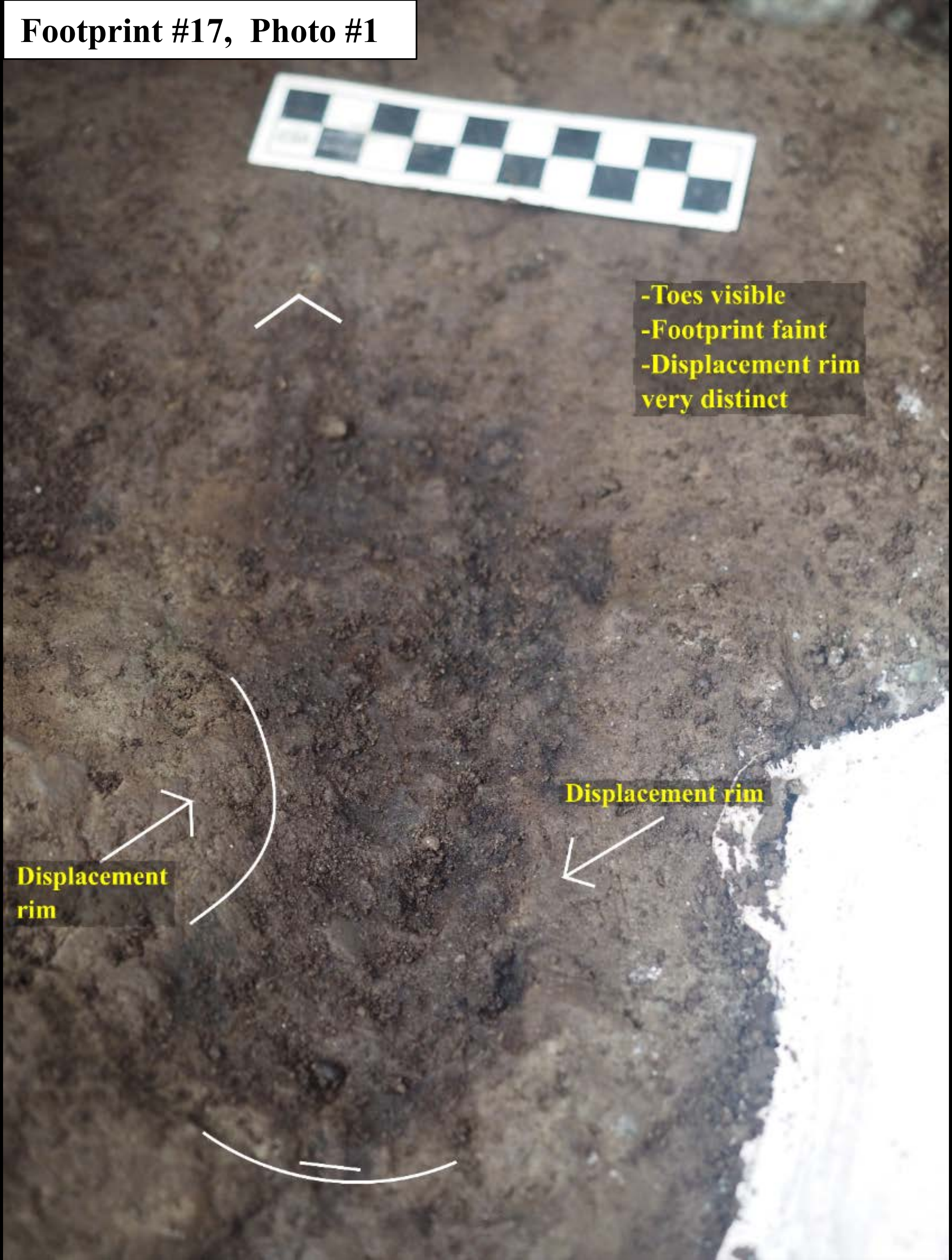

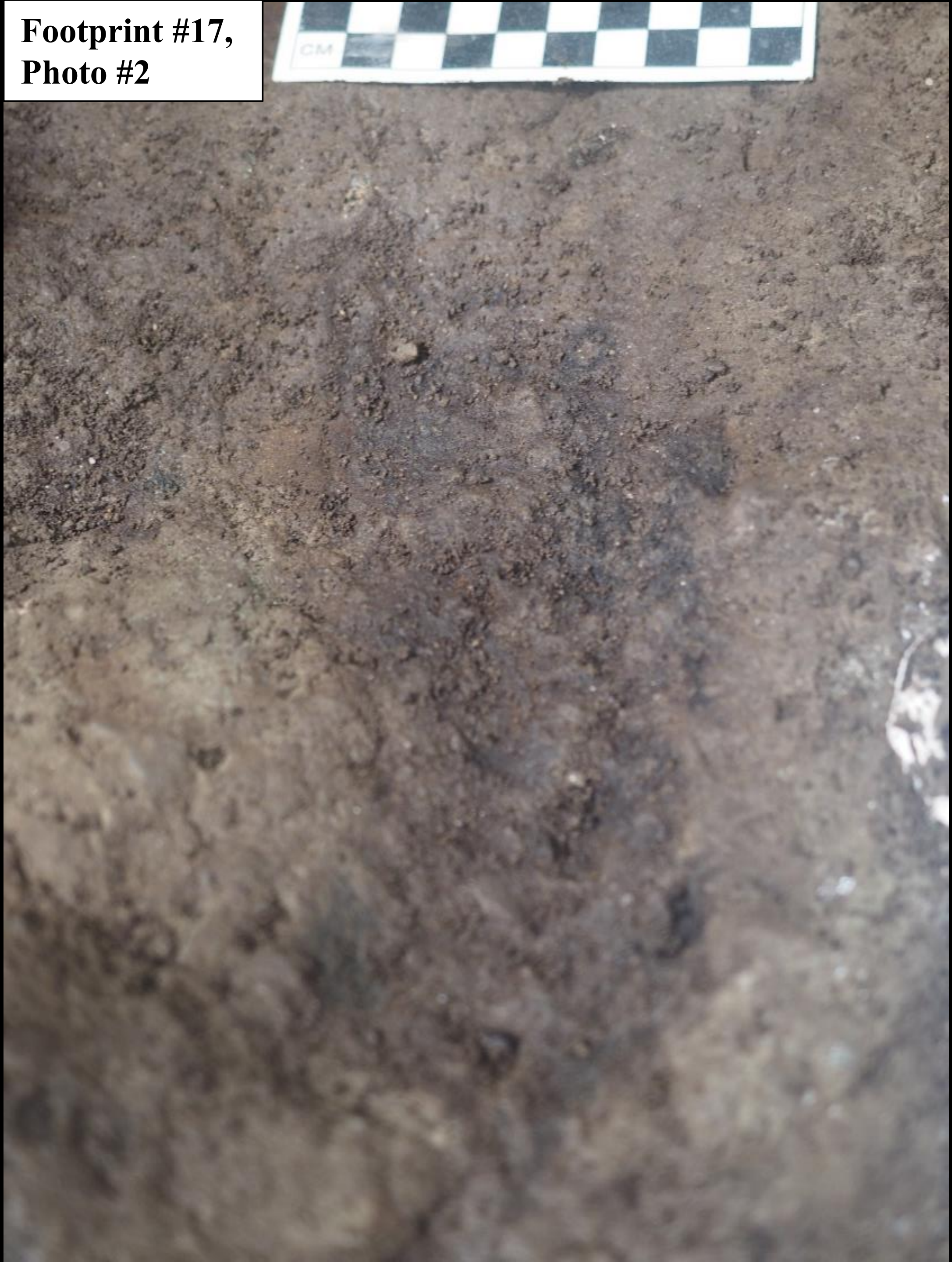

Footprint #17, Photo #2

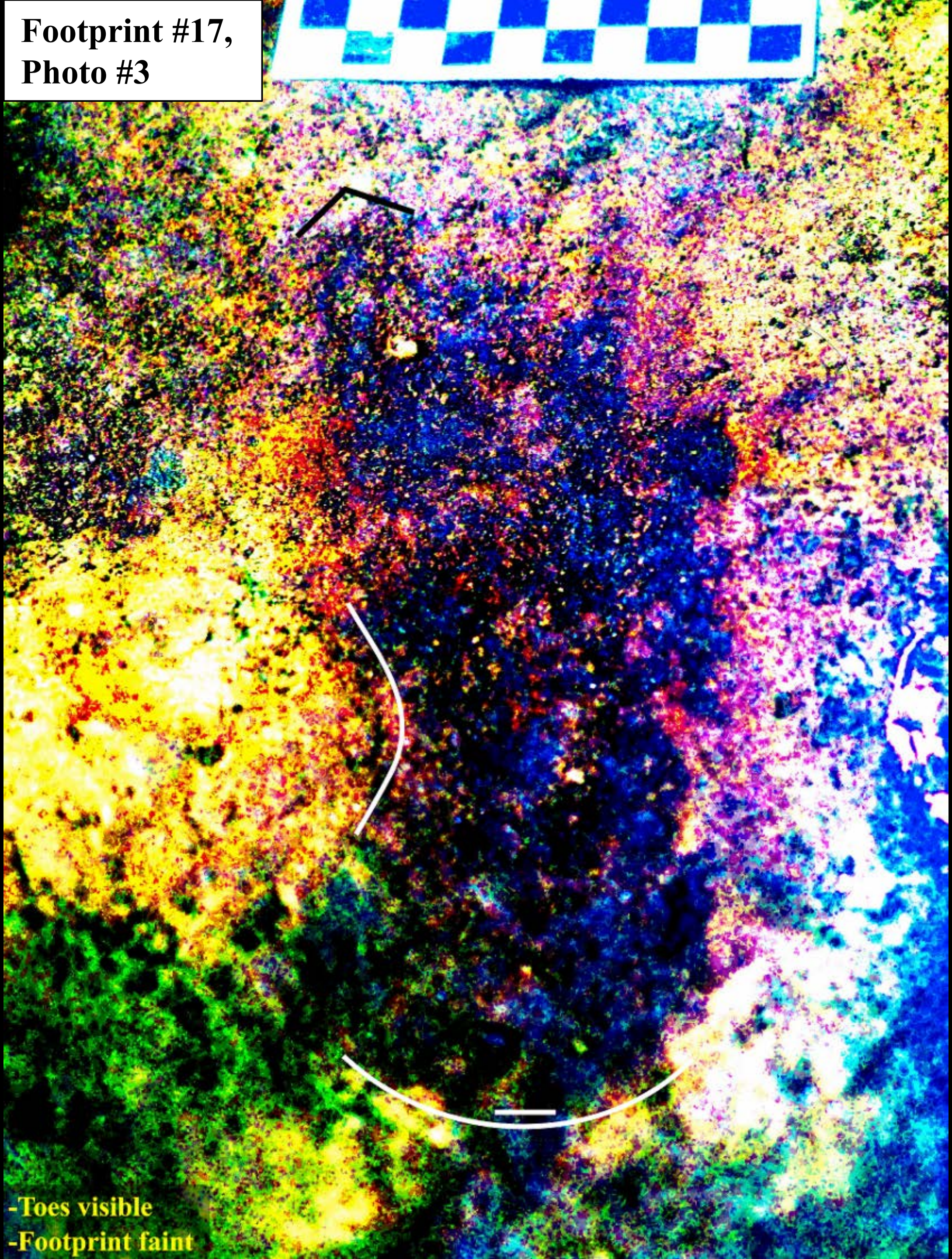
Footprint #17,
Photo #3
-Toes visible
-Footprint faint

Footprint #17, Tracing F.P. Outline, Photo #4

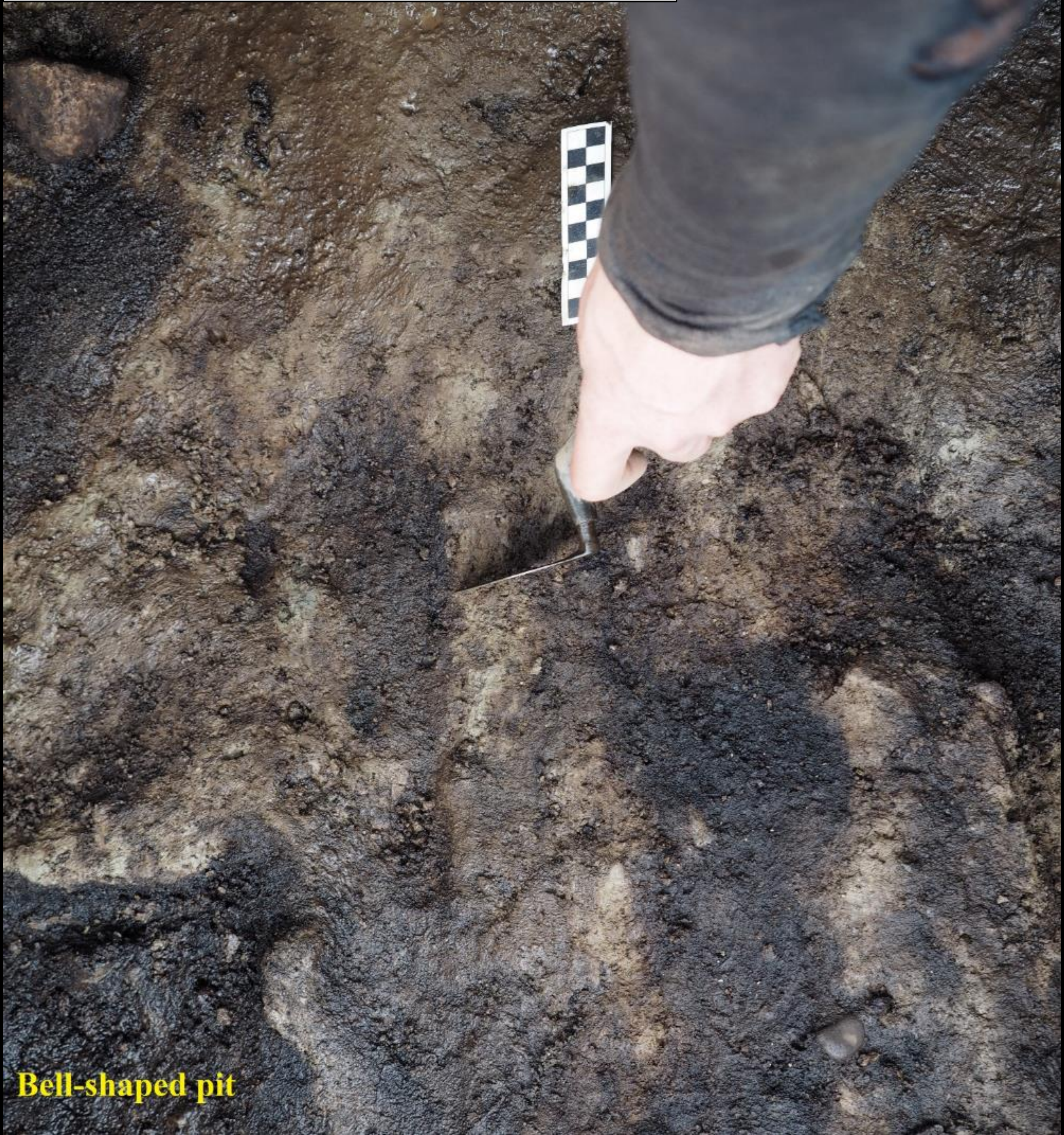

Footprint #17, Tracing F.P. Outline, Photo #5

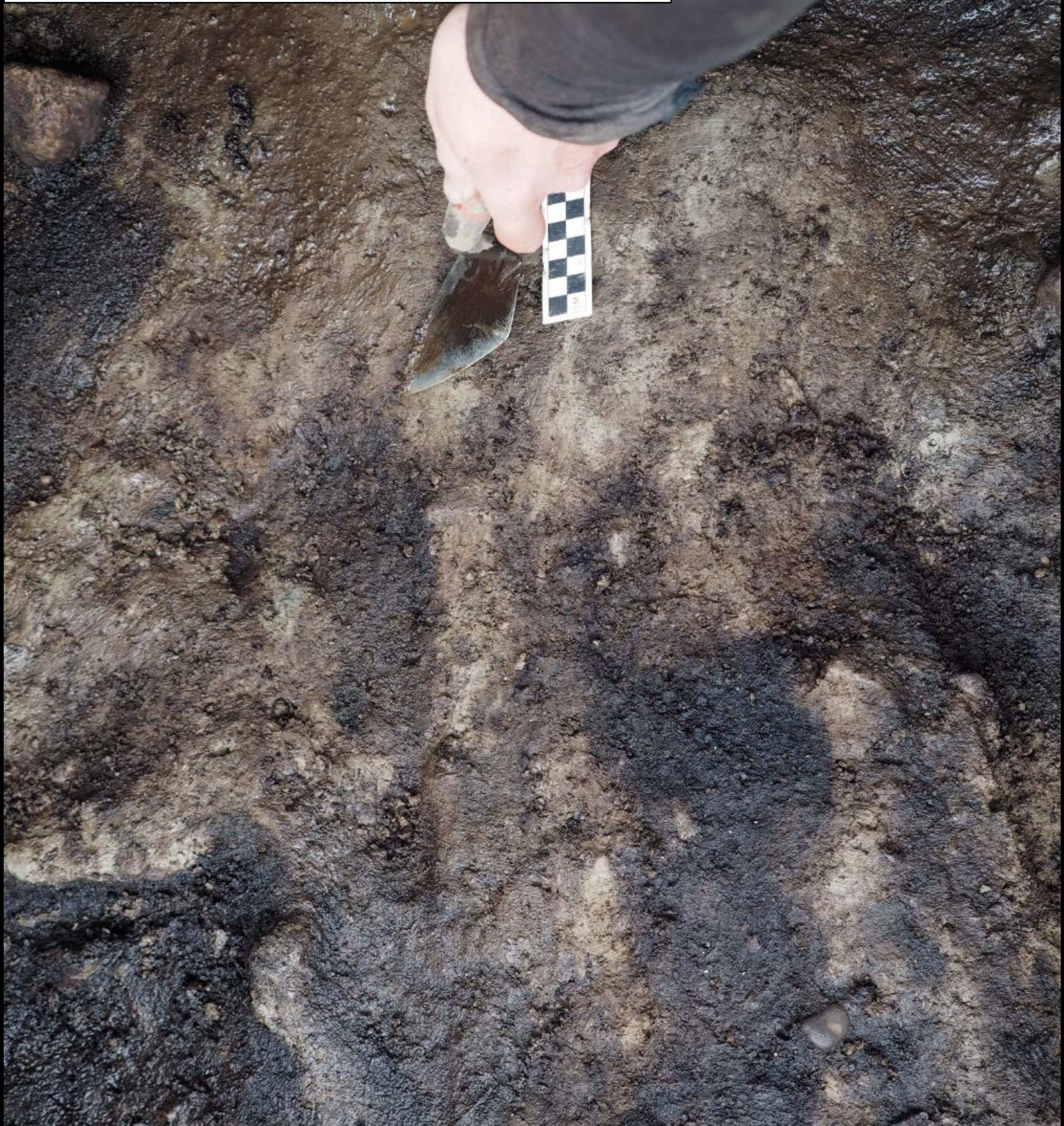

Footprint #17, Tracing F.P. Outline, Photo #6

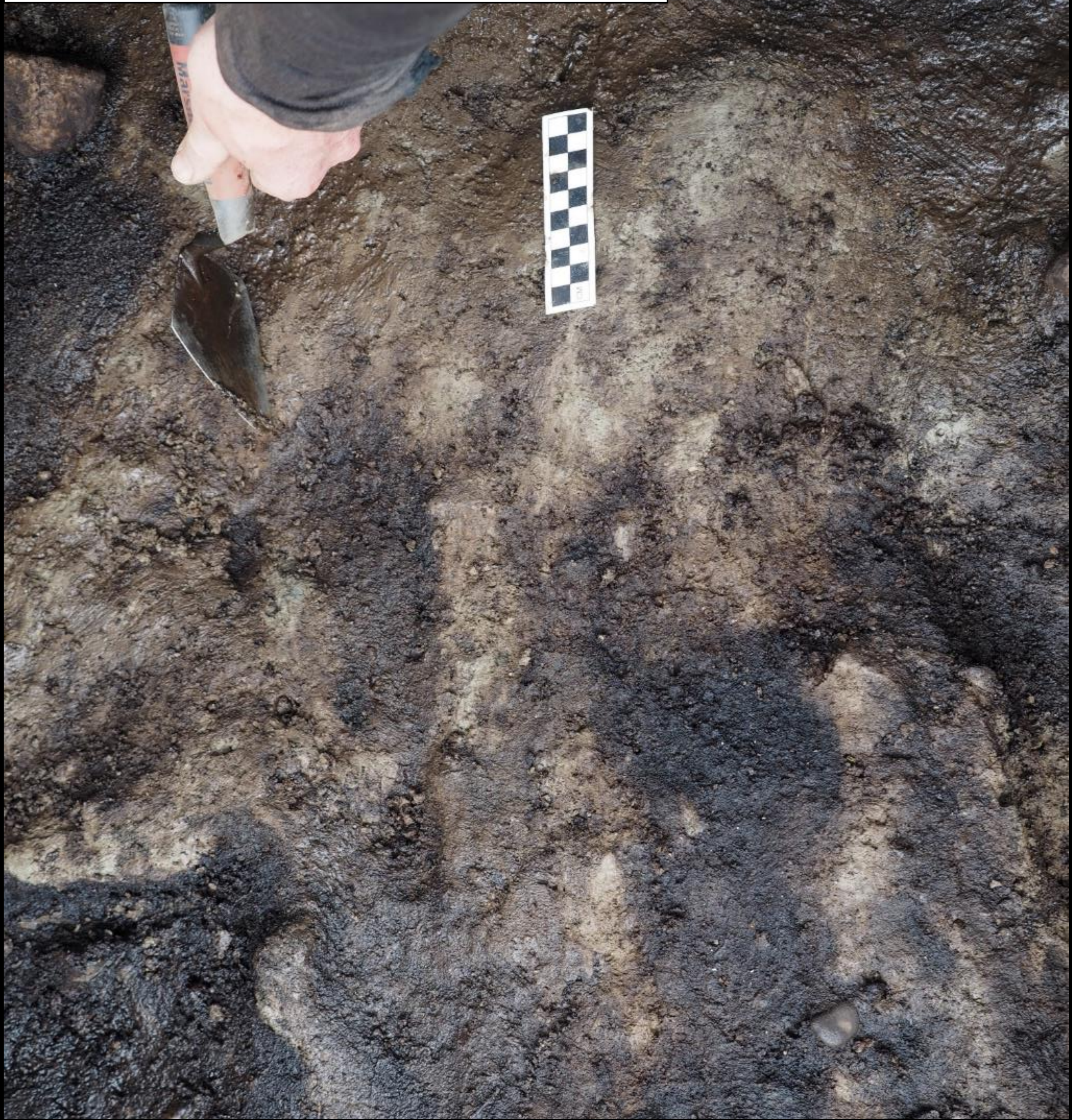

Footprint #17, Tracing F.P. Outline, Photo #7

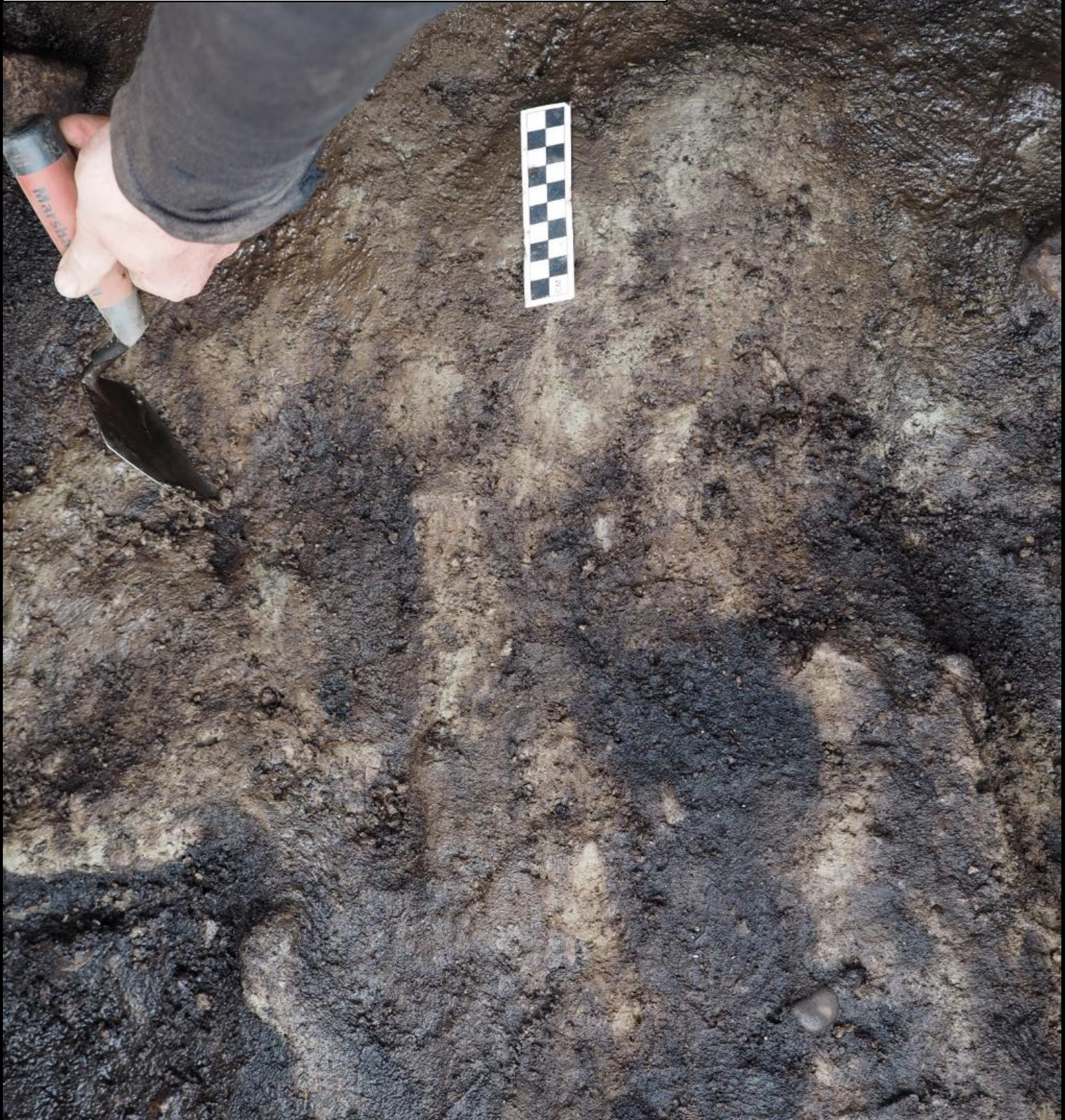

Footprint #17, Tracing F.P. Outline, Photo #8

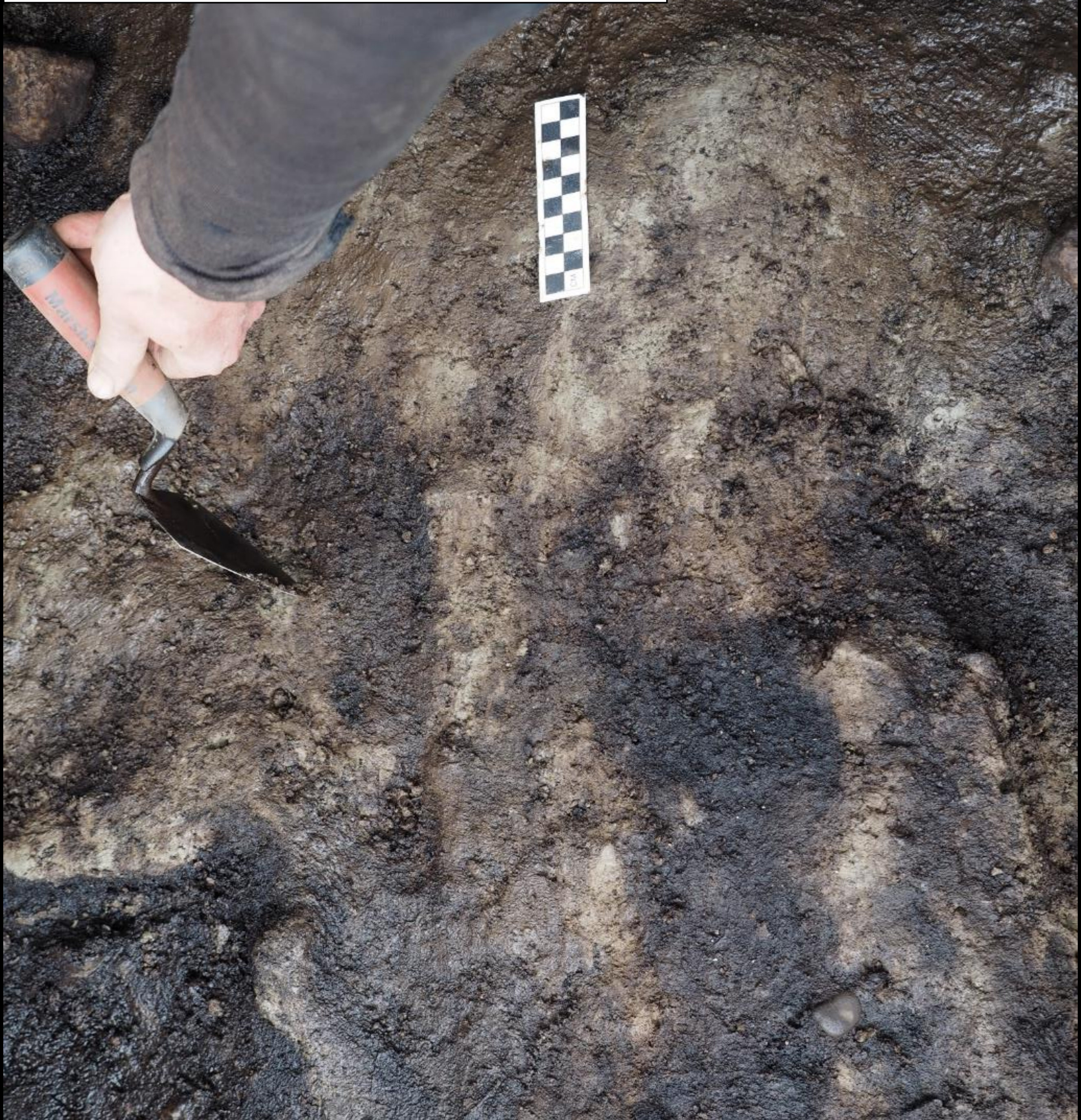

Footprint #20, Photo #1

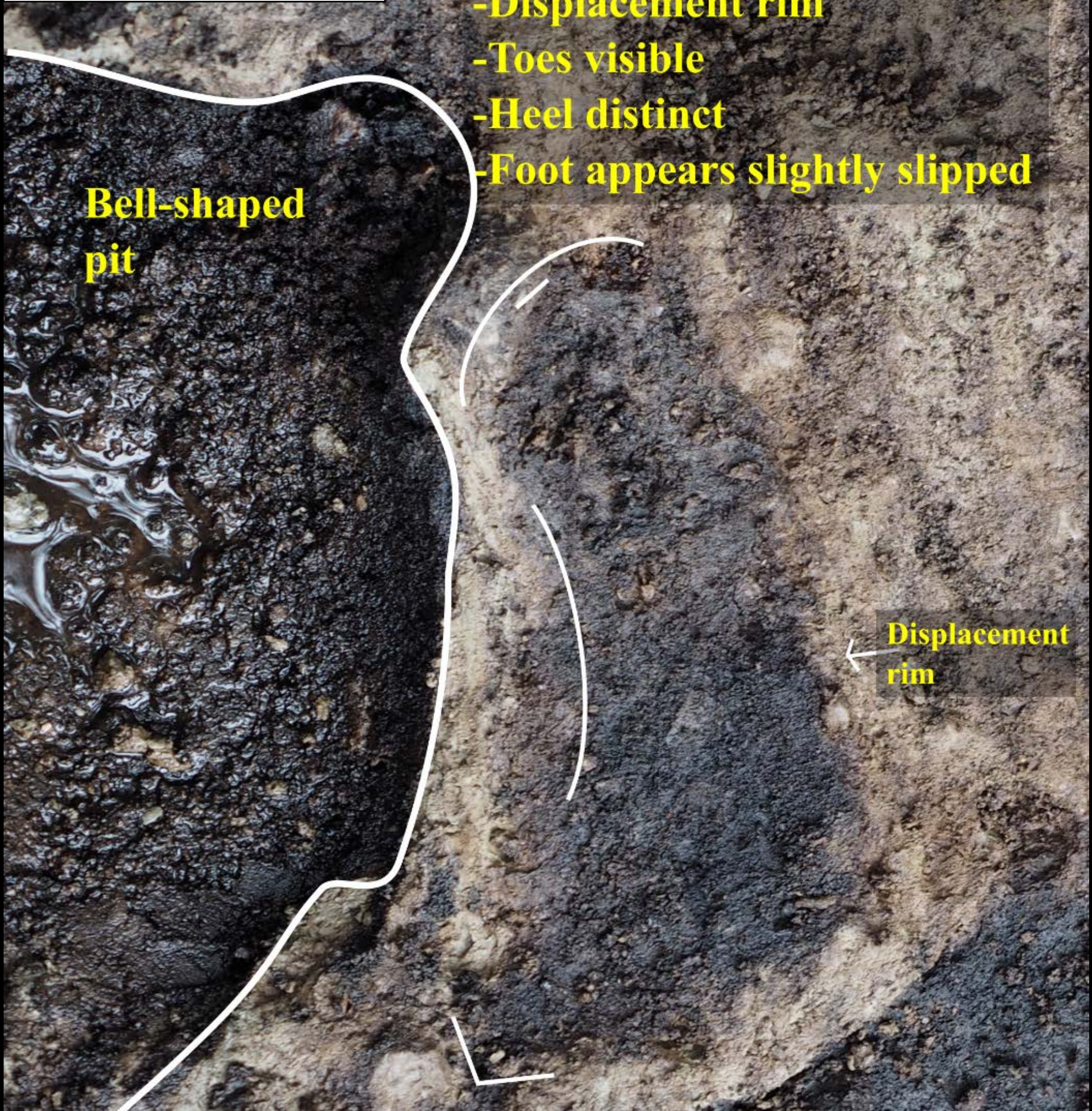

Footprint #20, Photo #2

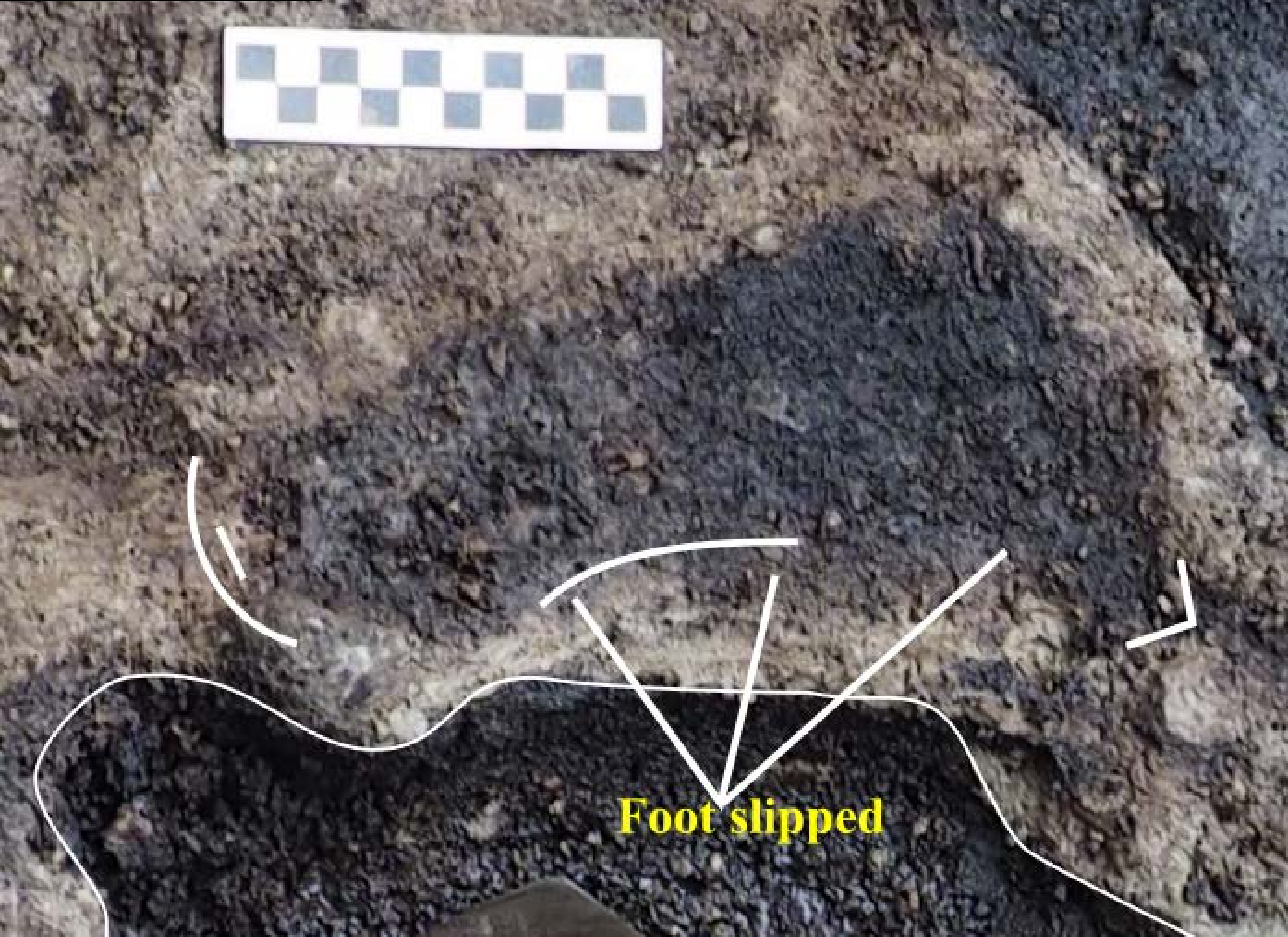

Footprint #20, Photo #3

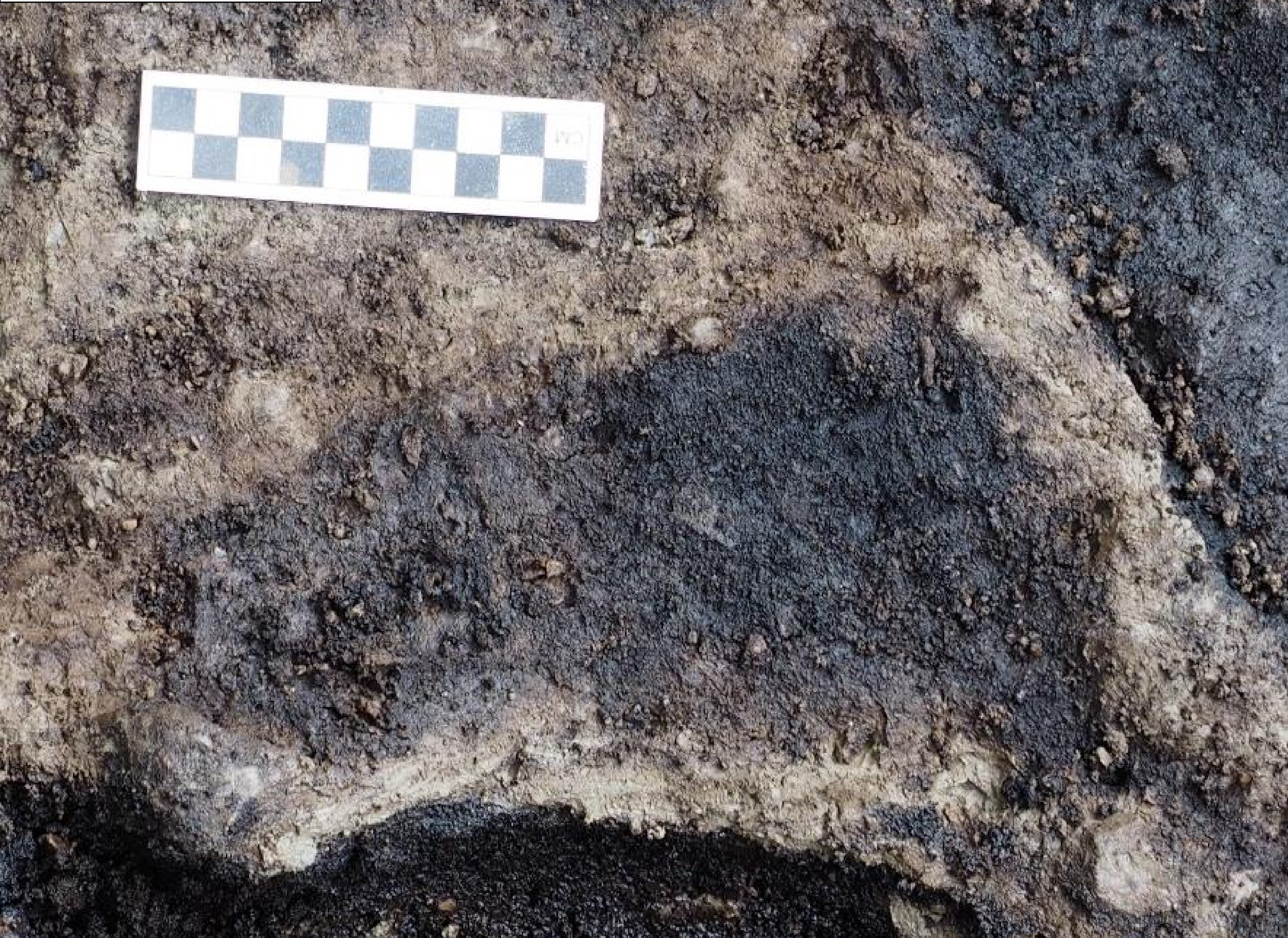

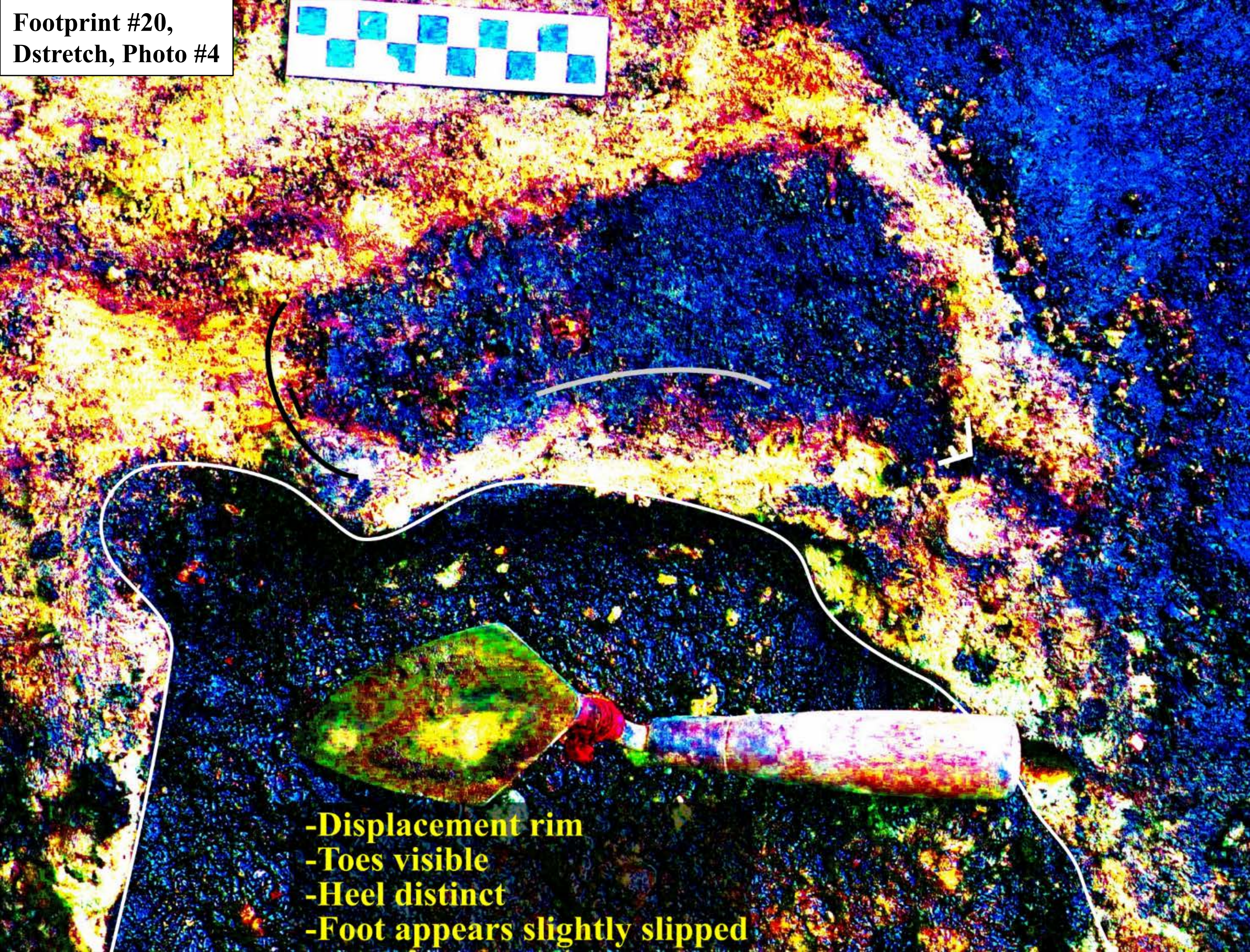
Footprint #20,
Dstretch, Photo #4
-Displacement rim
-Toes visible
-Heel distinct
-Foot appears slightly slipped

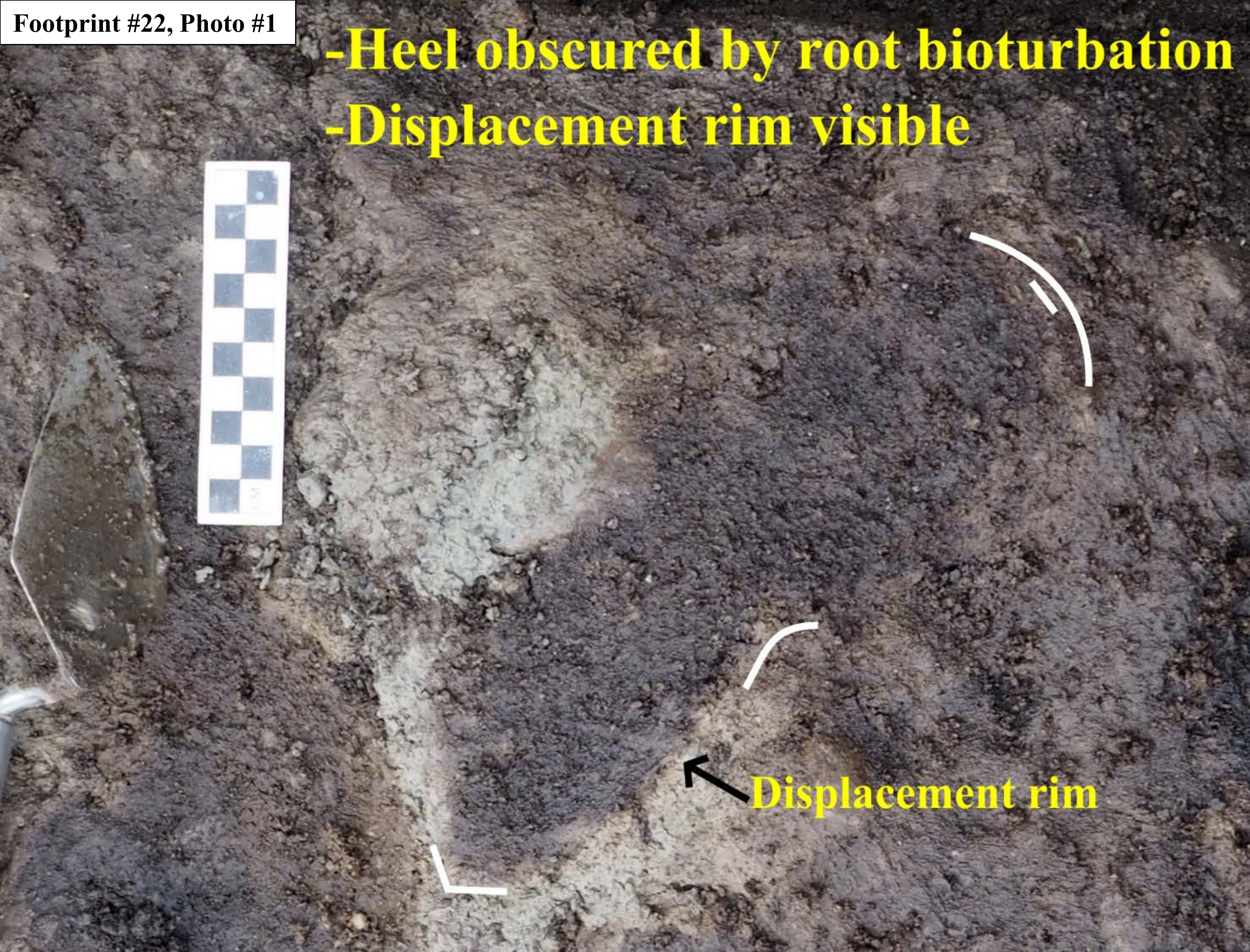
Footprint #22, Photo #1
-Heel obscured by root bioturbation
-Displacement rim visible
Displacement rim

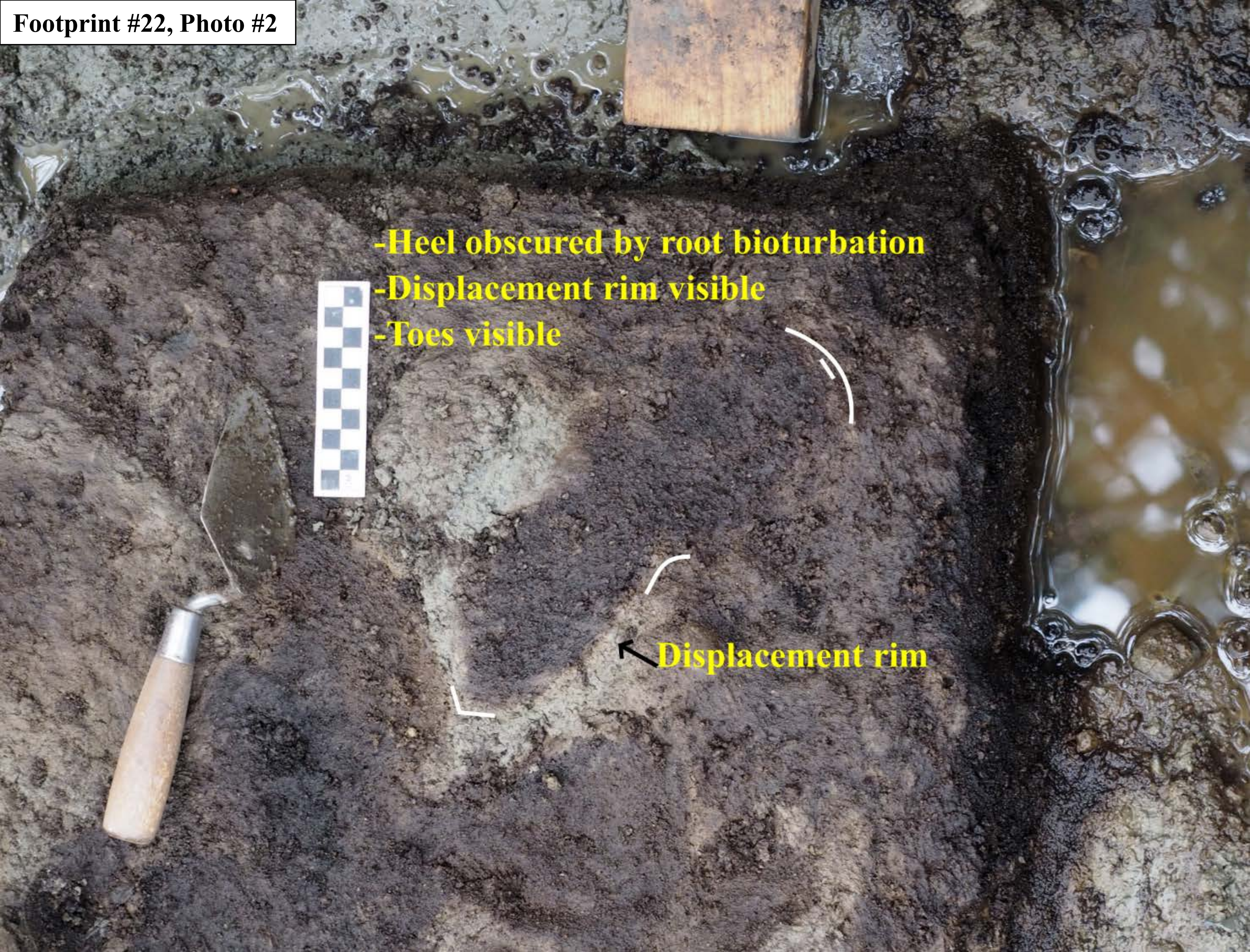
Footprint #22, Photo #2
-Heel obscured by root bioturbation
-Displacement rim visible
-Toes visible
Displacement rim

Footprint #22, Photo #3

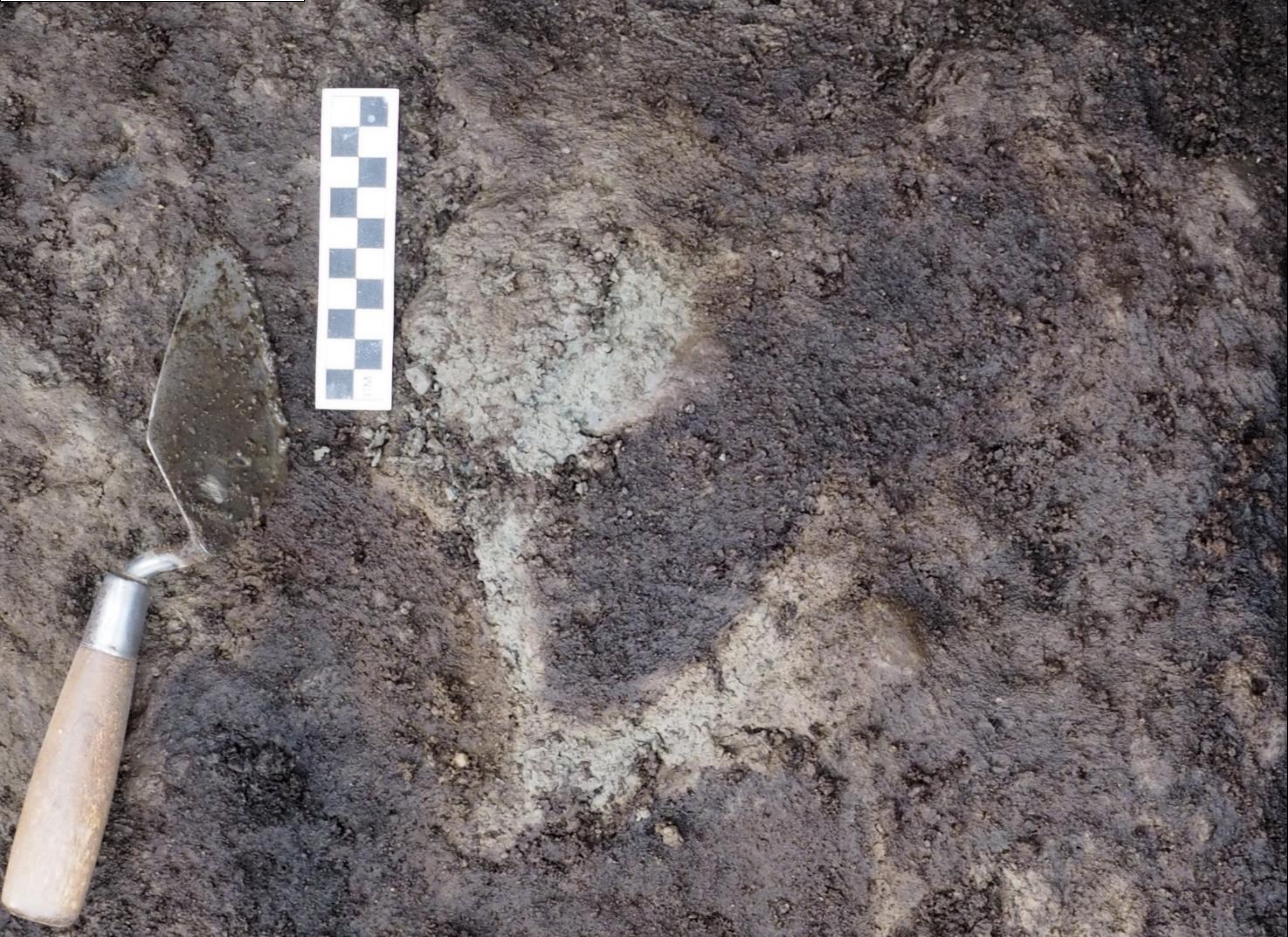

Footprint #22, Dstretch, Photo #4

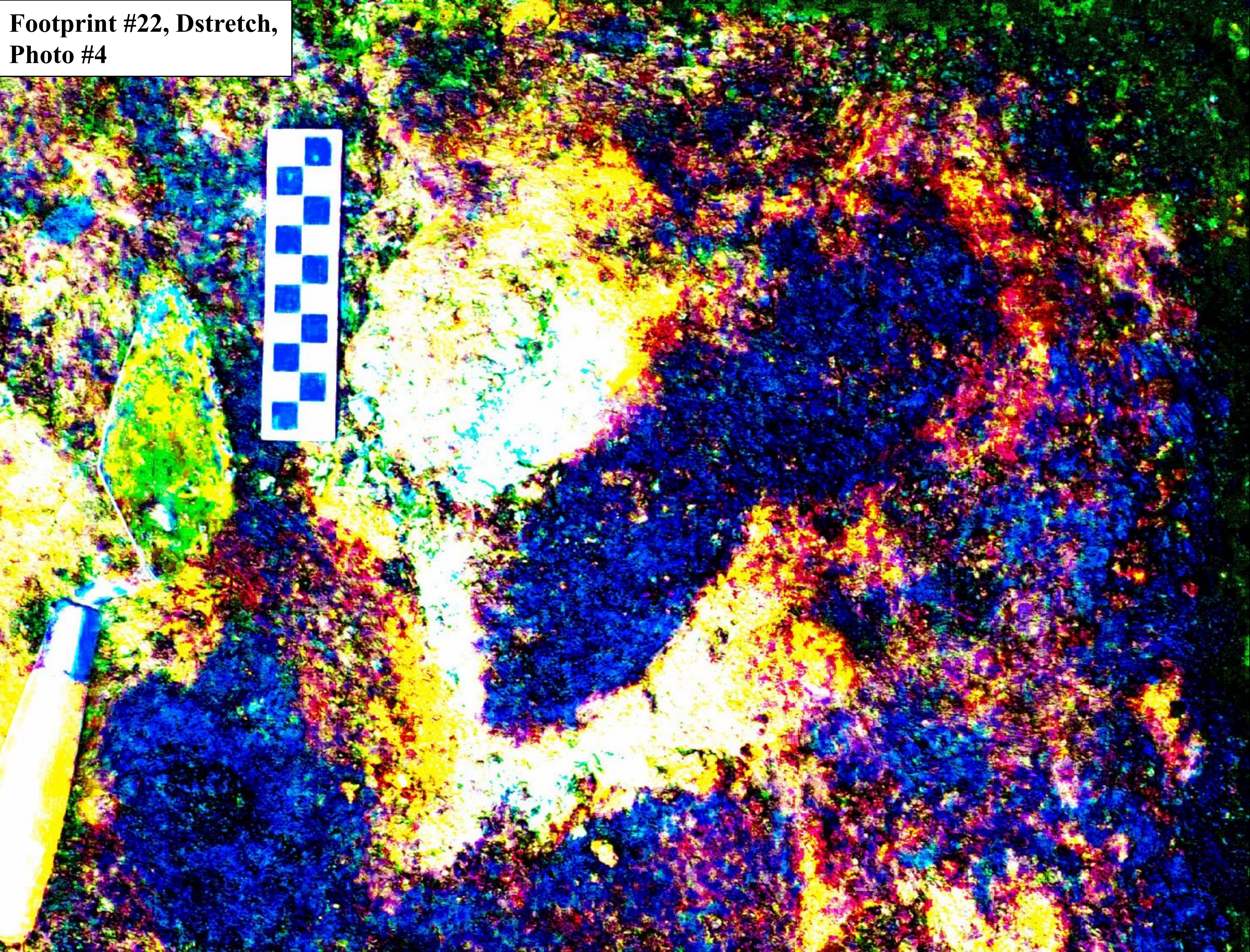

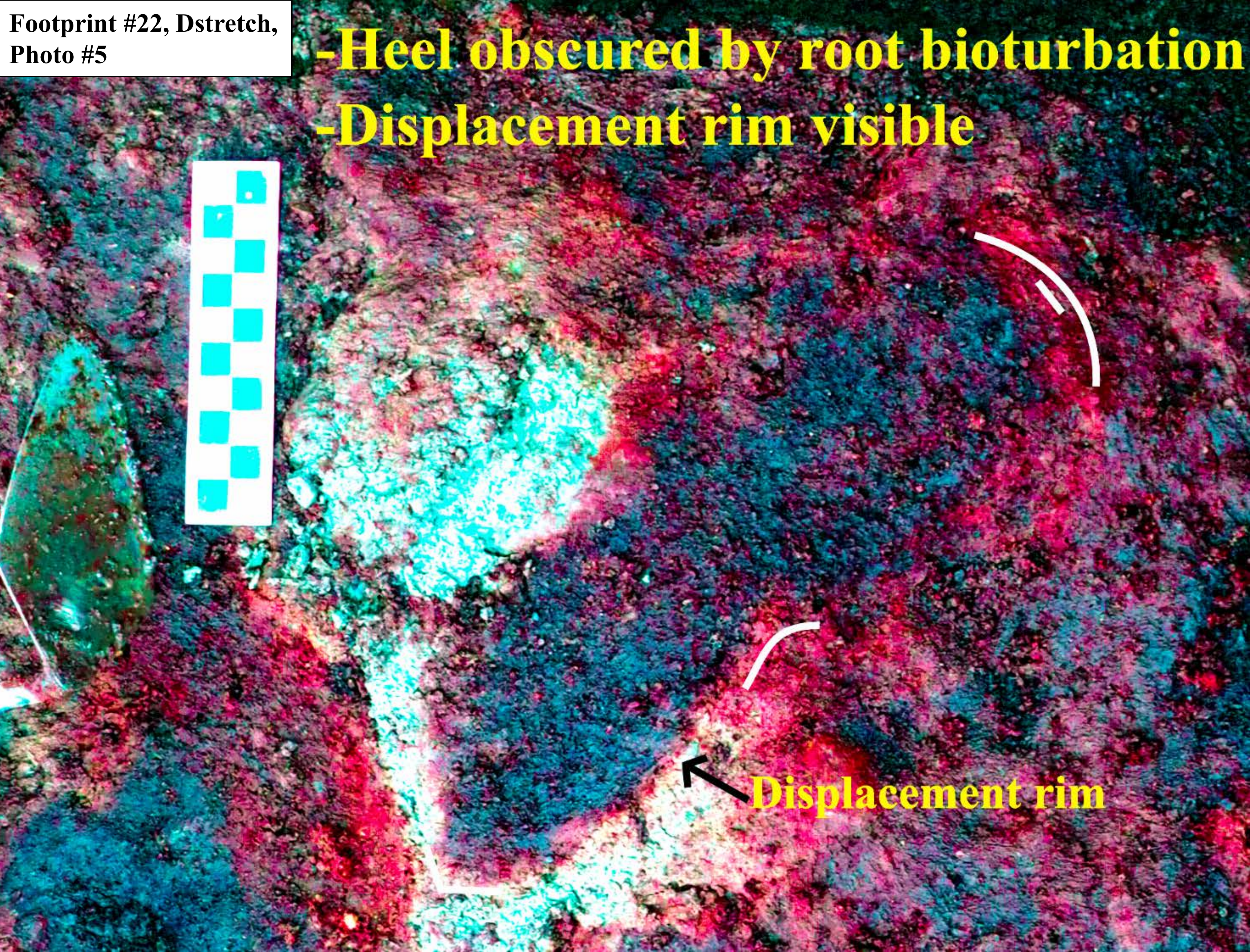
Footprint #22, Dstretch, Photo #5
-Heel obscured by root bioturbation
-Displacement rim visible
Displacement rim

Footprint #12, Photo #1

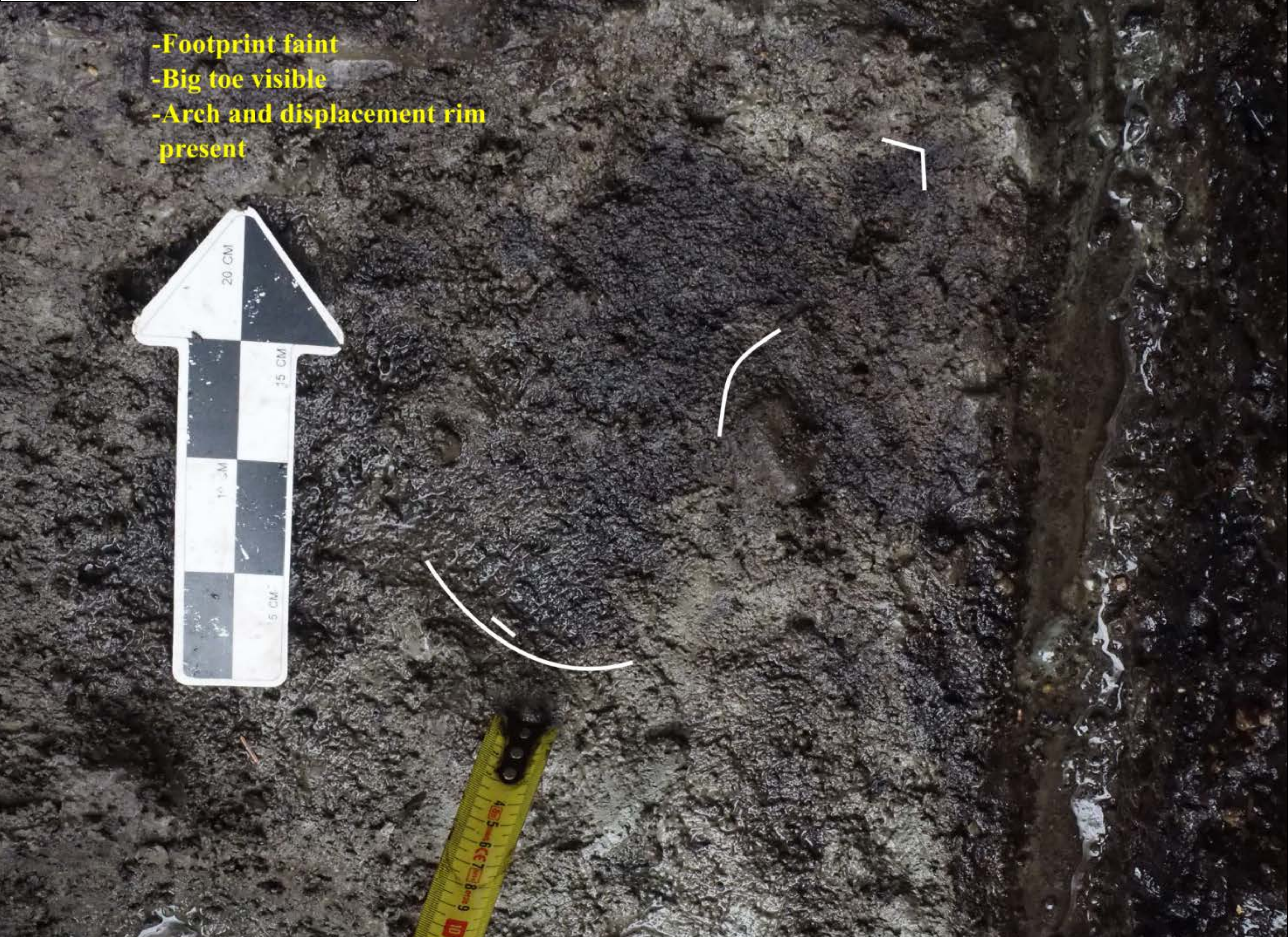

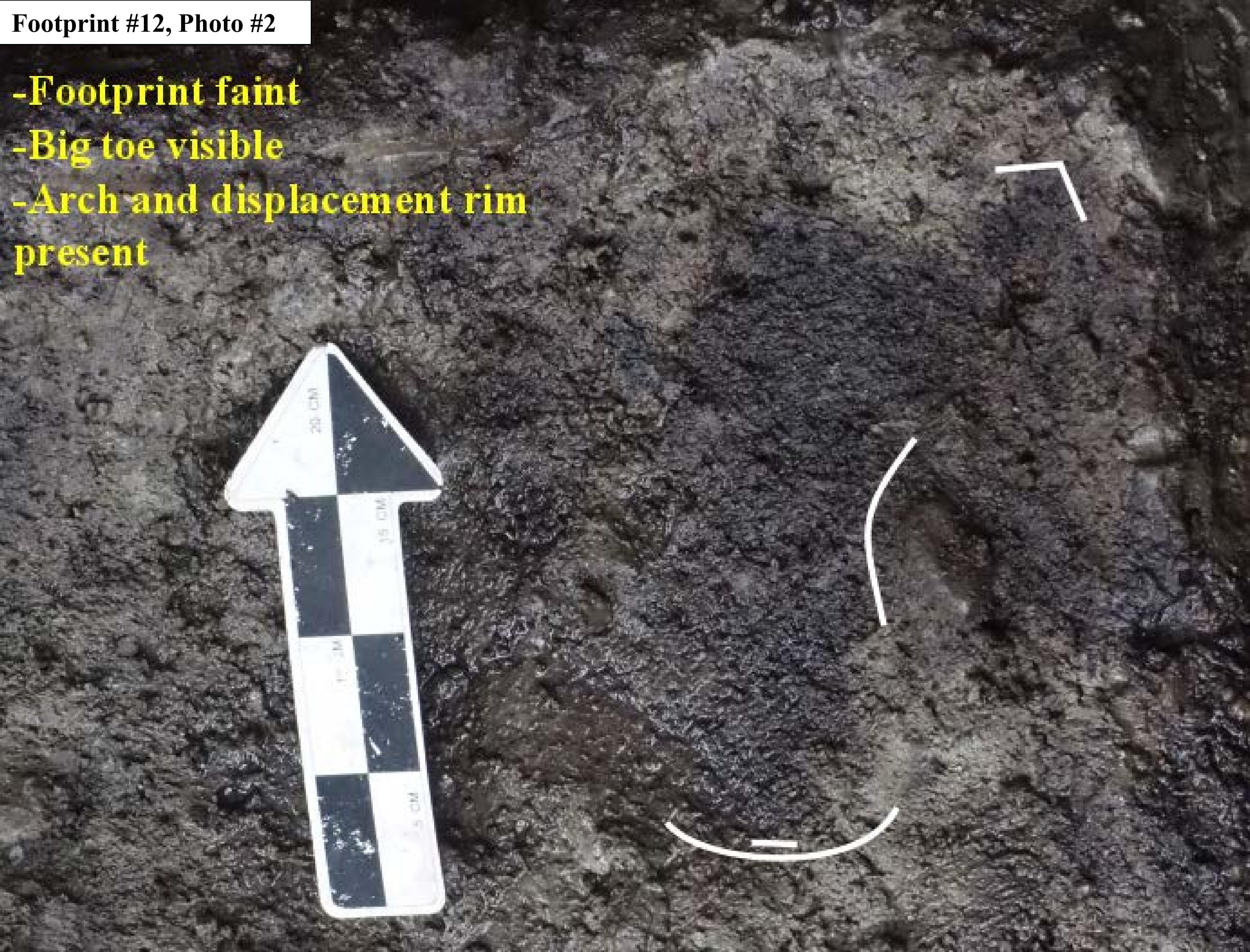
Footprint #12, Photo #2
-Footprint faint
-Big toe visible
-Arch and displacement rim
present

Footprint #12, Photo #3

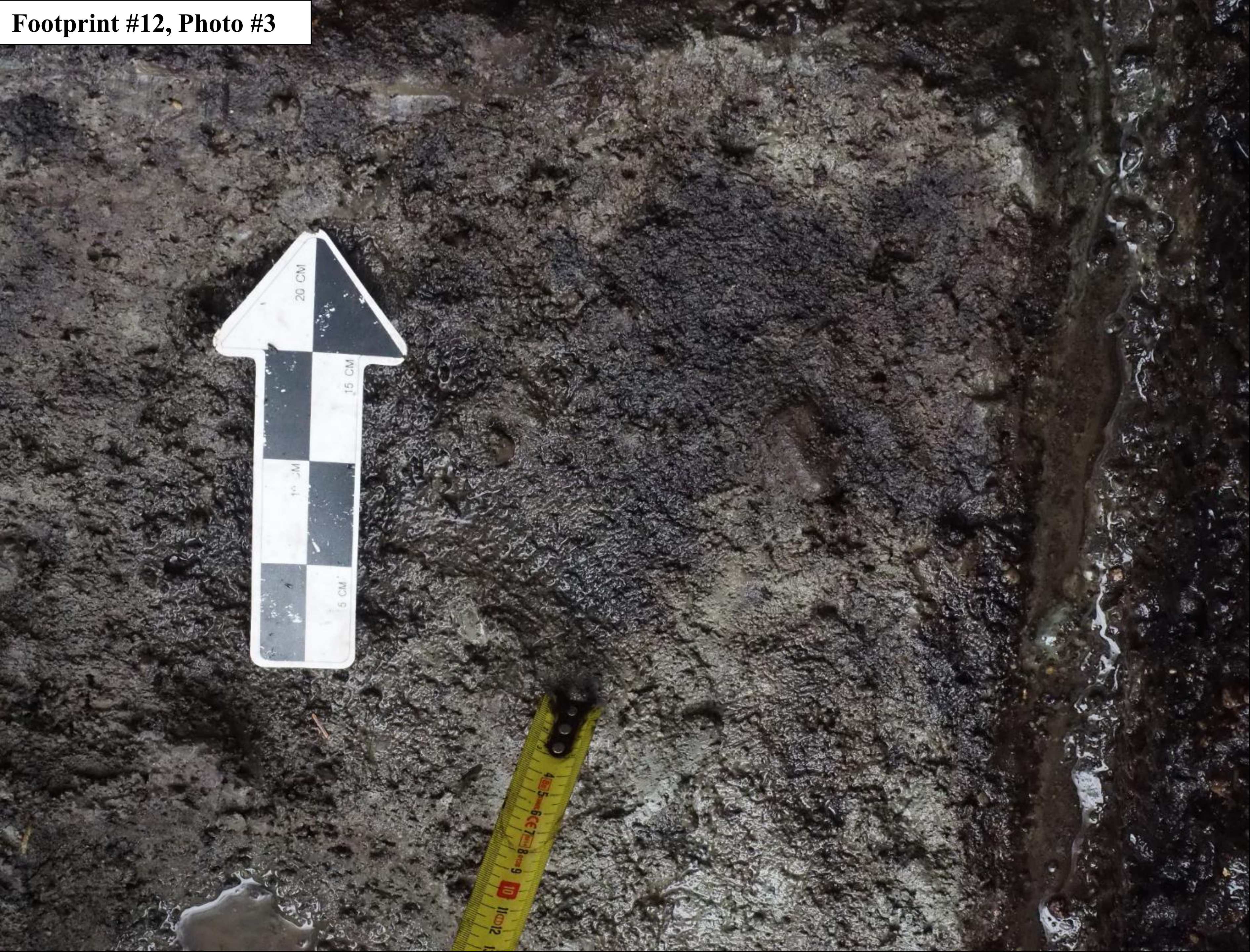

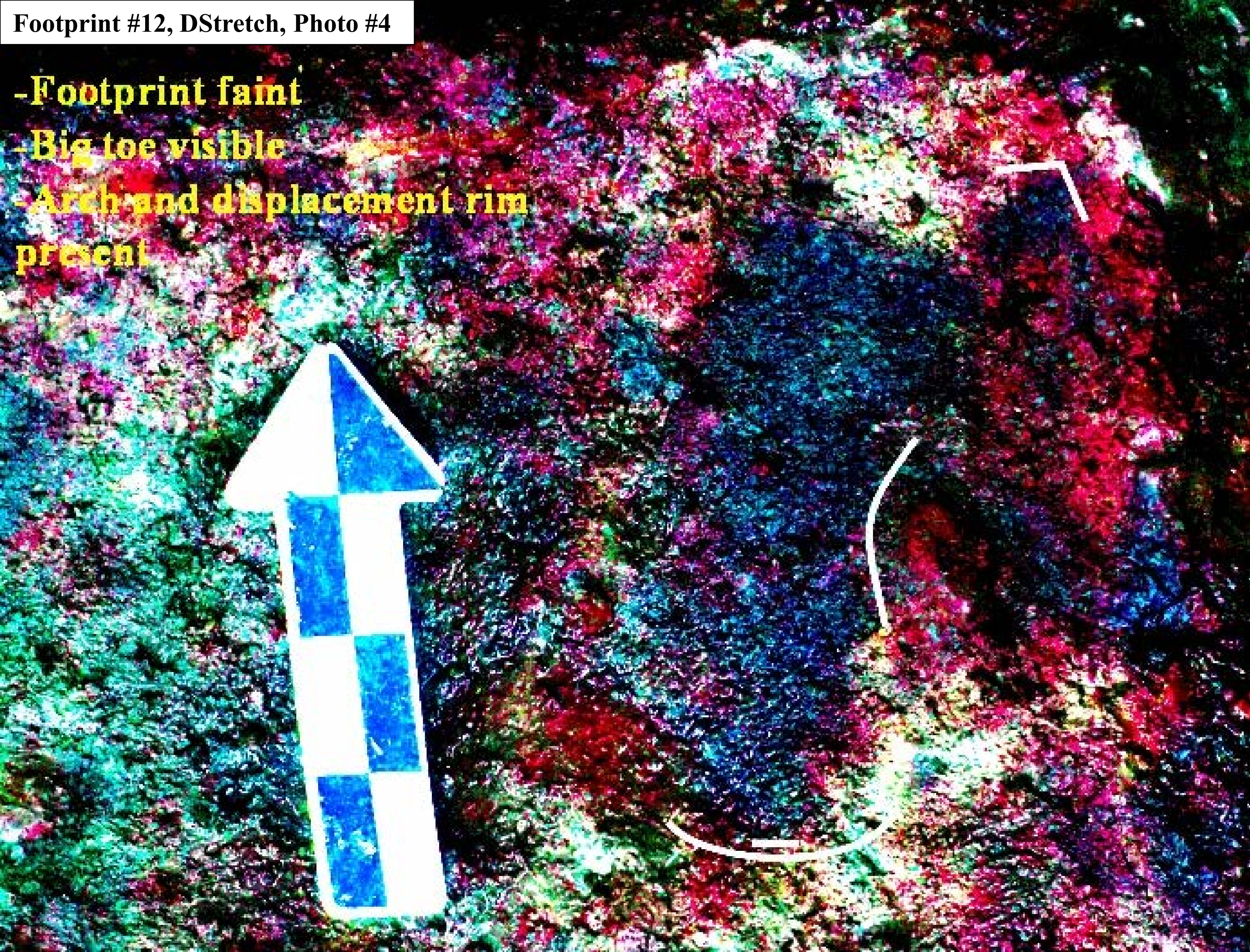
Footprint #12, DStretch, Photo #4
-Footprint faint
-Big toe visible
-Arch and displacement rim present

Footprint #21, Photo #1

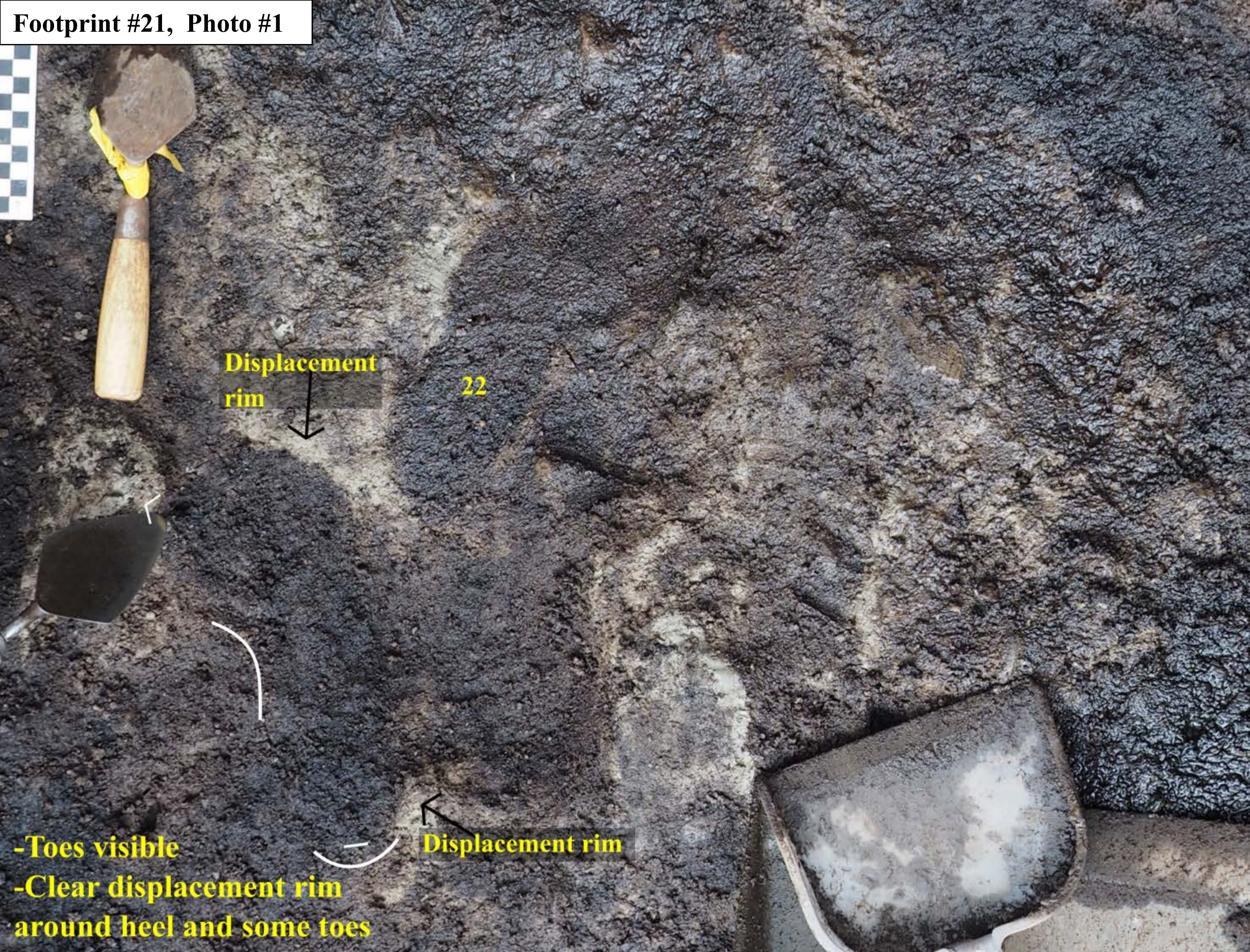

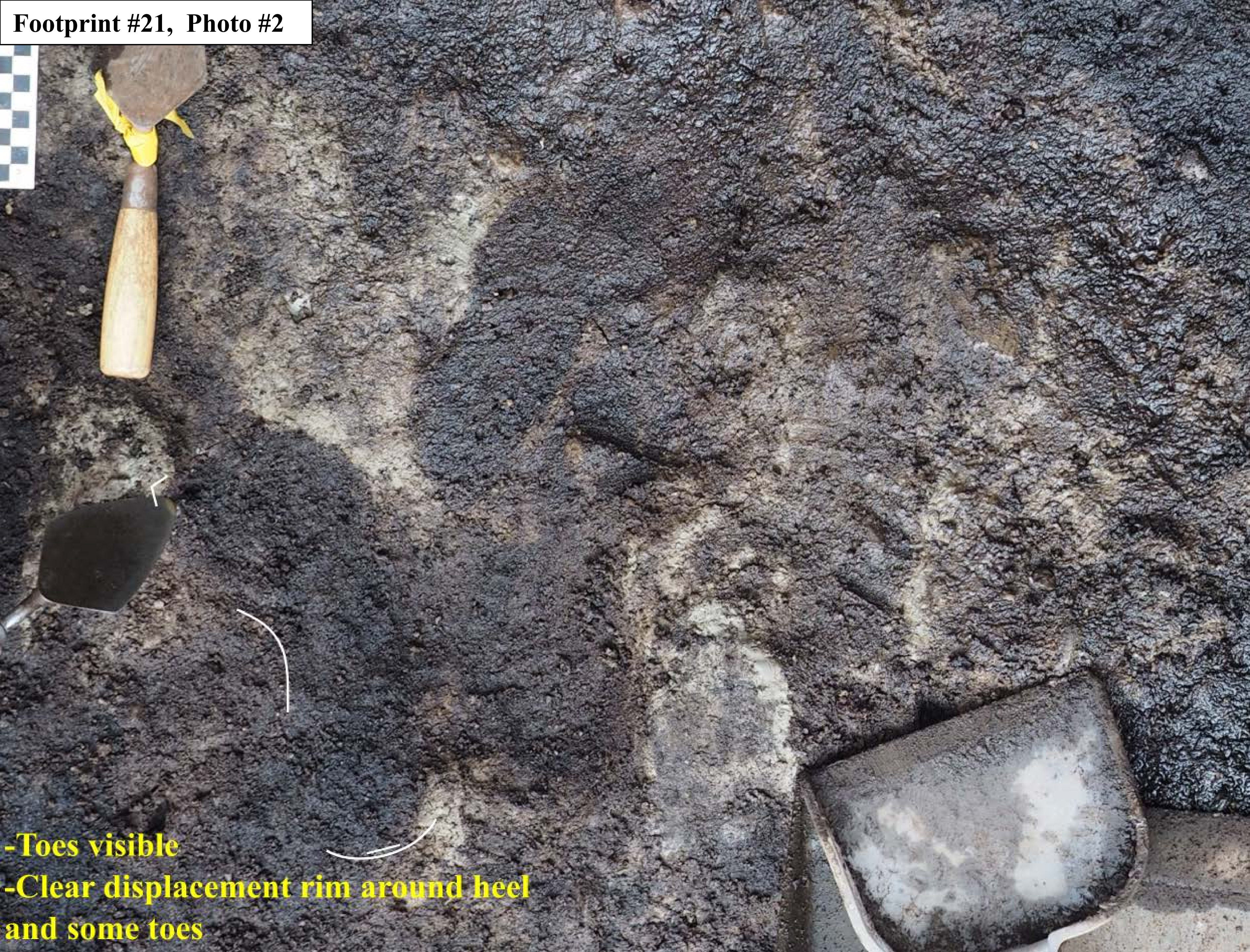
Footprint #21, Photo #2
-Toes visible
-Clear displacement rim around heel
and some toes

Footprint #21, Photo #3

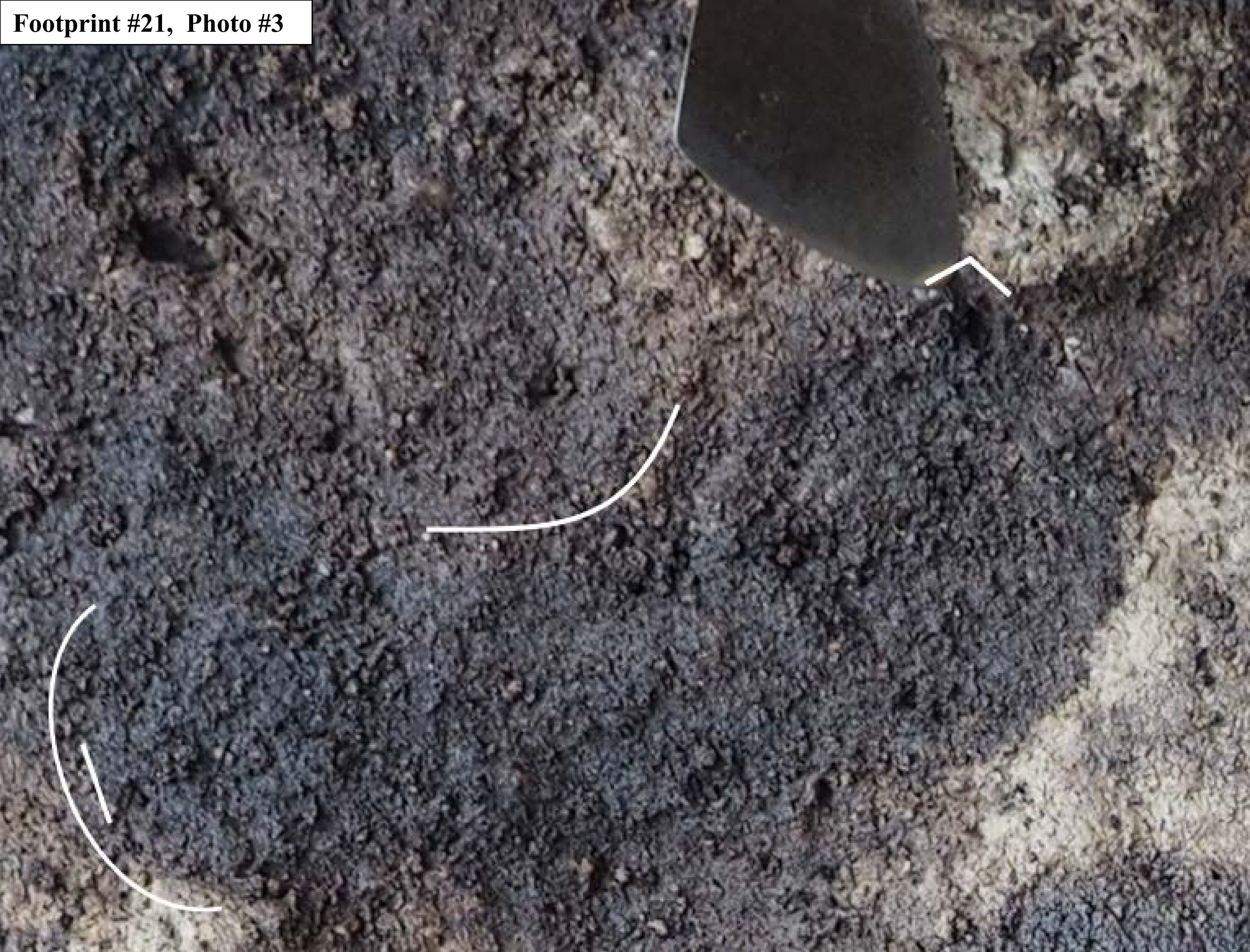

Footprint #21, DStretch, Photo #4

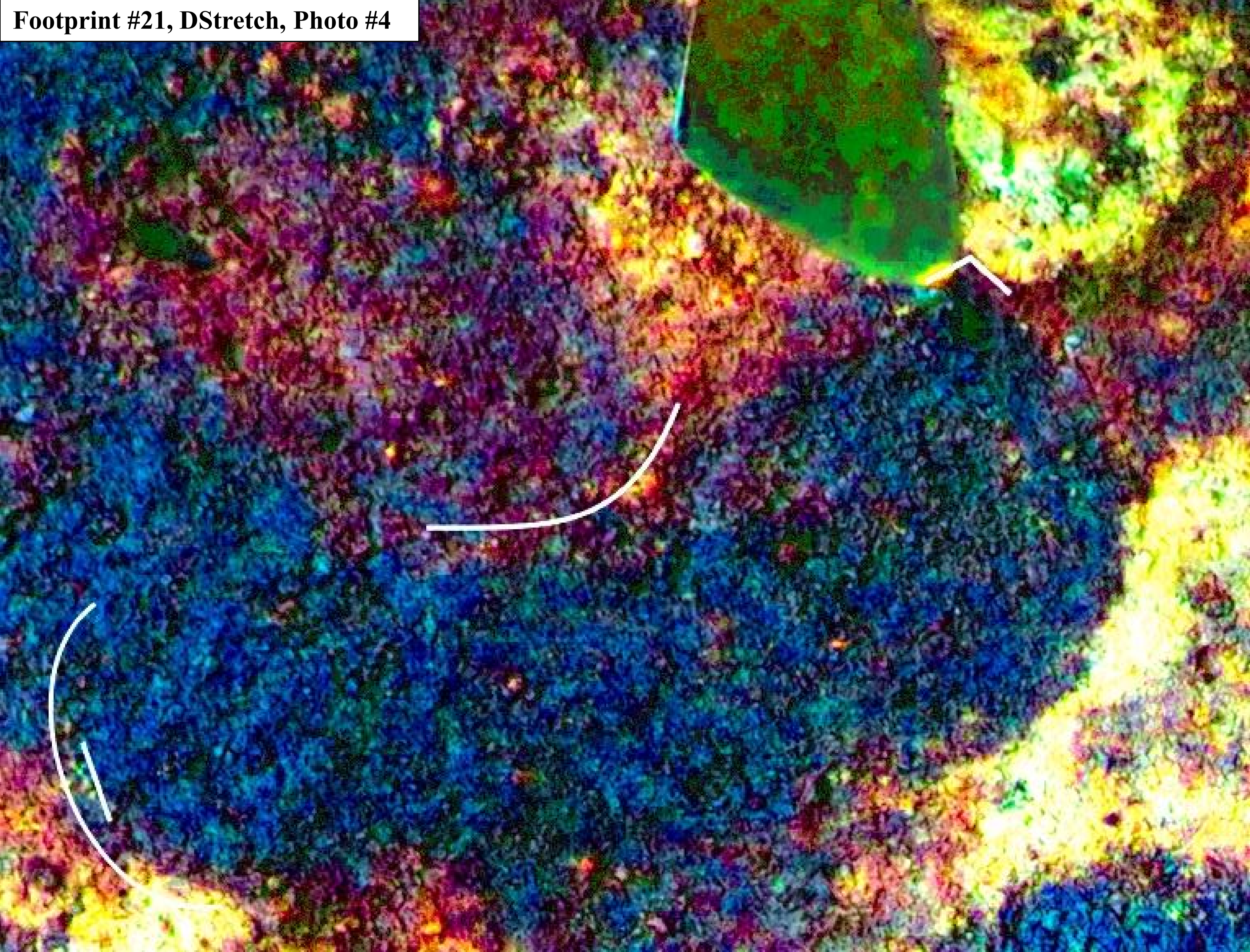

Footprint #21, Tracing F.P. Outline, Photo #5

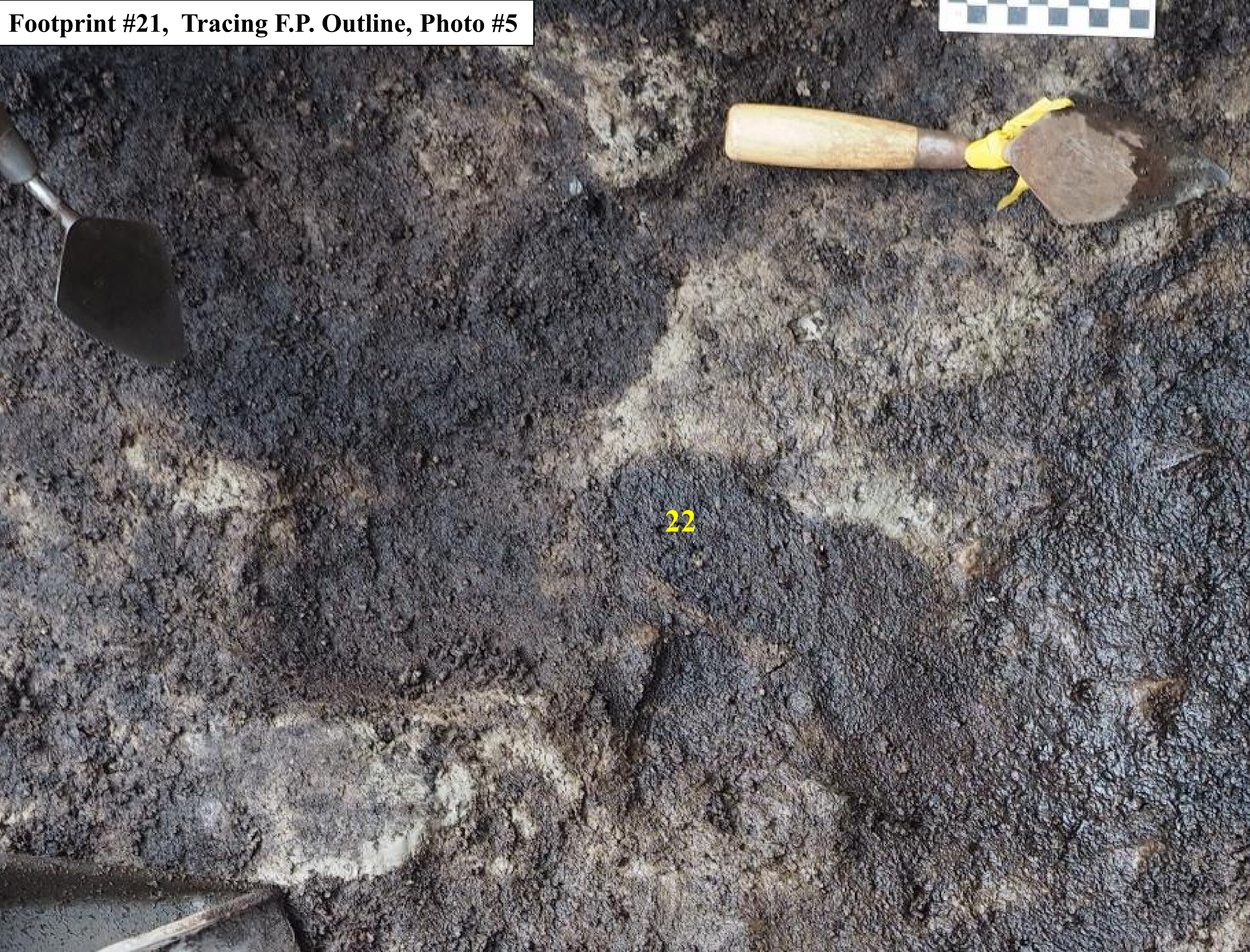

Footprint #21, Tracing F.P. Outline, Photo #6

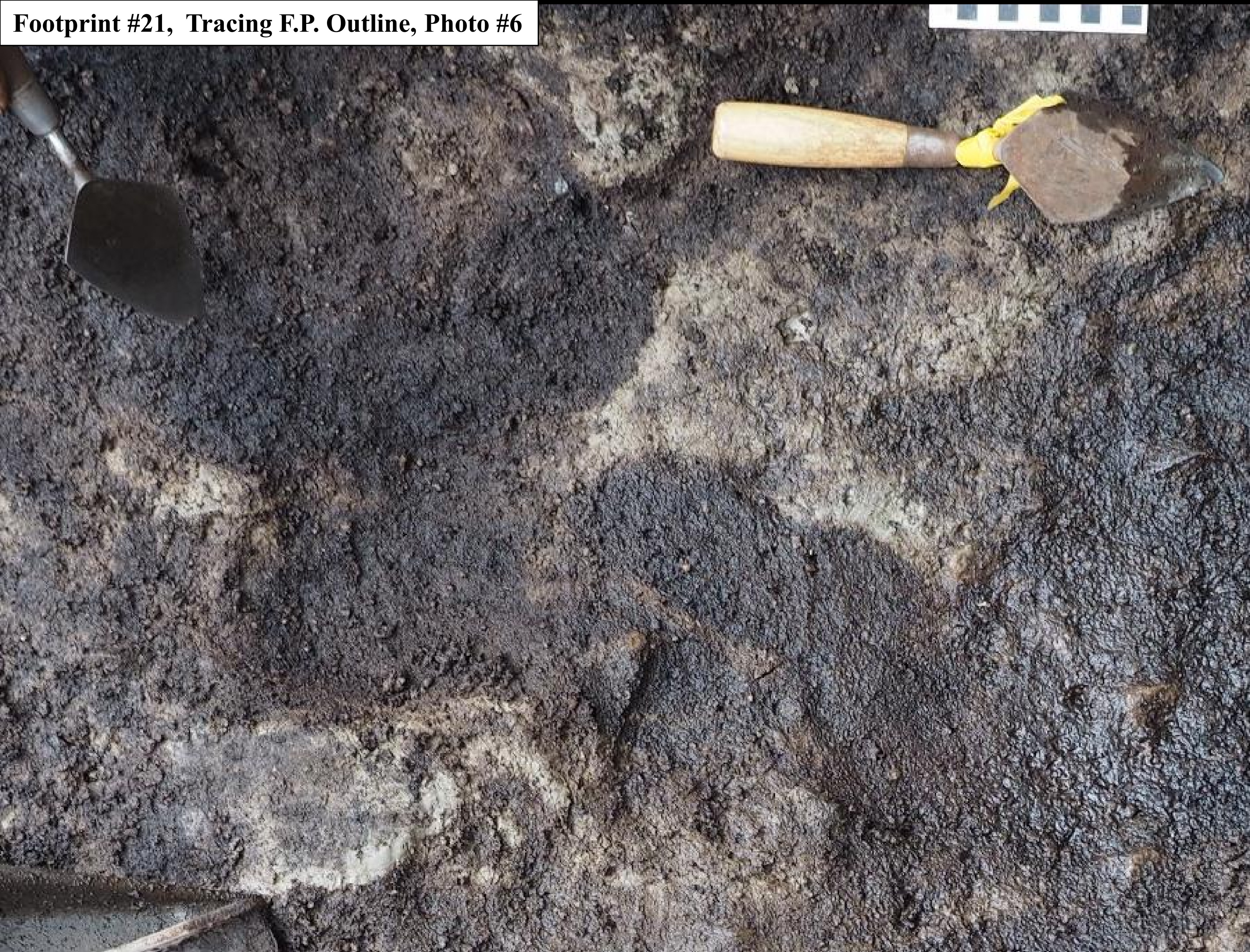

Footprint #21, Tracing F.P. Outline, Photo #7

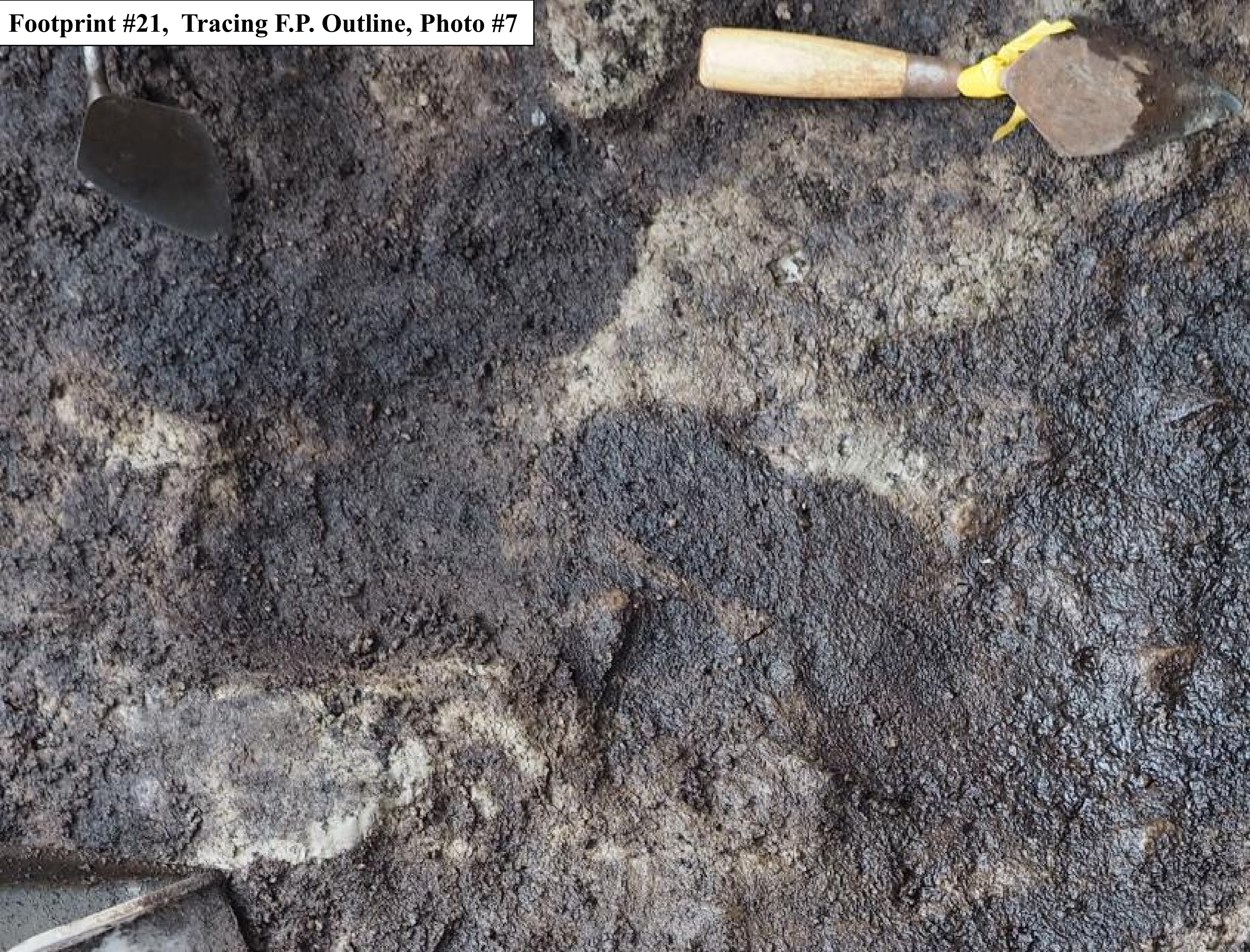

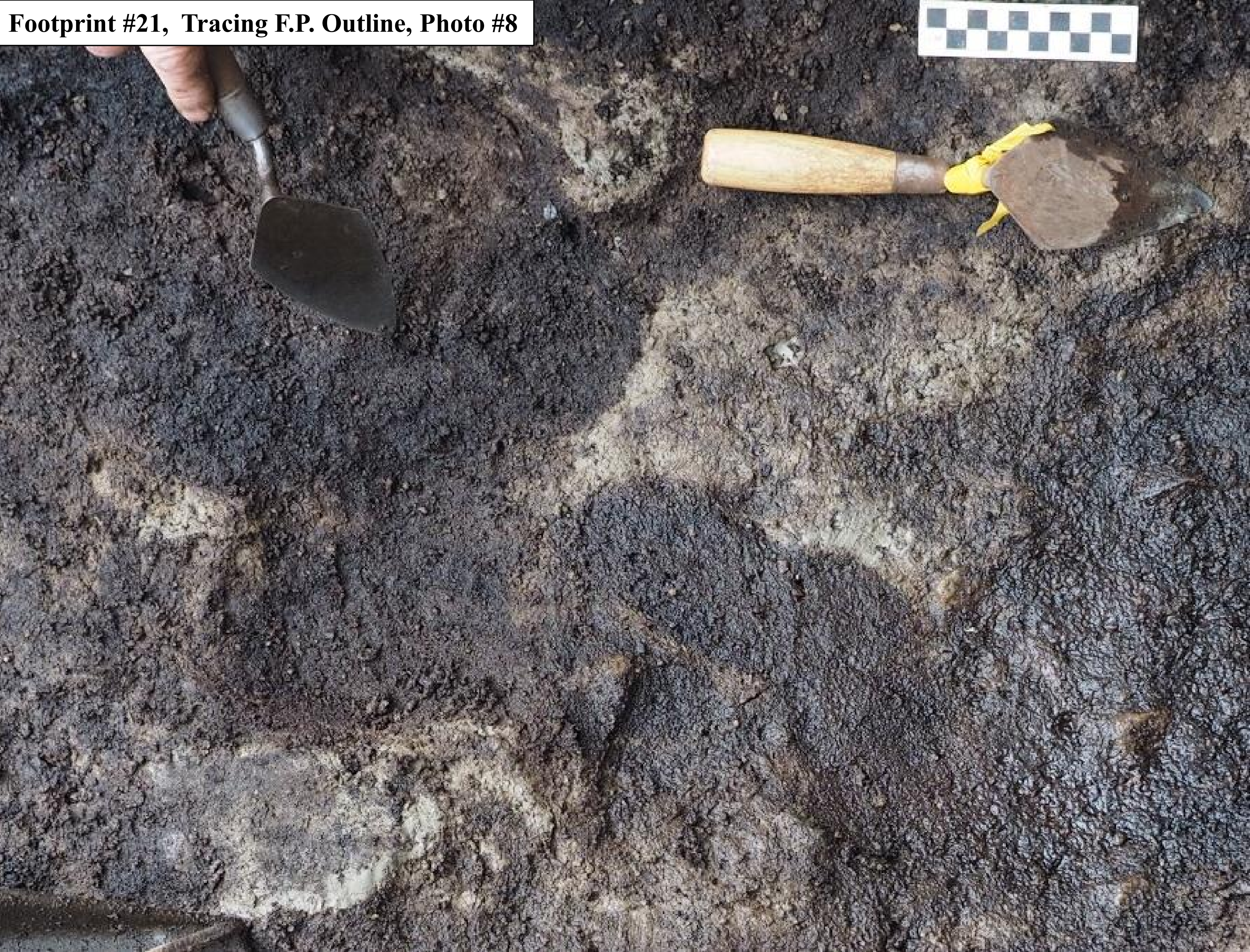

Footprint #21, Tracing F.P. Outline, Photo #8

Footprint #21, Tracing F.P. Outline, Photo #9

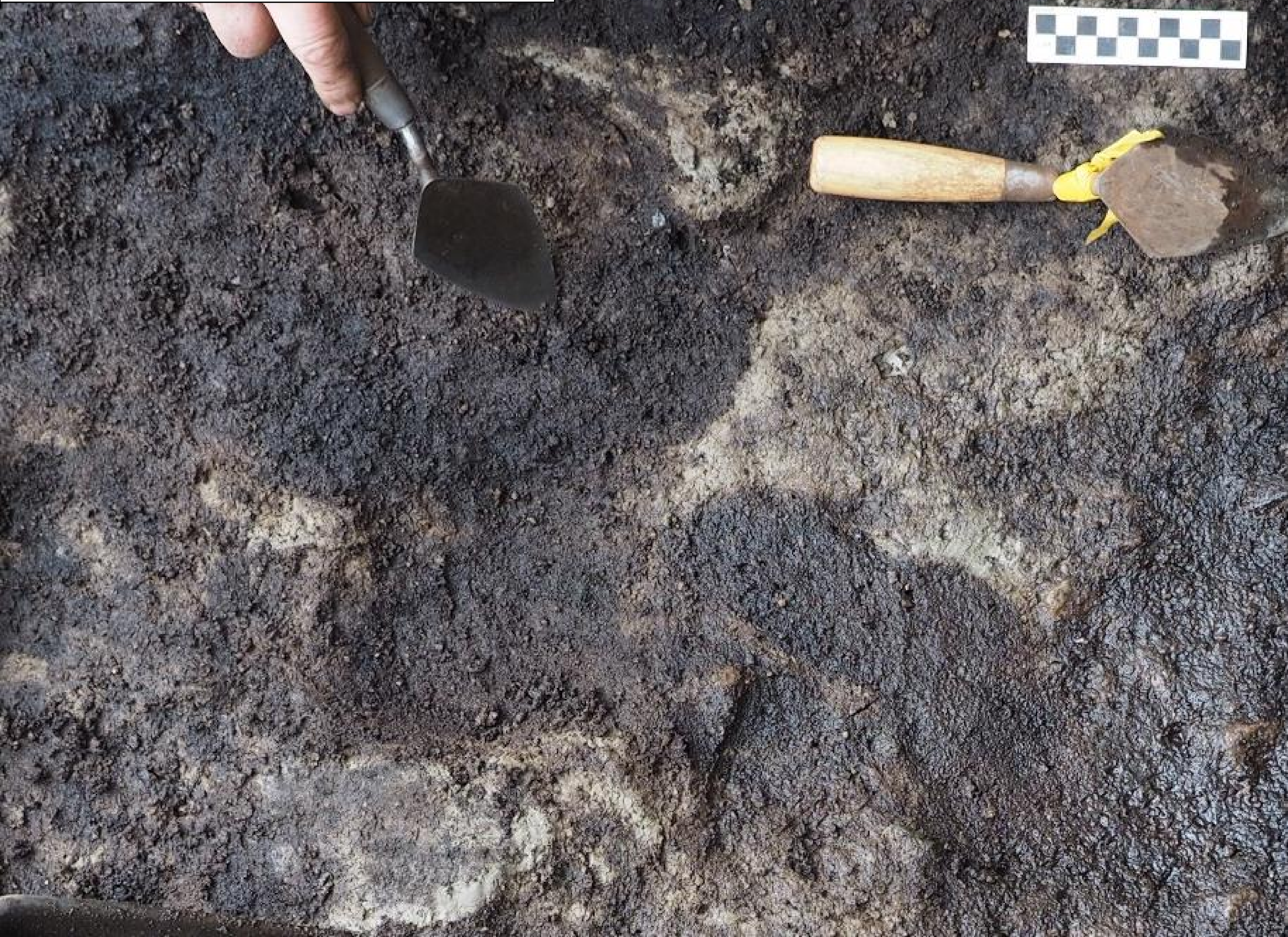

Footprint #21, Tracing F.P. Outline, Photo #10

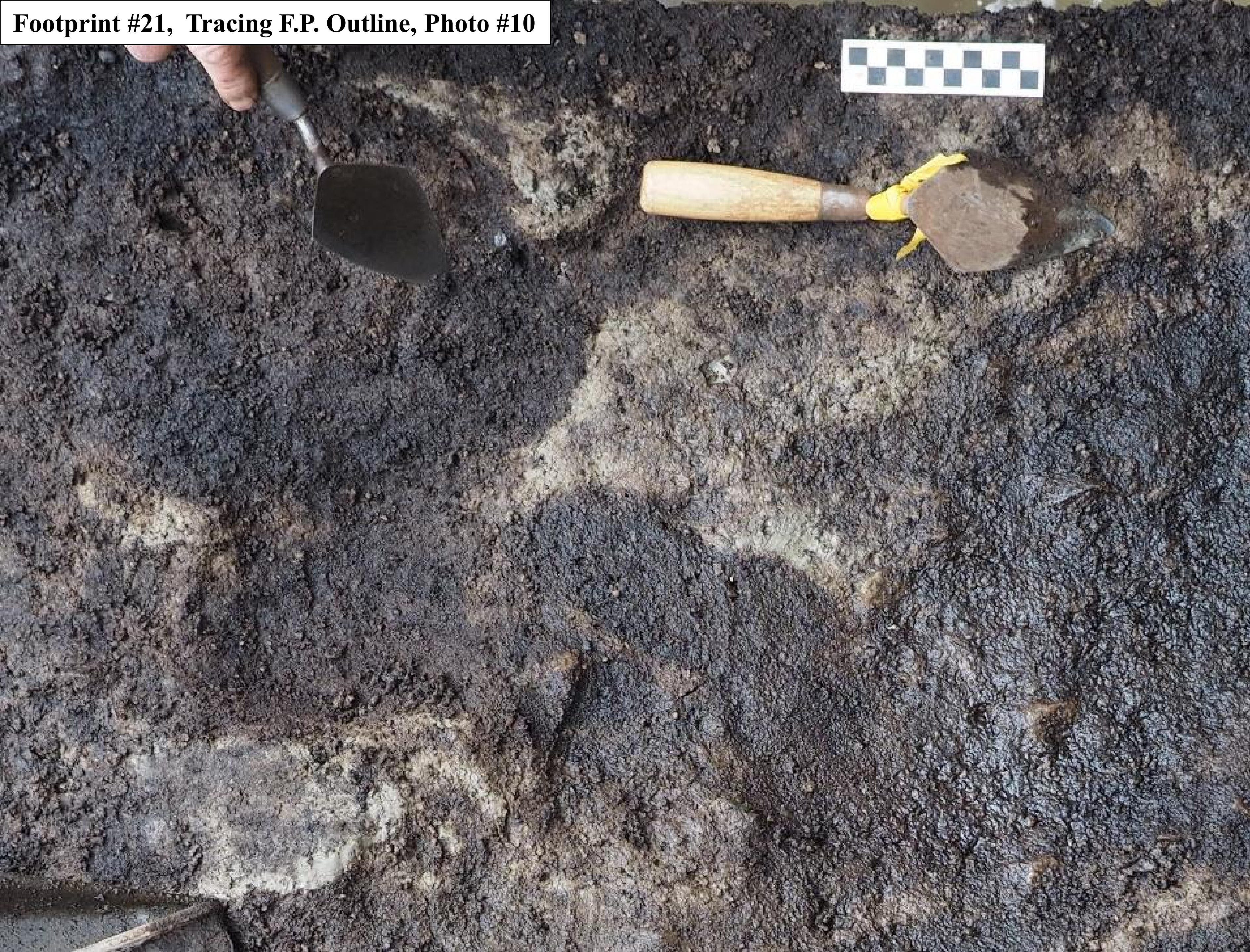

Footprint #21, Tracing F.P. Outline, Photo #11

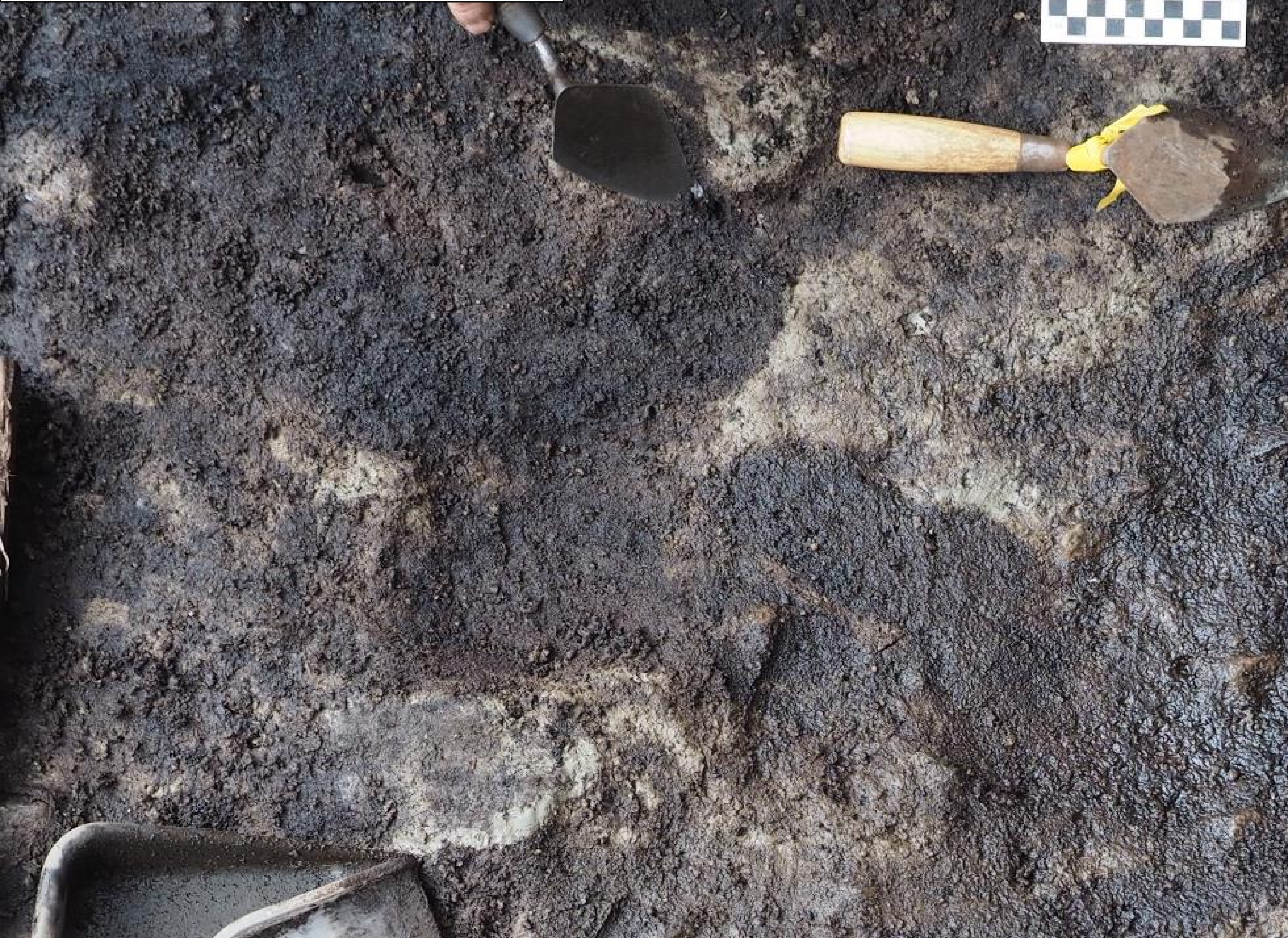

Footprint #21, Tracing F.P. Outline, Photo #12

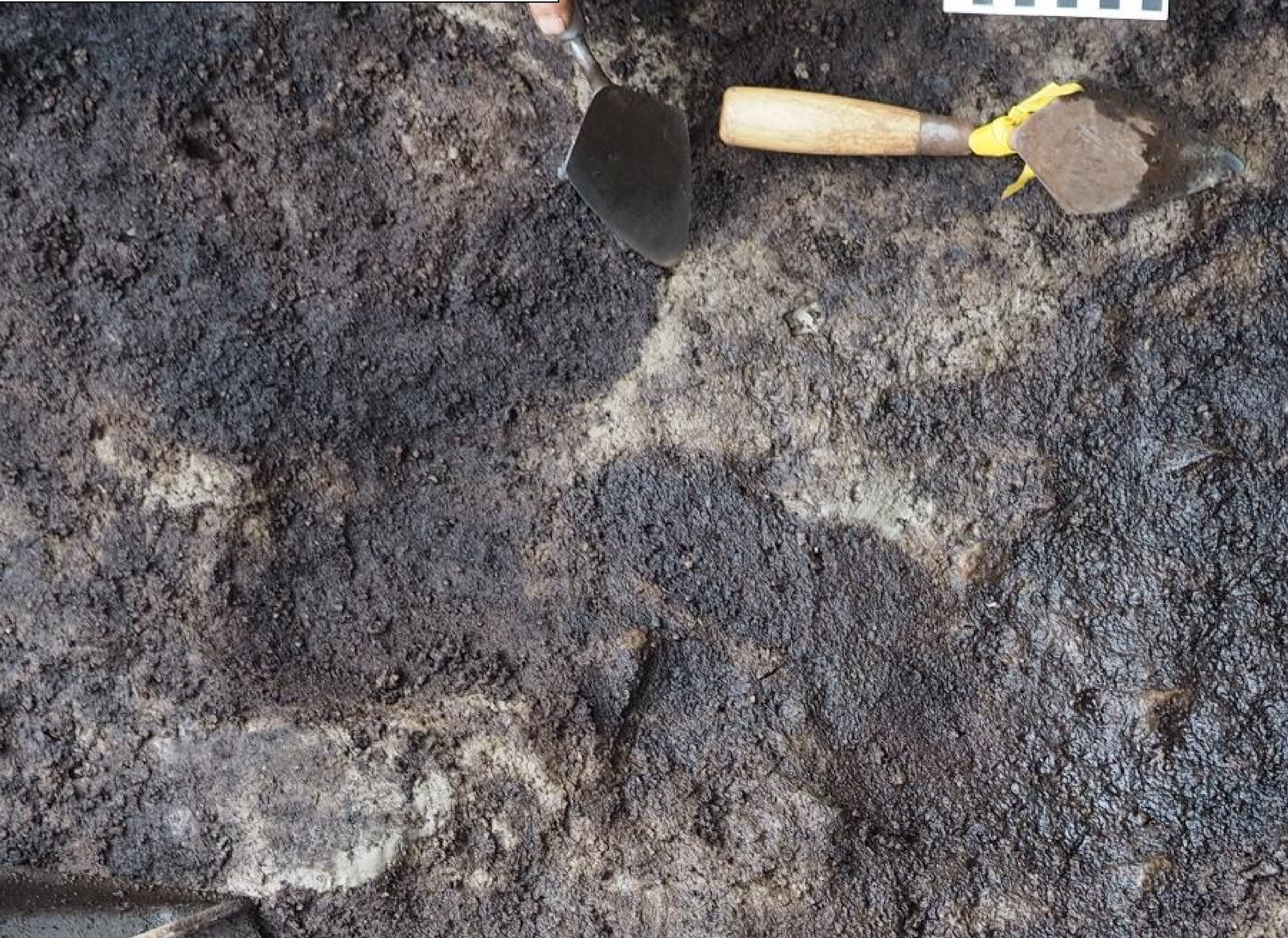

Footprint #21, Tracing F.P. Outline, Photo #13

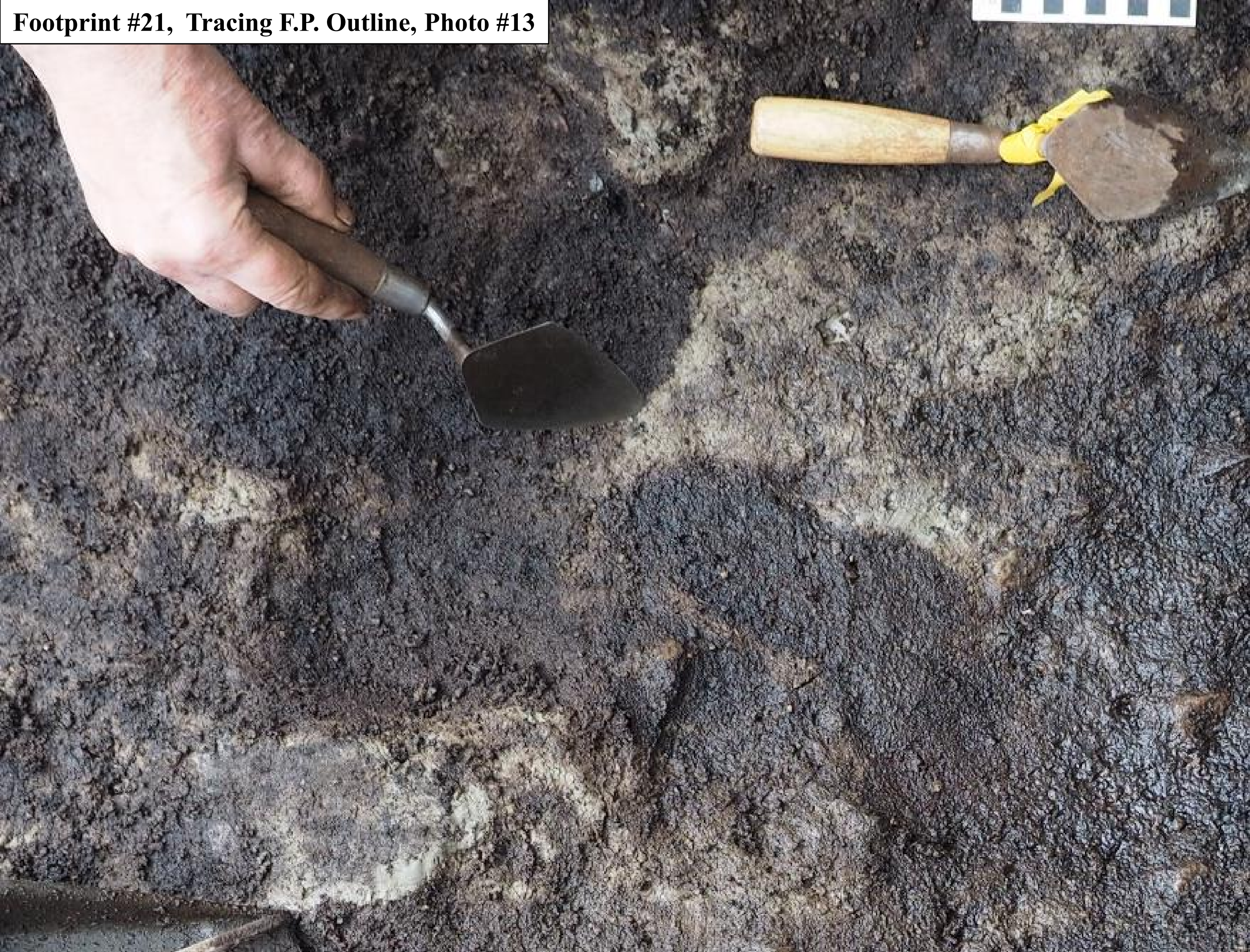

Footprint #21, Tracing F.P. Outline, Photo #14

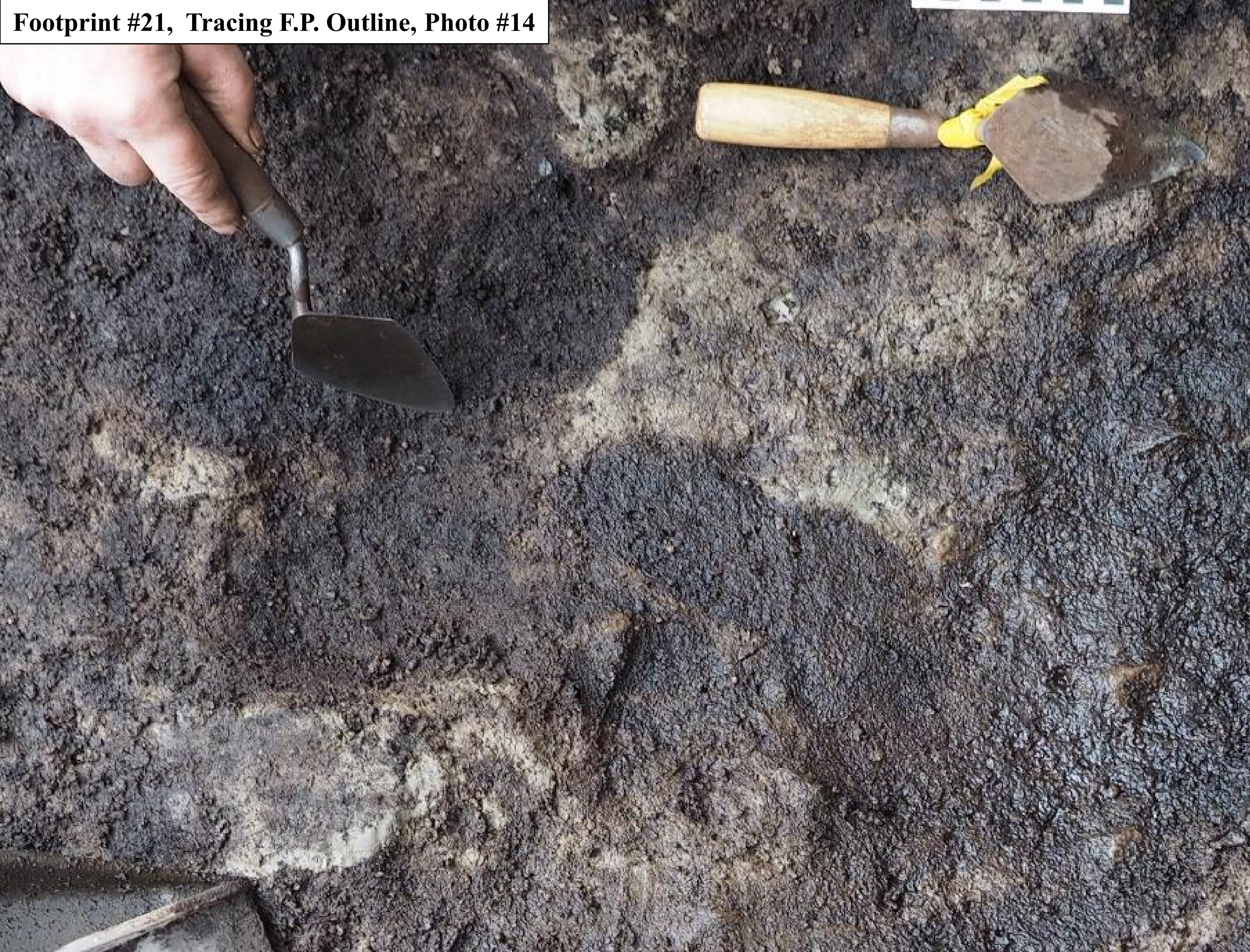

Footprint #21, Tracing F.P. Outline, Photo #15

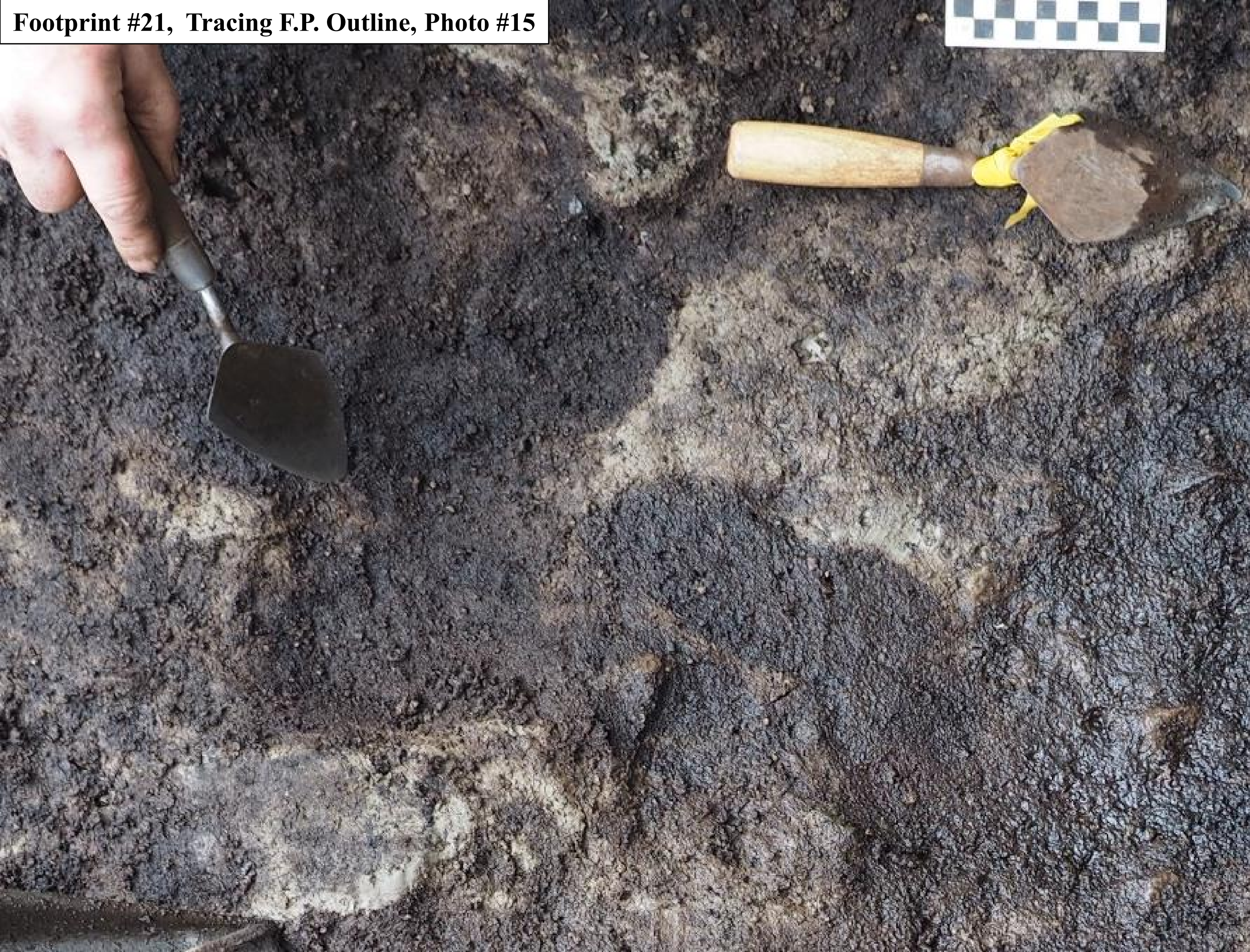

Footprint #21, Tracing F.P. Outline, Photo #16

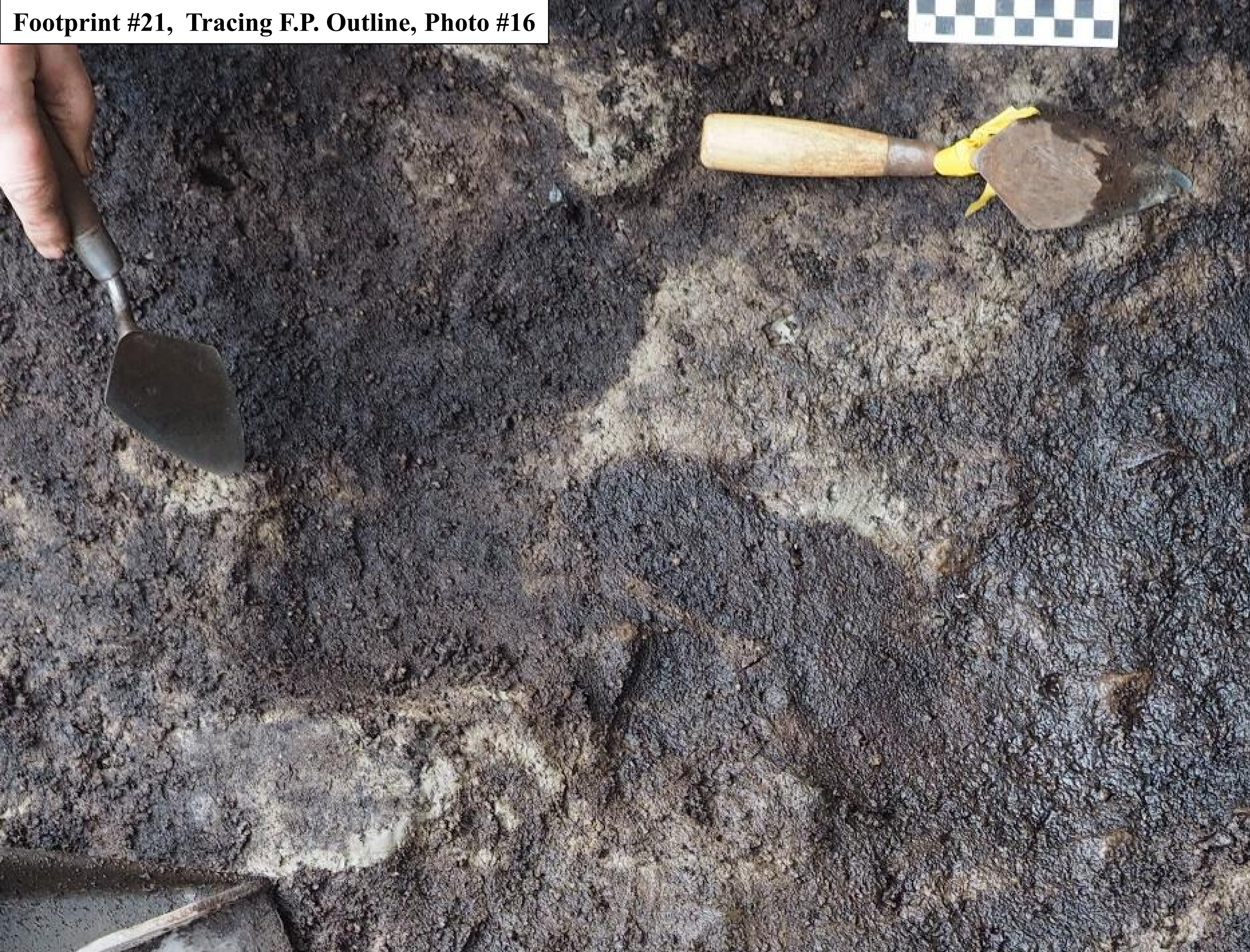

Footprint #21, Tracing F.P. Outline, Photo #17

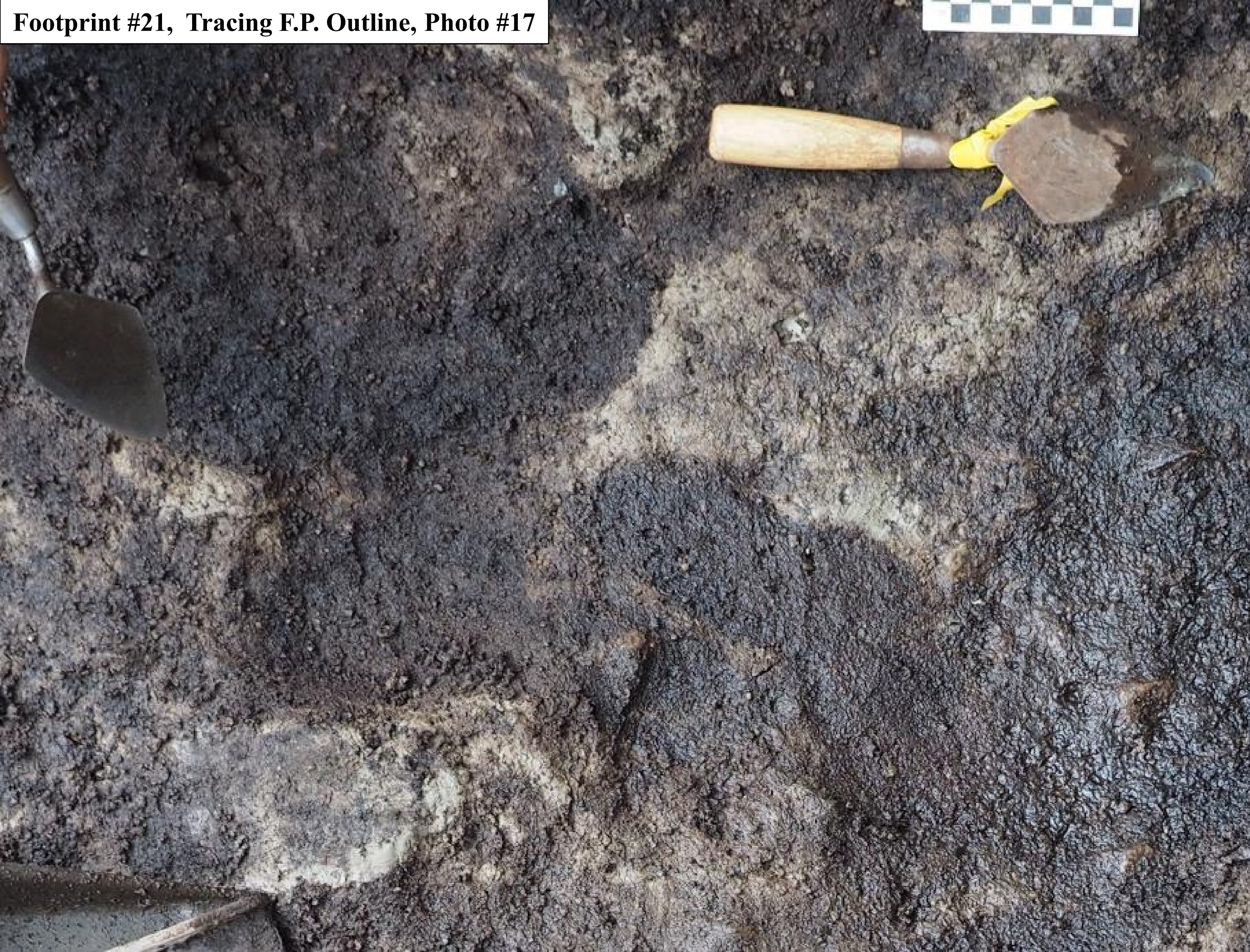

Footprint #7, Photo #1

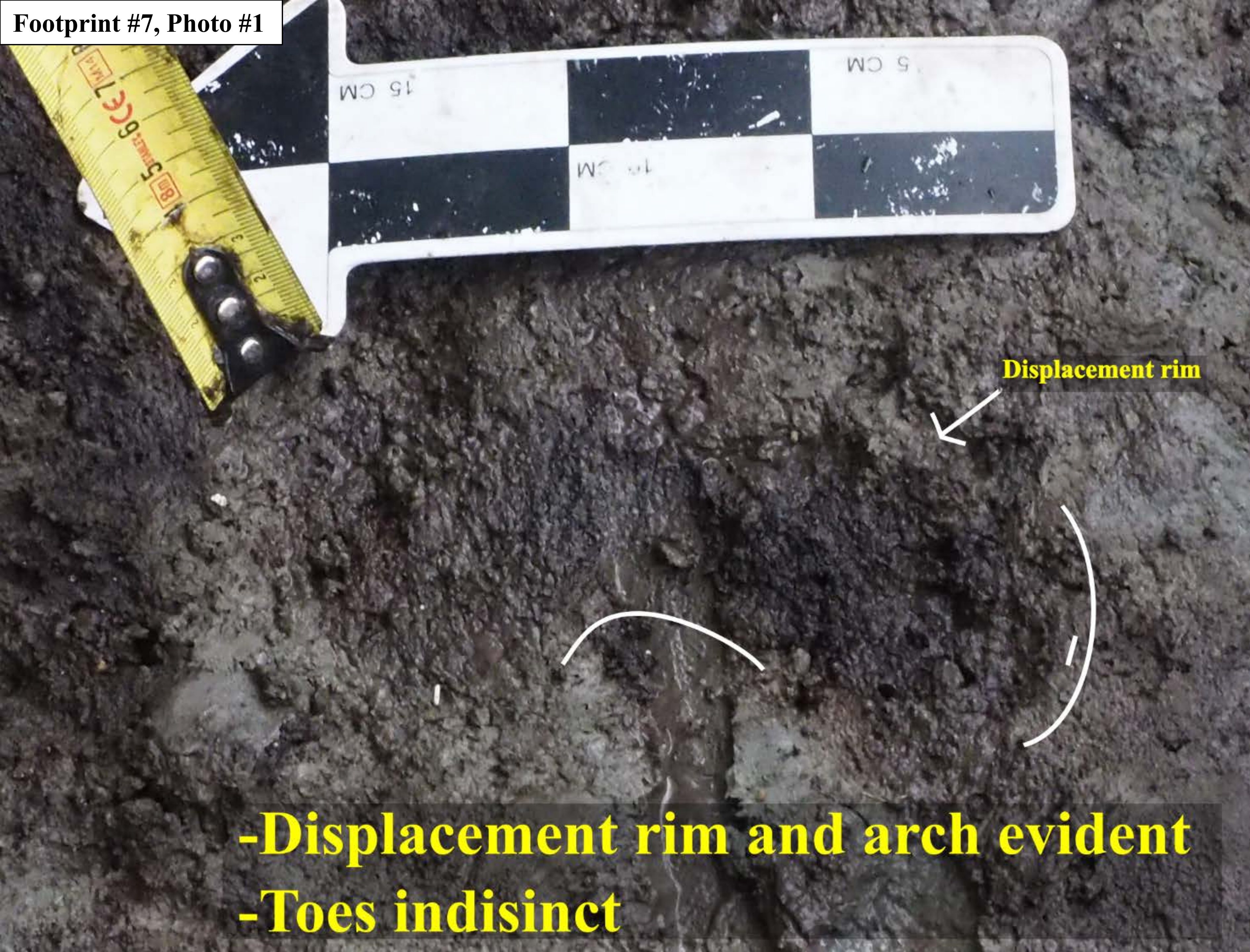

Footprint #7, Photo #2

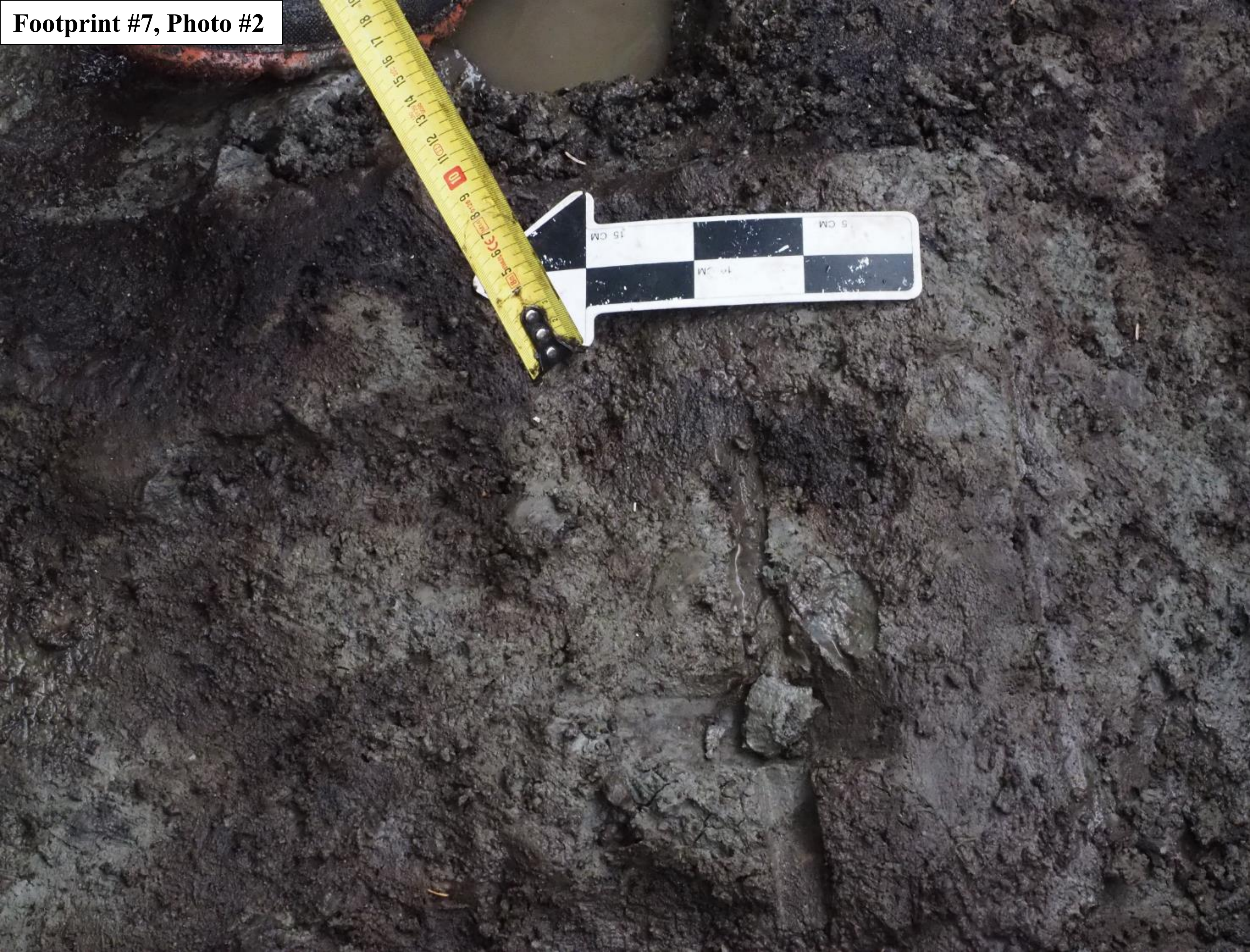

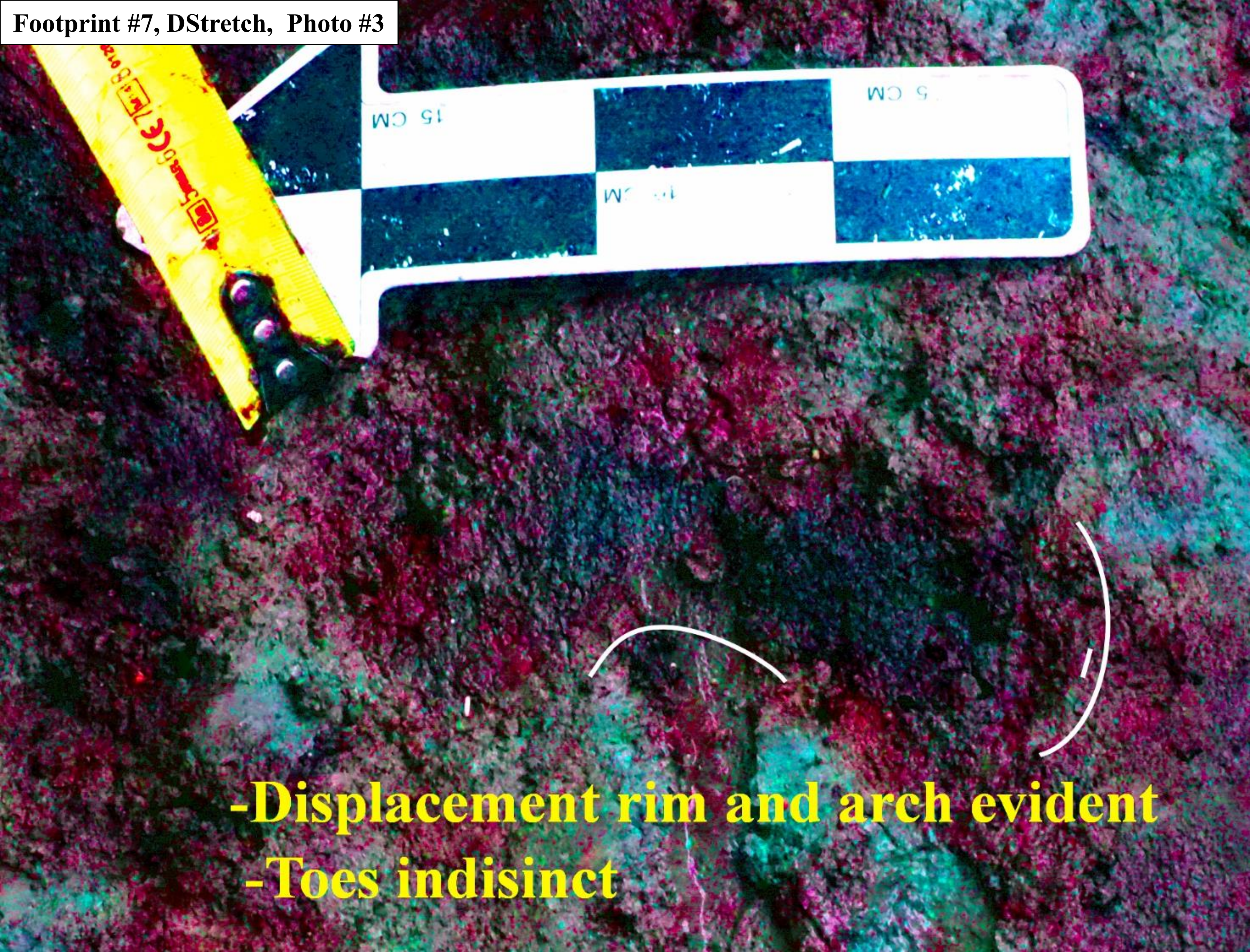

Footprint #7, DStretch, Photo #3

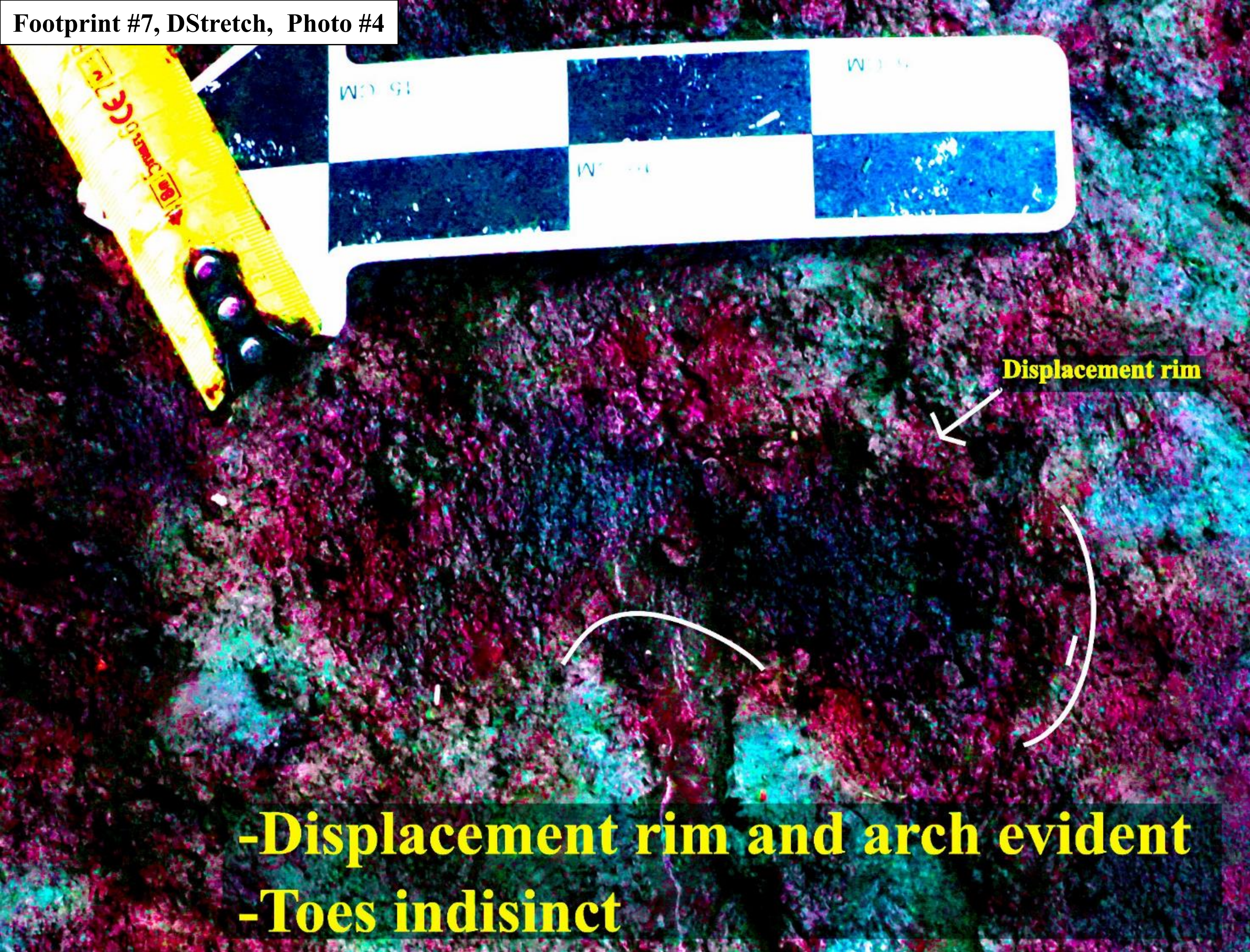
Footprint #7, DStretch, Photo #4
15 CM
Displacement rim
-Displacement rim and arch evident
-Toes indisinct

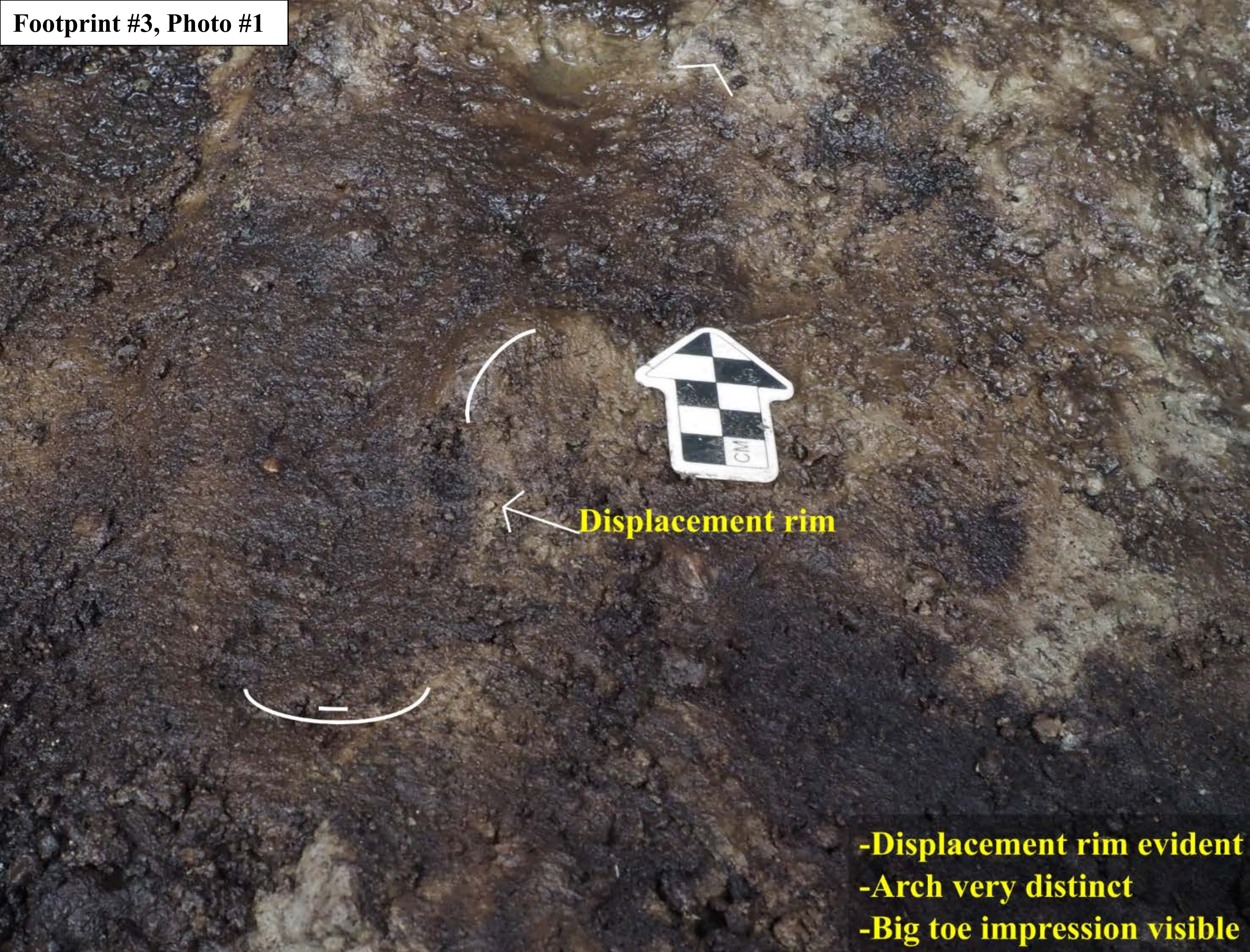
Footprint #3, Photo #1
CM
Displacement rim
-Displacement rim evident
-Arch very distinct
-Big toe impression visible

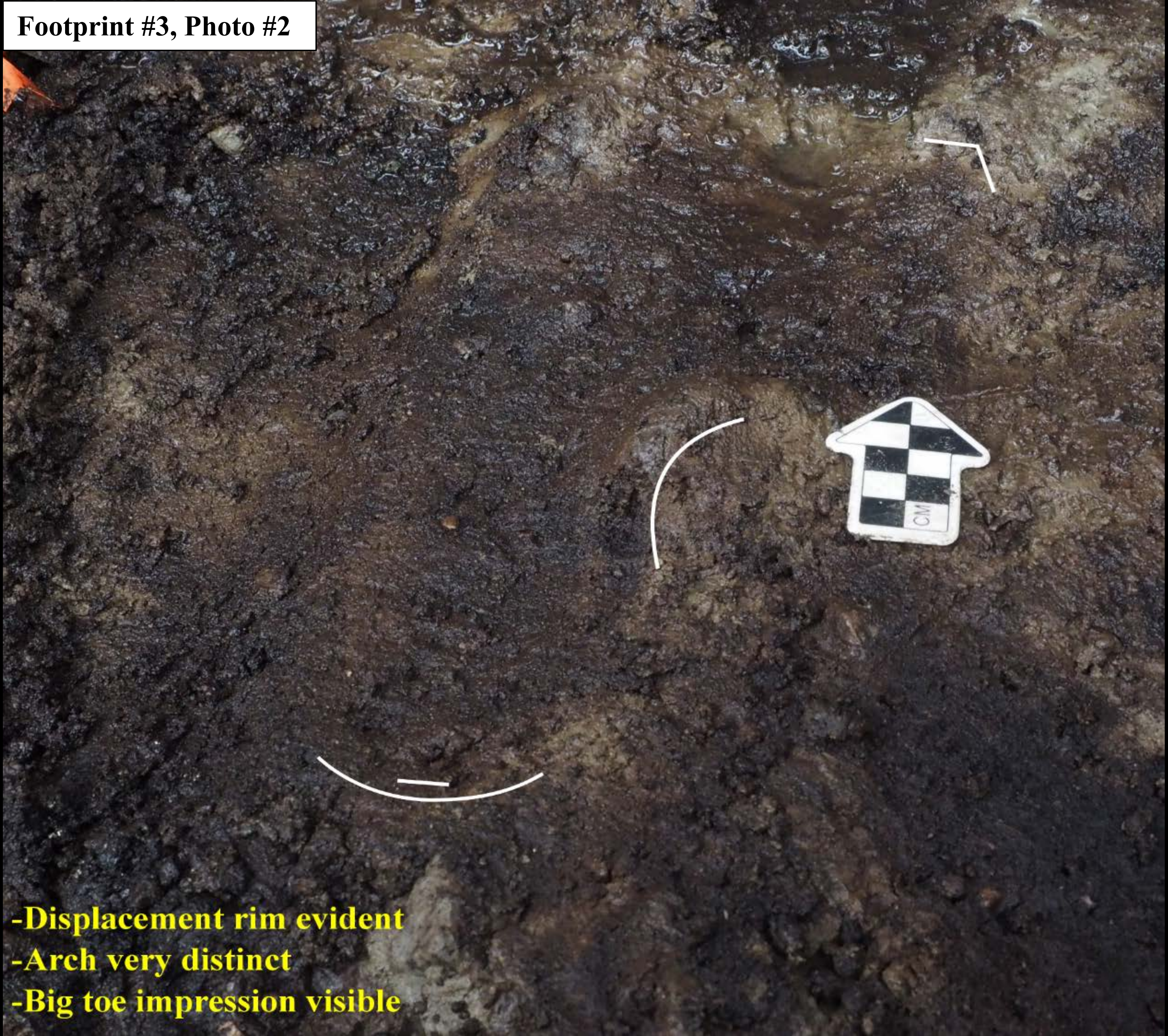
Footprint #3, Photo #2
-Displacement rim evident
-Arch very distinct
-Big toe impression visible

Footprint #3, Photo #3

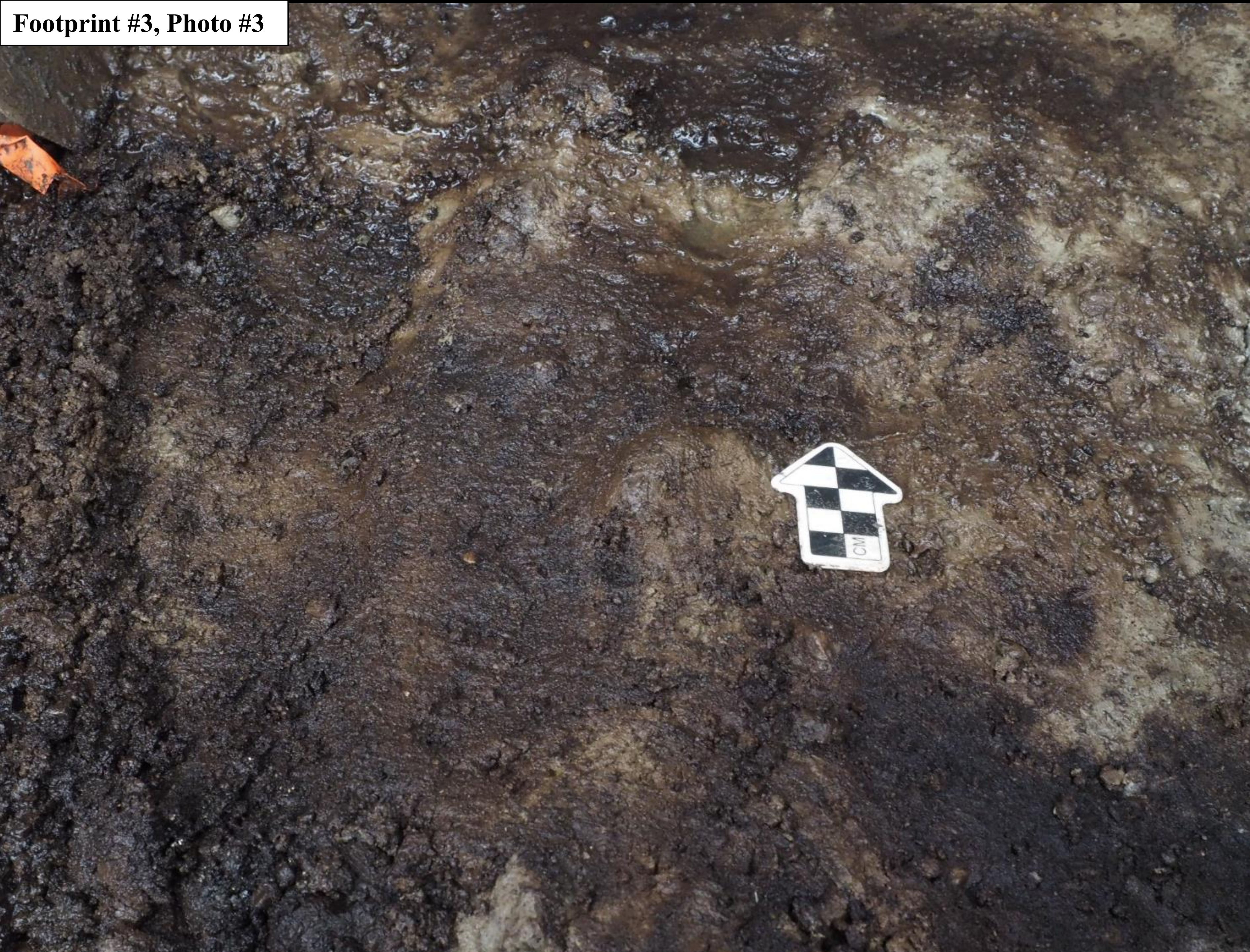

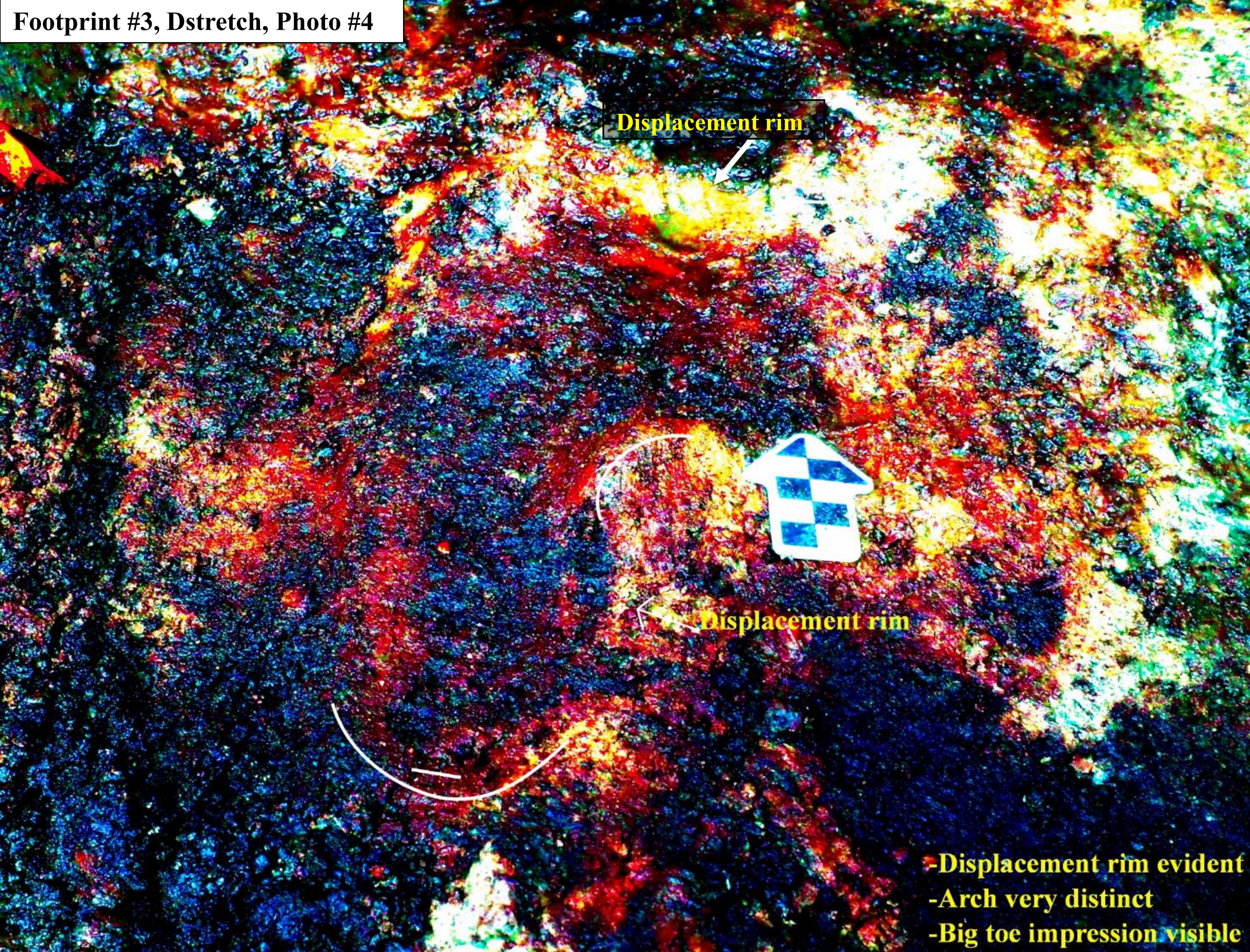
Footprint #3, Dstretch, Photo #4
Displacement rim
Displacement rim
-Displacement rim evident
-Arch very distinct
-Big toe impression visible

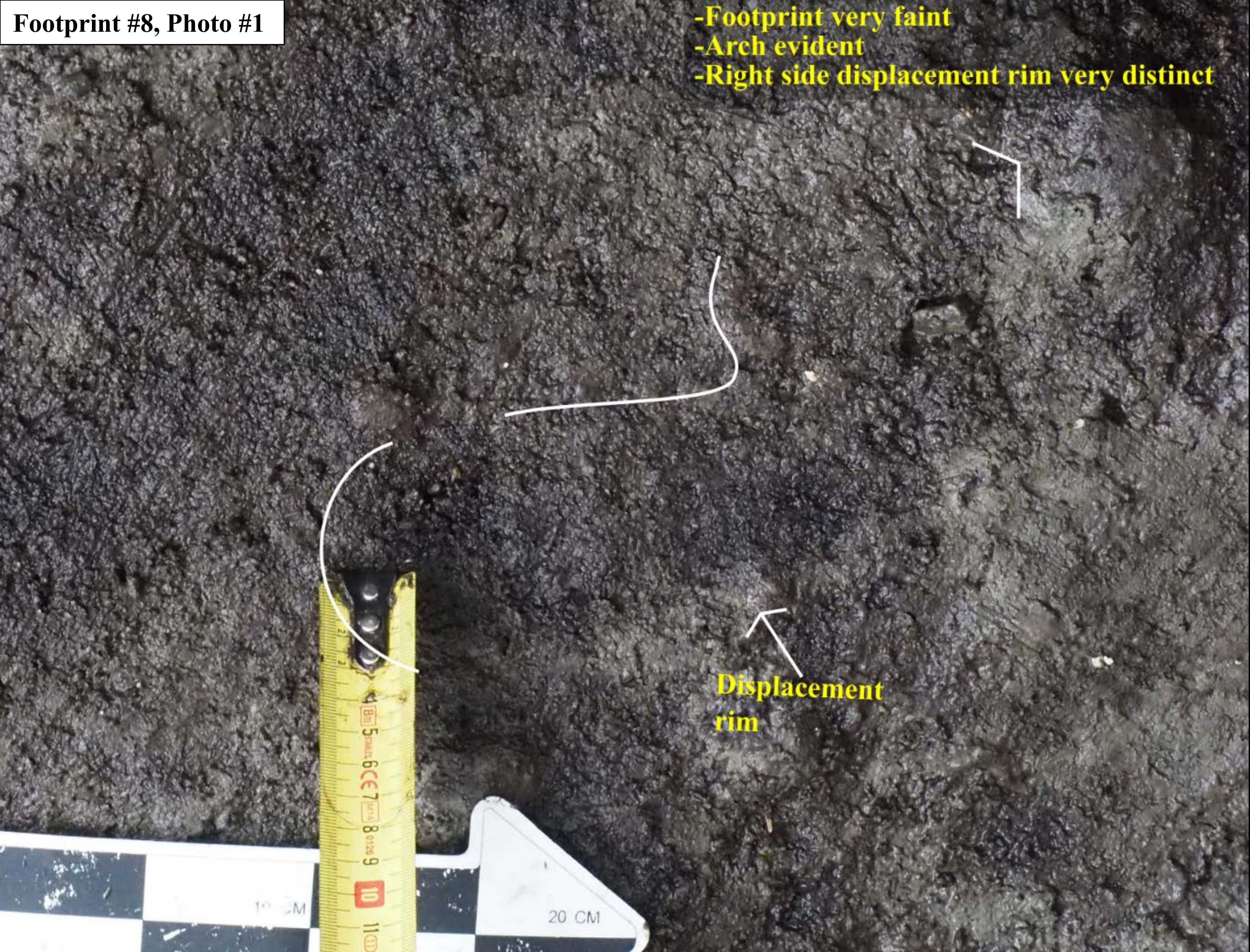
Footprint #8, Photo #1
-Footprint very faint
-Arch evident
-Right side displacement rim very distinct
Displacement rim
20 CM

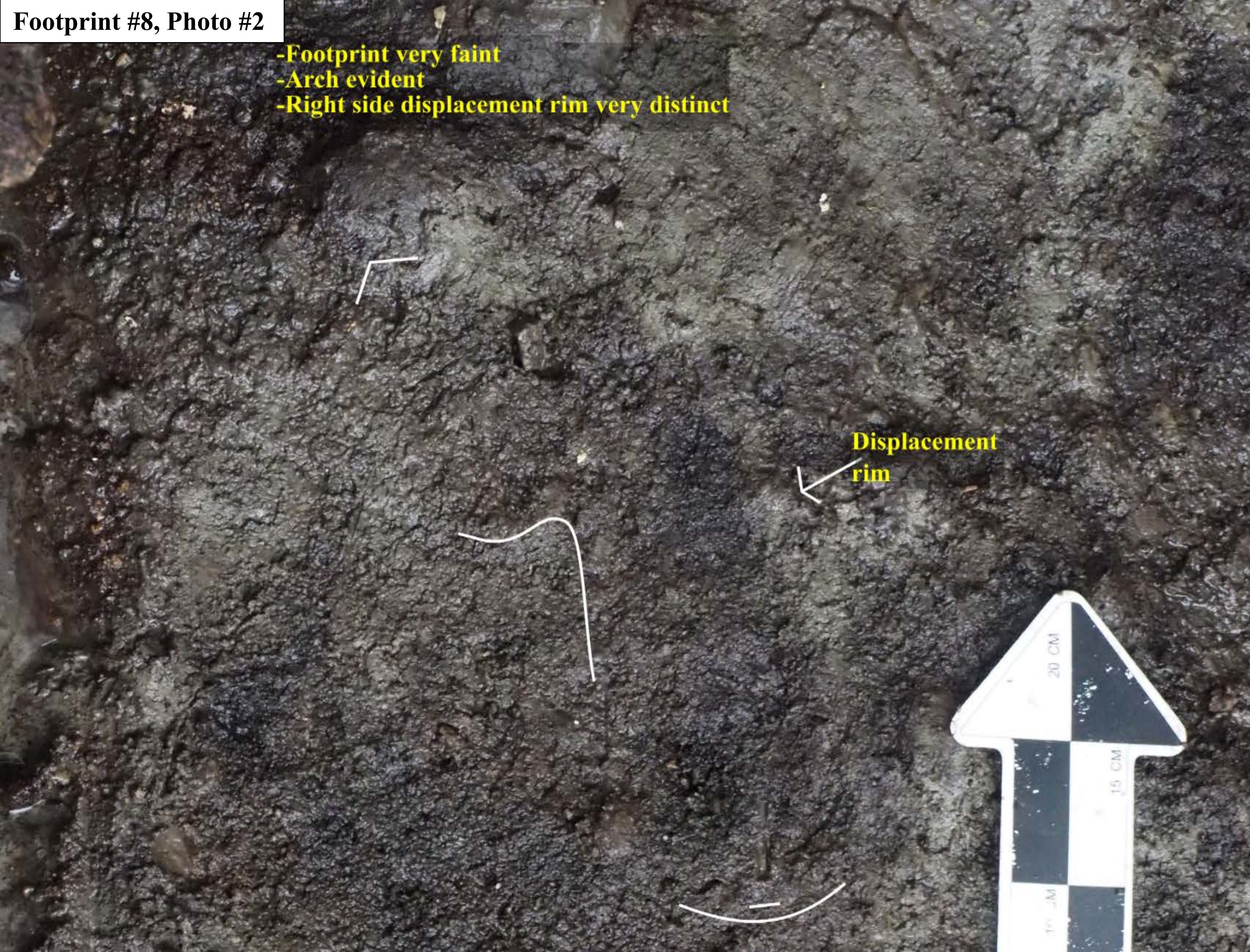
Footprint #8, Photo #2
-Footprint very faint
-Arch evident
-Right side displacement rim very distinct
Displacement rim
20 CM
15 CM

Footprint #8, Photo #3

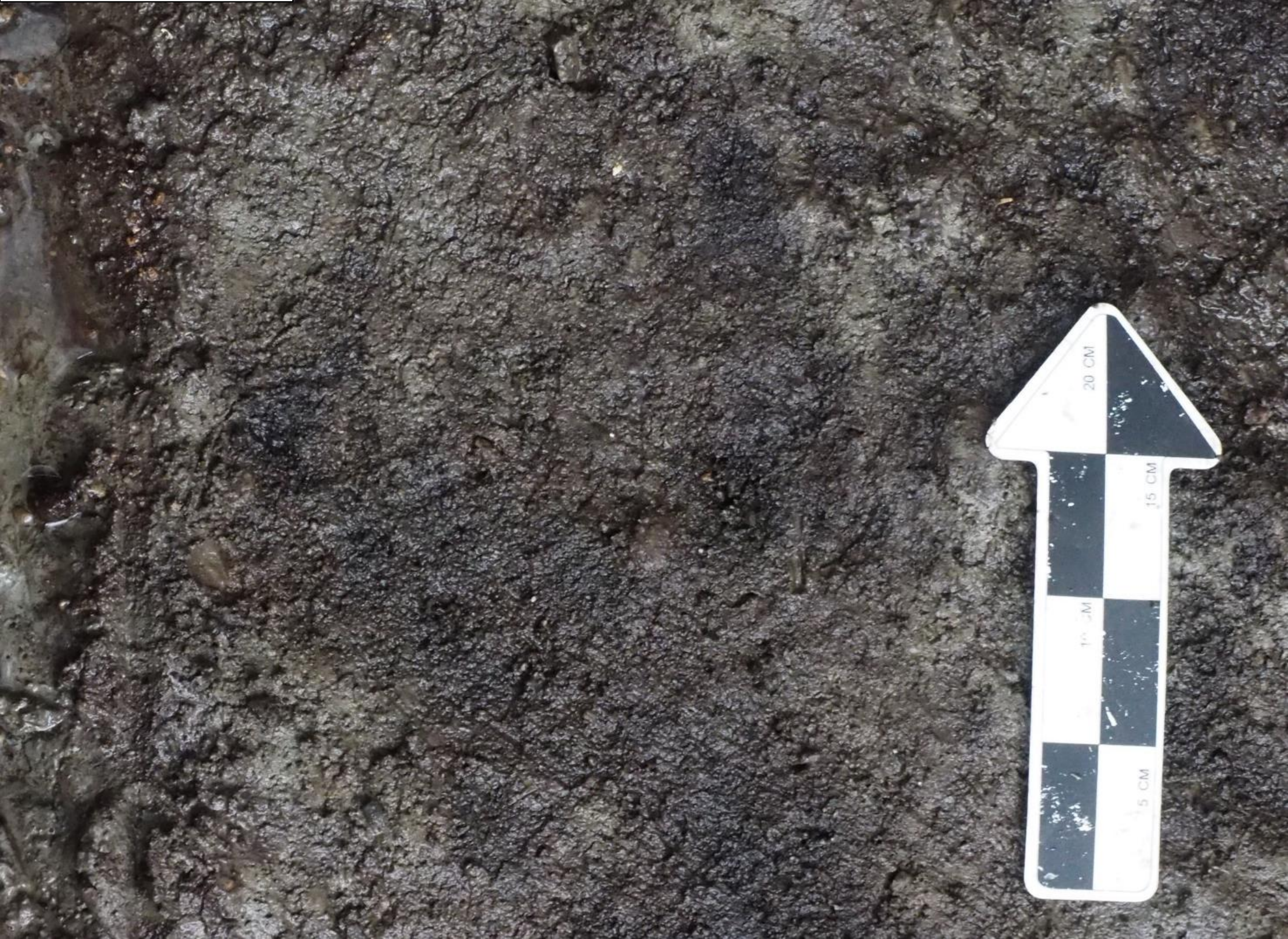

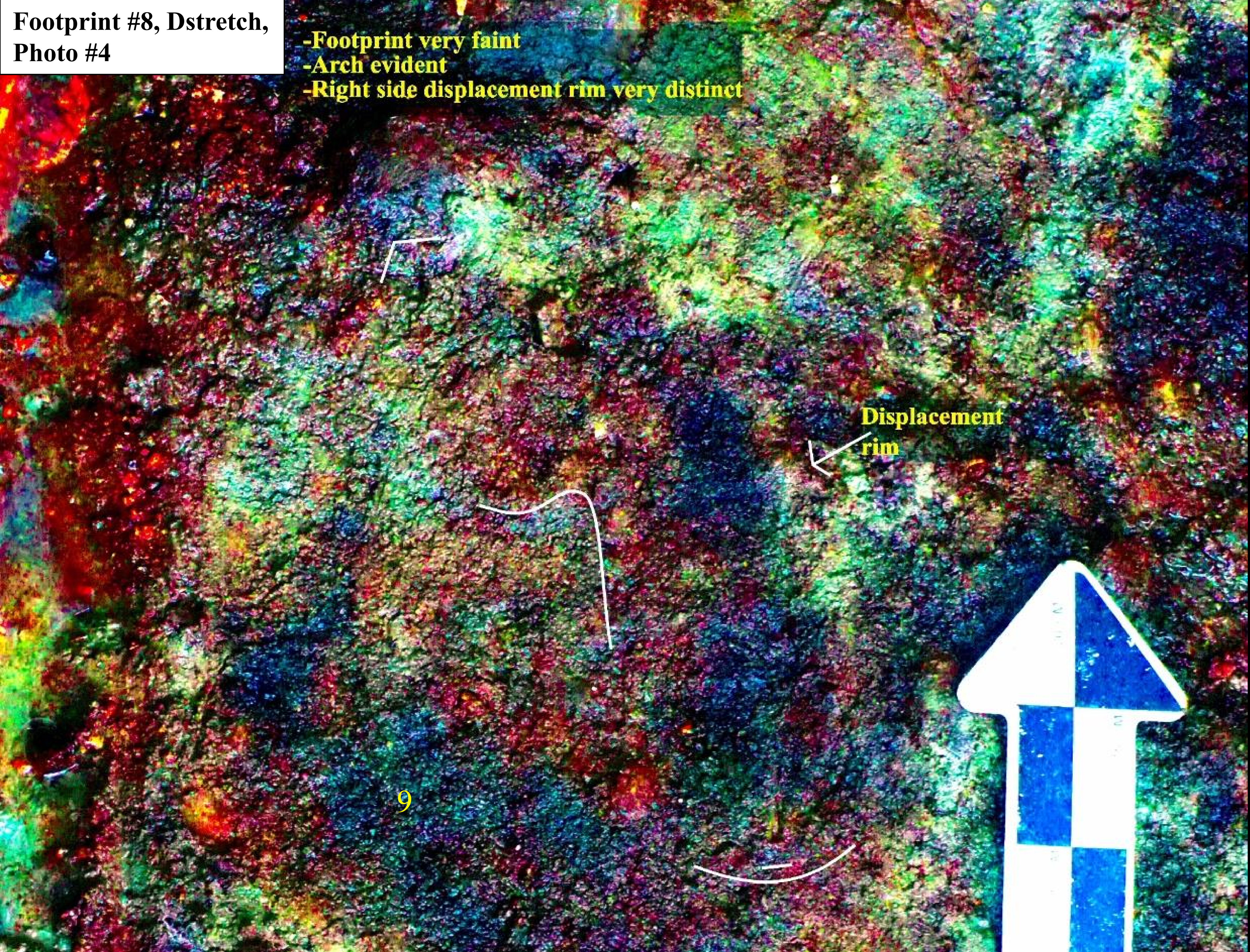

Footprint #8, Dstretch, Photo #4

Footprint #8, Footprint #9 too faint to photograph well, Photo #5

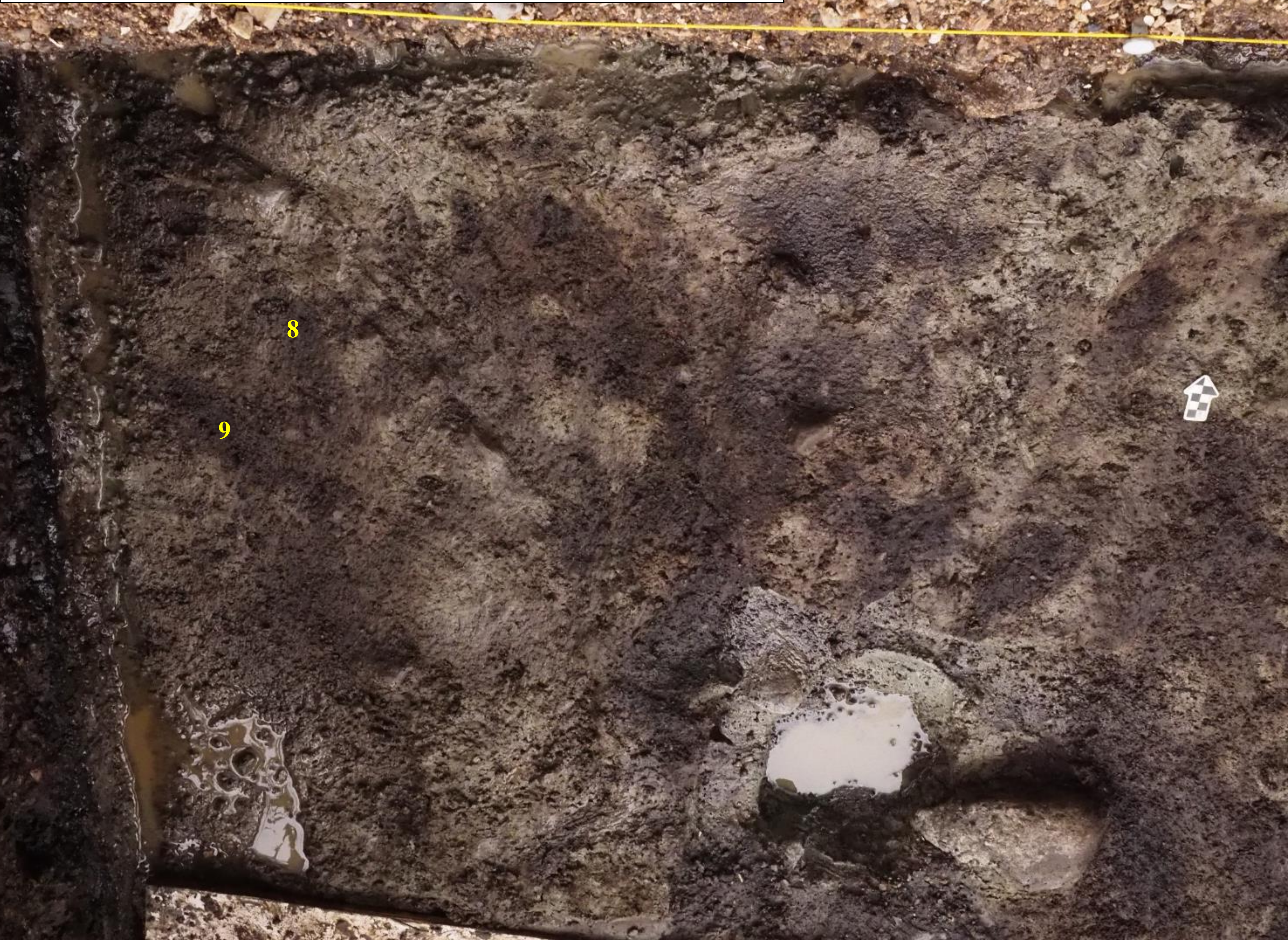

Footprint #8, Footprint #9 too faint to photograph well,-DStretch, Photo #6

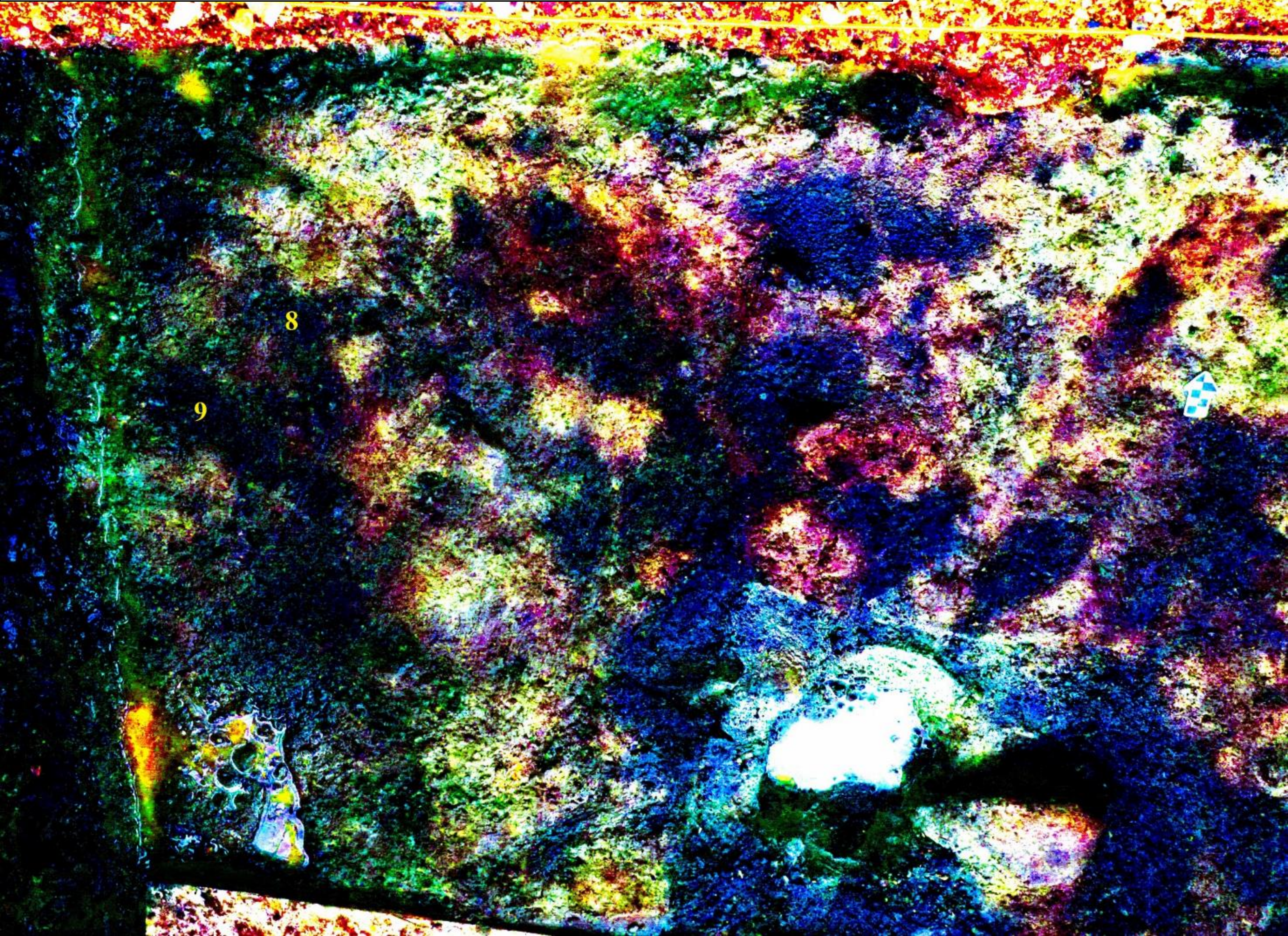

Footprint #10 & 11, Photo #1

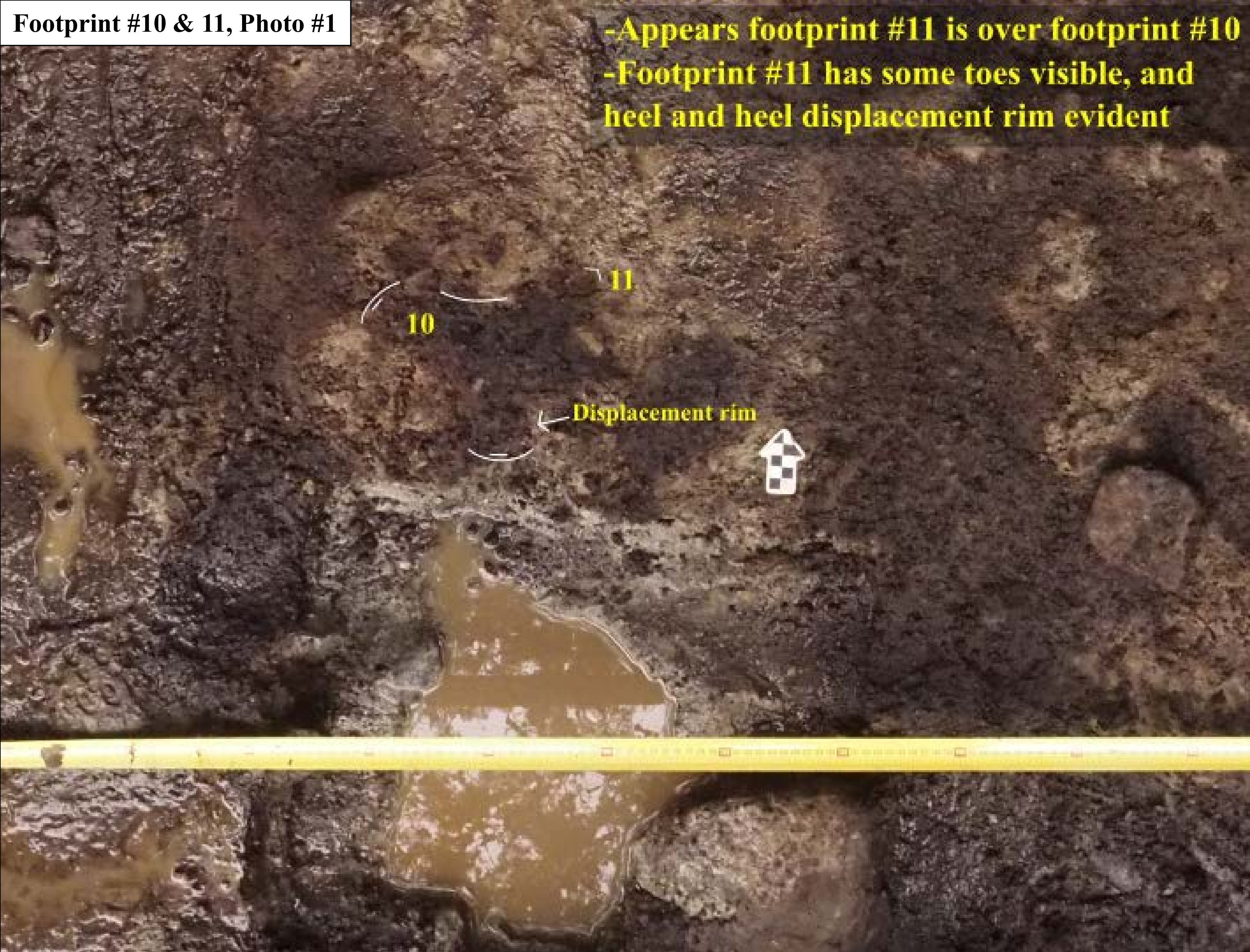

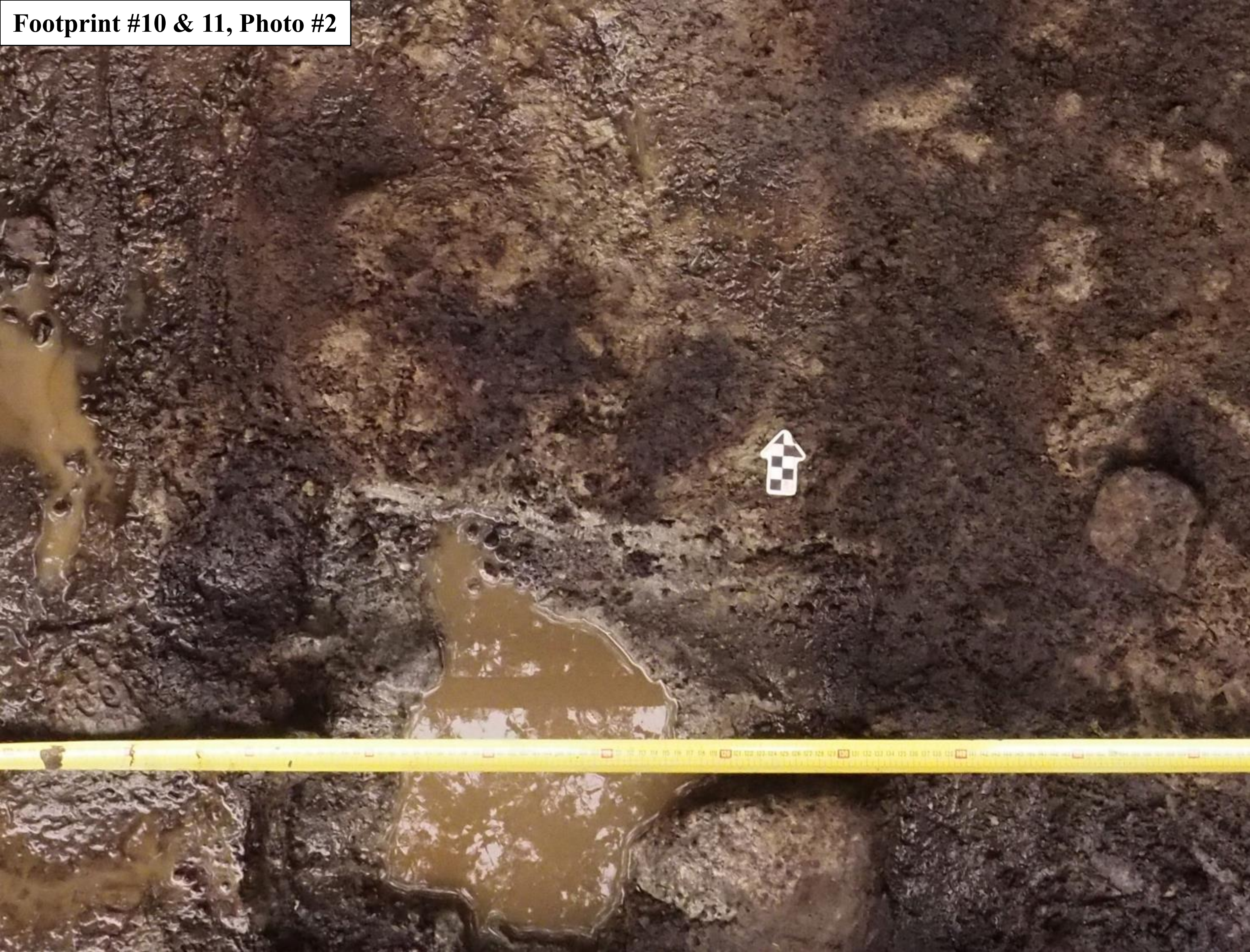

Footprint #10 & 11, Photo #2

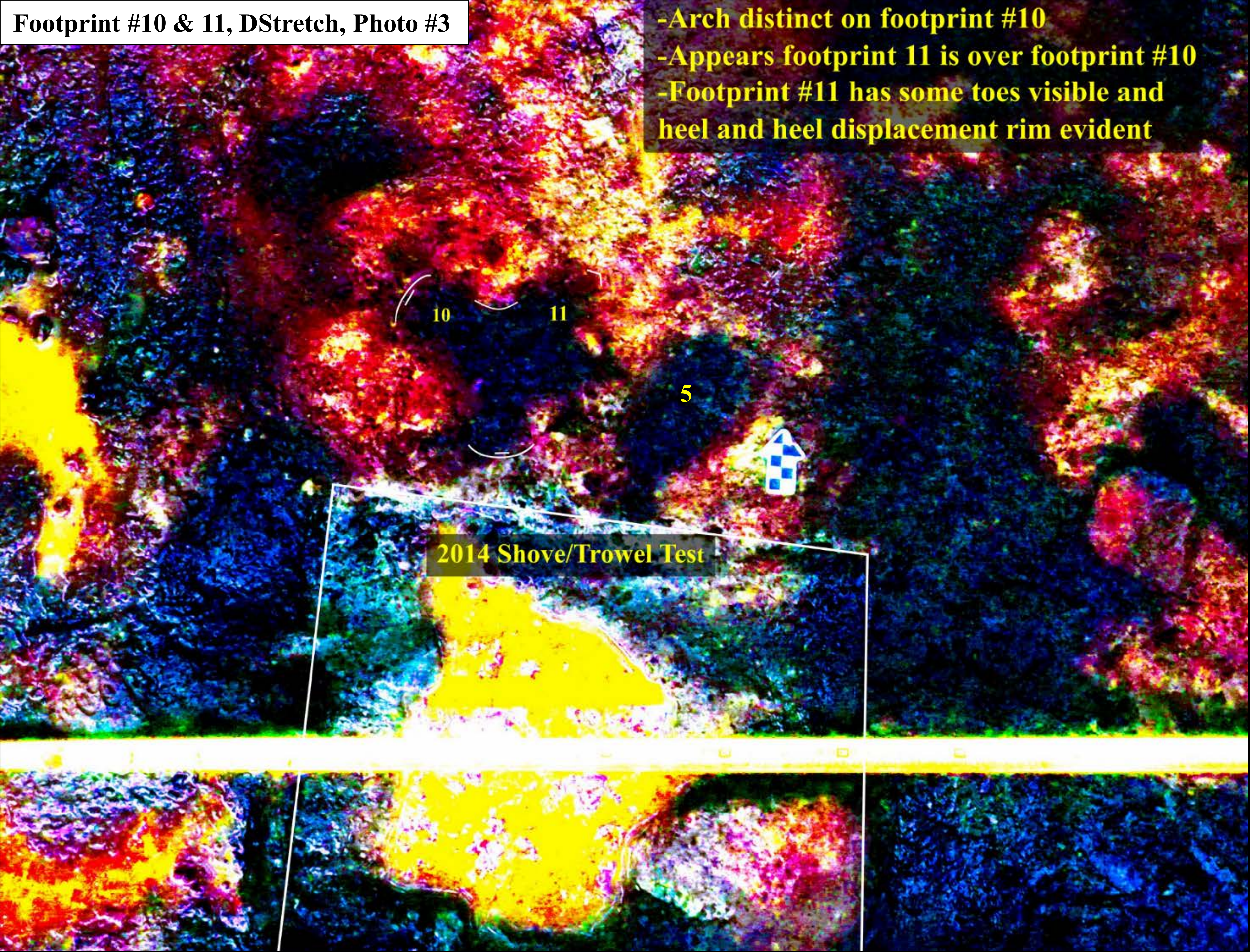
Footprint #10 & 11, DStretch, Photo #3
-Arch distinct on footprint #10
-Appears footprint 11 is over footprint #10
-Footprint #11 has some toes visible and
heel and heel displacement rim evident
10
11
5
2014 Shove/Trowel Test

Footprint #10 & 11, DStretch, Photo #4

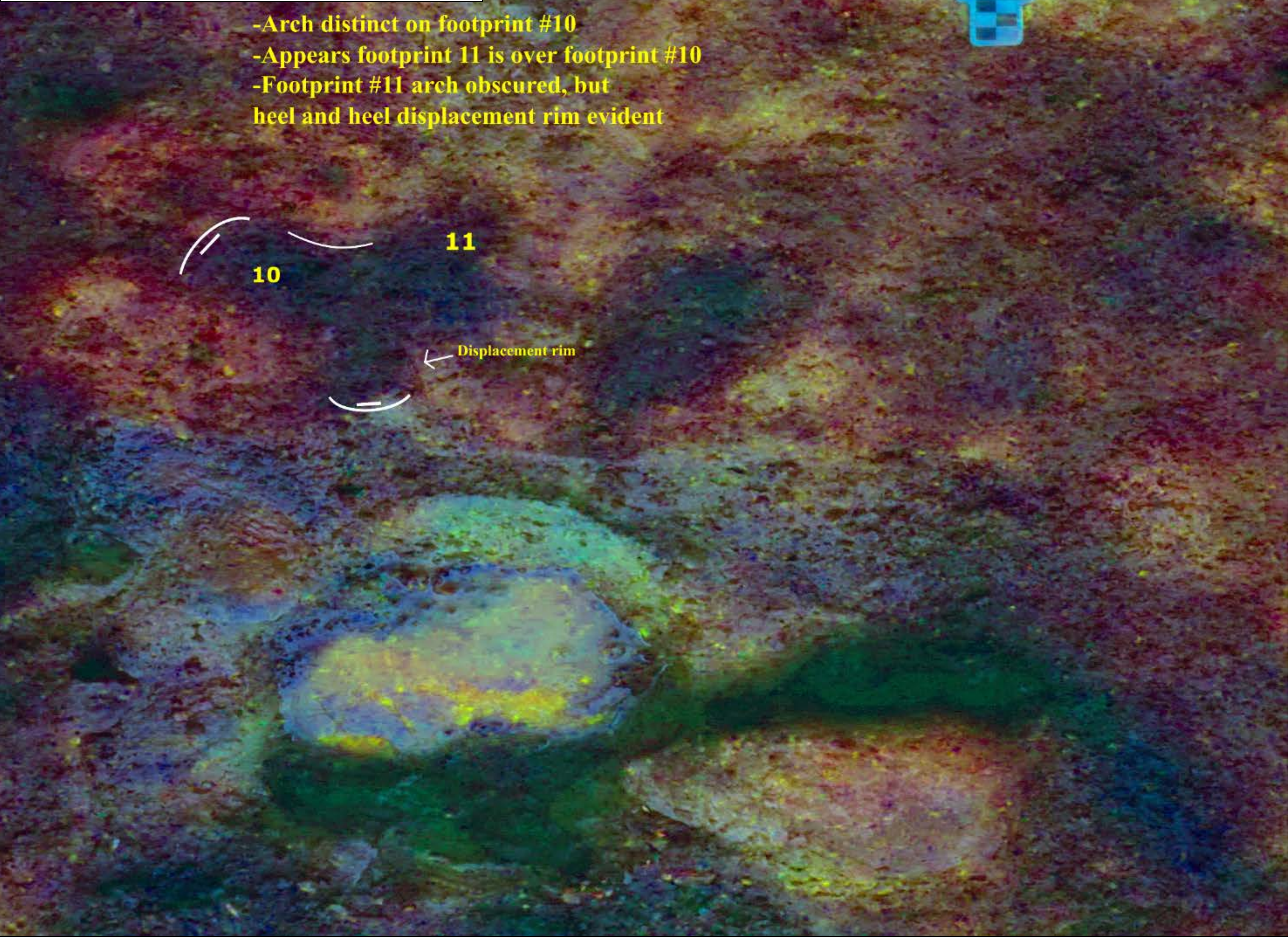

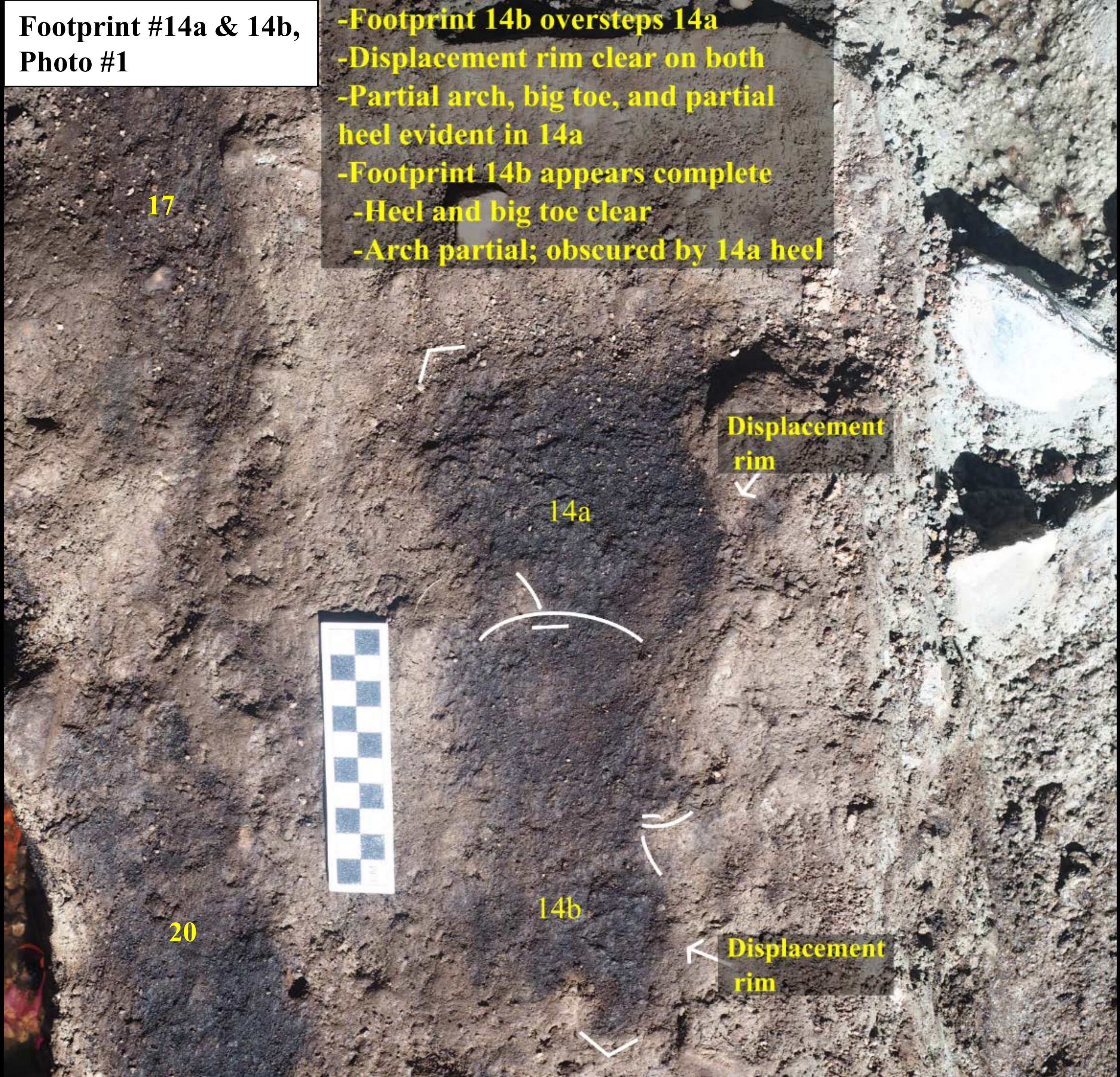
Footprint #14a & 14b,
Photo #1
-Footprint 14b oversteps 14a
-Displacement rim clear on both
-Partial arch, big toe, and partial heel evident in 14a
-Footprint 14b appears complete
-Heel and big toe clear
-Arch partial; obscured by 14a heel
17
Displacement rim
14a
14b
20
Displacement rim

Footprint #14a & 14b, Photo #2

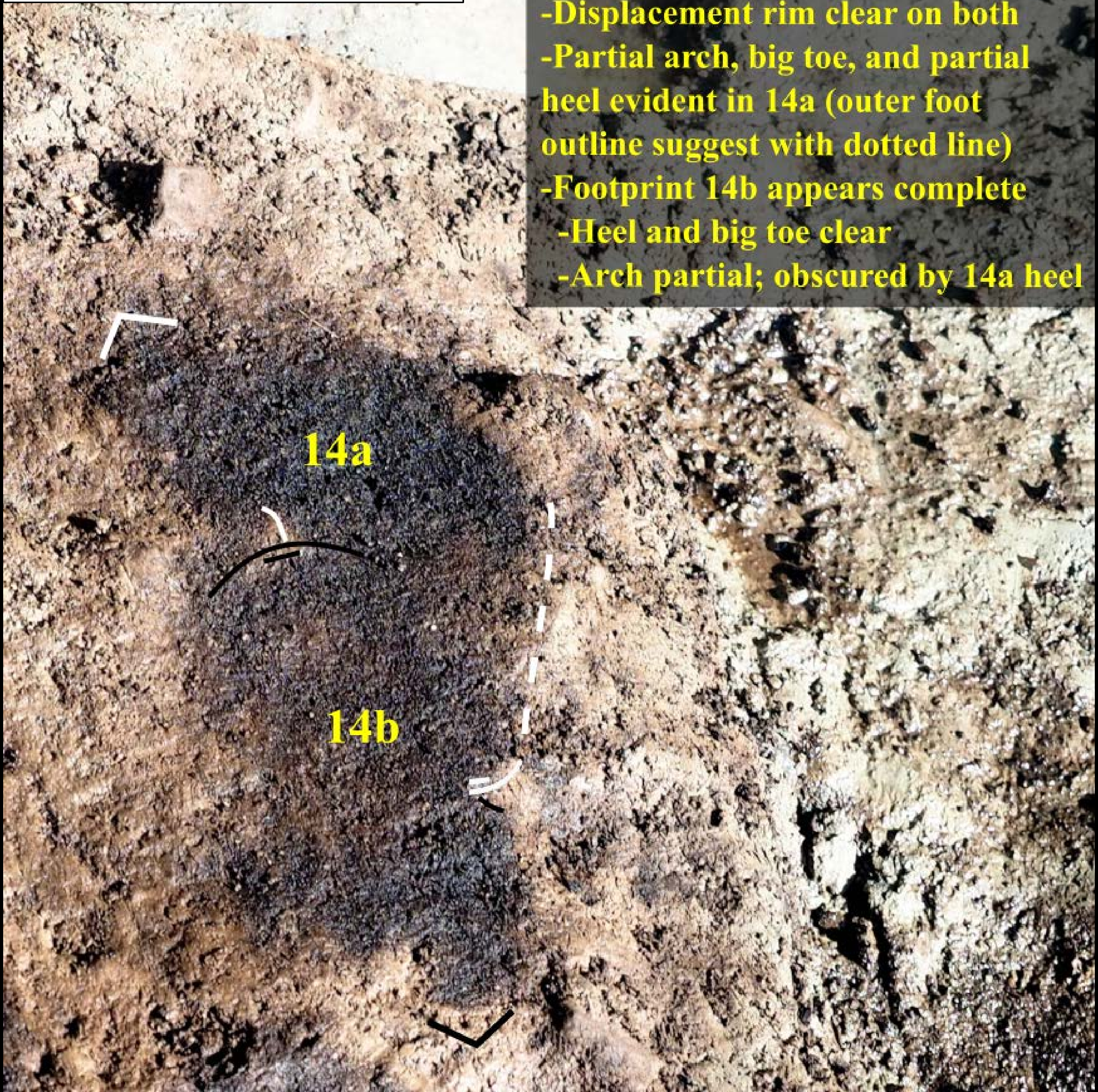

Footprint #14a & 14b, Photo #3

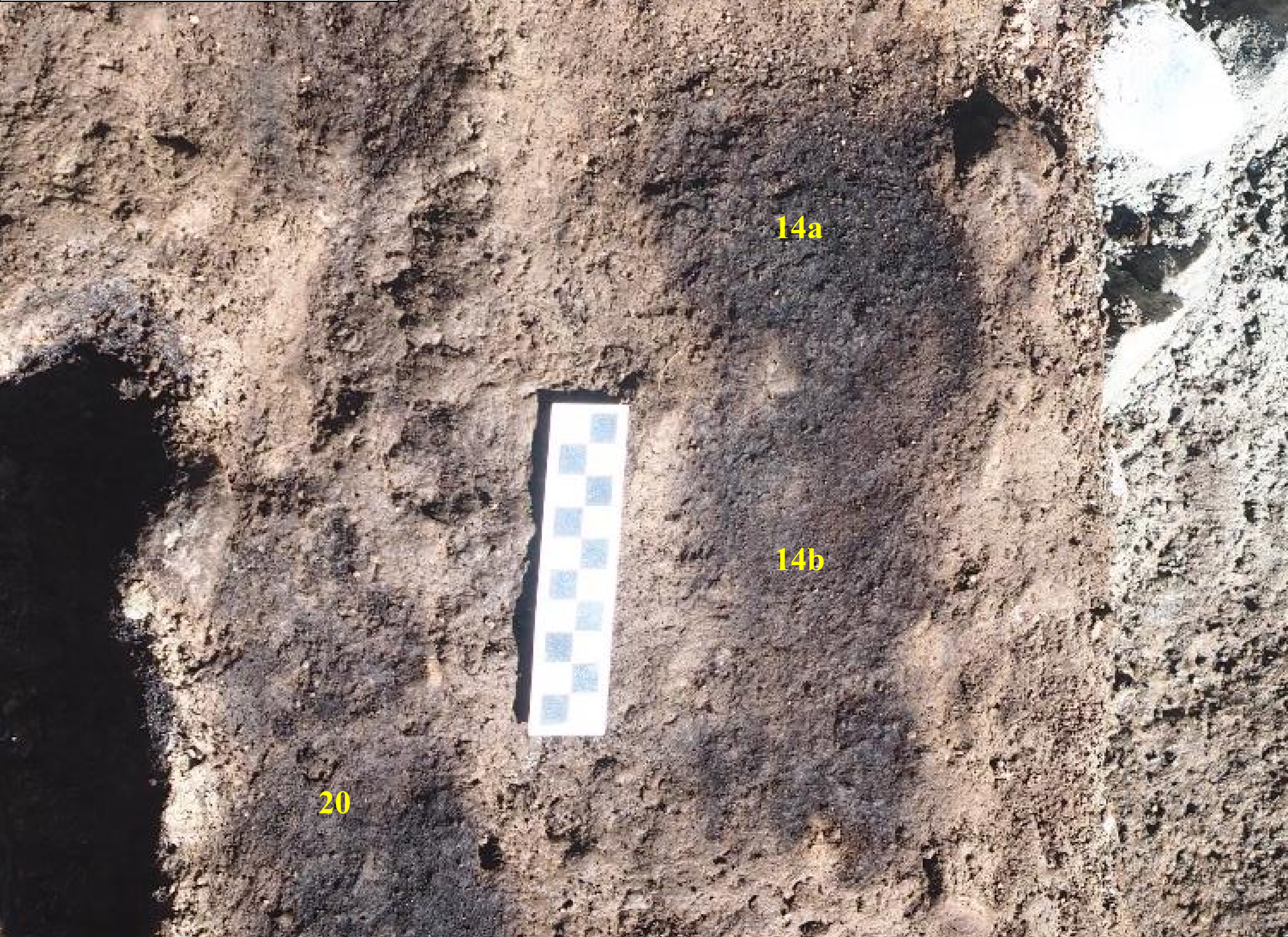

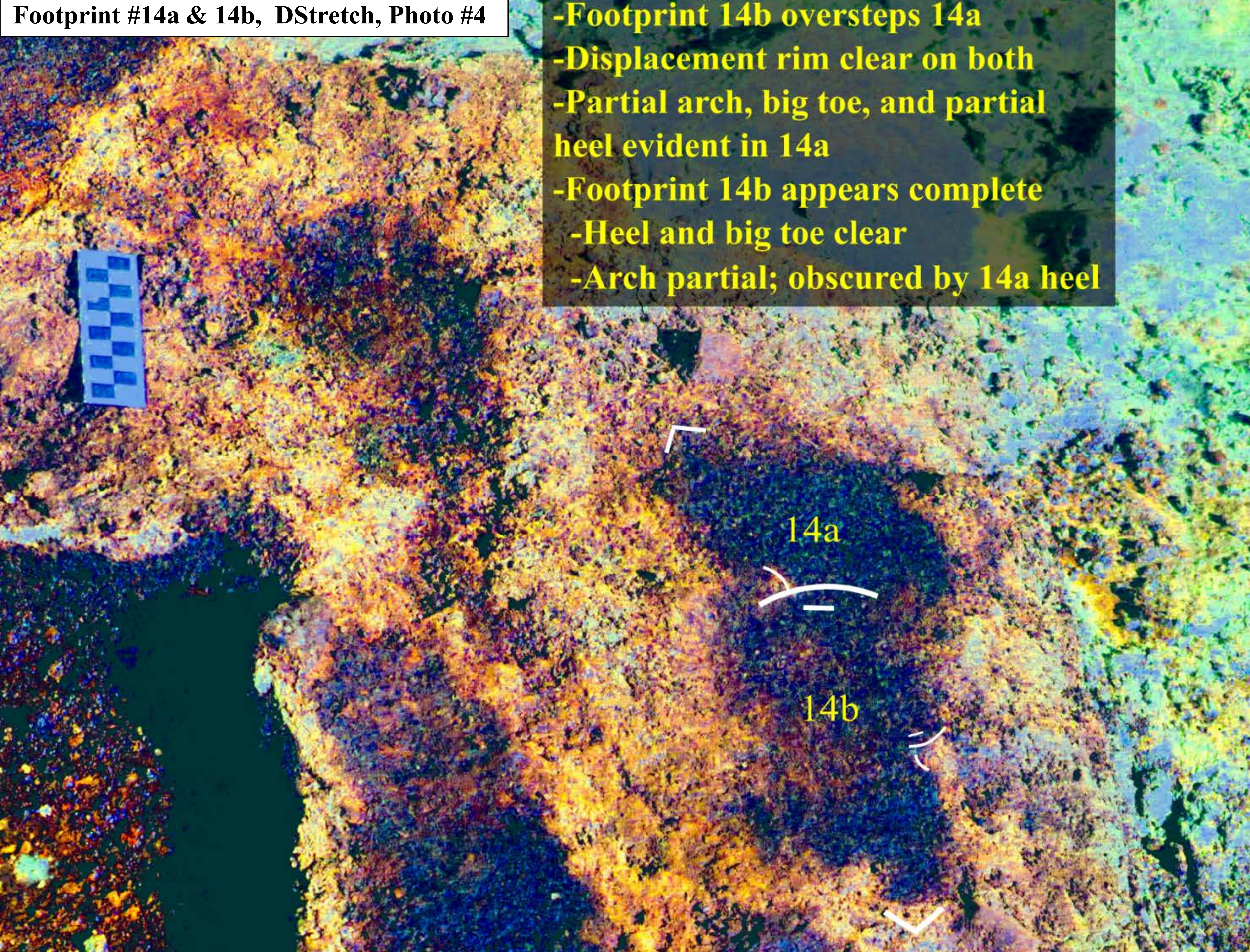
Footprint #14a & 14b, DStretch, Photo #4
-Footprint 14b oversteps 14a
-Displacement rim clear on both
-Partial arch, big toe, and partial heel evident in 14a
-Footprint 14b appears complete
-Heel and big toe clear
-Arch partial; obscured by 14a heel
14a
14b

Footprint #14a & 14b, DStretch, Photo #5

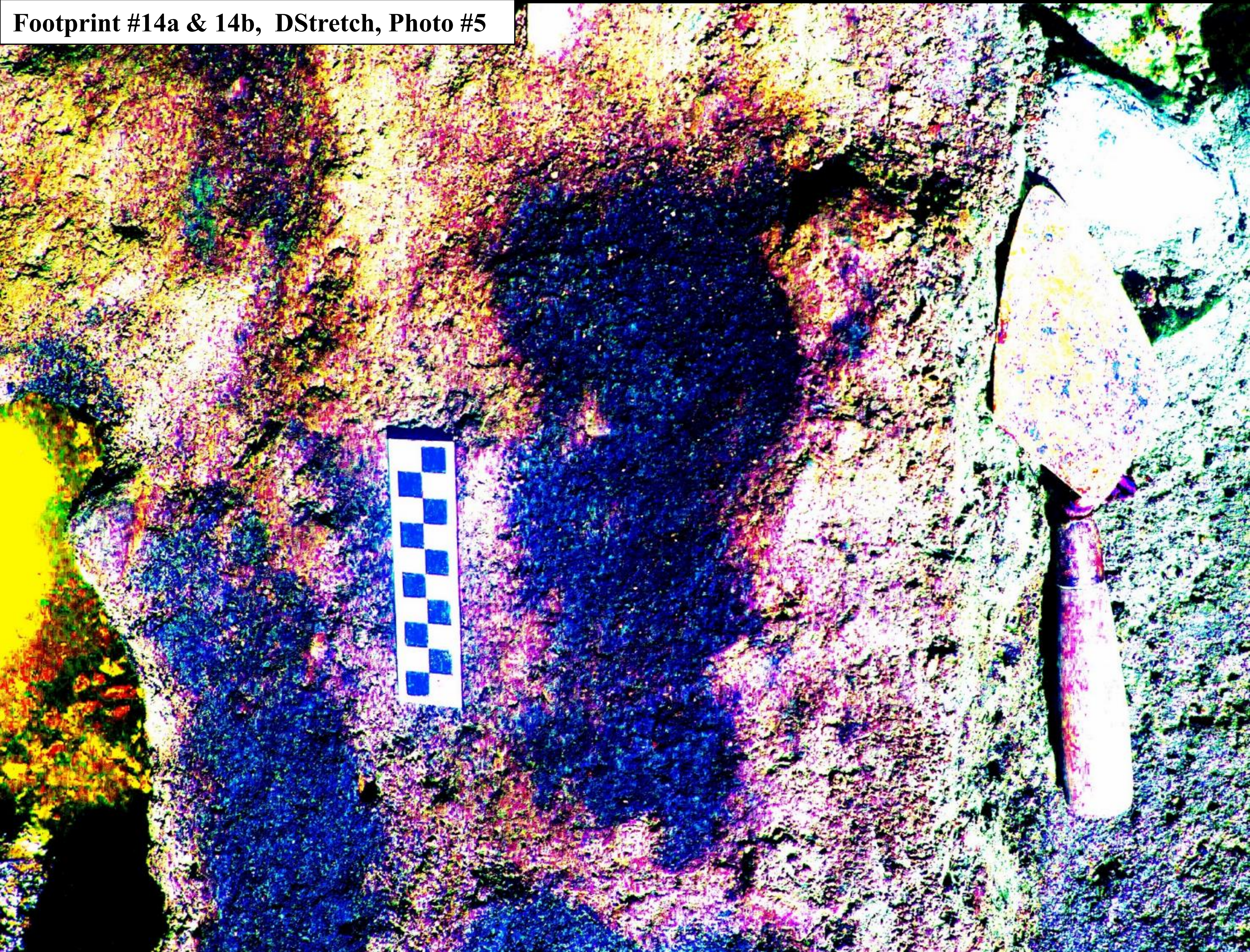

Footprint #16, Photo #1

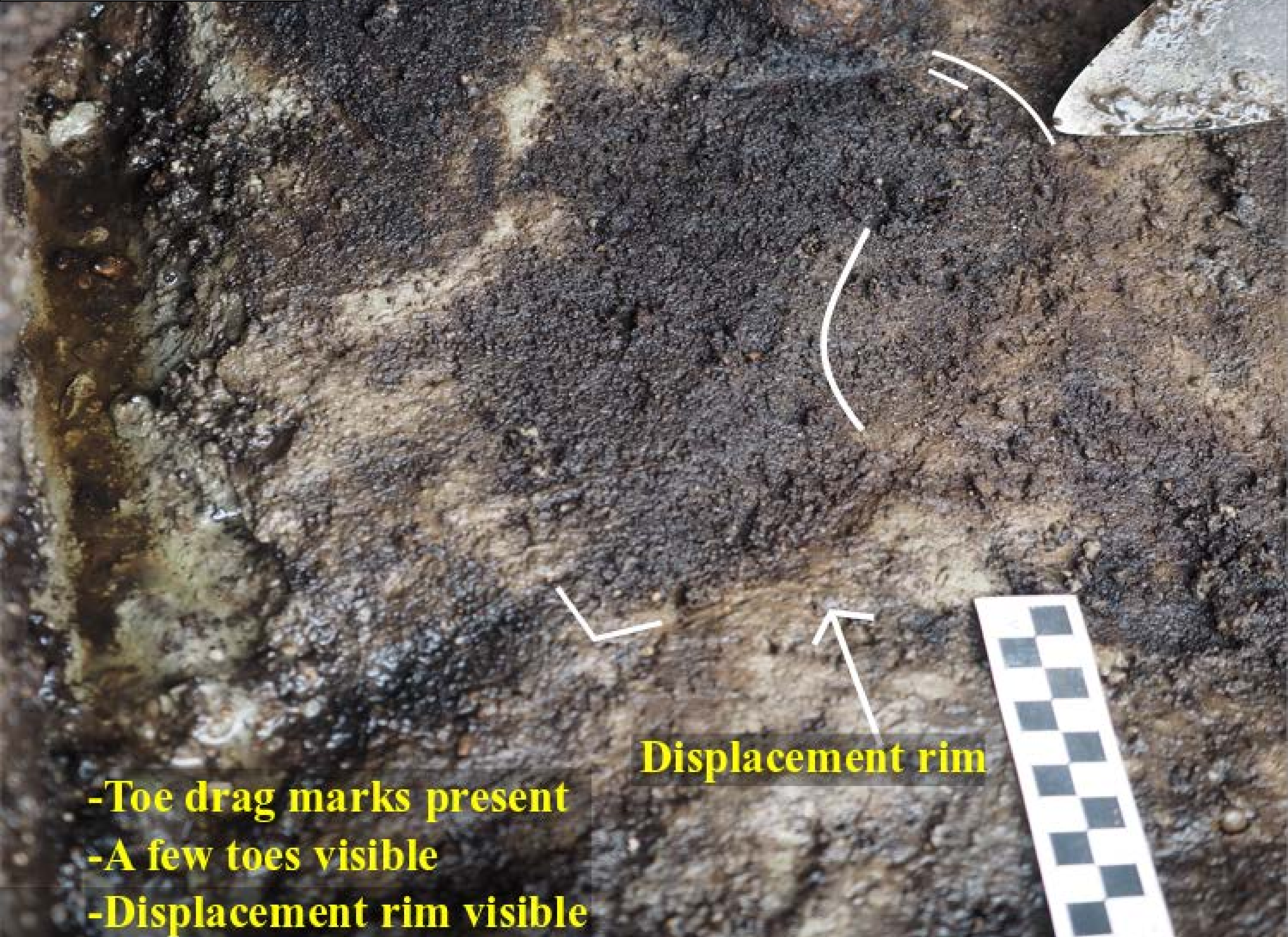

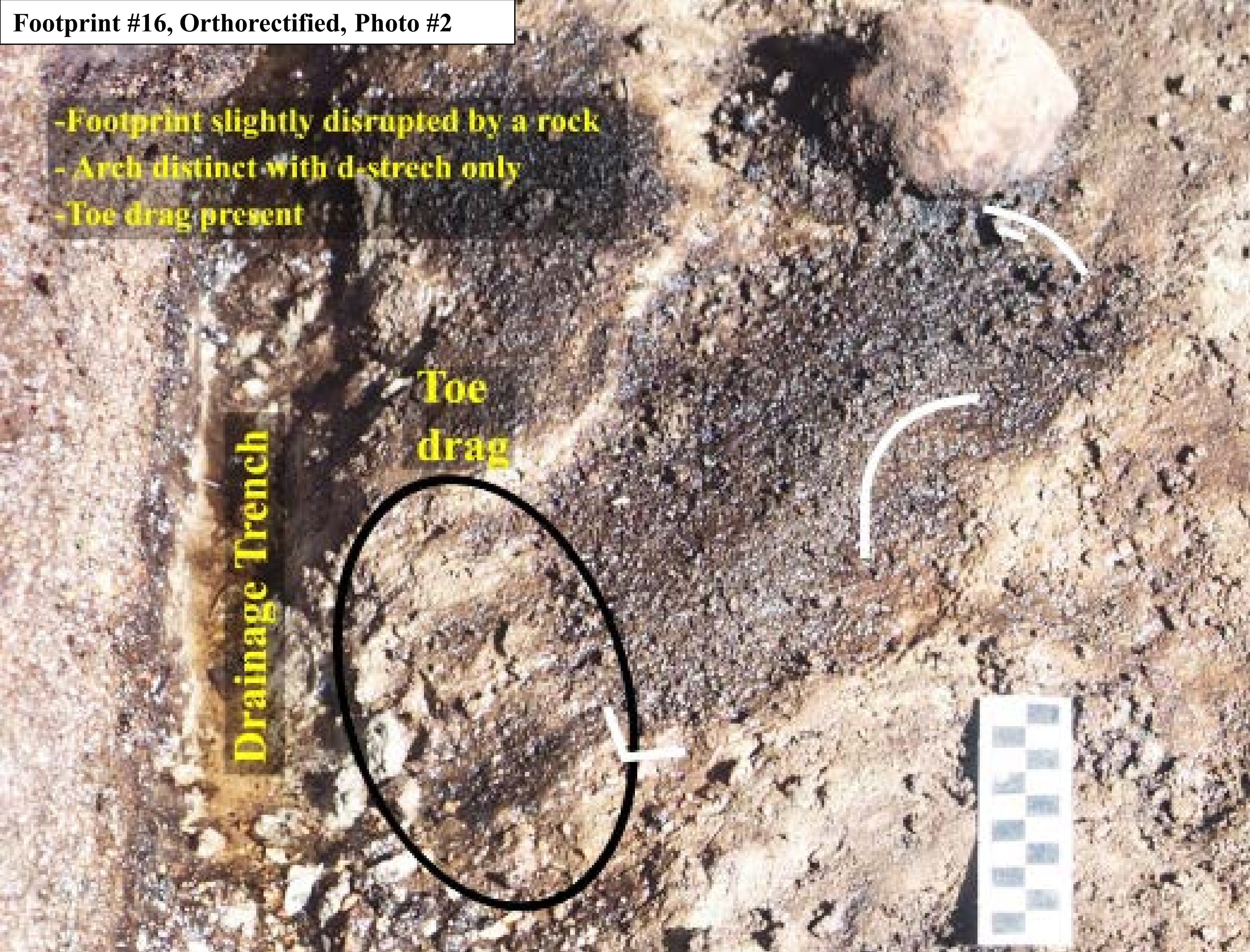

Footprint #16, Orthorectified, Photo #2

Footprint #16, Orthorectified, Photo #3

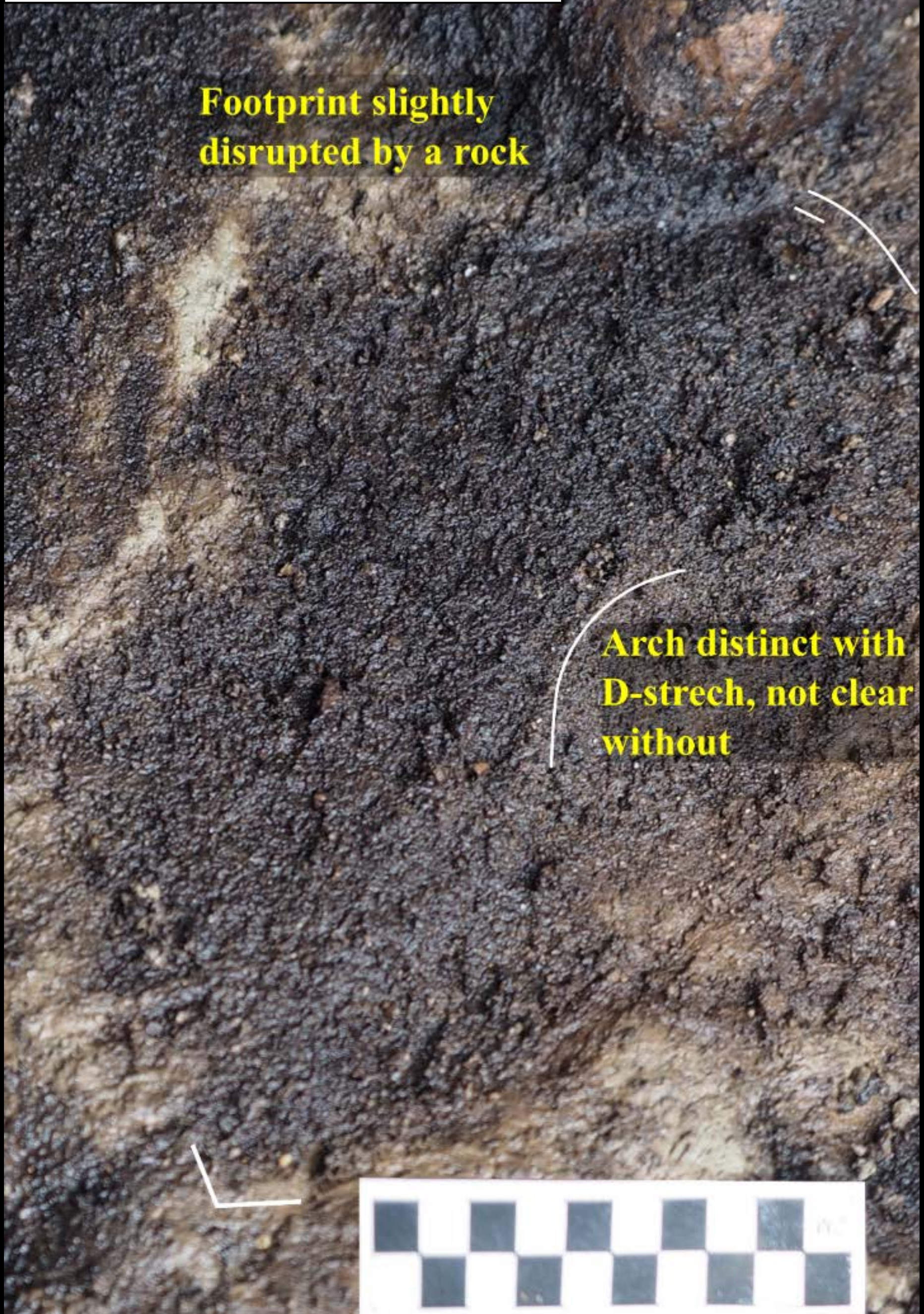

Footprint #16, Orthorectified, DStretch, Photo #4

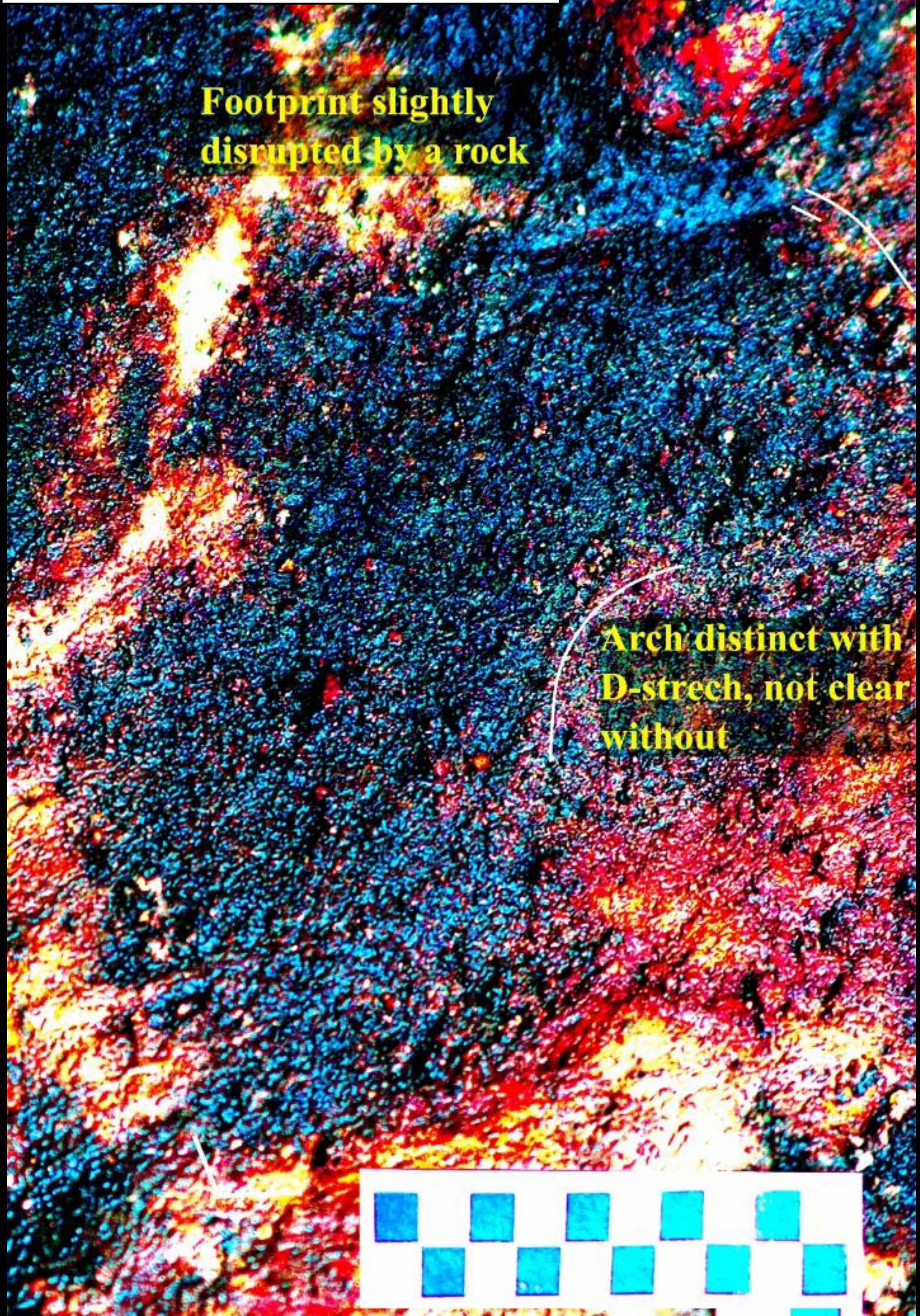

Footprint #16, Orthorectified, DStretch, Photo #5

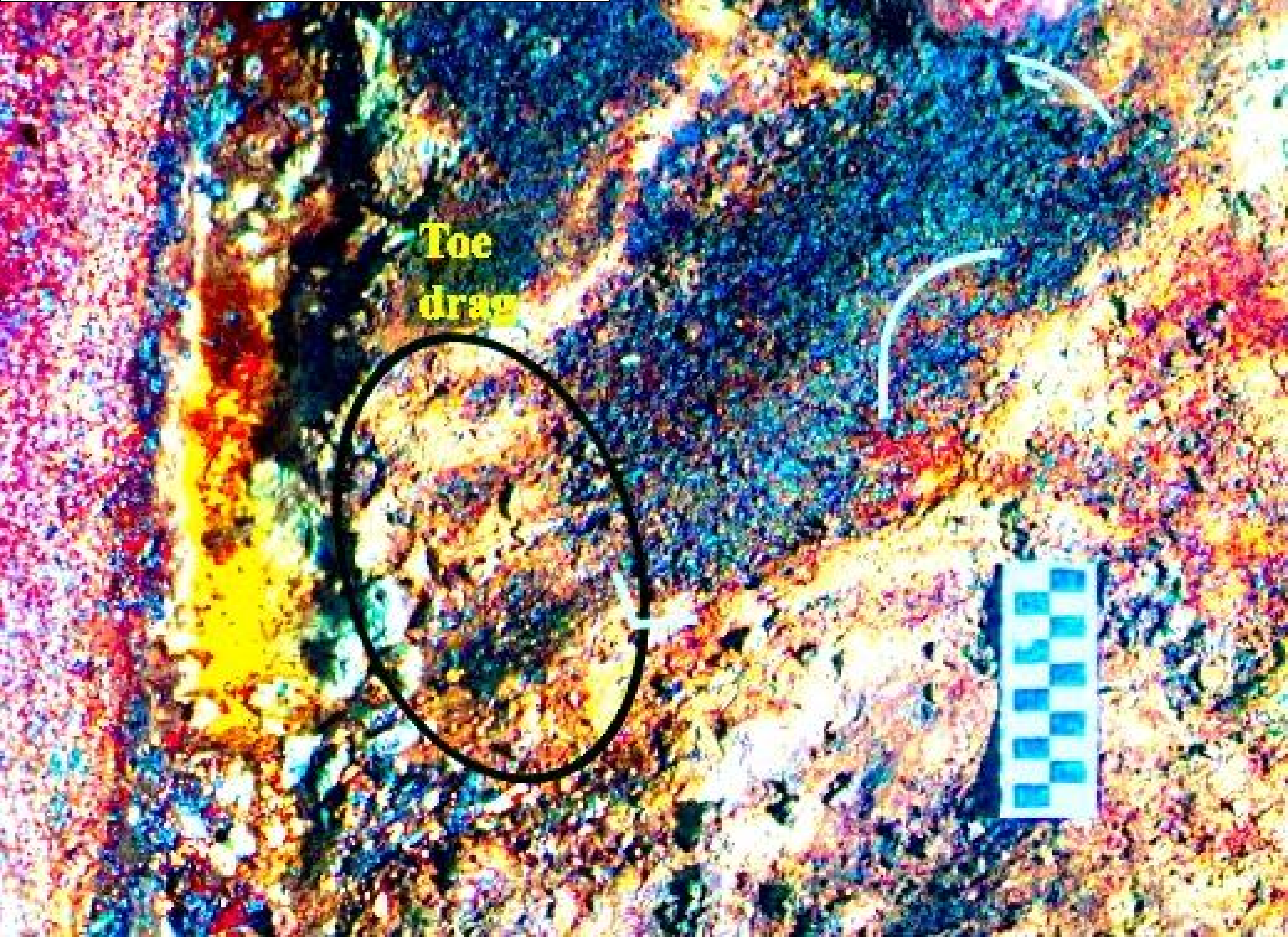

Footprint #23, Photo #1

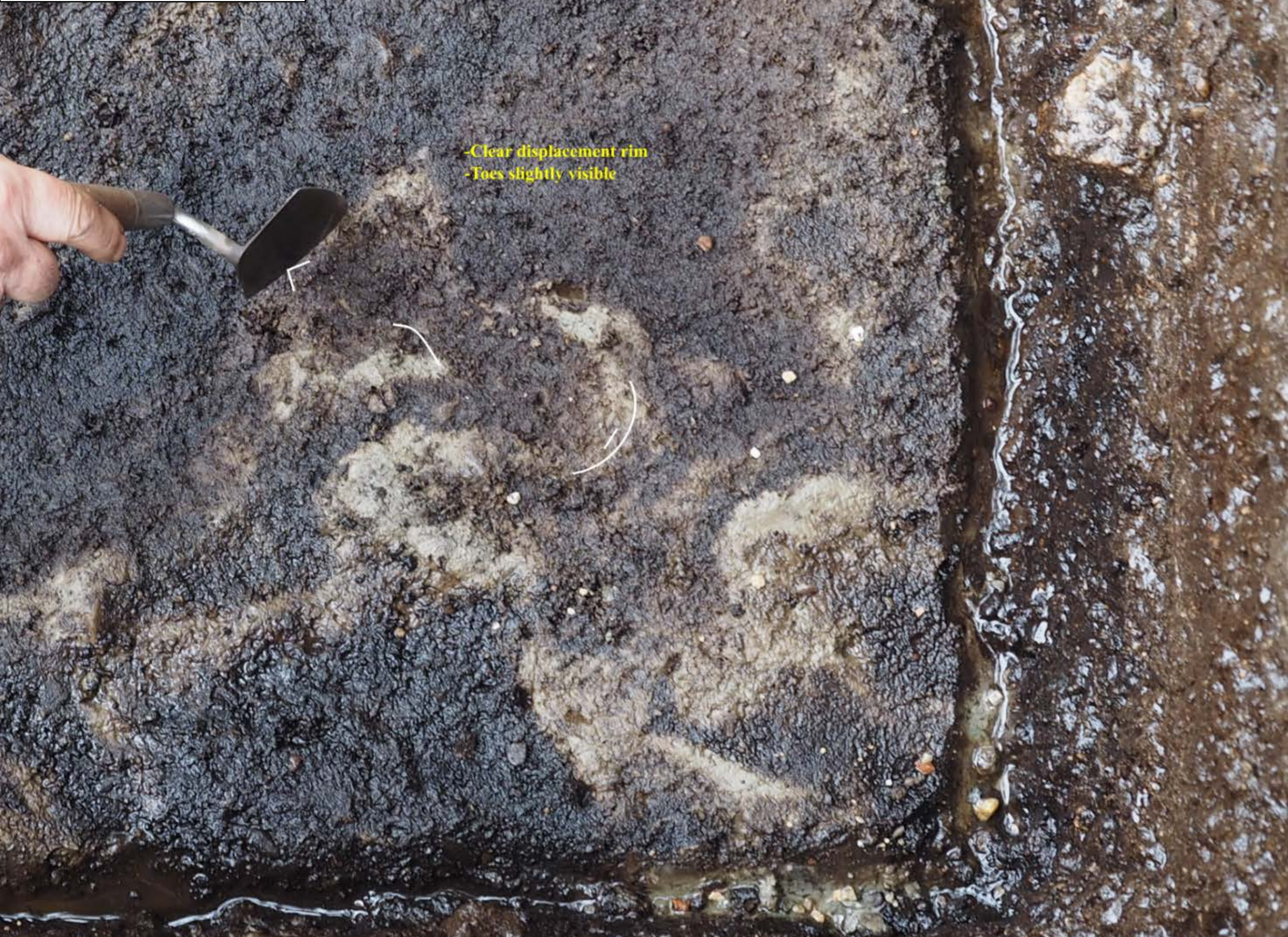

Footprint #23, Photo #2

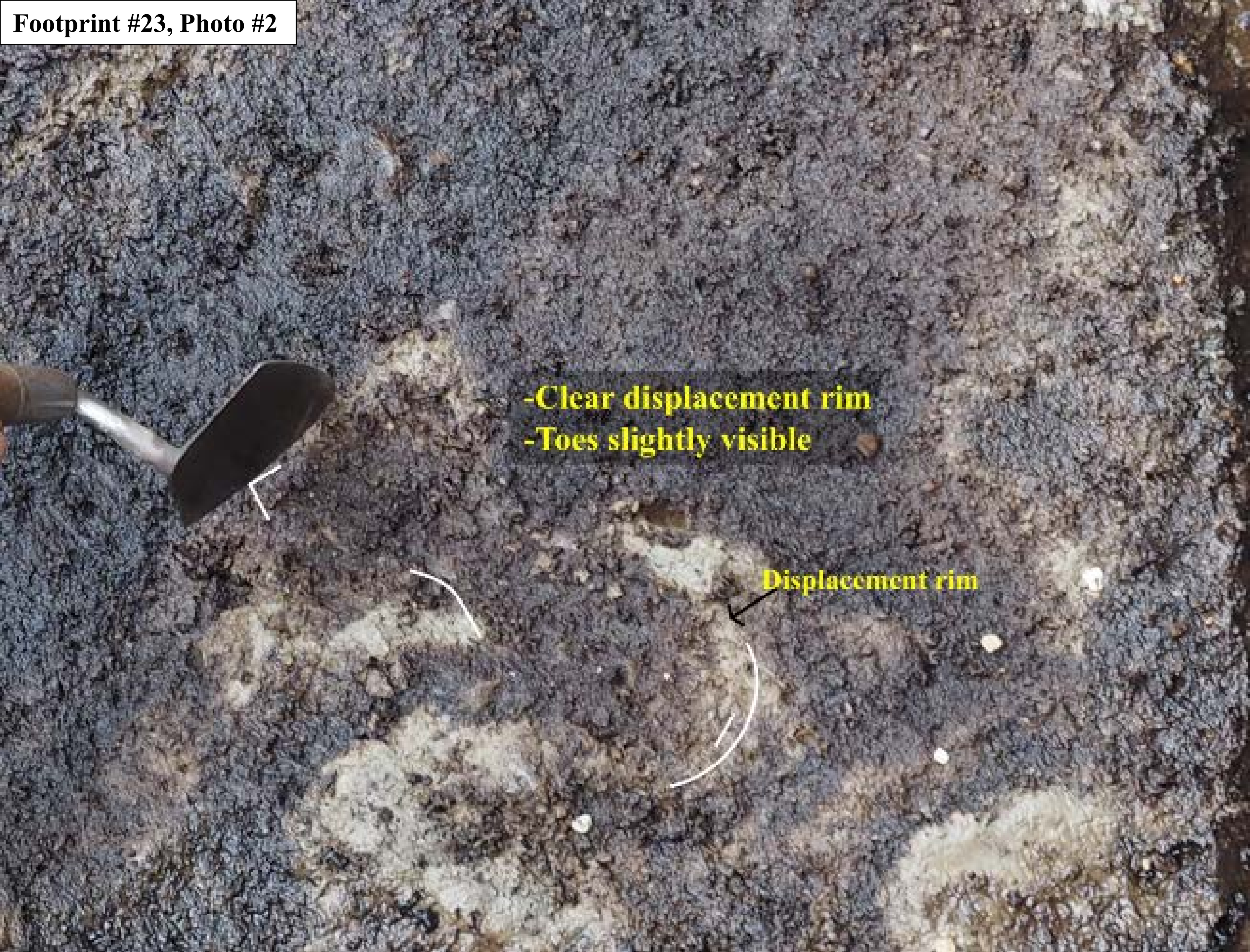

Footprint #23, Photo #3

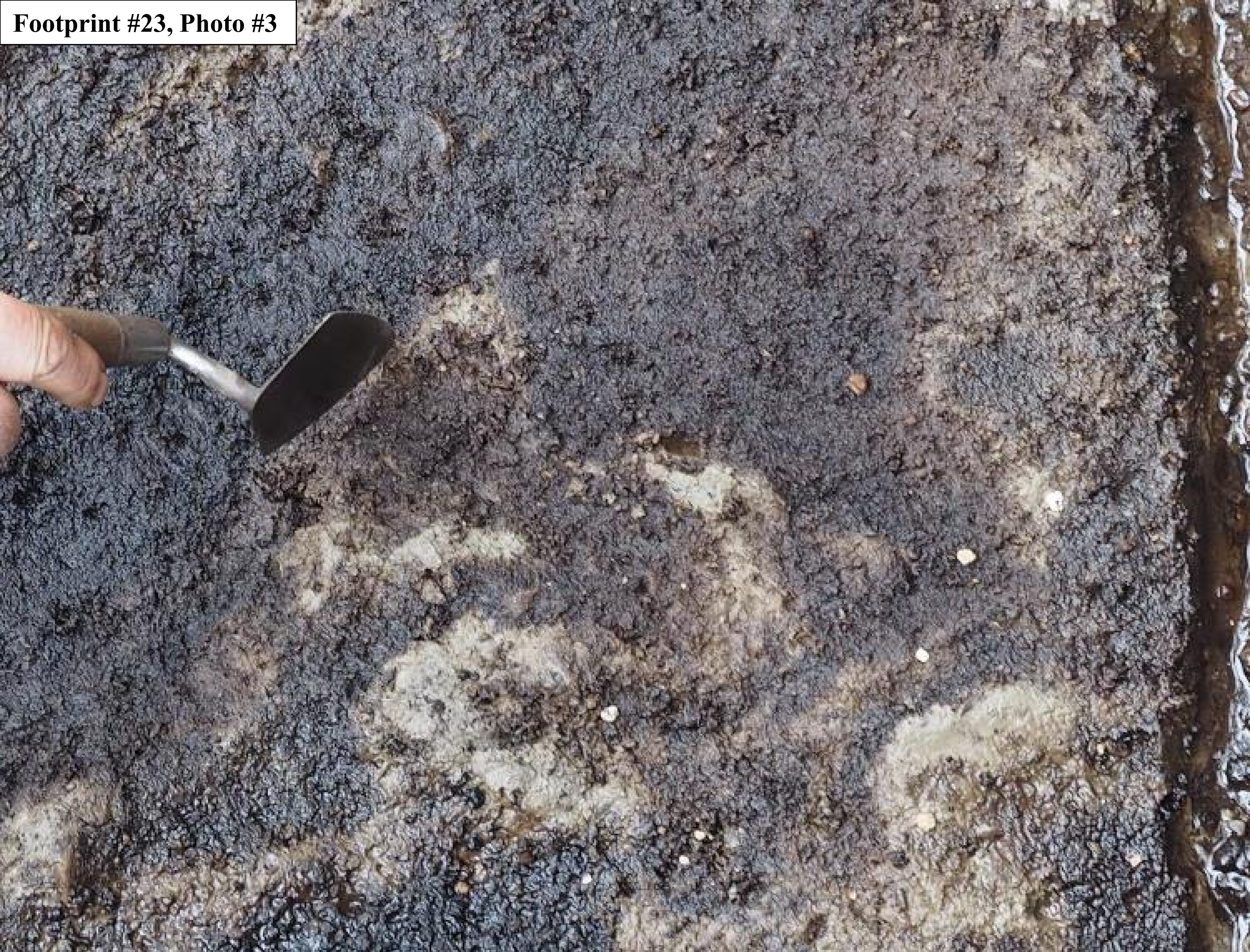

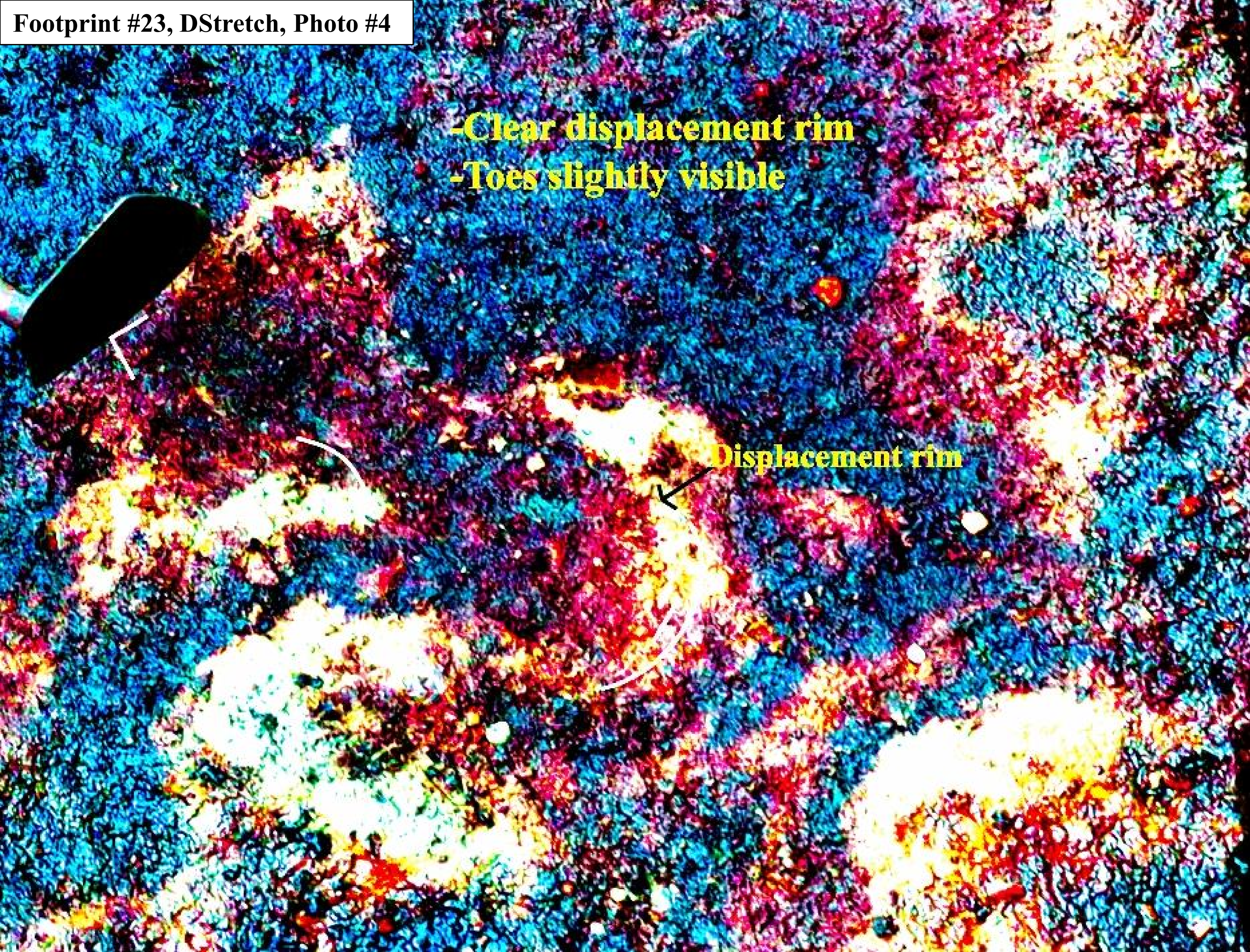
Footprint #23, DStretch, Photo #4
-Clear displacement rim
-Toes slightly visible
Displacement rim

Footprint #23, DStretch, Photo #5

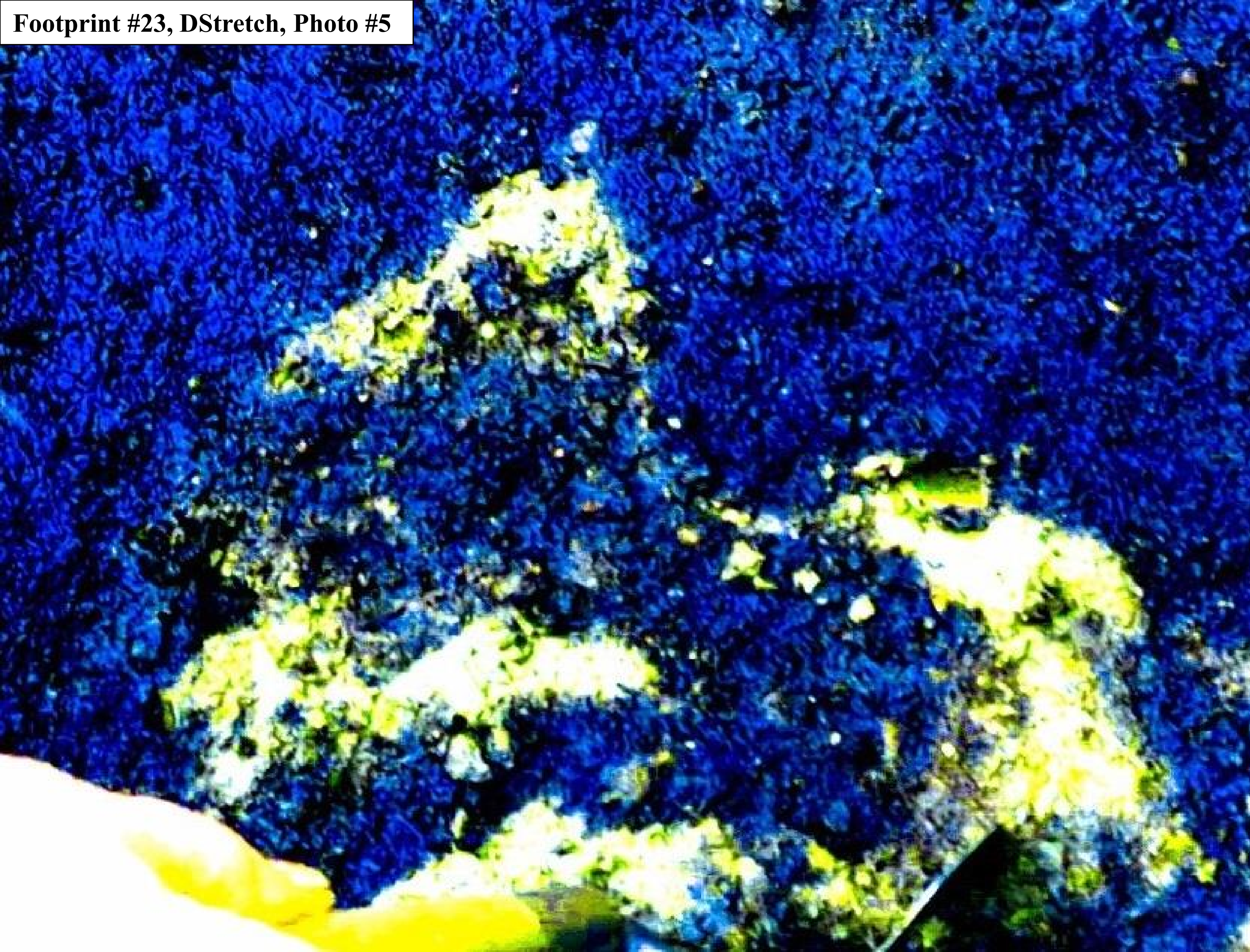

Footprint #23, Tracing F.P. Outline, Photo #6

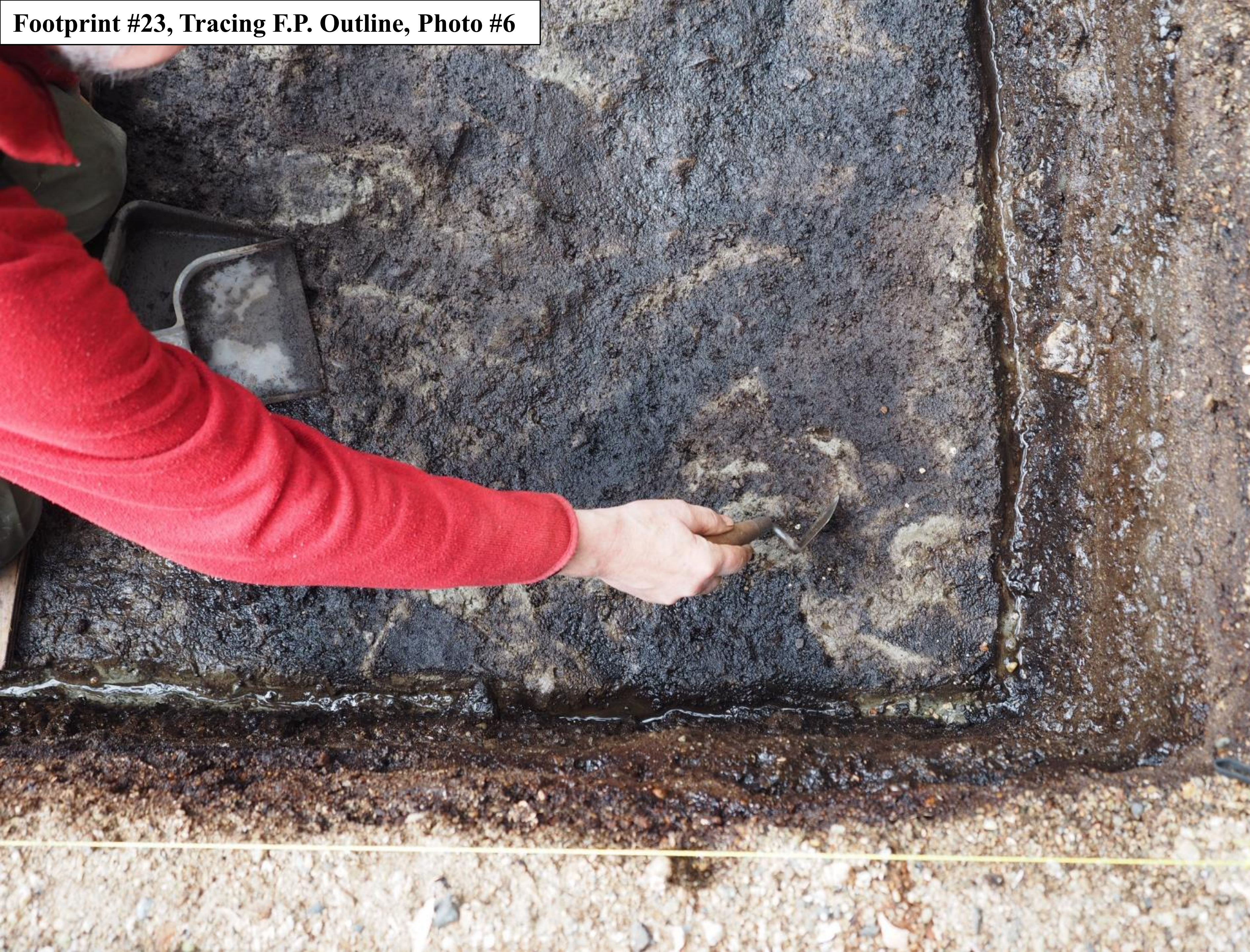

Footprint #23, Tracing F.P. Outline, Photo #7

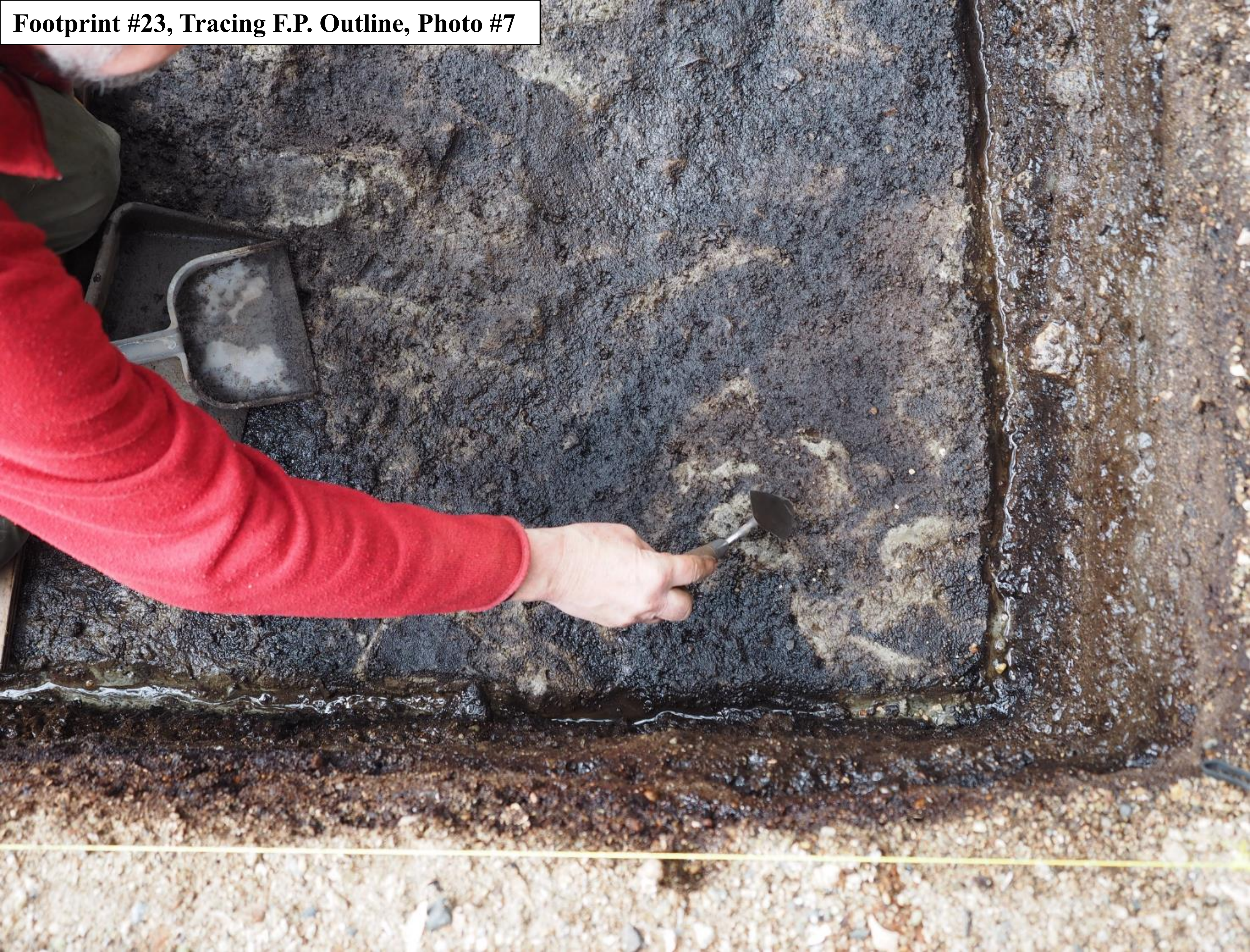

Footprint #23, Tracing F.P. Outline, Photo #8

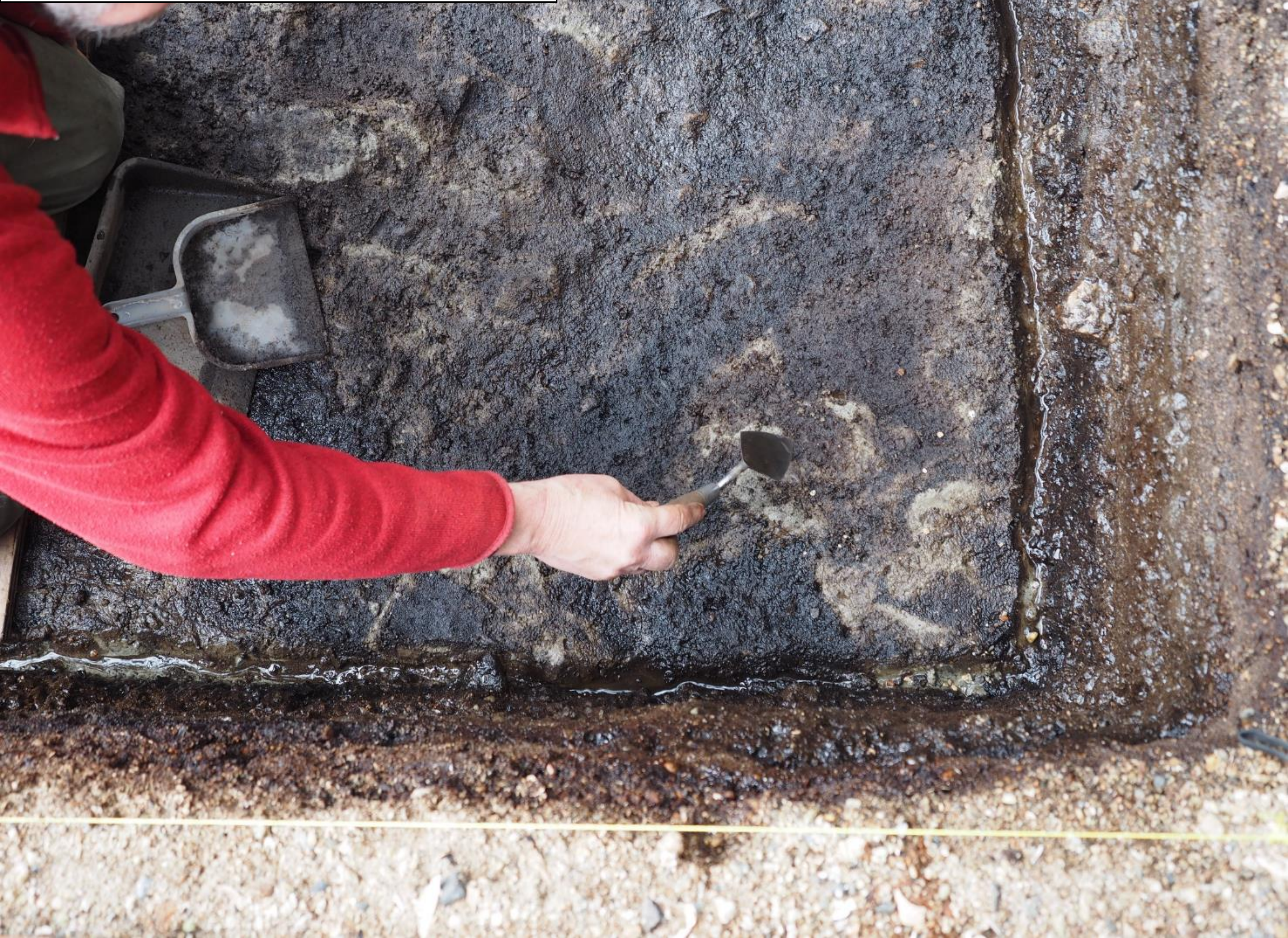

Footprint #23, Tracing F.P. Outline, Photo #9

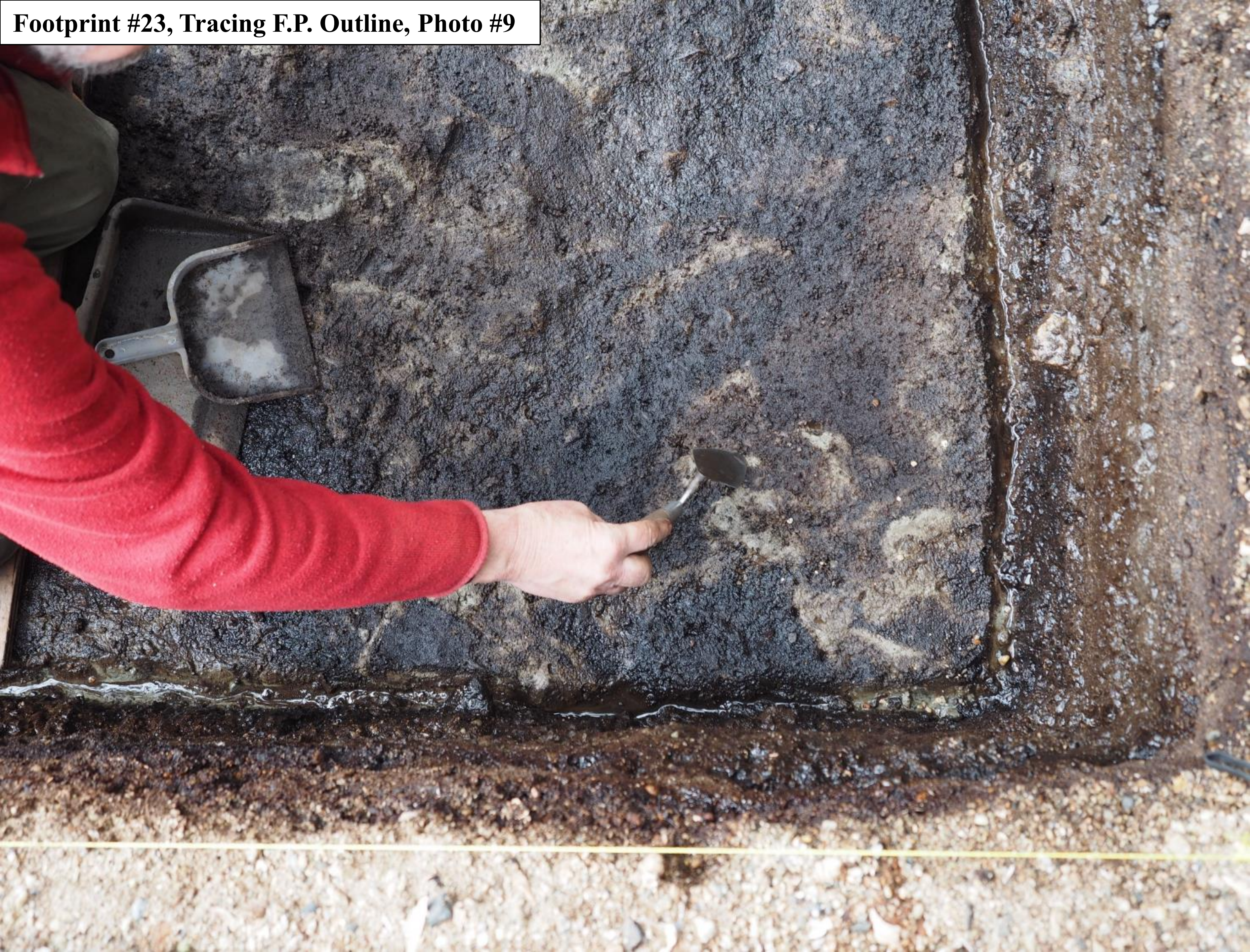

Footprint #23, Tracing F.P. Outline, Photo #10

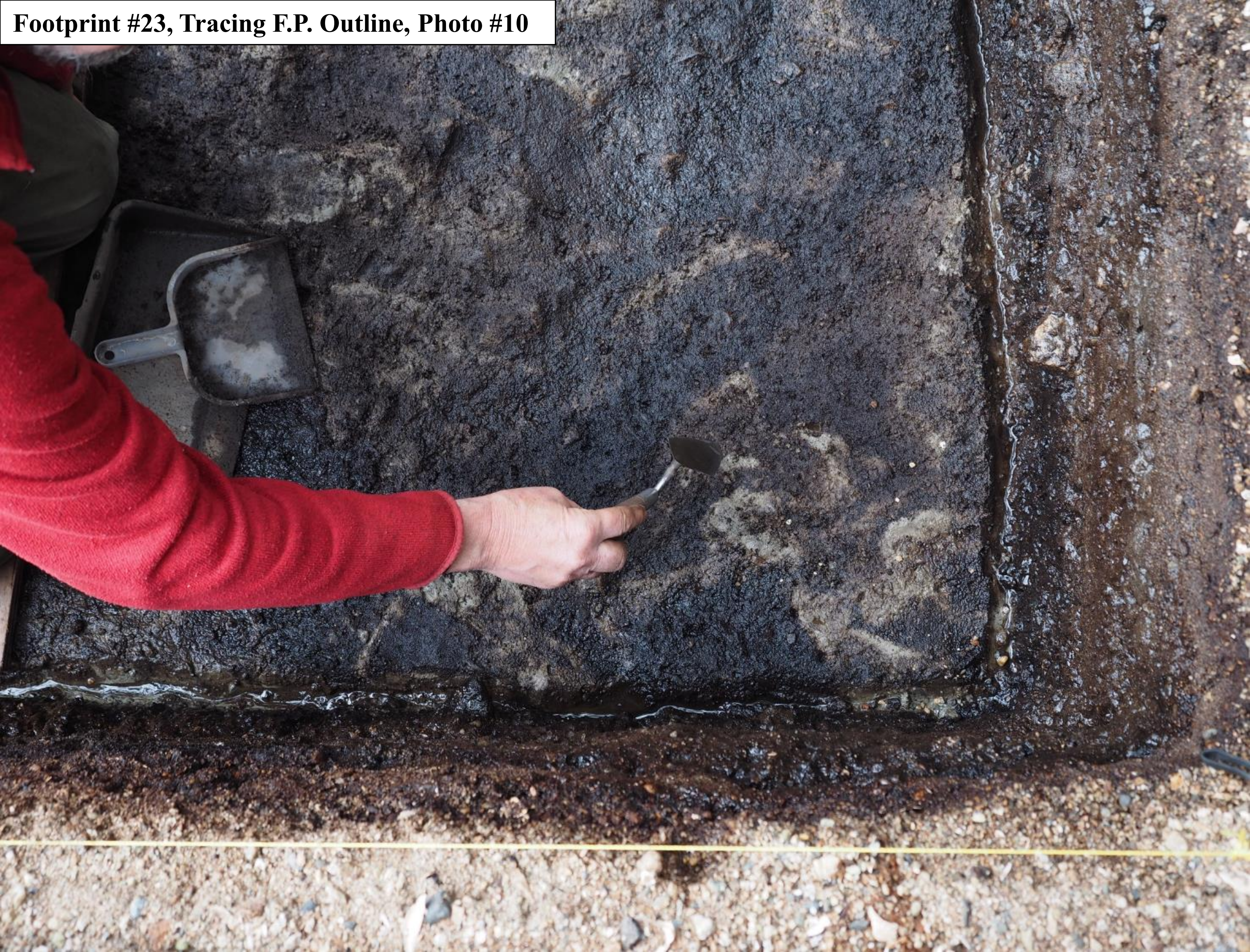

Footprint #23, Tracing F.P. Outline, Photo #11

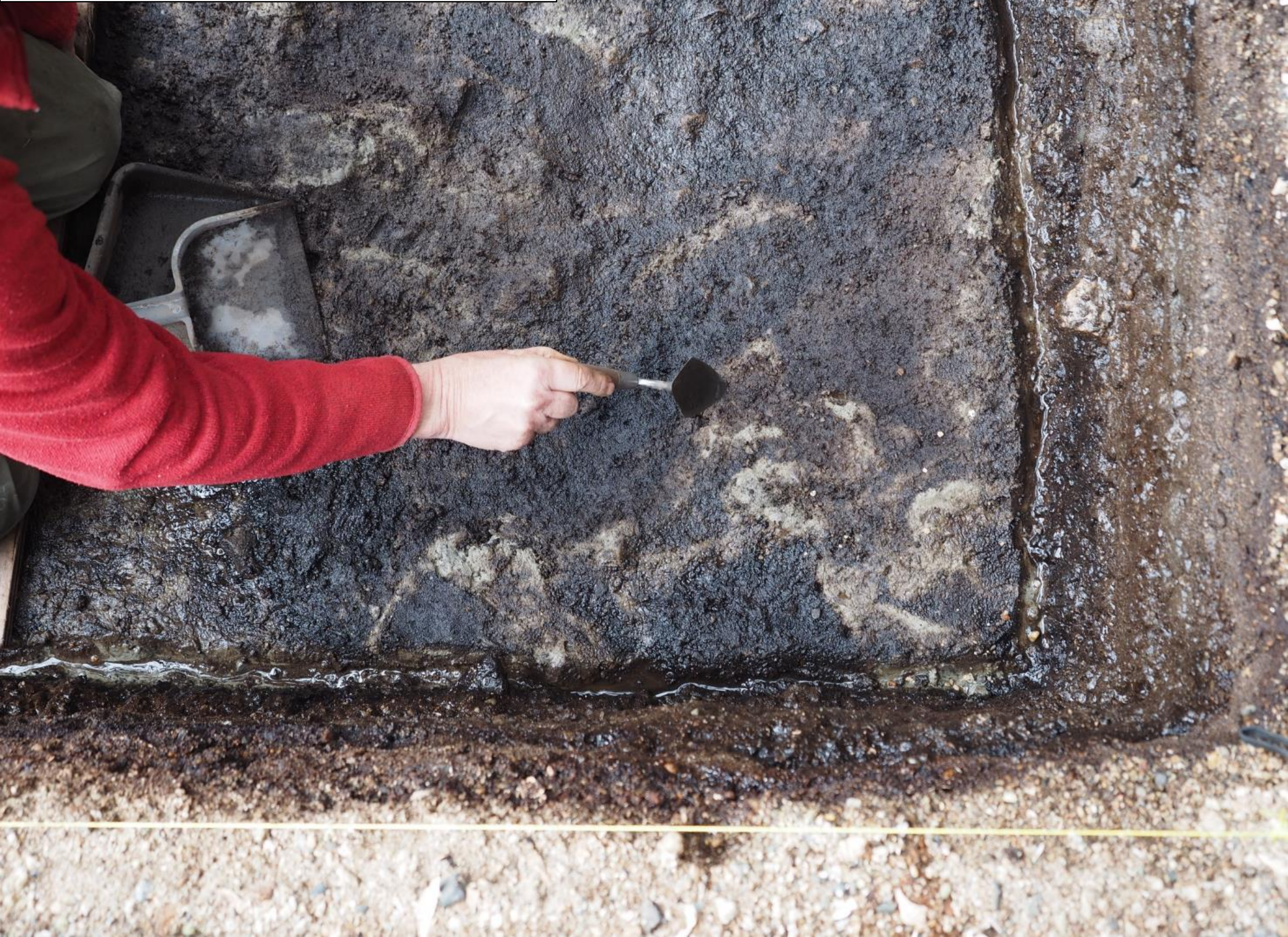

Footprint #23, Tracing F.P. Outline, Photo #12

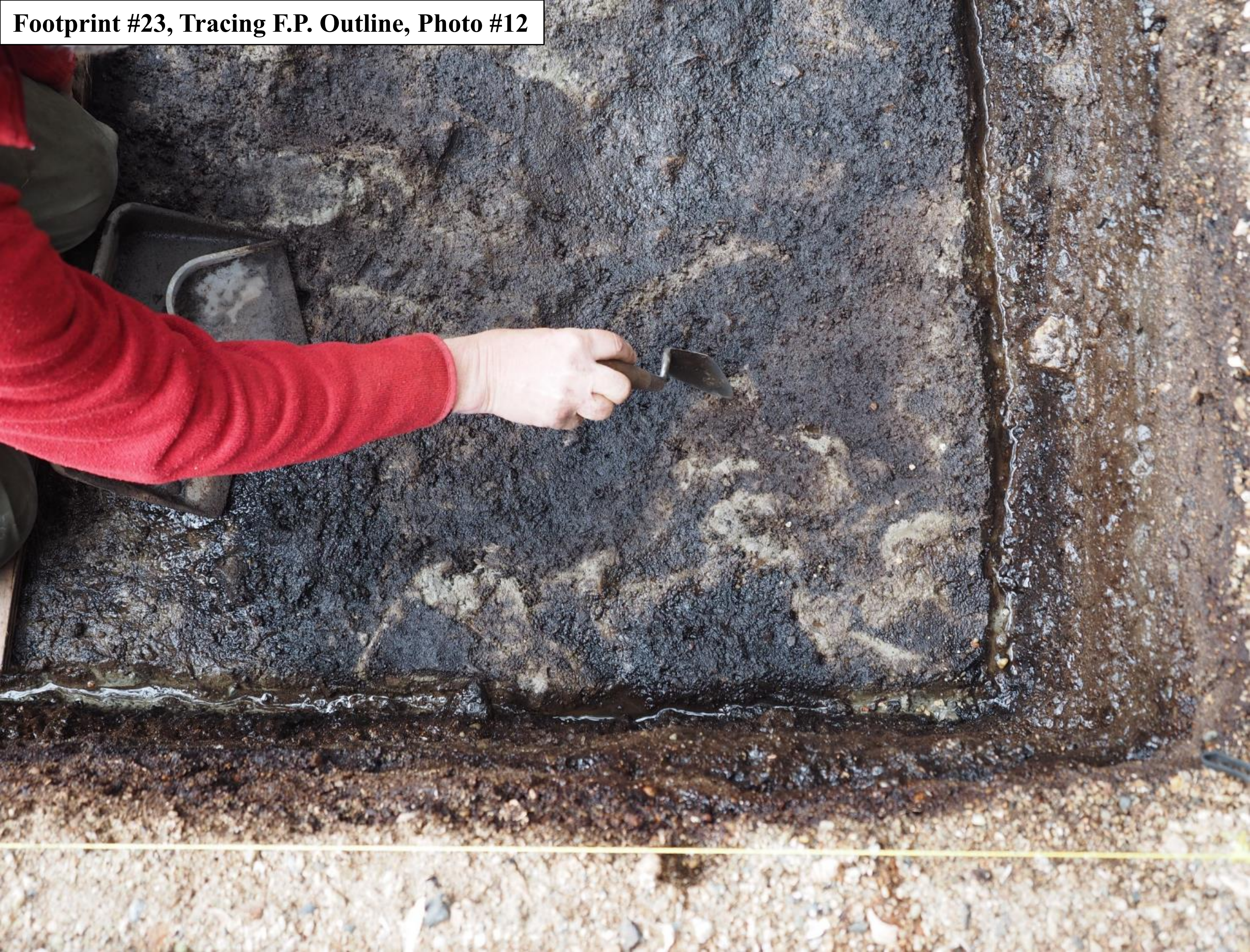

Footprint #23, Tracing F.P. Outline, Photo #13

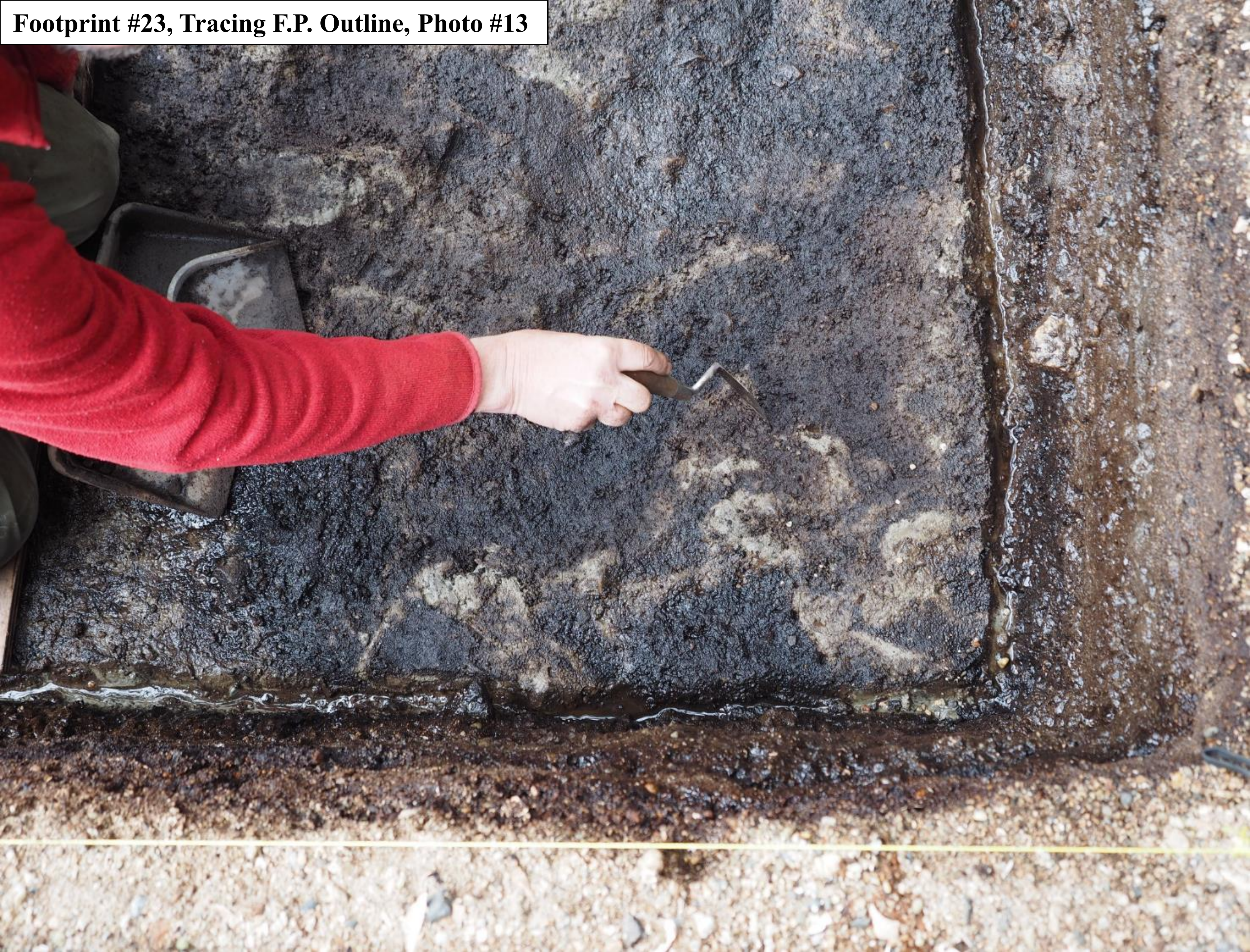

Footprint #23, Tracing F.P. Outline, Photo #14

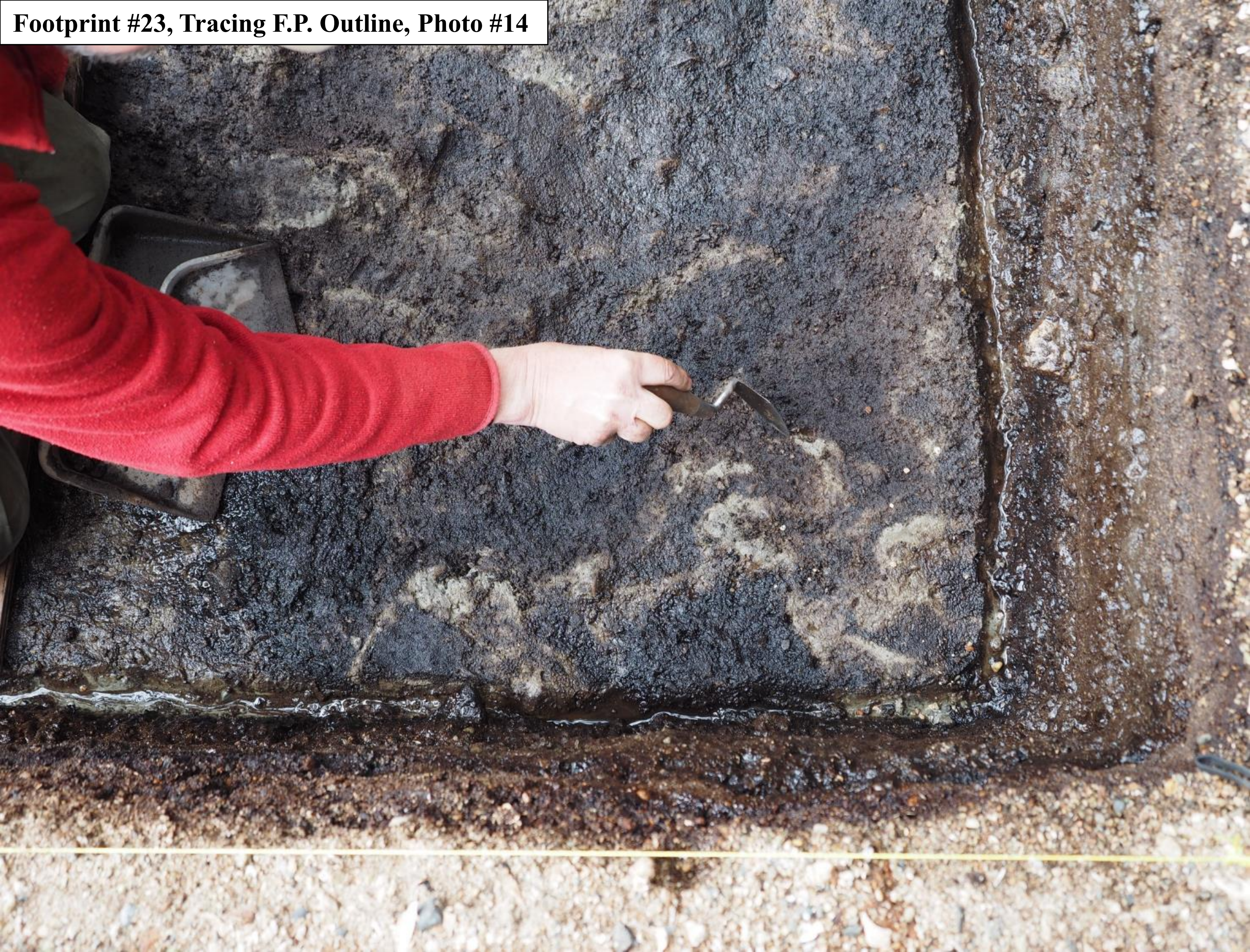

Footprint #23, Tracing F.P. Outline, Photo #15

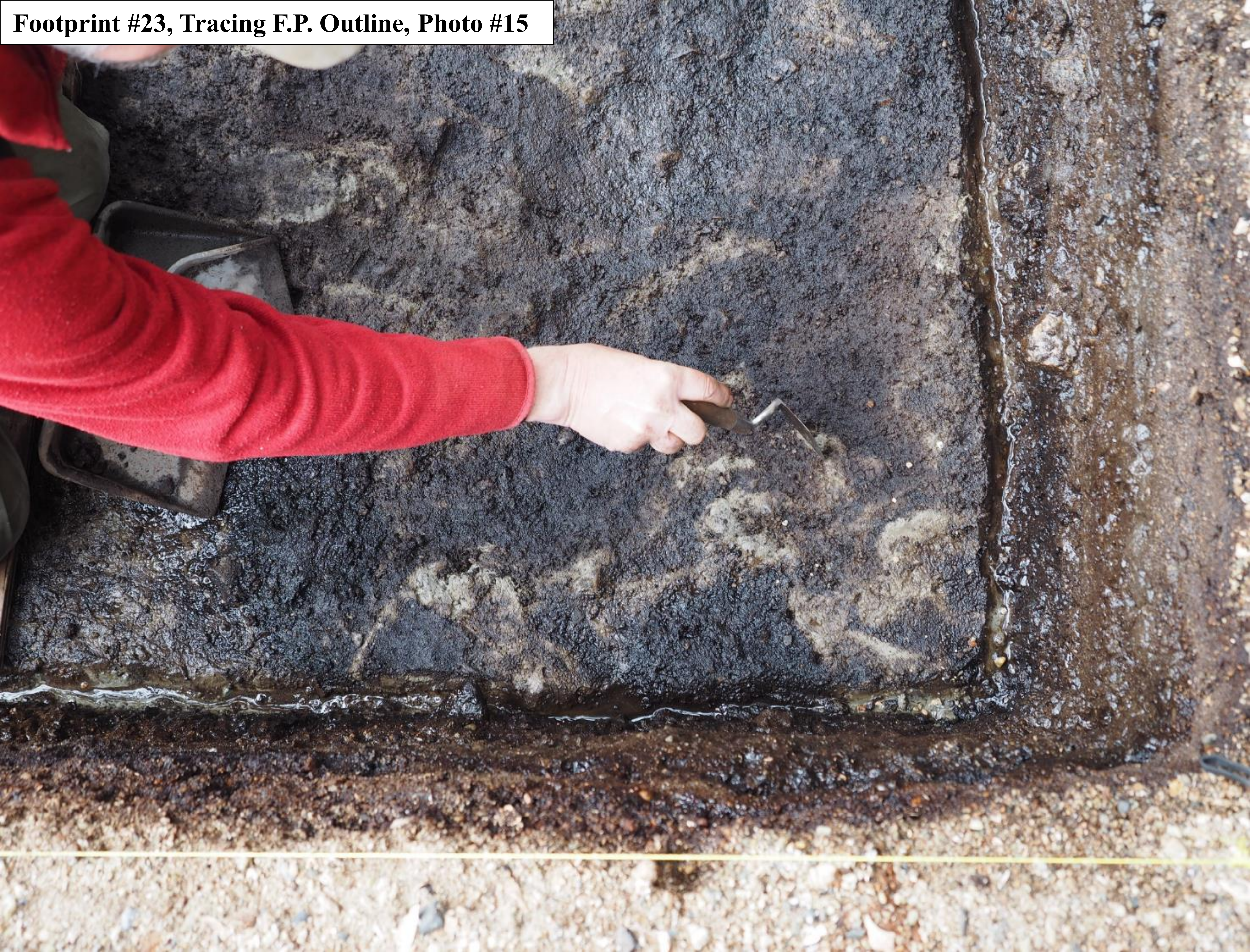

Footprint #18, Photo #1

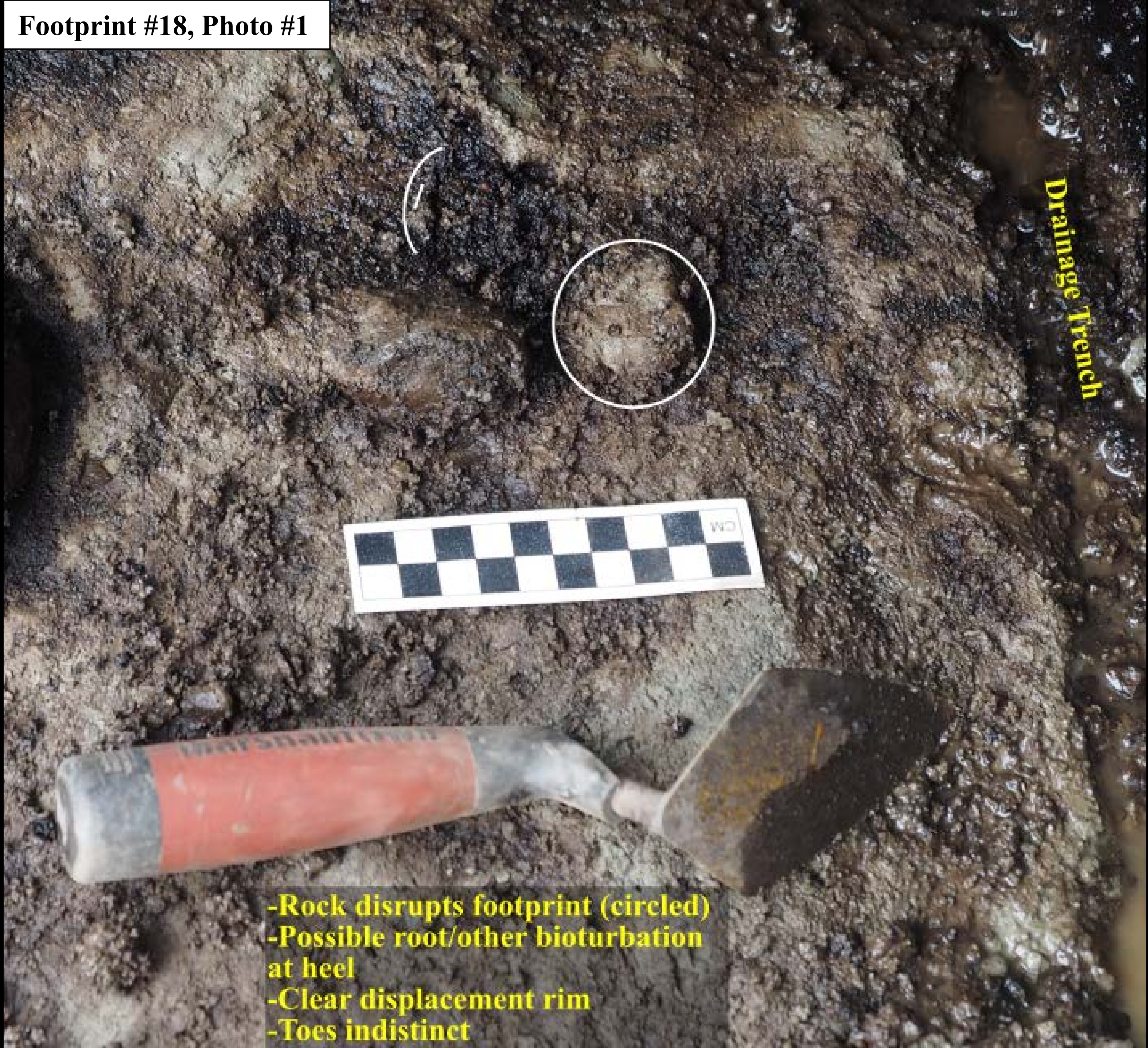

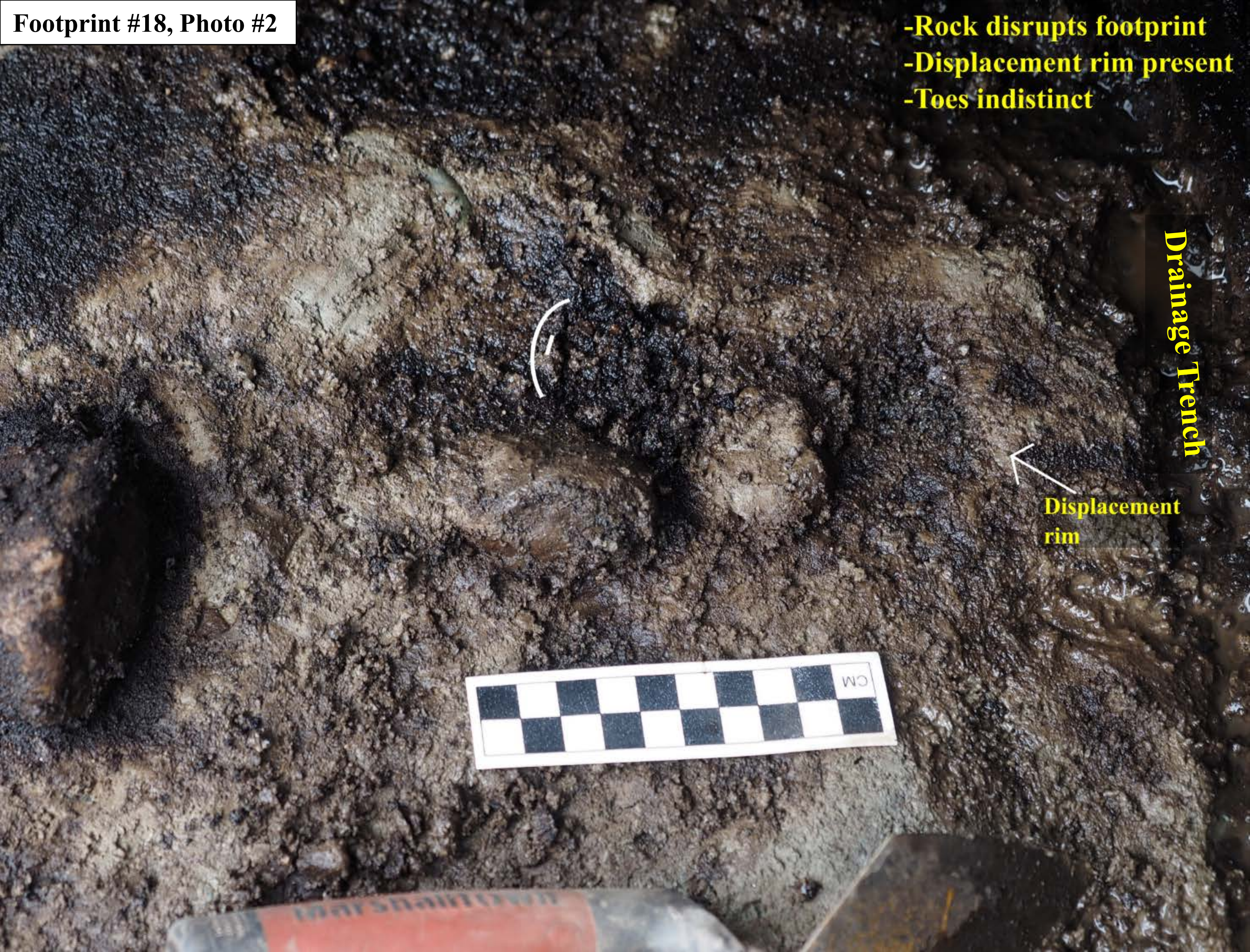
Footprint #18, Photo #2
-Rock disrupts footprint
-Displacement rim present
-Toes indistinct
Drainage Trench
Displacement rim
CM

Footprint #18, DStretch Photo #3

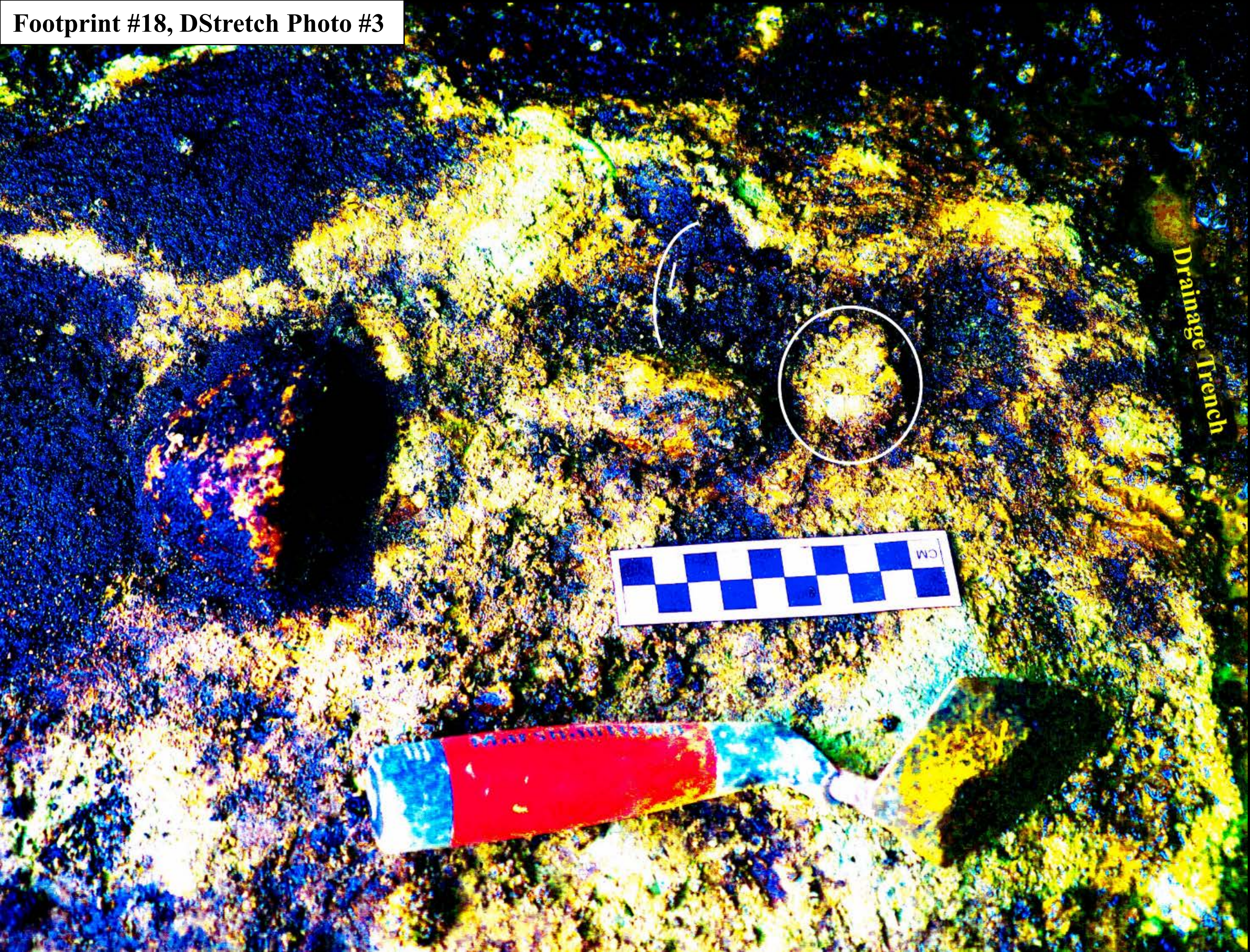

Footprint #24, Side Profile, Photo #1

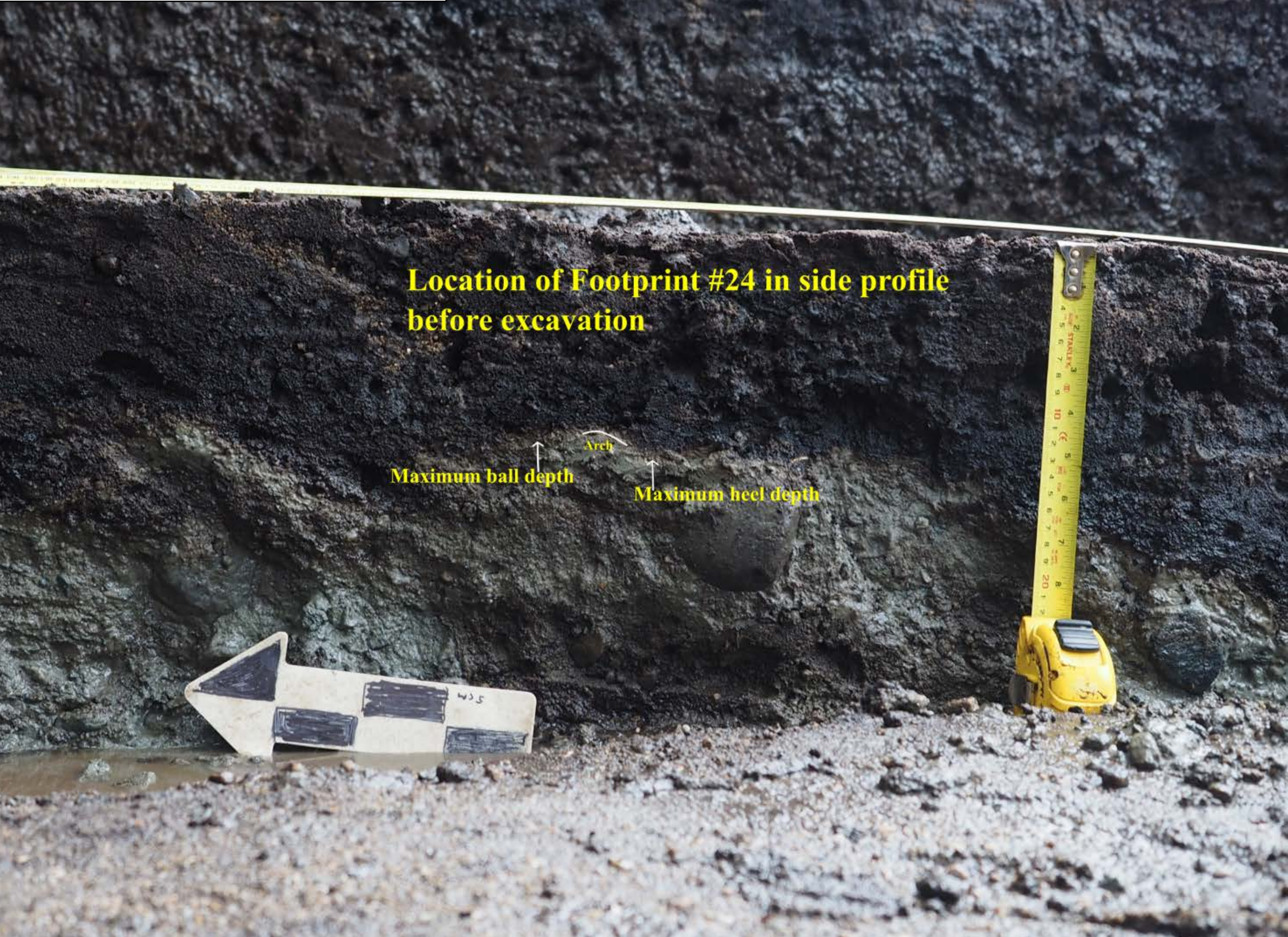

Footprint #24, Side Profile, Photo #2

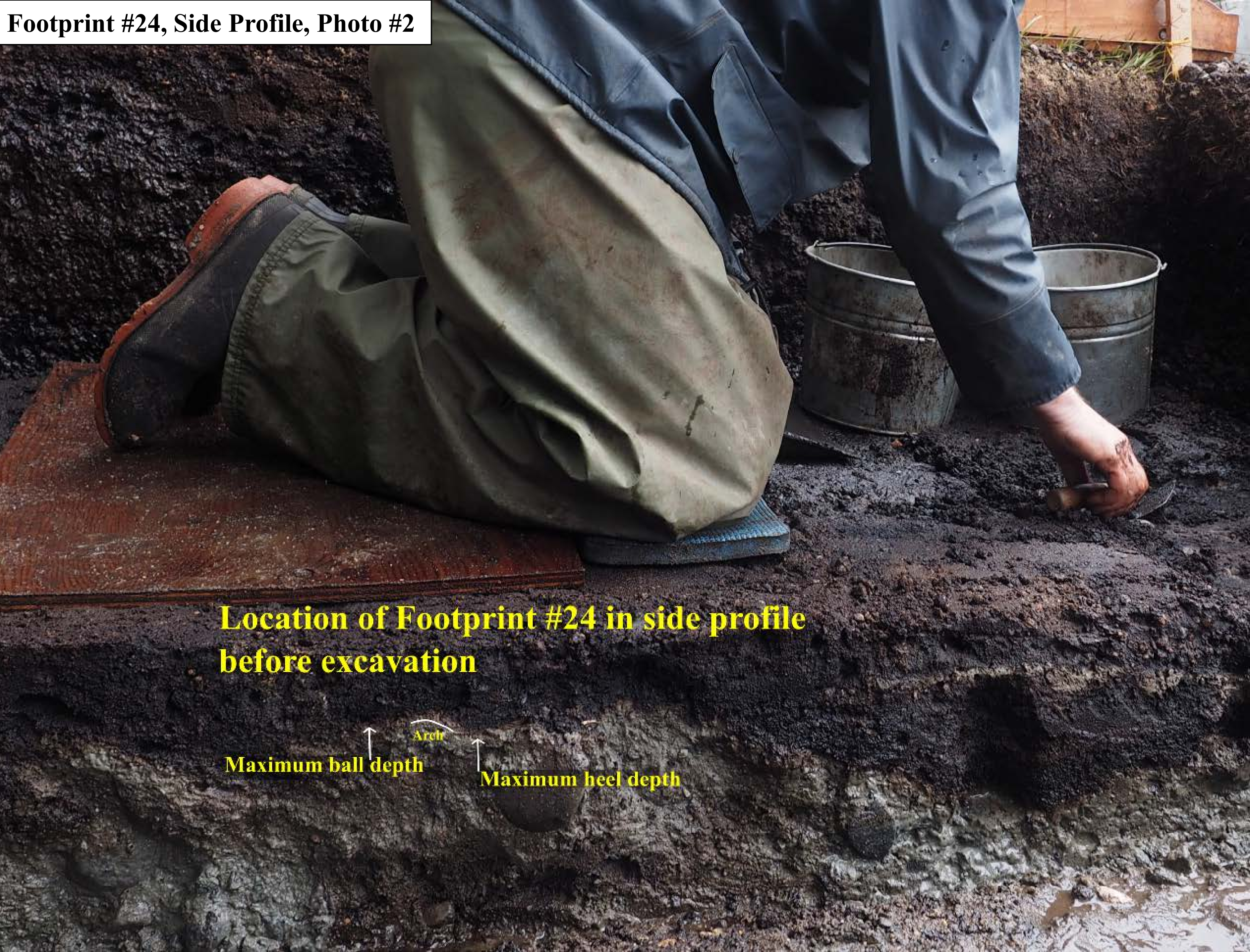

Footprint #24, Side Profile, Photo #3

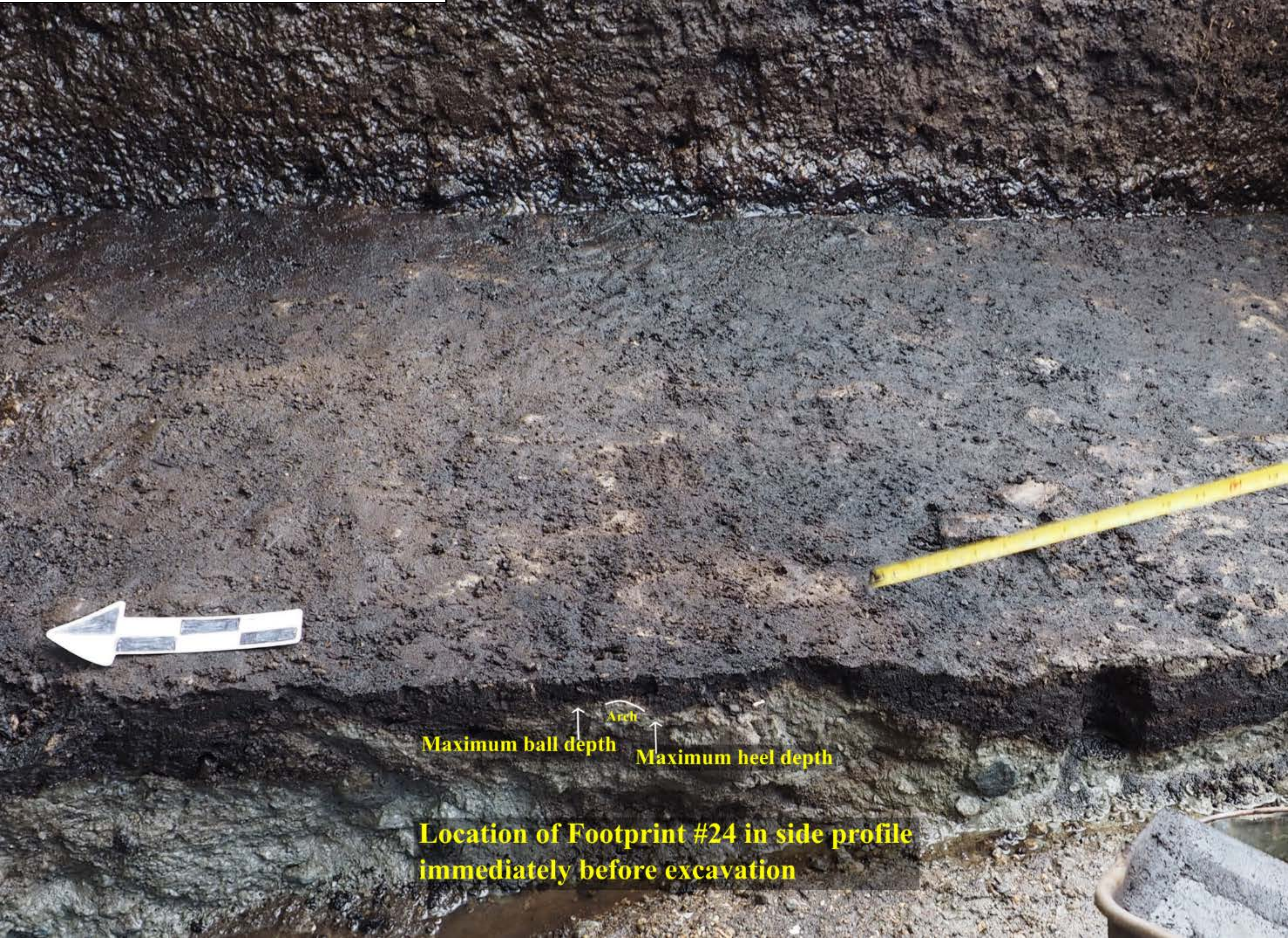

Footprint #24, Side Profile, Photo #4

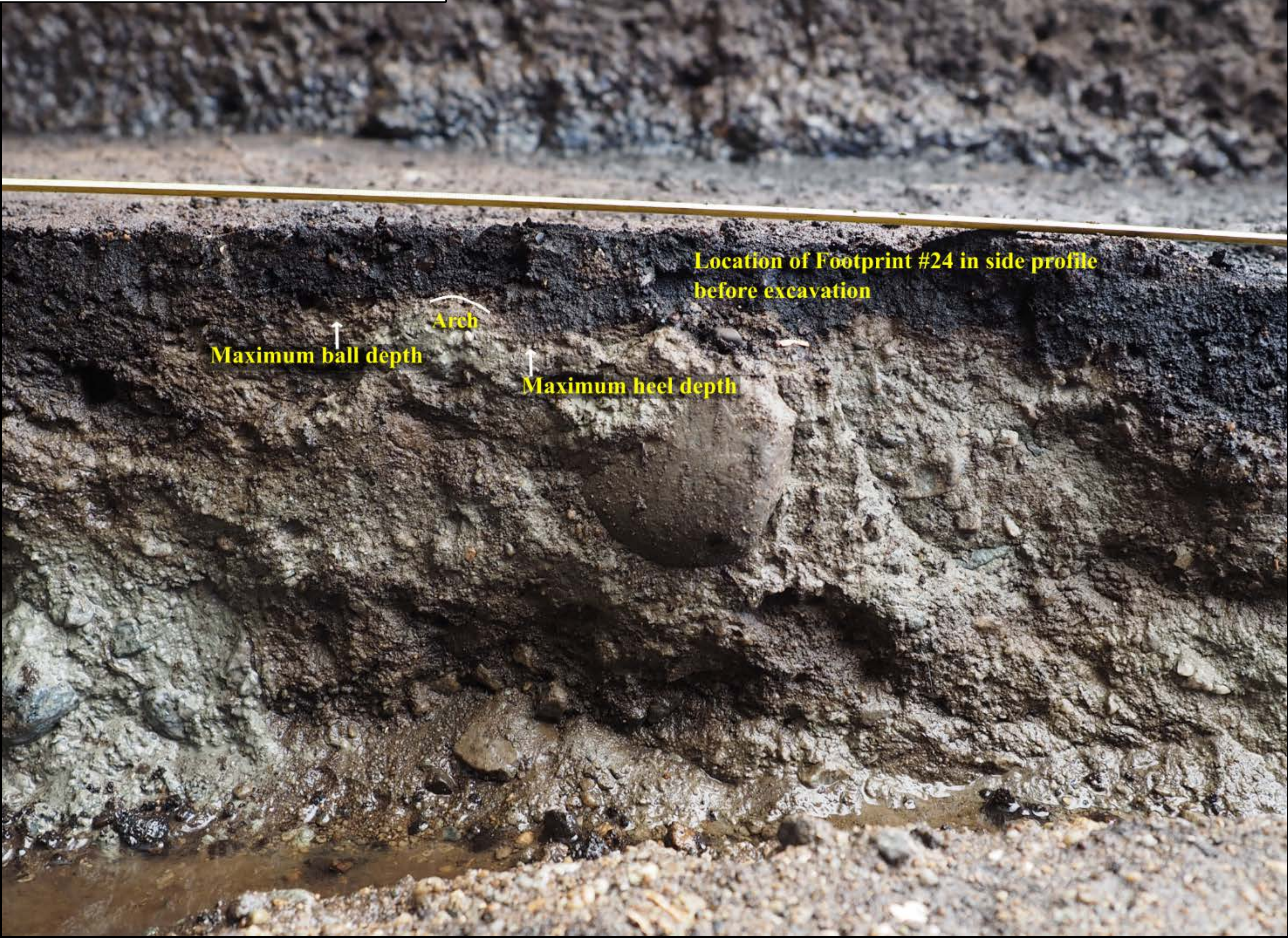

Footprint #24, Beginning to Excavate, Photo #5

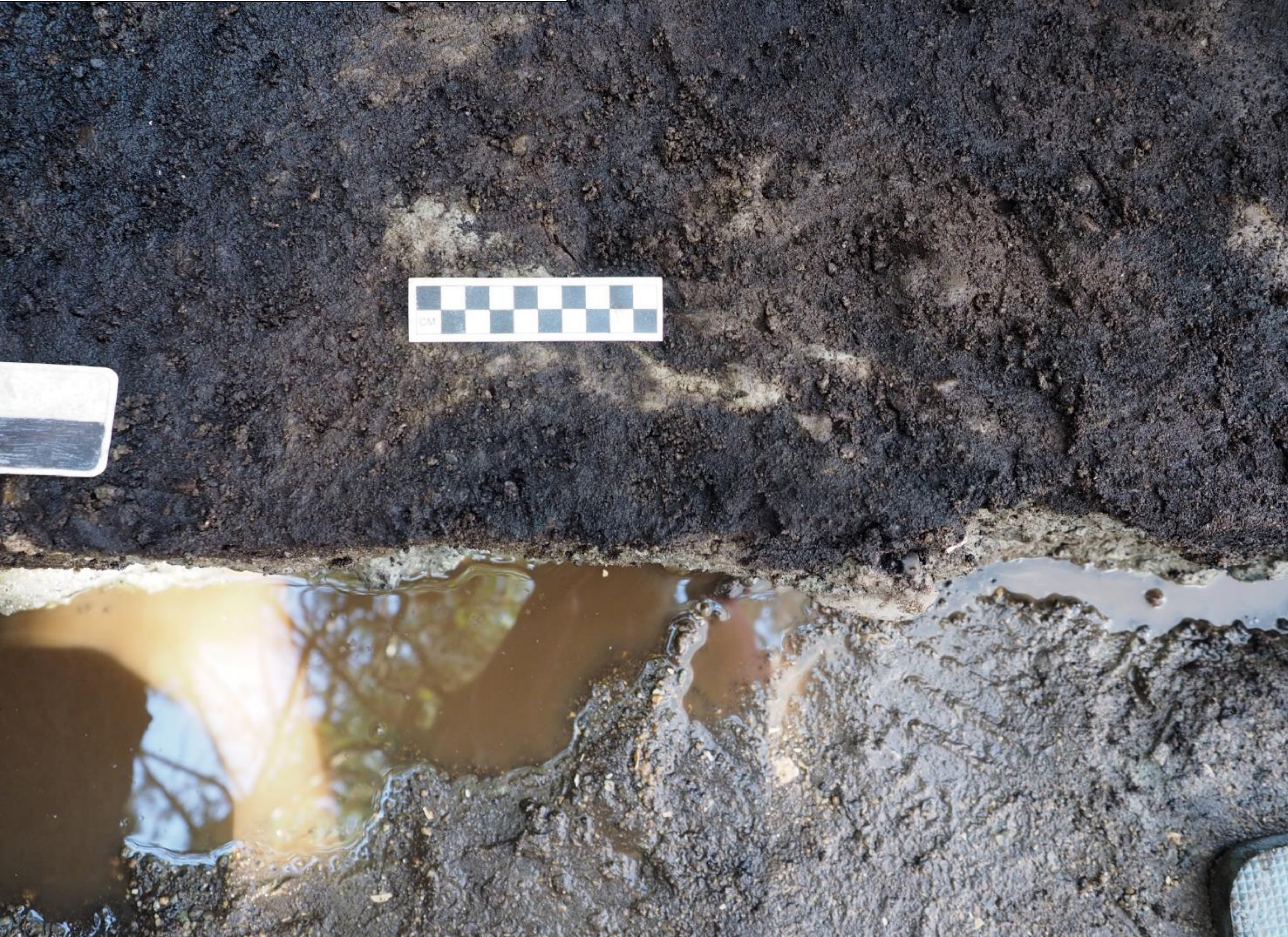

Footprint #24, Beginning to Excavate-DStretch, Photo #6

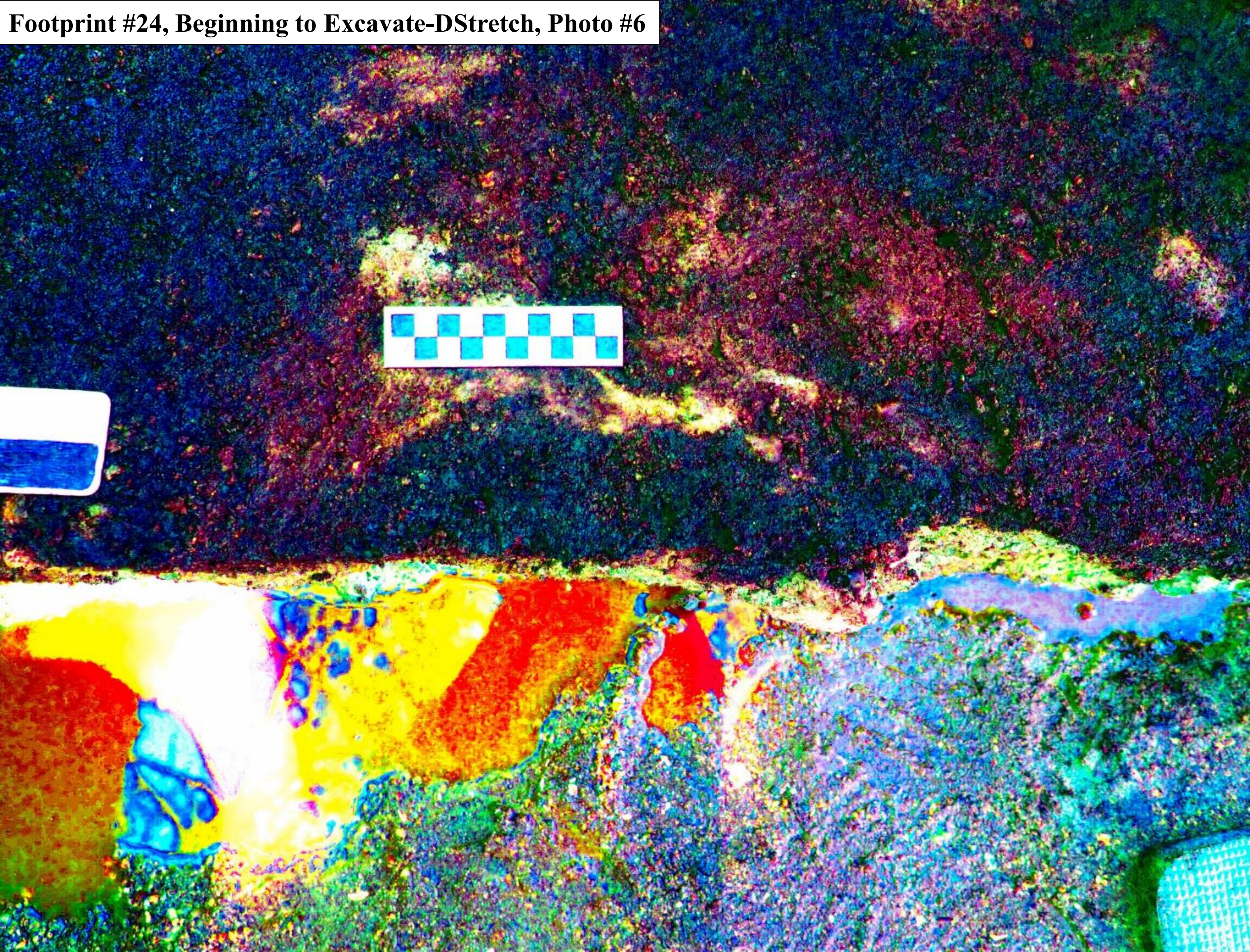

Footprint #24, Photo #7

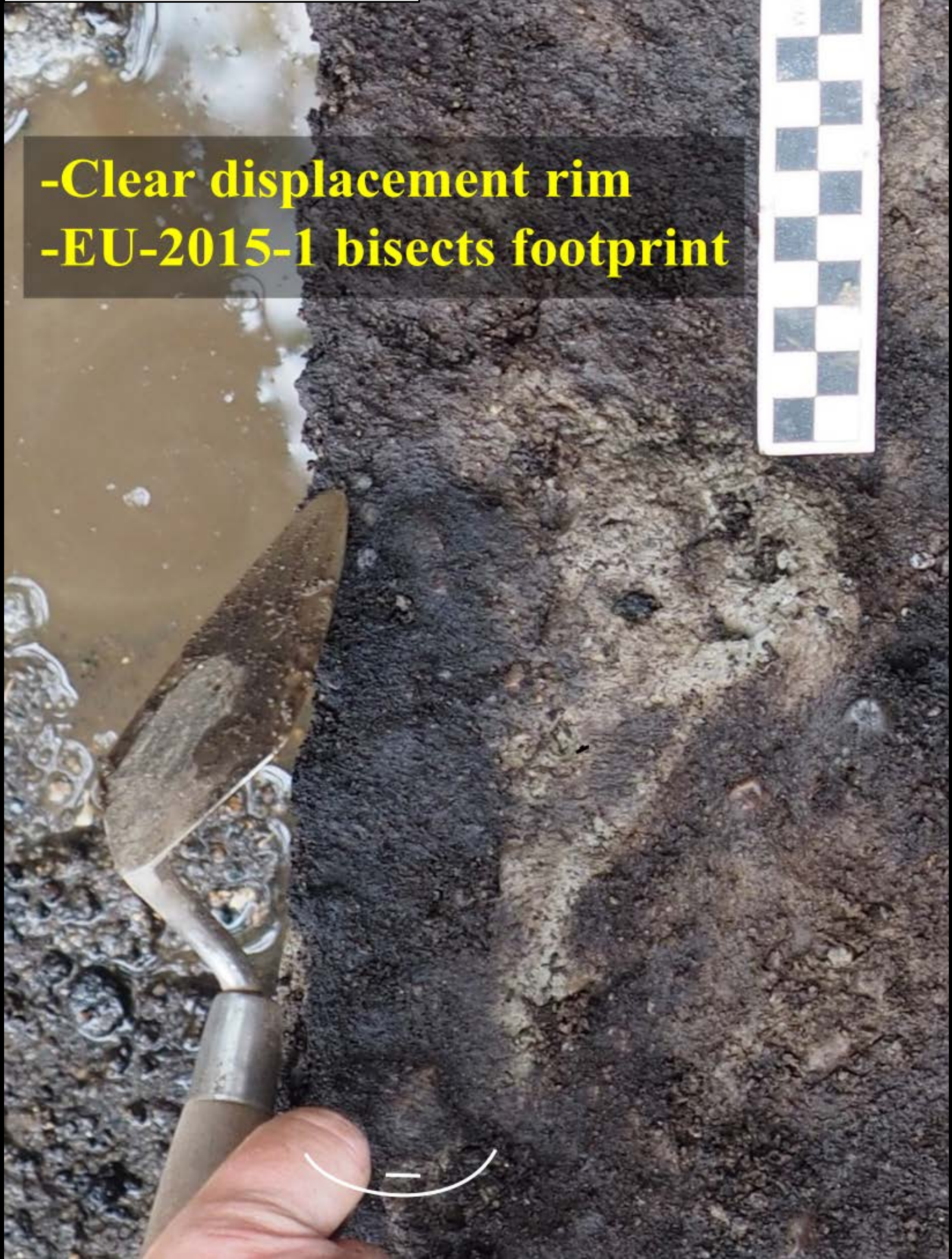

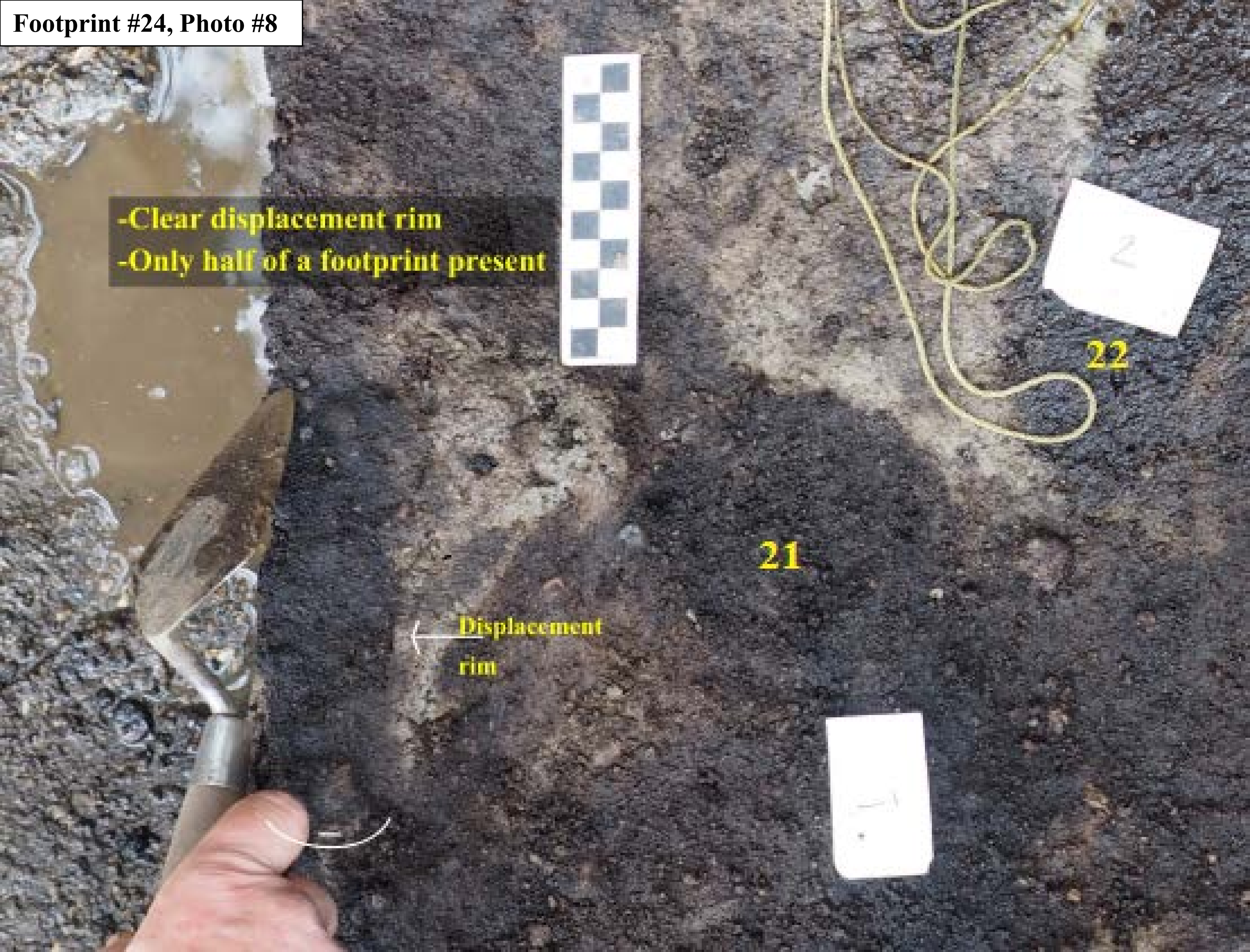
Footprint #24, Photo #8
-Clear displacement rim
-Only half of a footprint present
22
21
Displacement rim

Footprint #24, Tracing F.P. Outline, Photo #9

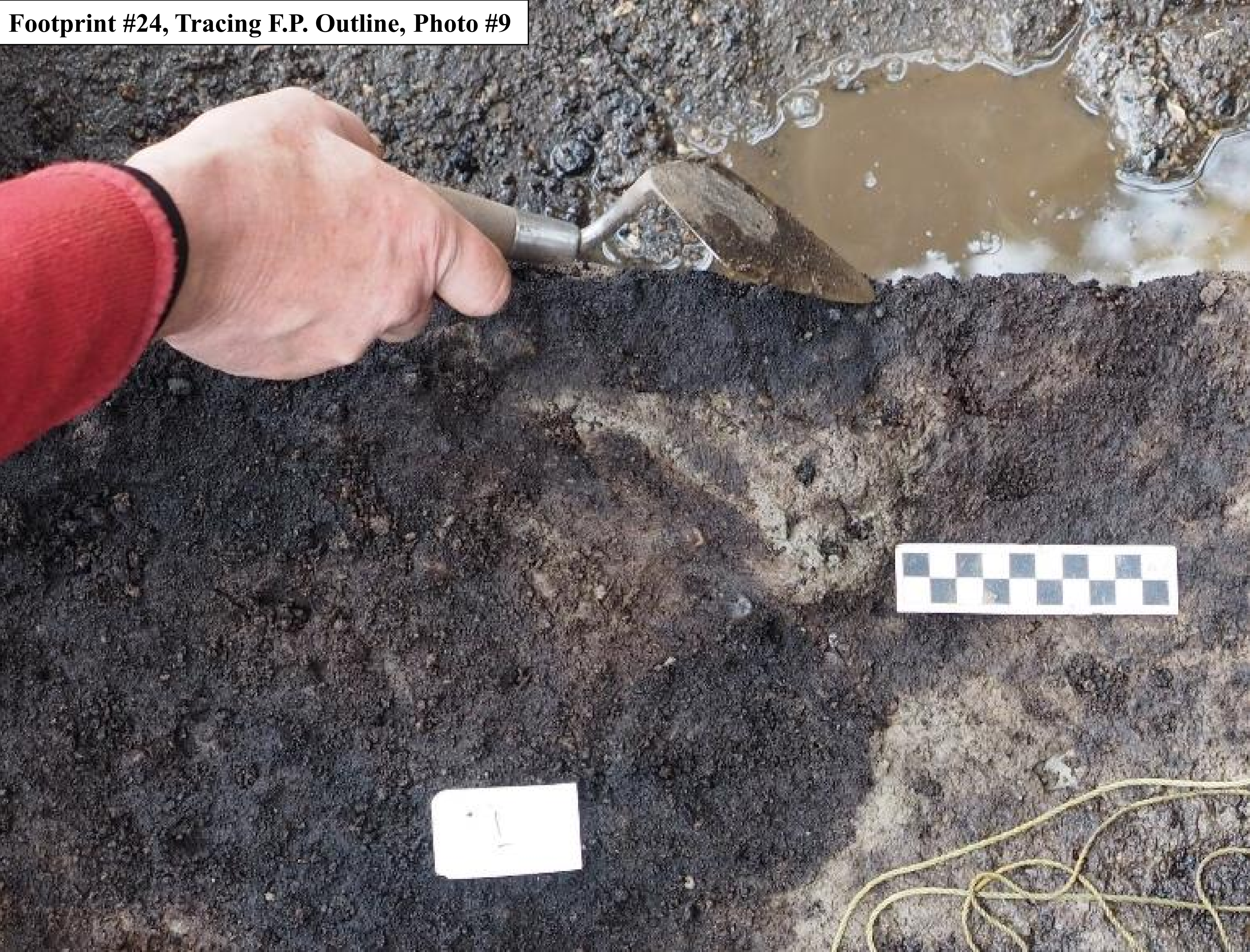

Footprint #24, Tracing F.P. Outline, Photo #10

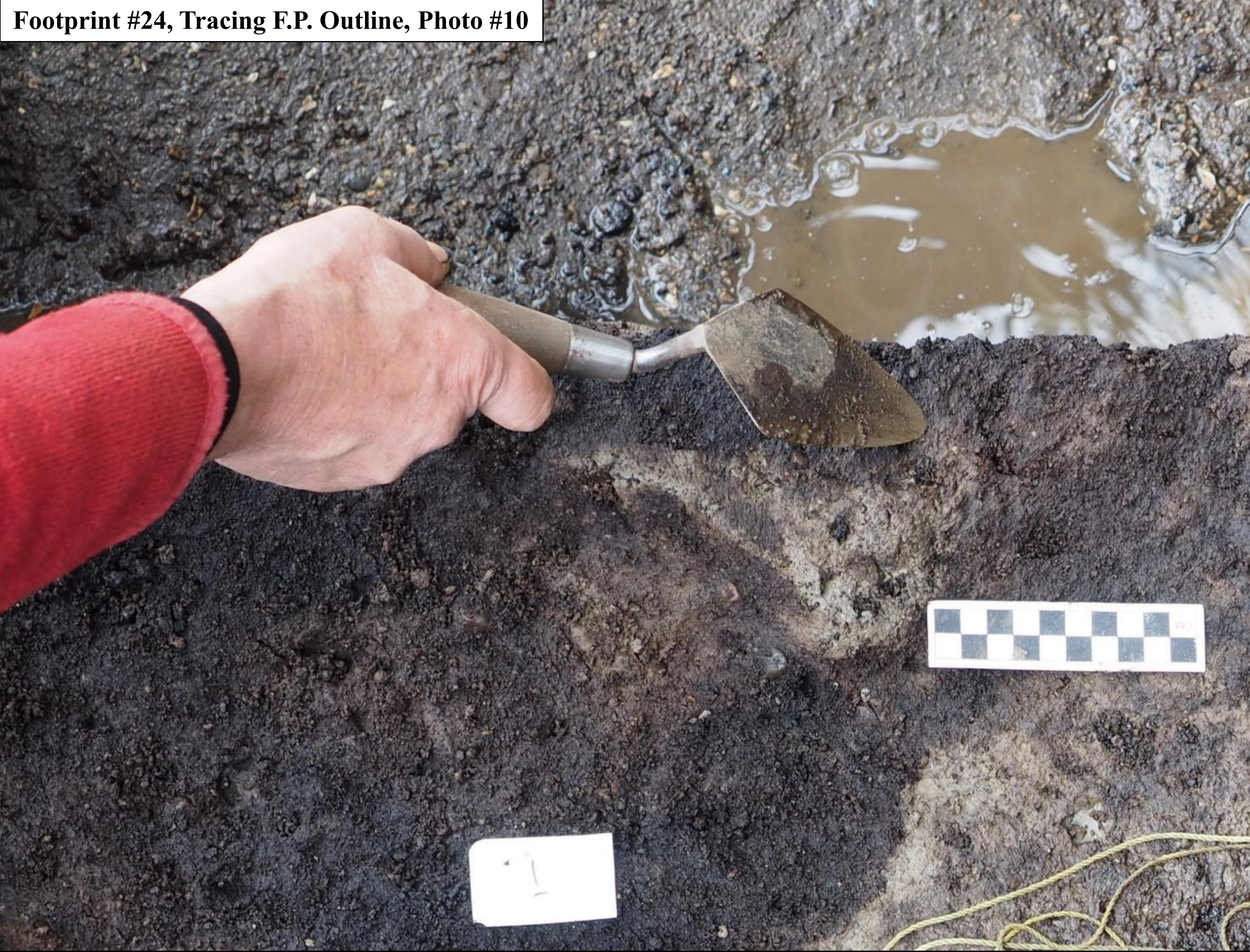

Footprint #24, Tracing F.P. Outline, Photo #11

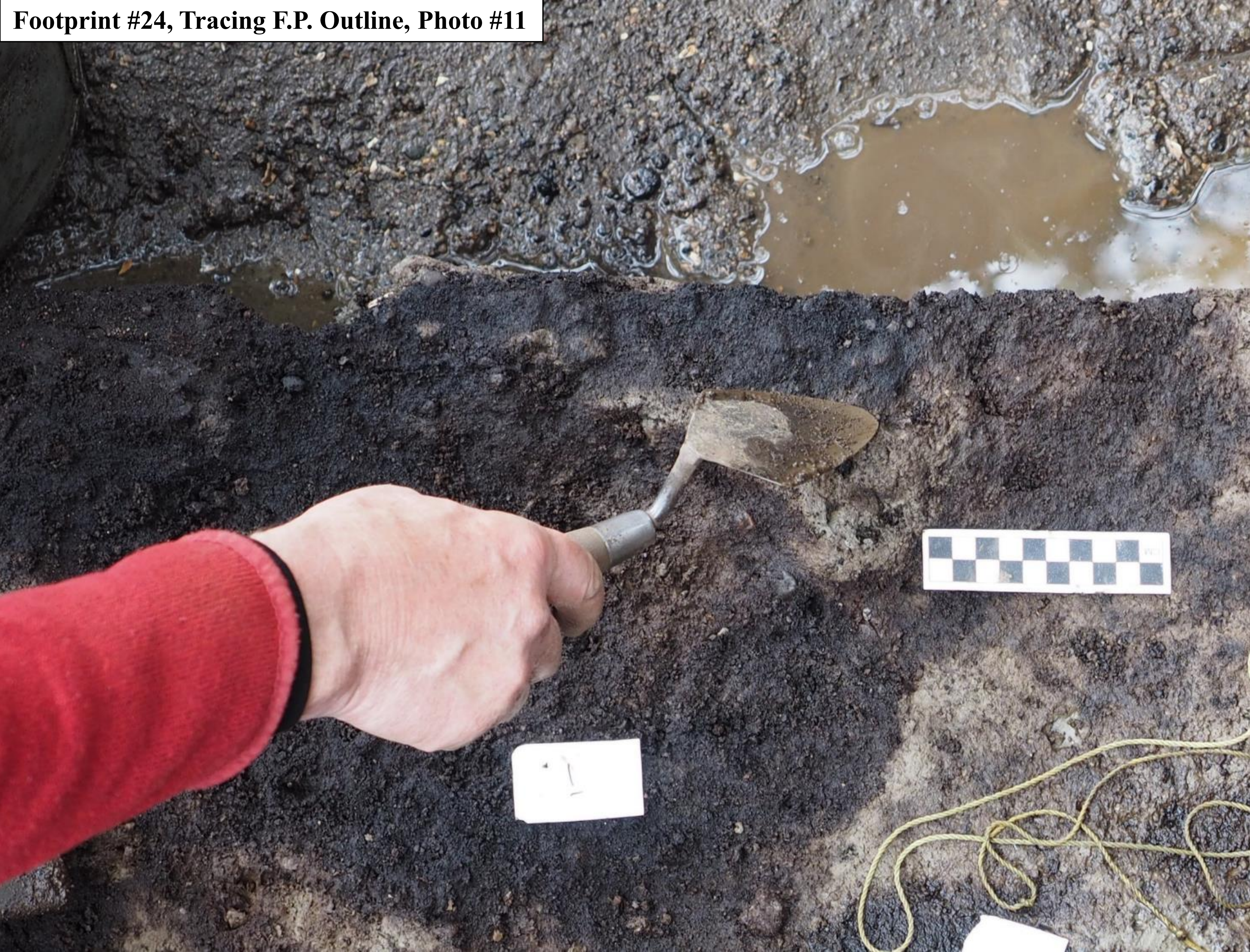

Footprint #24, Tracing F.P. Outline, Photo #12

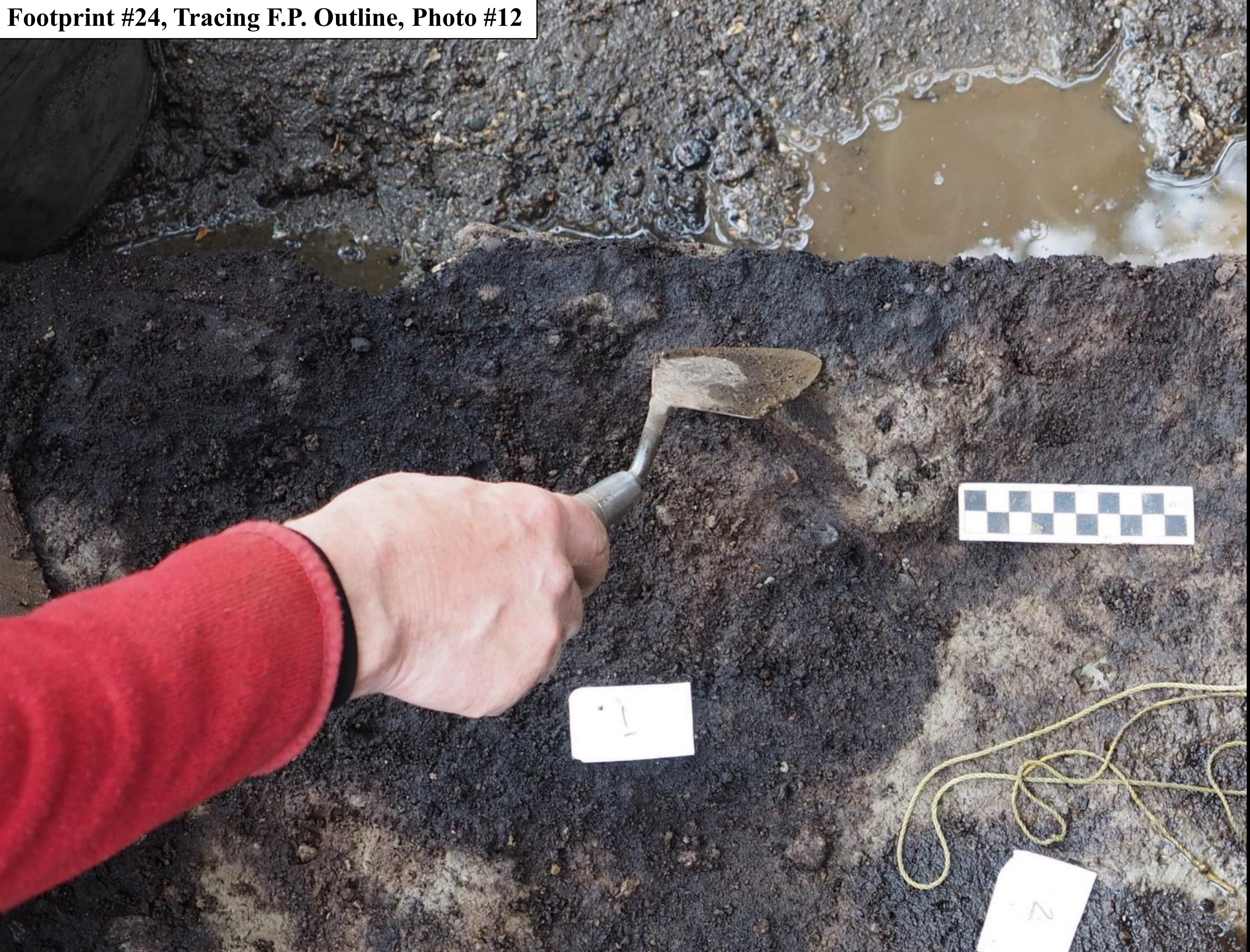

Footprint #24, Tracing F.P. Outline, Photo #13

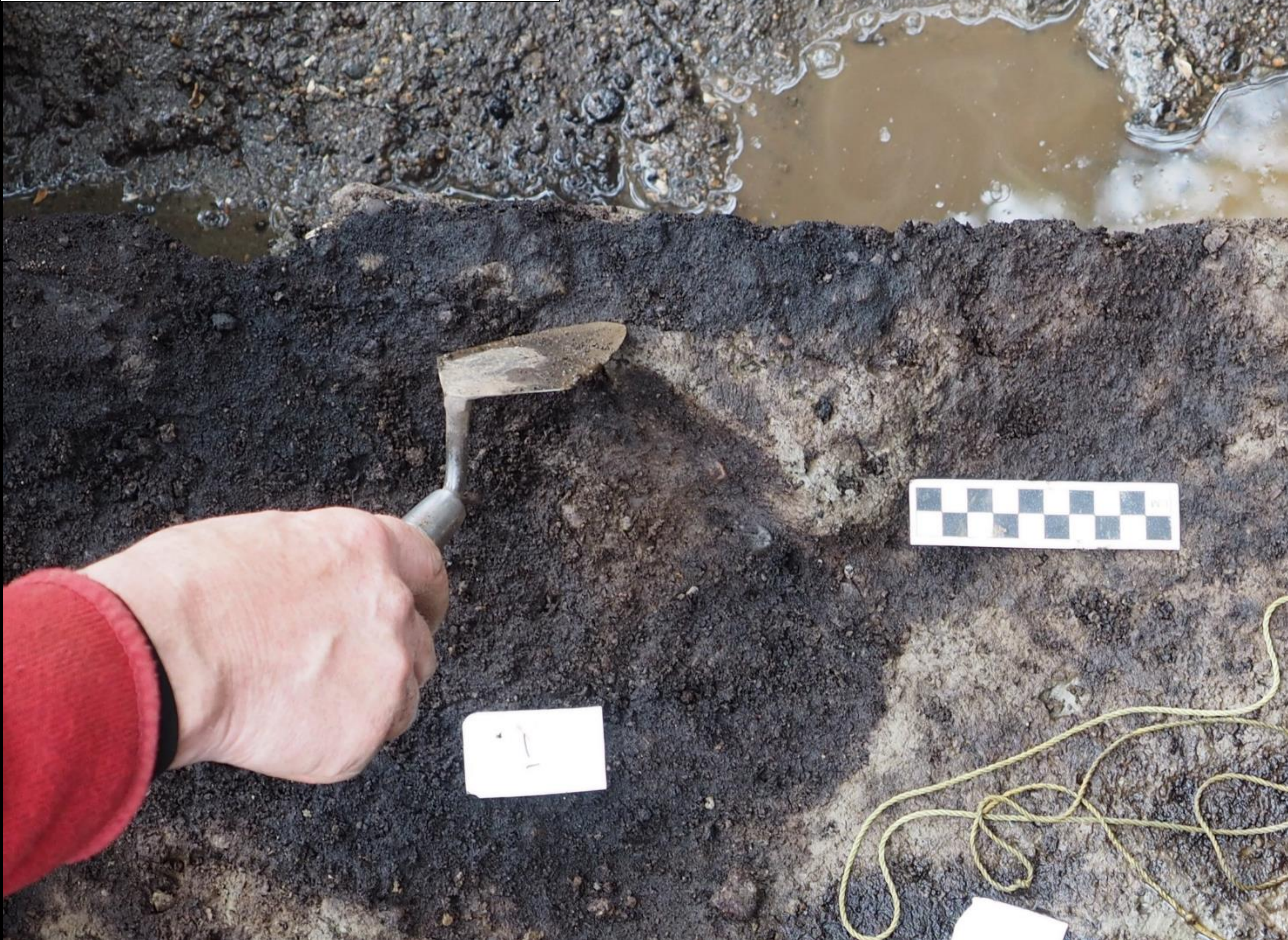

Footprint #24, Tracing F.P. Outline, Photo #14

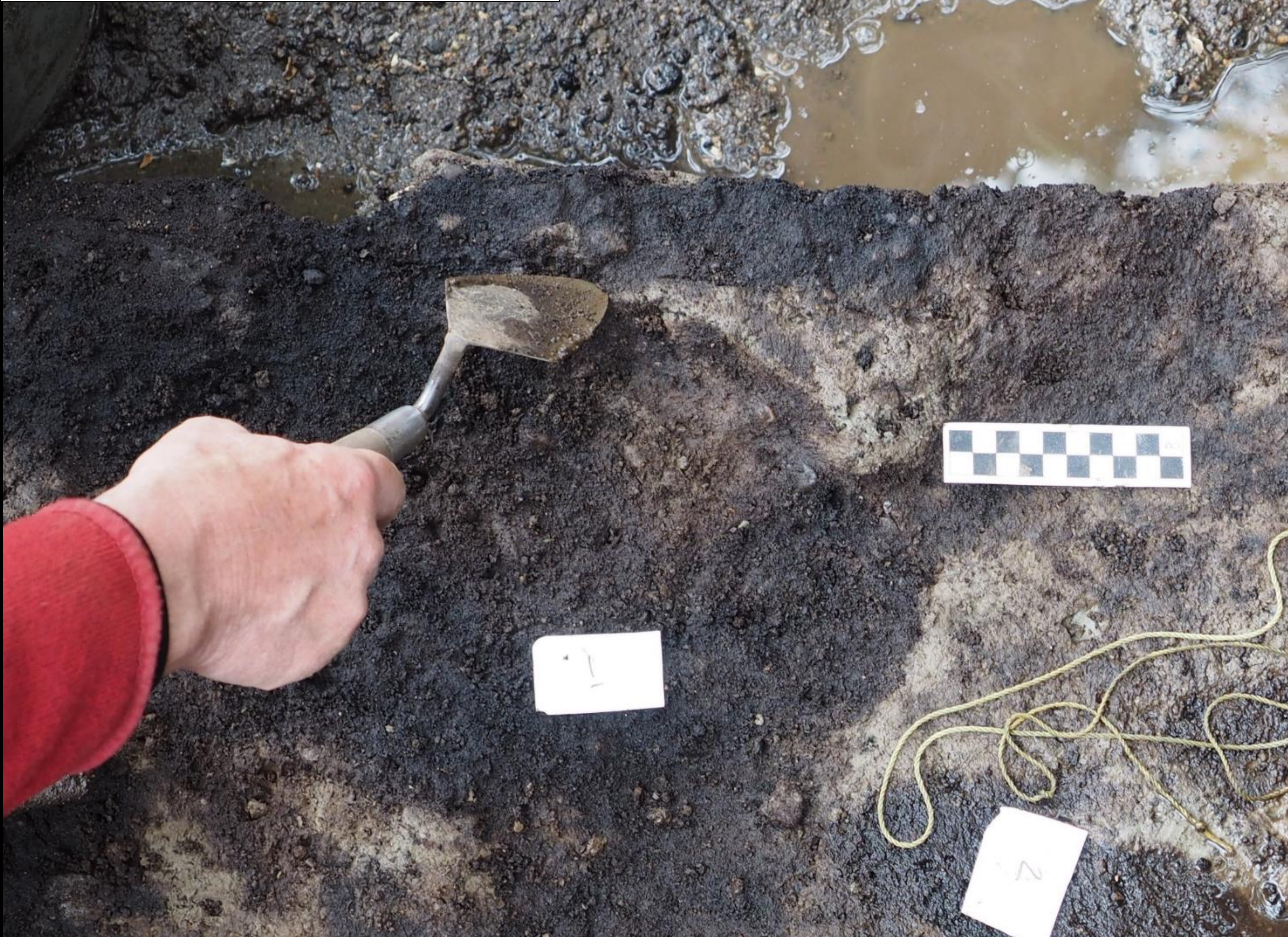

Footprint #24, Tracing F.P. Outline, Photo #15

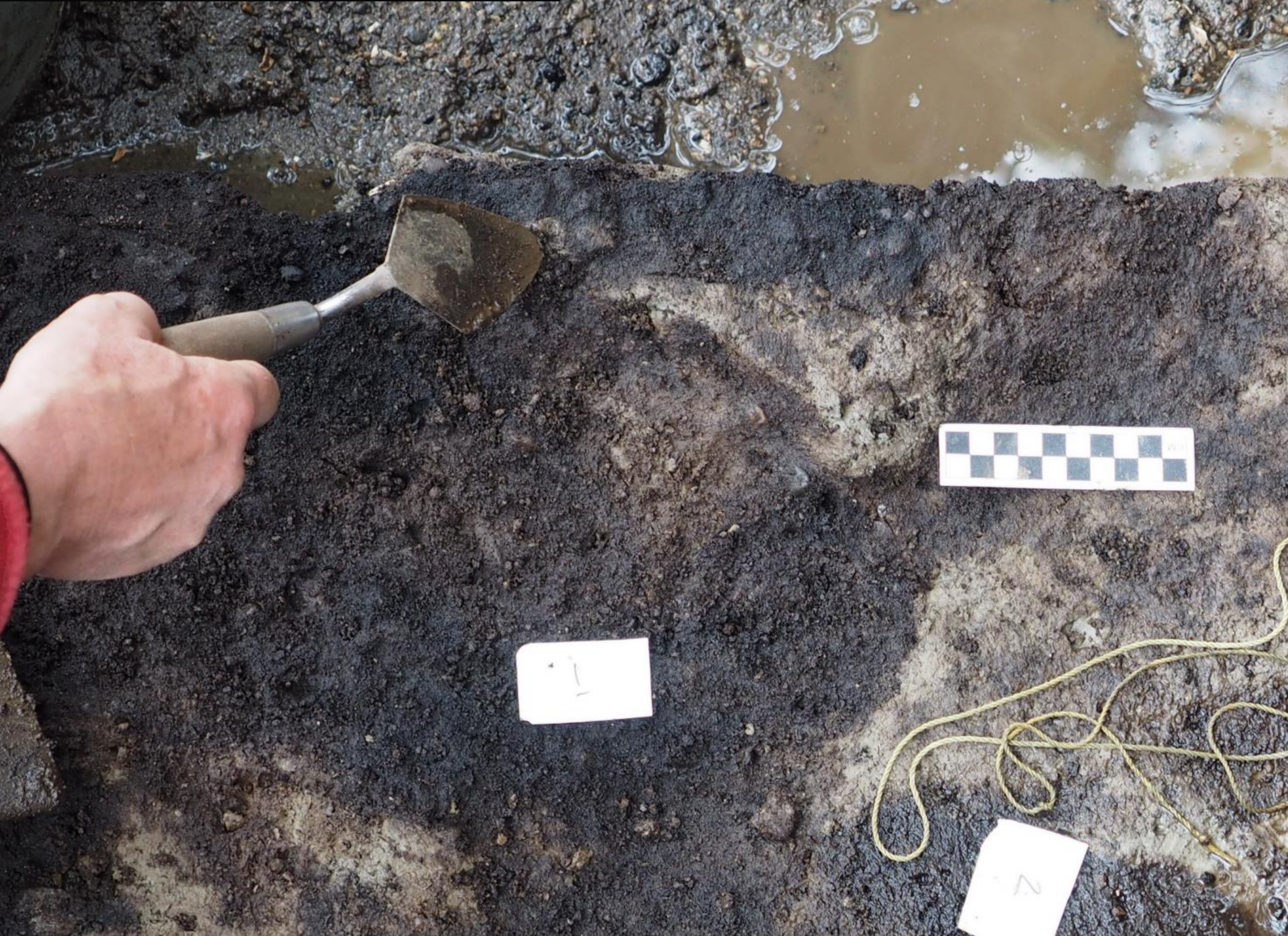

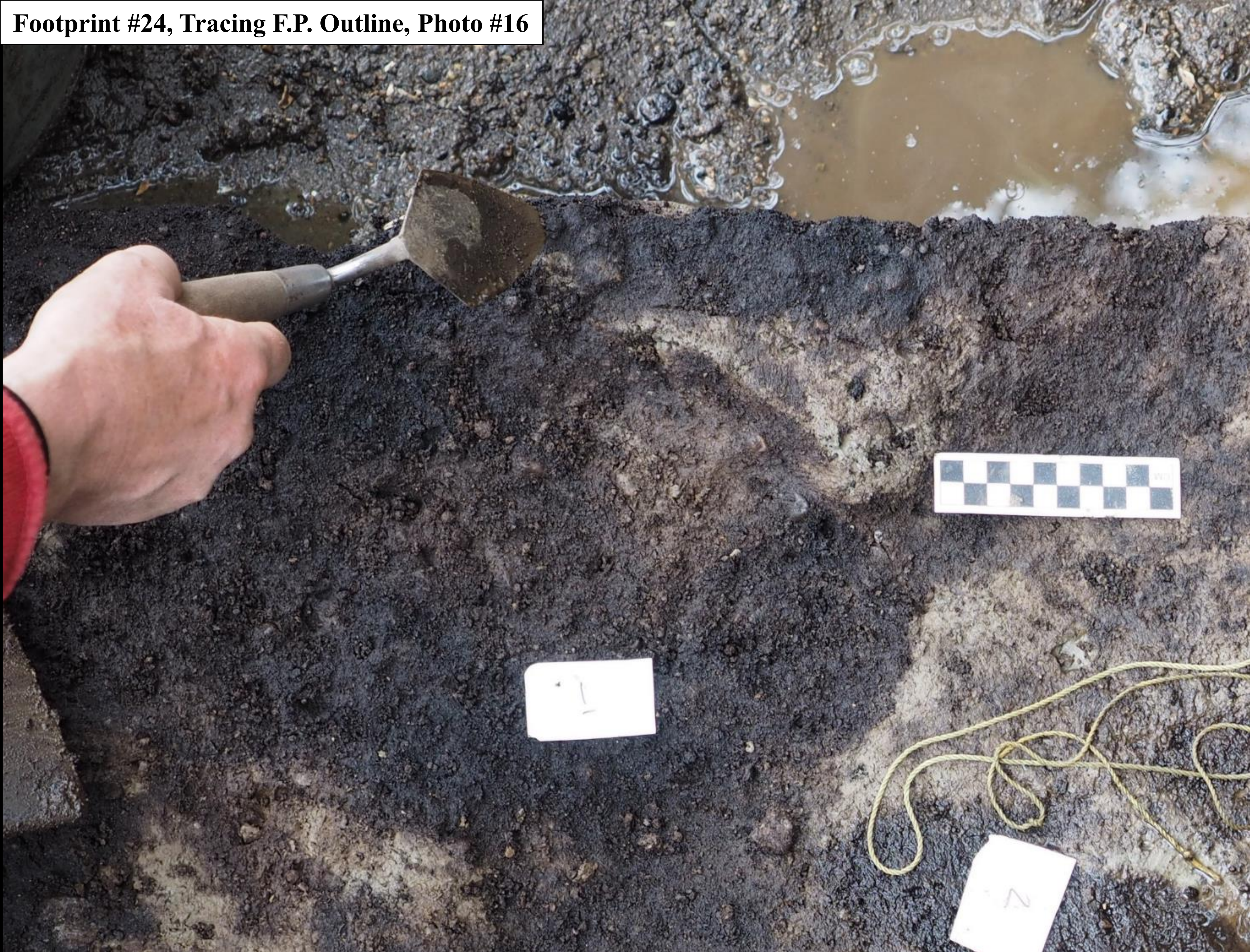

Footprint #24, Tracing F.P. Outline, Photo #16

Footprint #25, Very Slipped, Photo #1

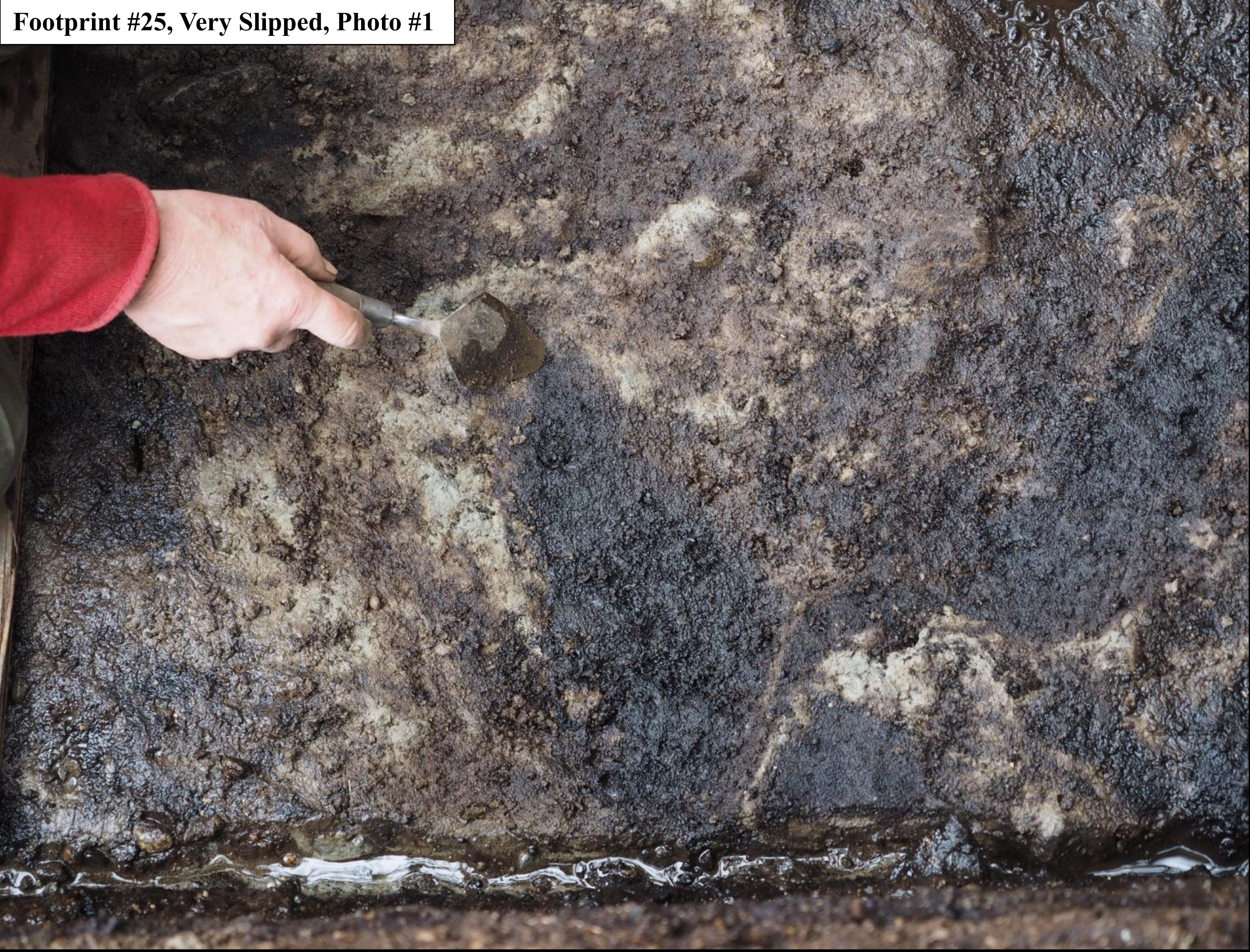

Footprint #25, Very Slipped-Dstretch, Photo #2

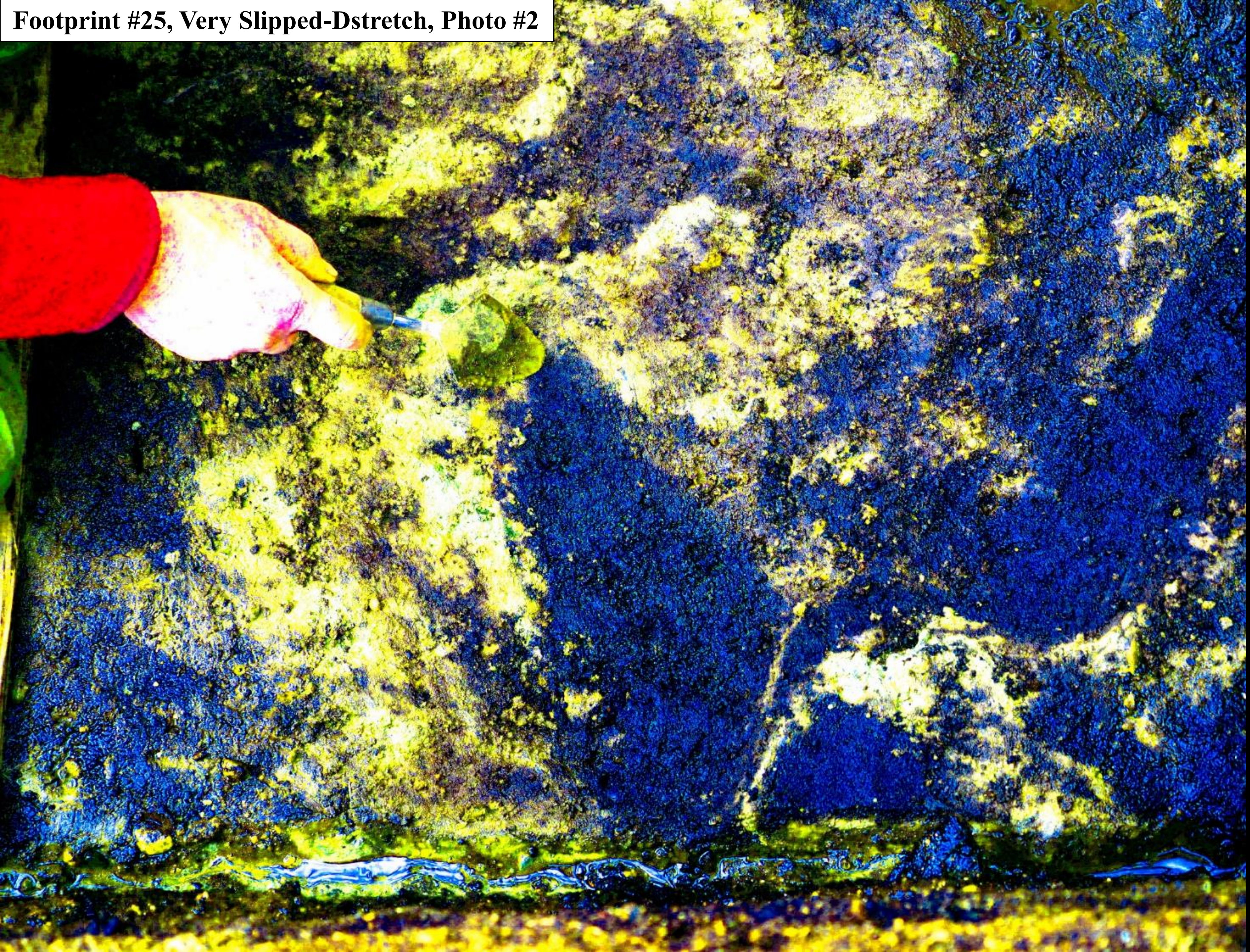

Footprint #27, Photo #1

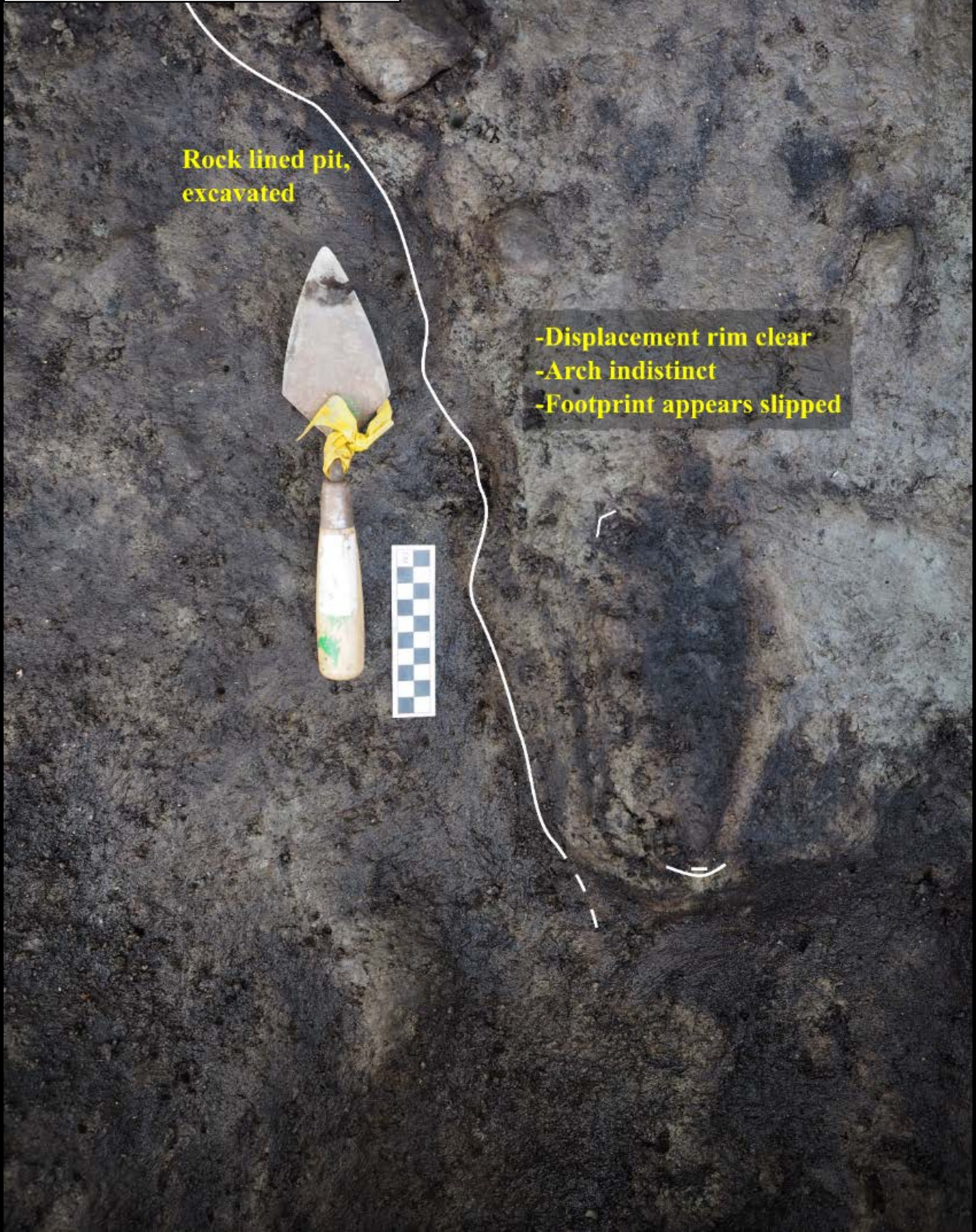

Footprint #27, Photo #2

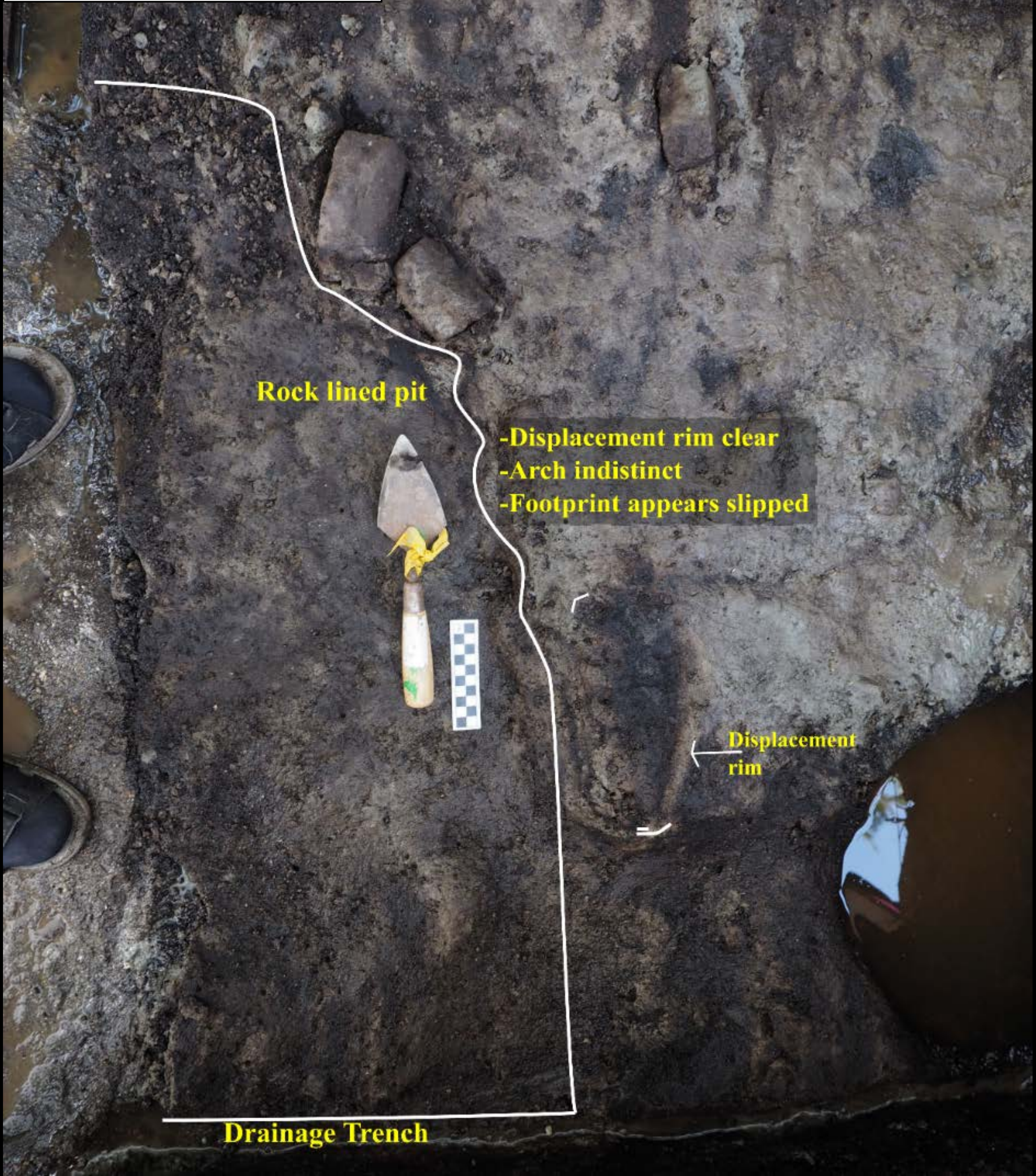

Footprint #27, Photo #3

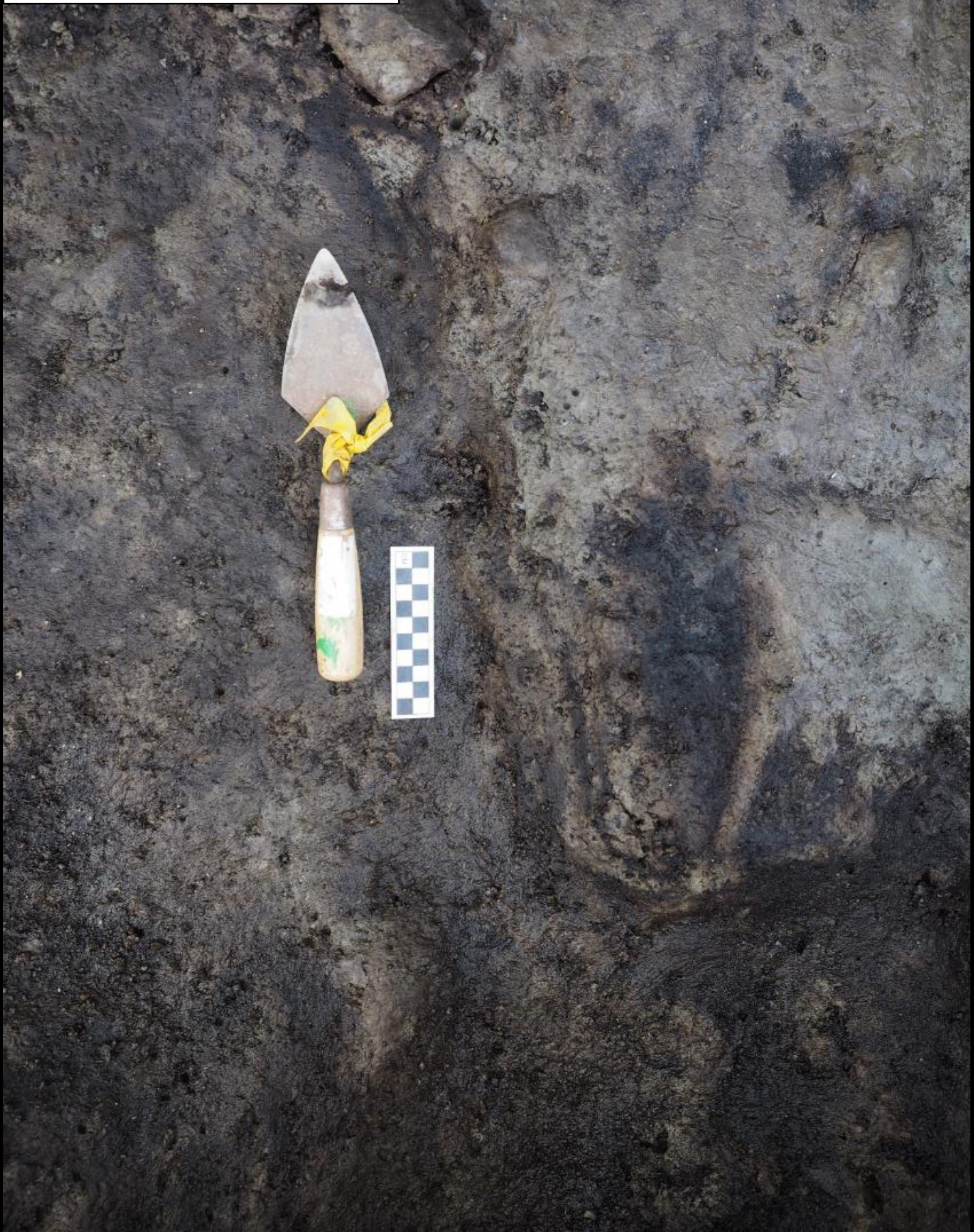

Footprint #27, DStretch, Photo #4

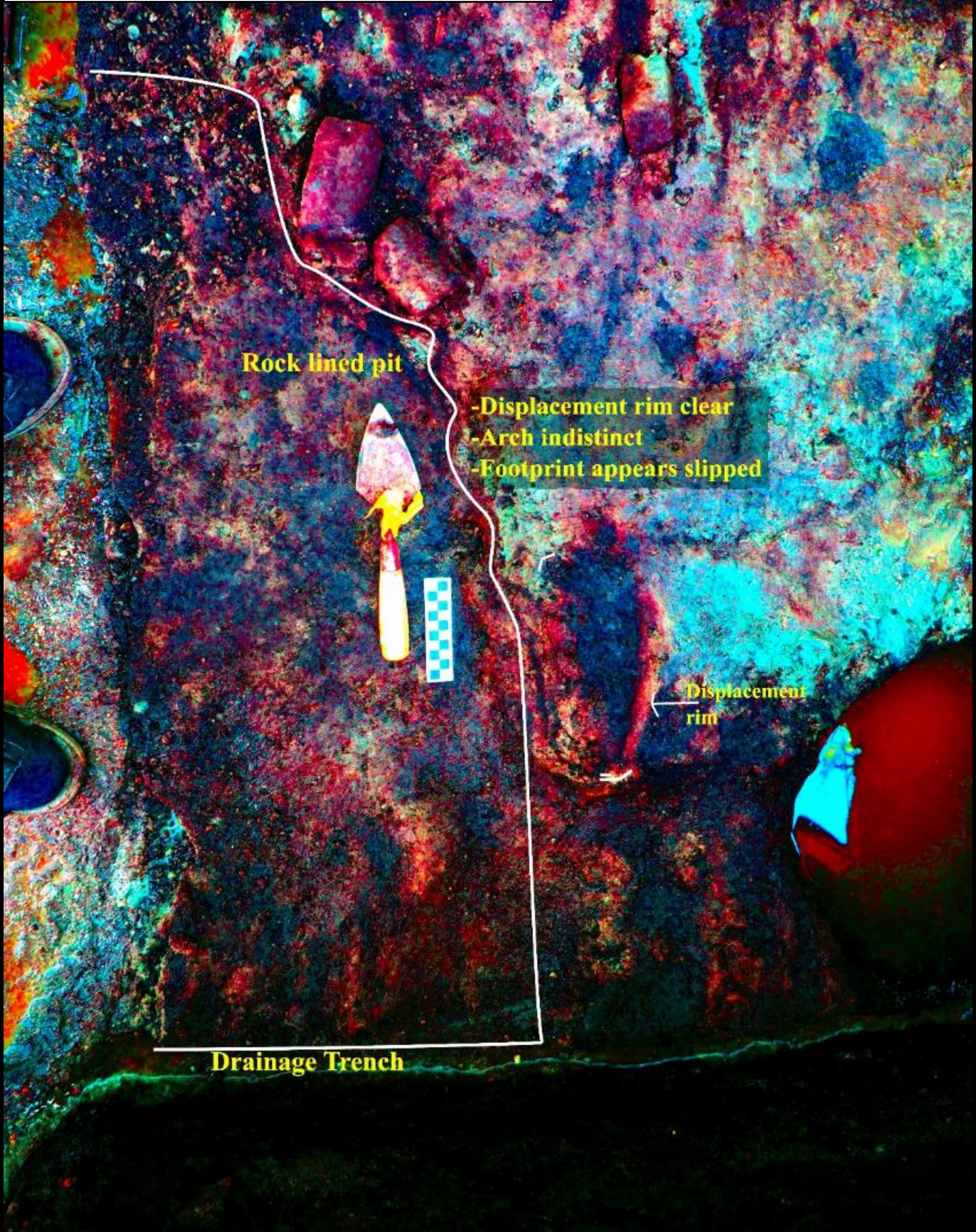

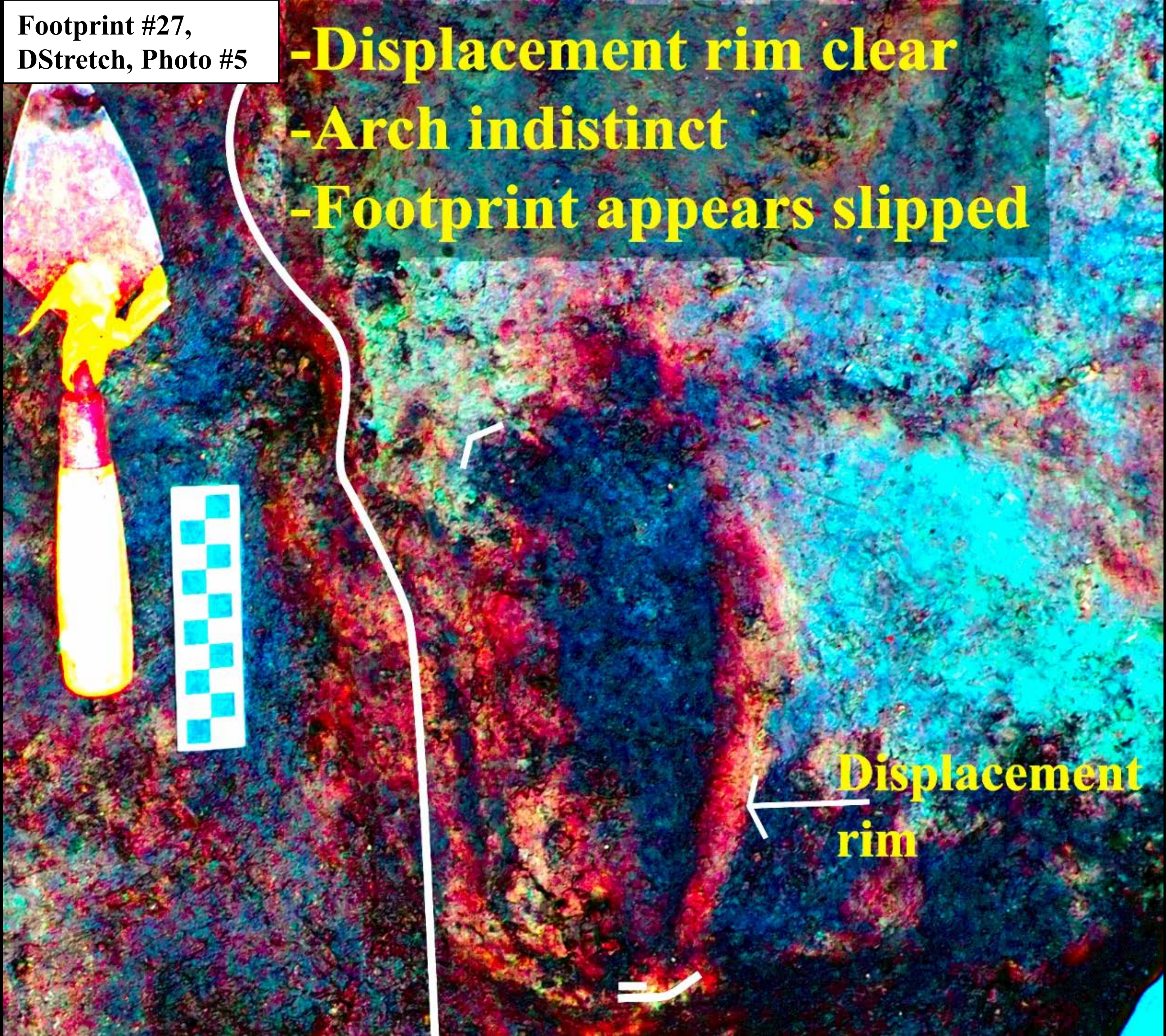
Footprint #27,
DStretch, Photo #5
-Displacement rim clear
-Arch indistinct
-Footprint appears slipped
Displacement
rim

Footprint #13, Photo #1

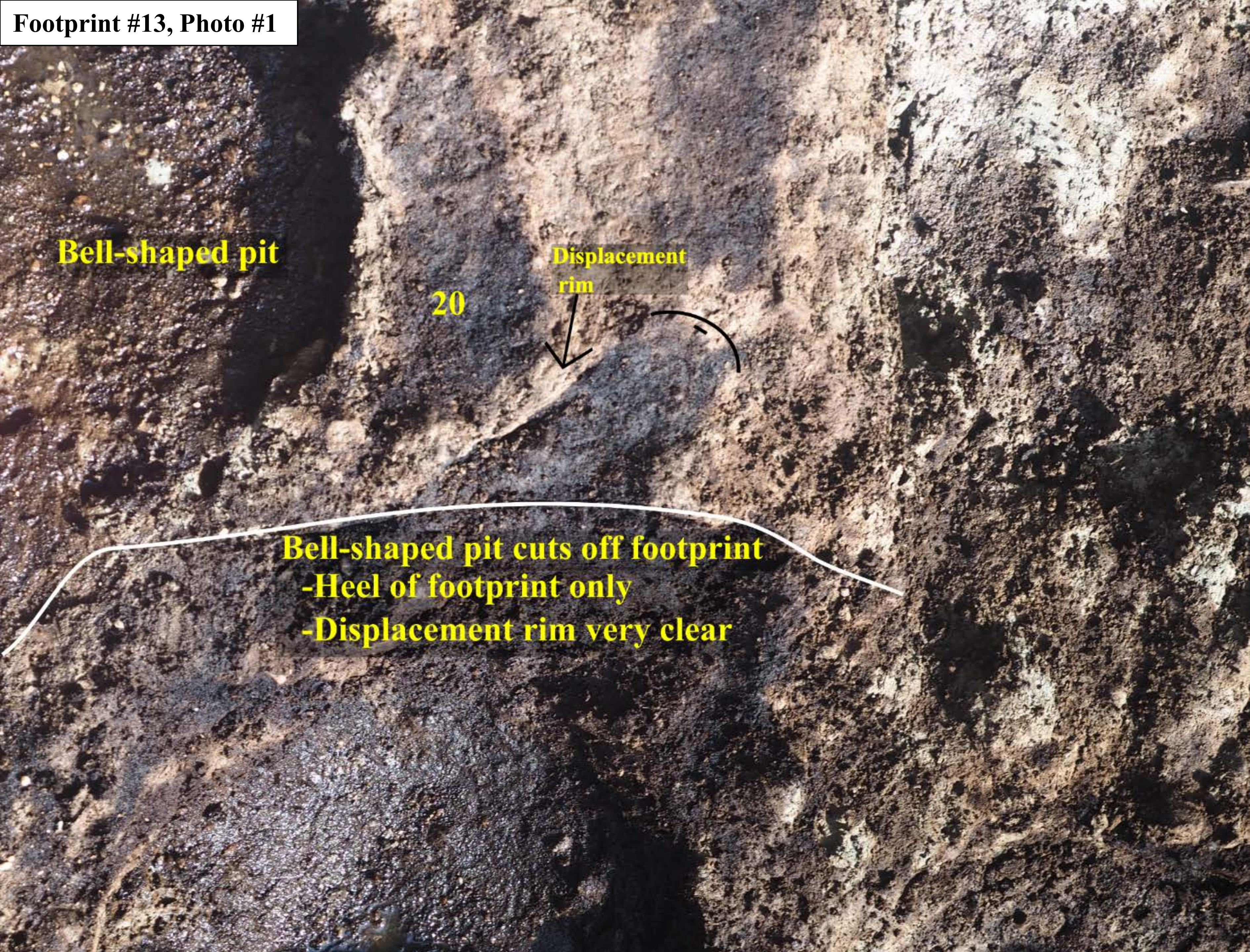

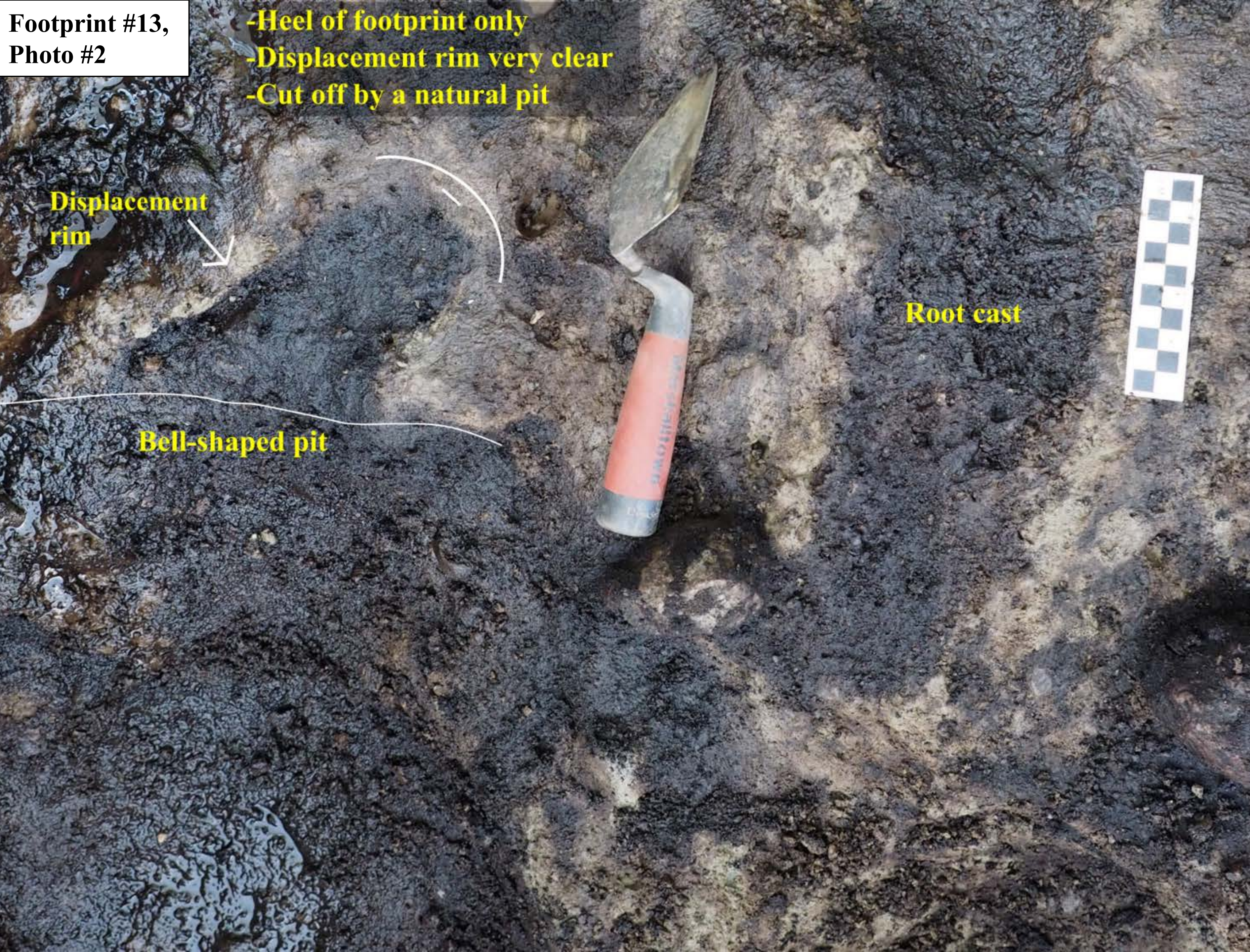

Footprint #13, Photo #2

Footprint #13, DStretch, Photo #3

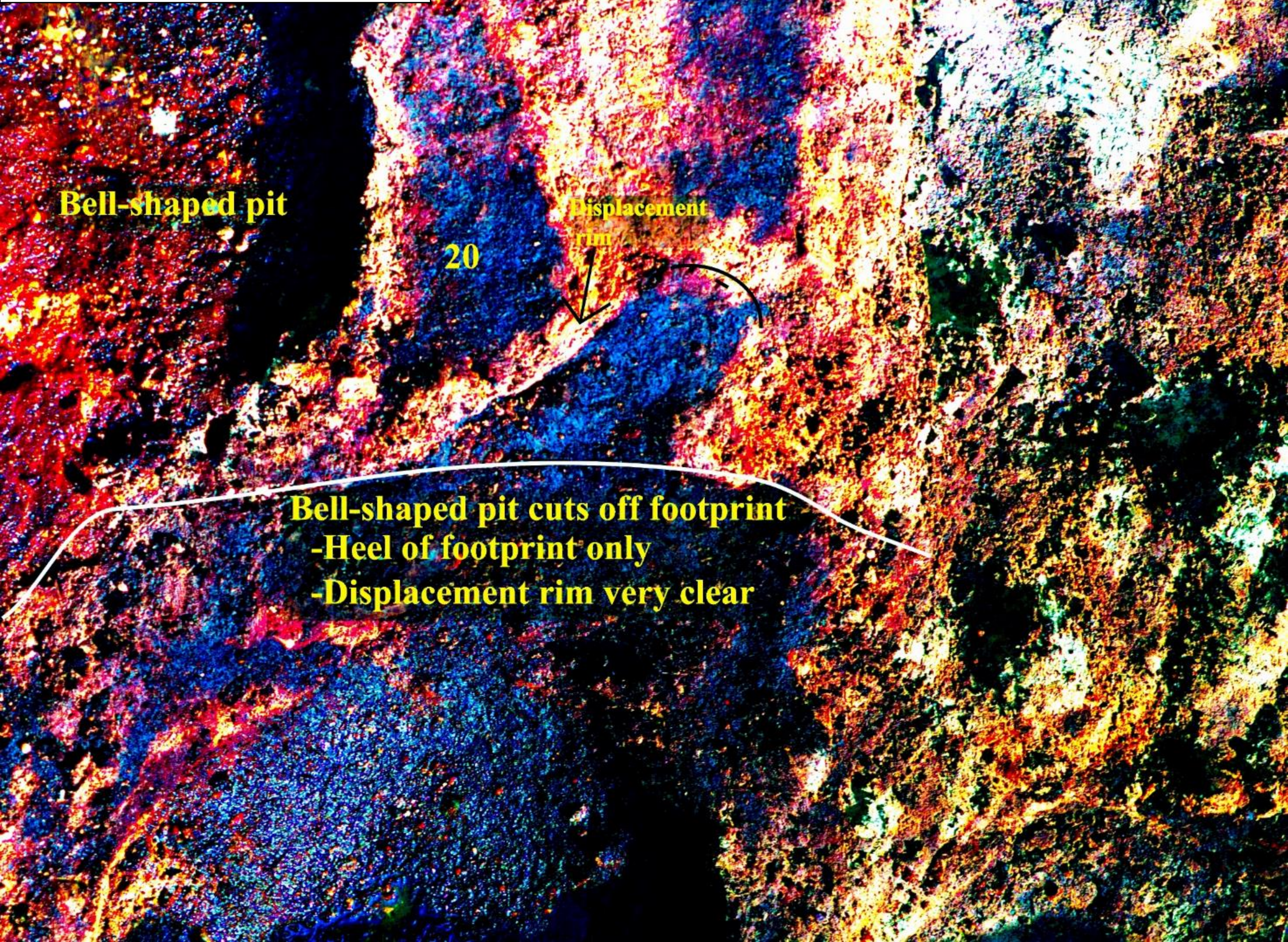

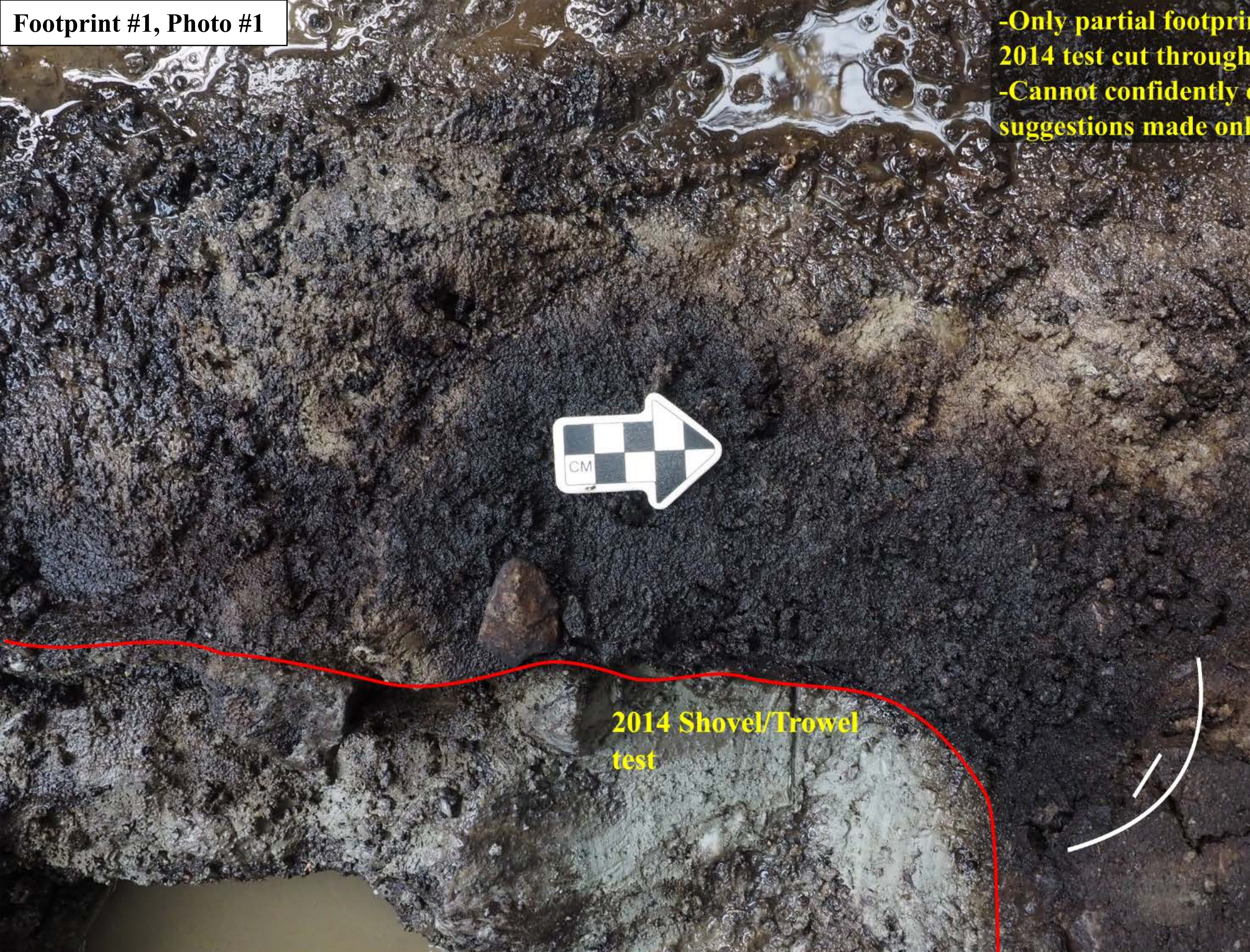
Footprint #1, Photo #1
-Only partial footpri
2014 test cut through
-Cannot confidently
suggestions made onl
CM
2014 Shovel/Trowel
test

Footprint #1, Photo #2

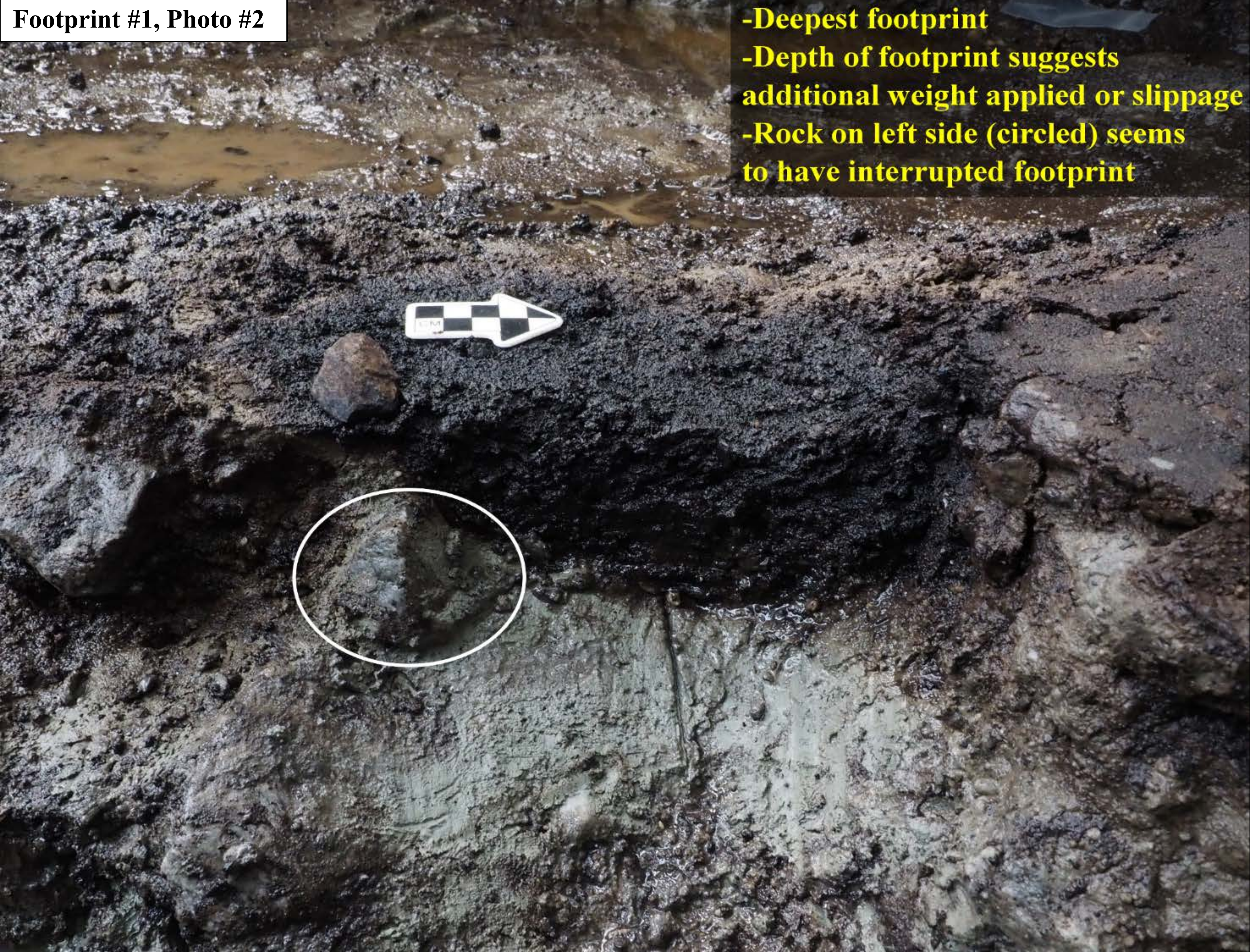

Footprint #1, Photo #3

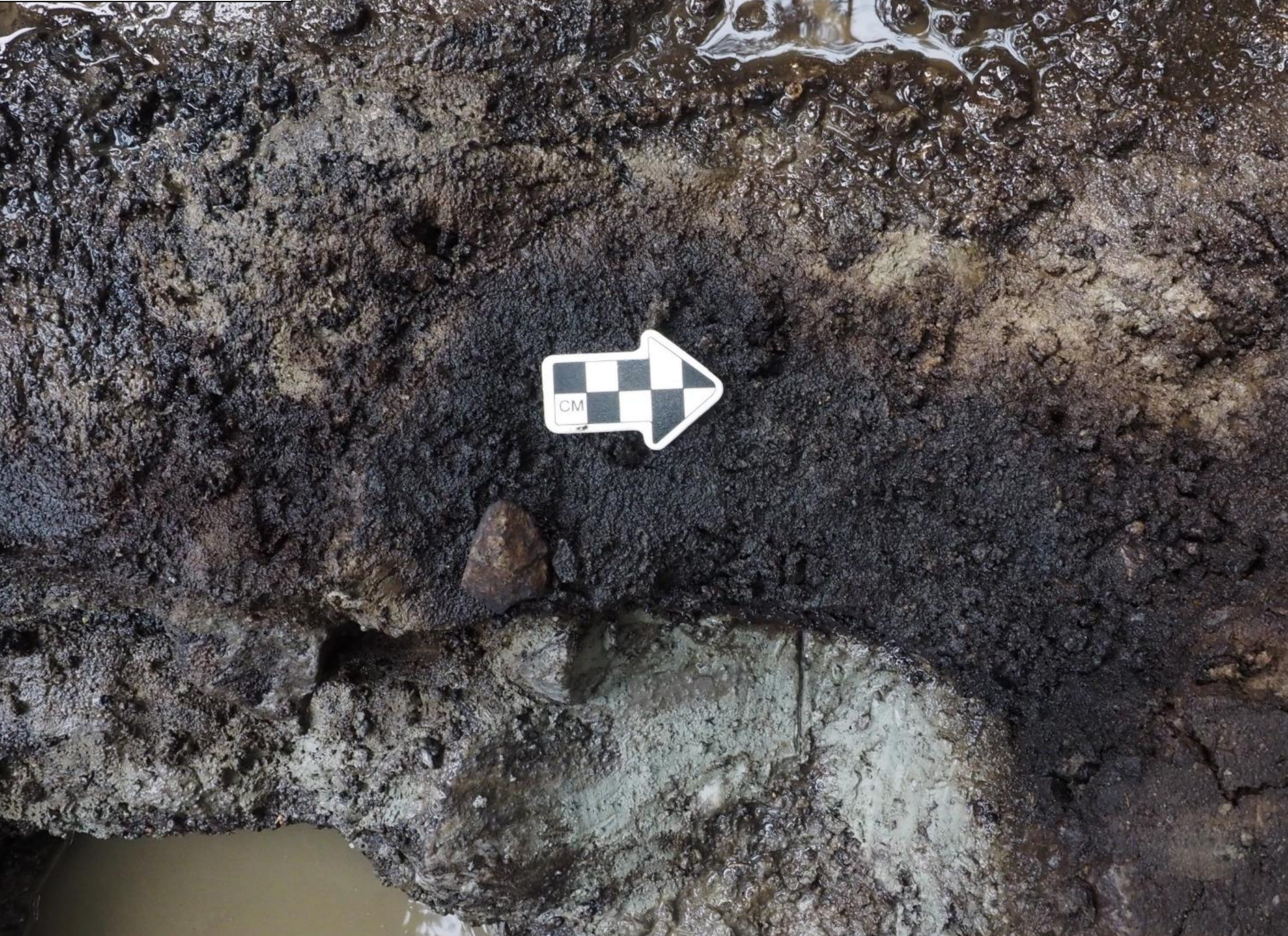

Footprint #1, DStretch, Photo #4

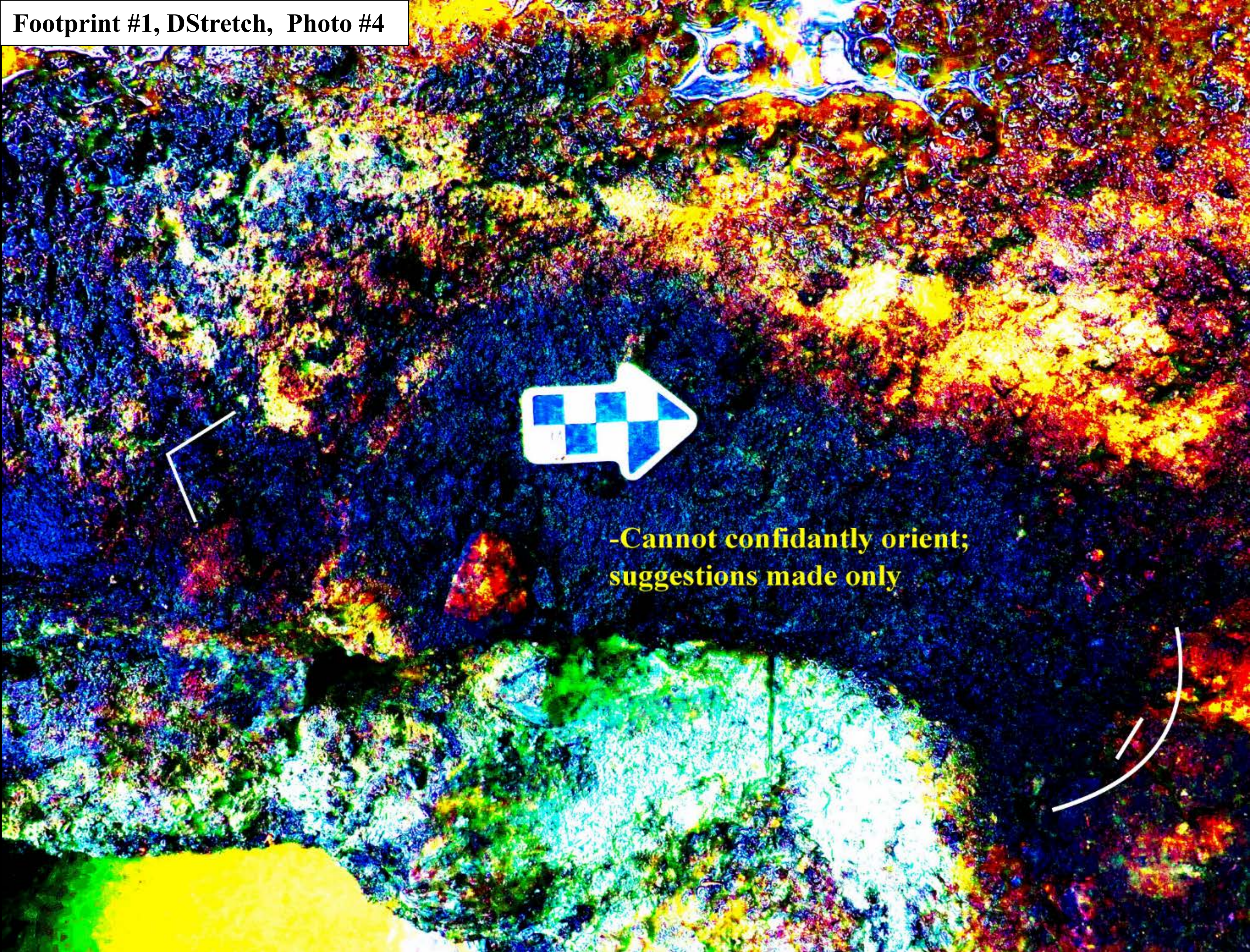

Footprint #1, DStretch, Photo #5

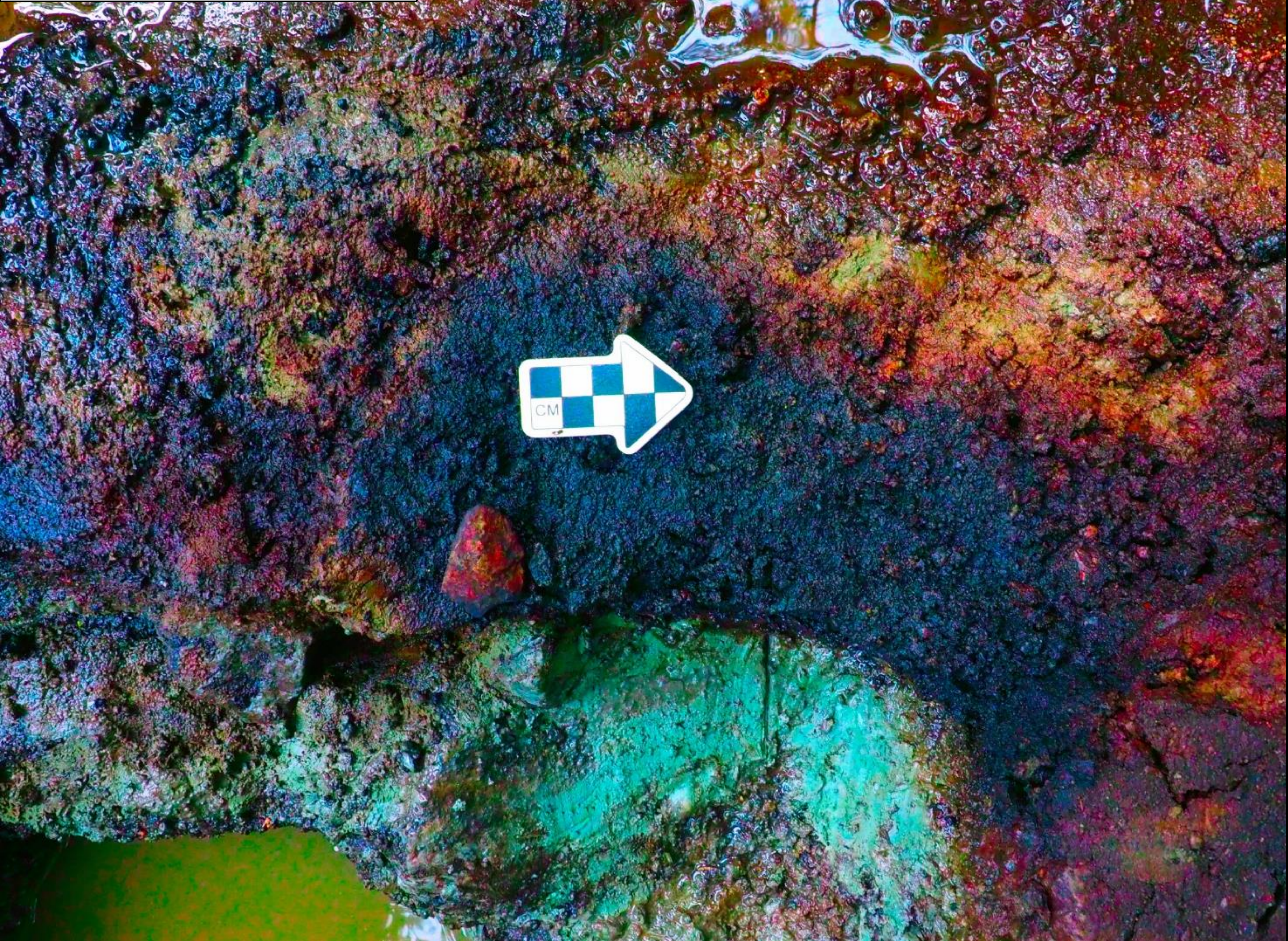

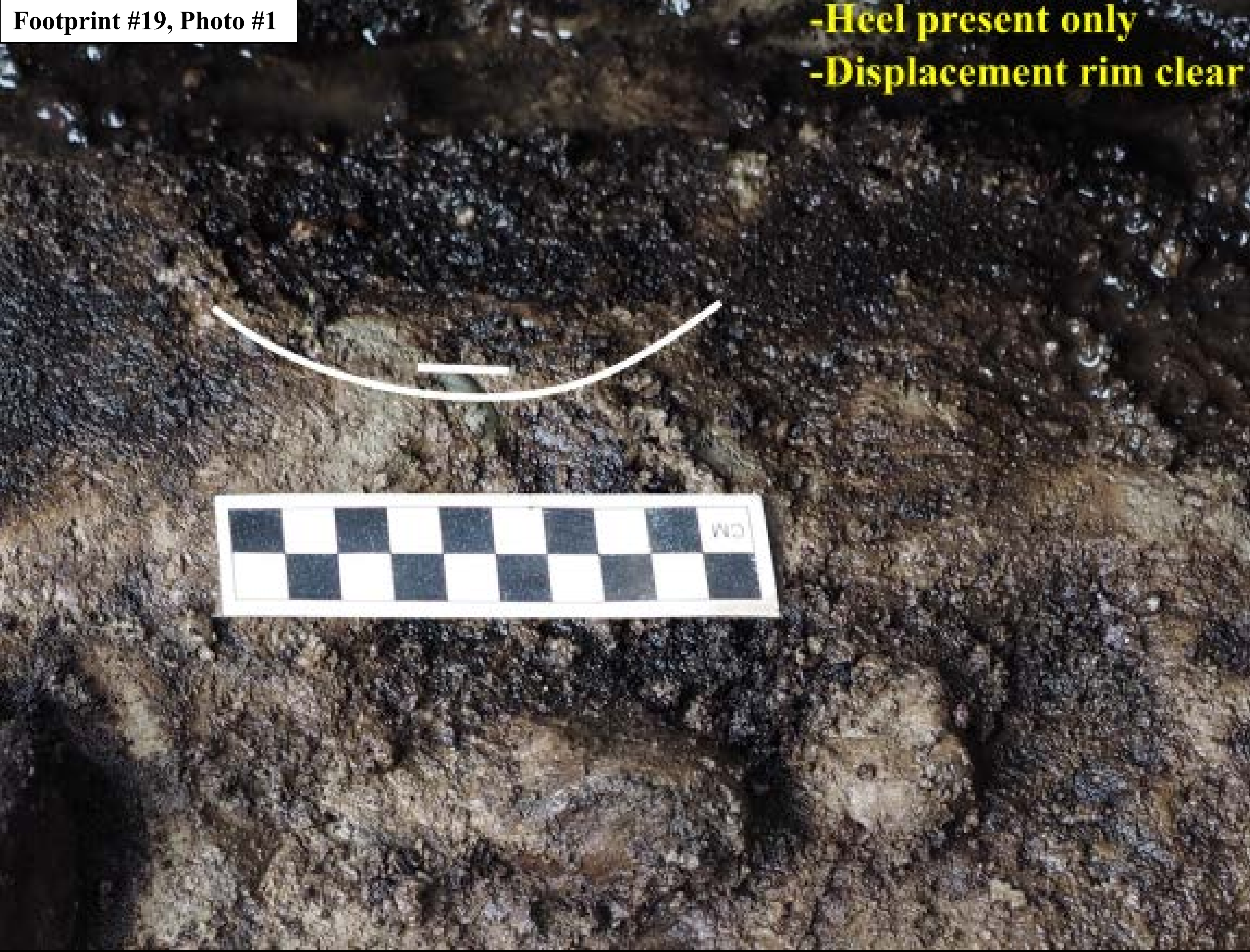
Footprint #19, Photo #1
-Heel present only
-Displacement rim clear

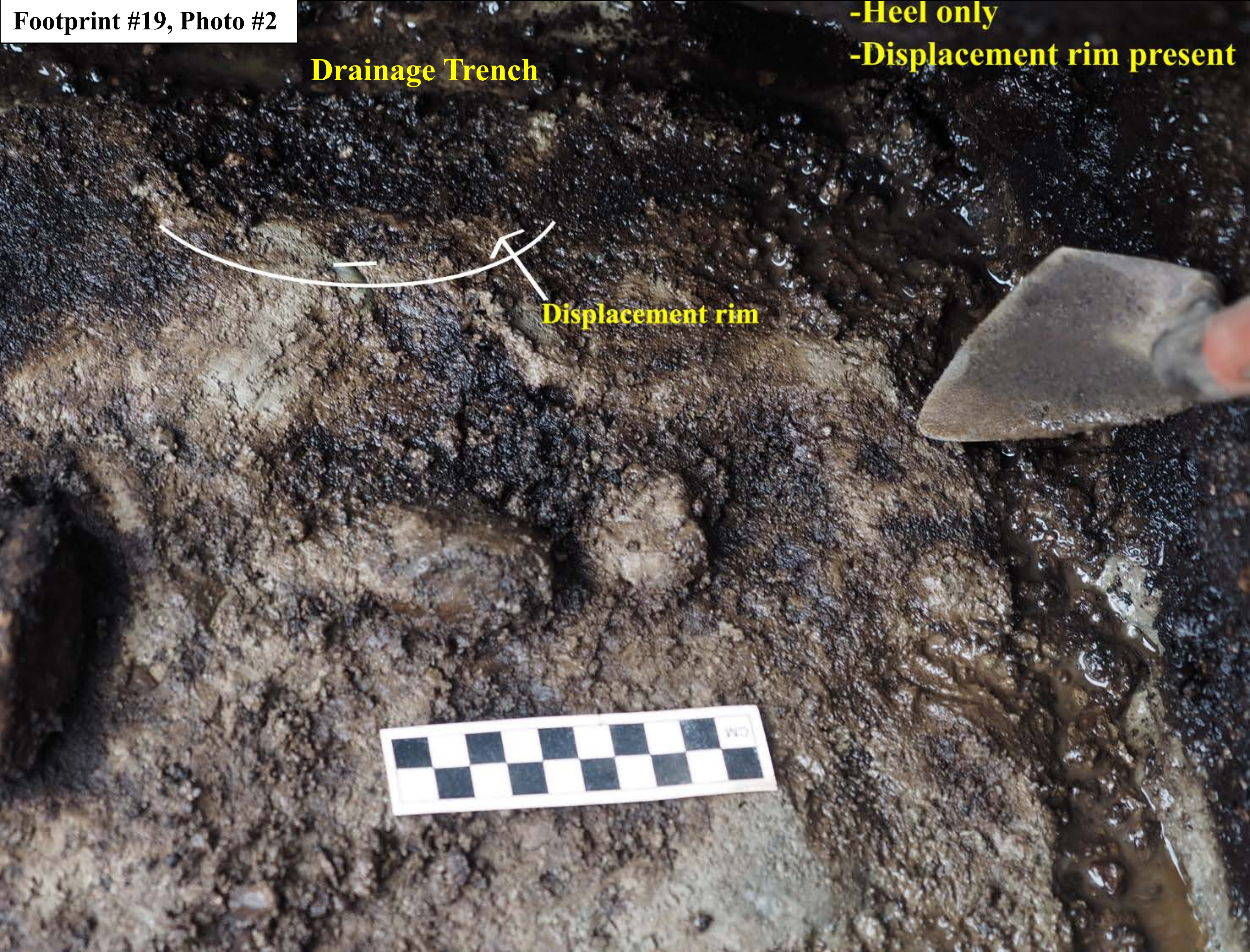
Footprint #19, Photo #2
Drainage Trench
-Heel only
-Displacement rim present
Displacement rim
CM

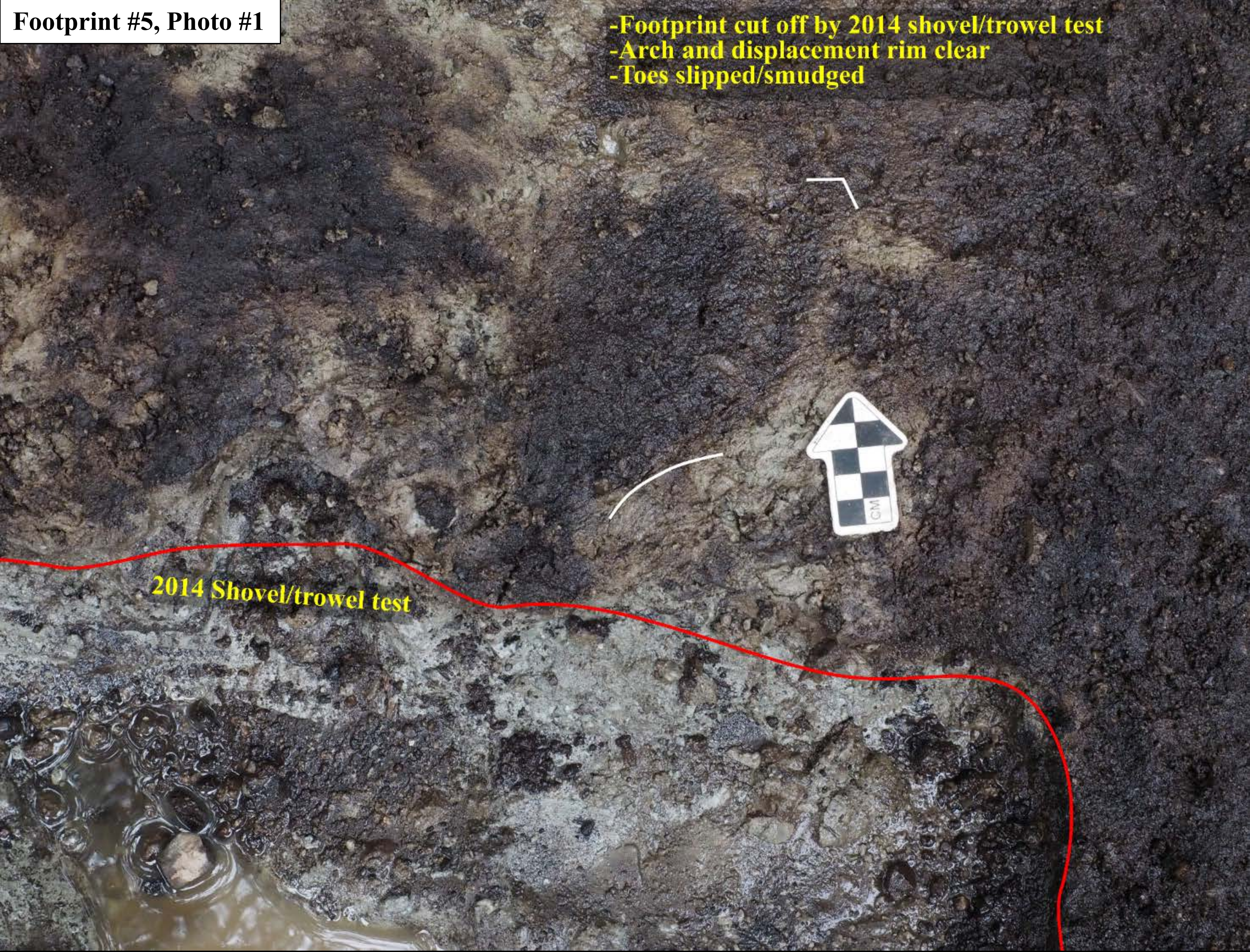
Footprint #5, Photo #1
-Footprint cut off by 2014 shovel/trowel test
-Arch and displacement rim clear
-Toes slipped/smudged
2014 Shovel/trowel test
CM

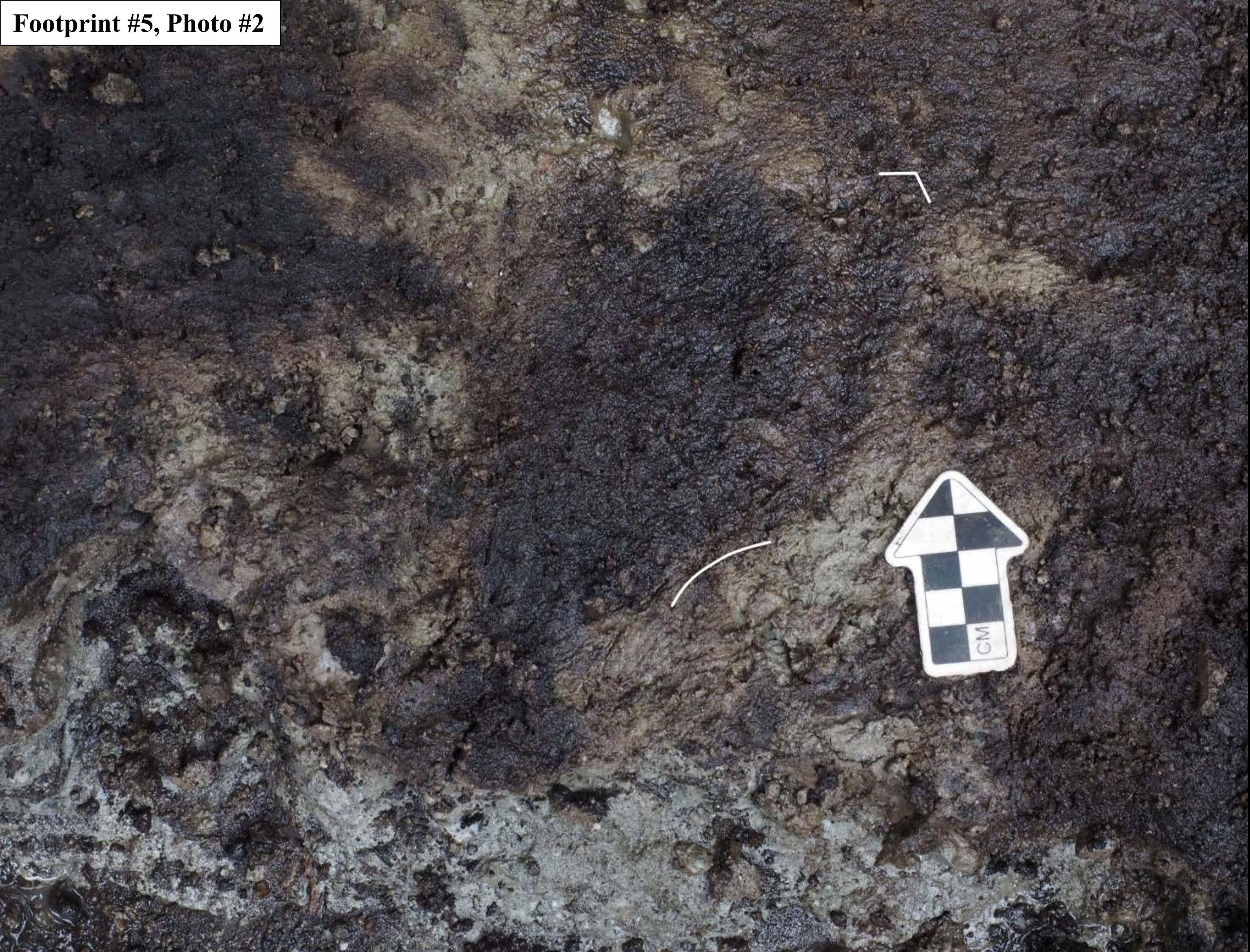

Footprint #5, Photo #2

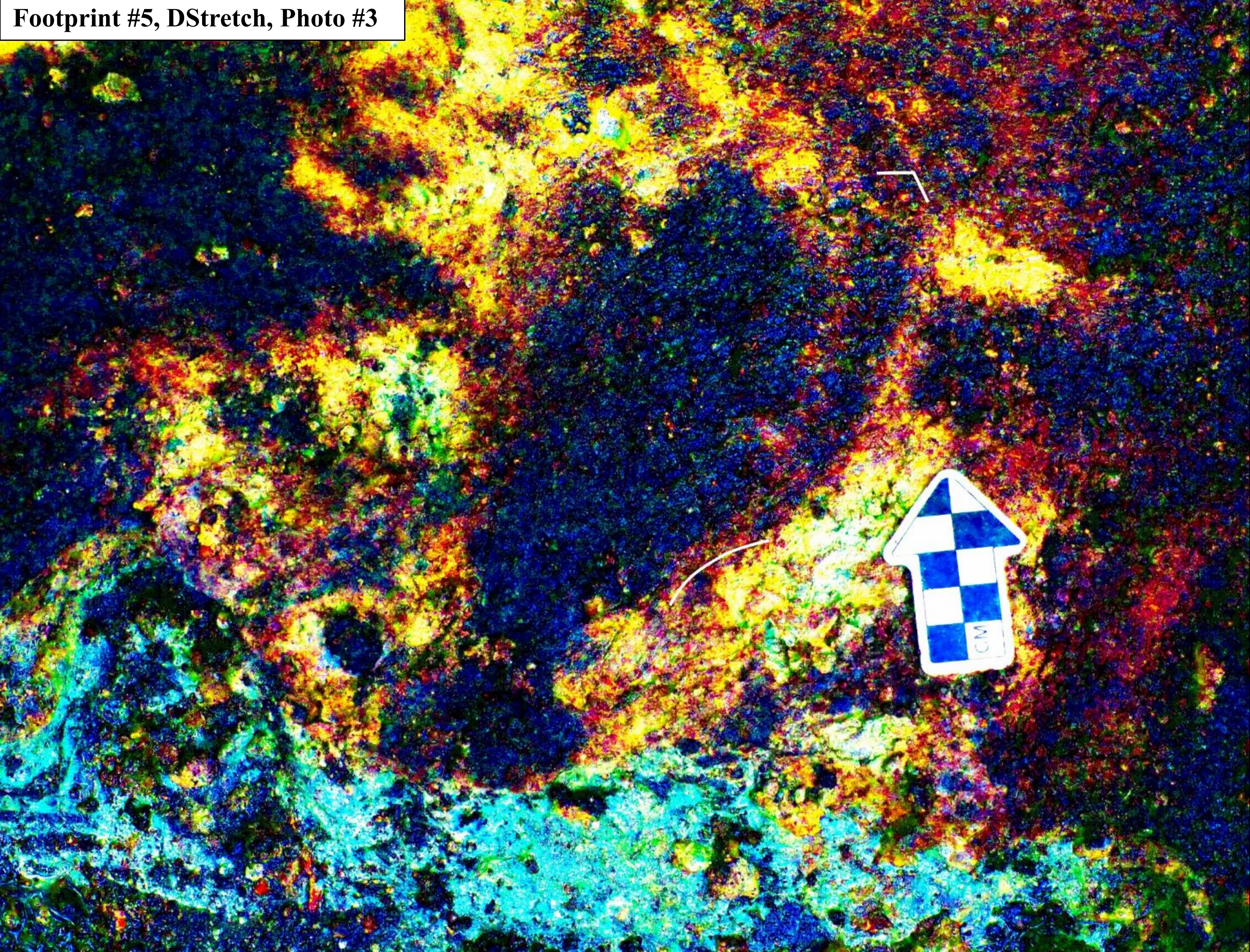

Footprint #5, DStretch, Photo #3

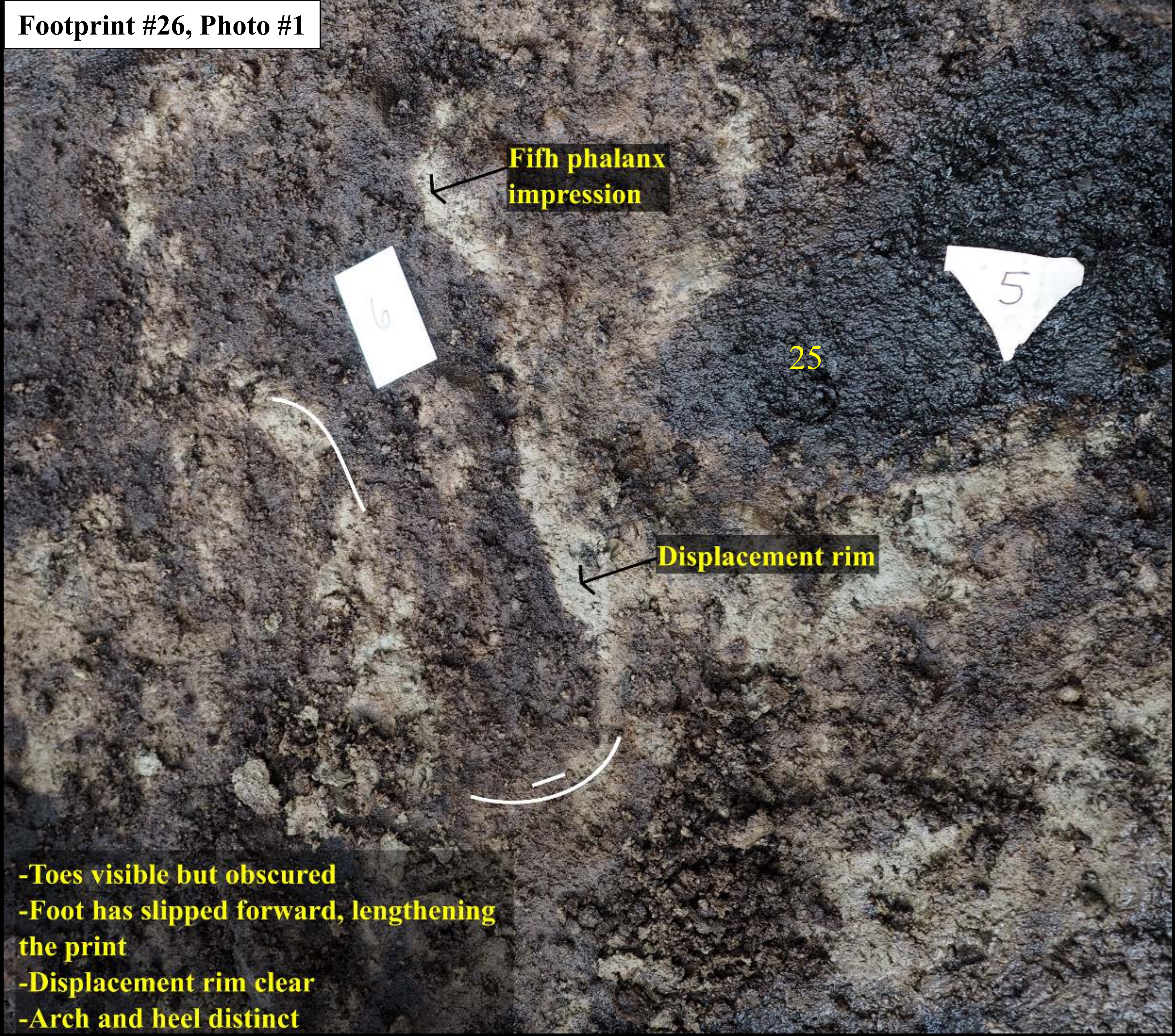
Footprint #26, Photo #1
Fifh phalanx impression
6
5
25
Displacement rim
-Toes visible but obscured
-Foot has slipped forward, lengthening the print
-Displacement rim clear
-Arch and heel distinct

Footprint #26, Photo #2

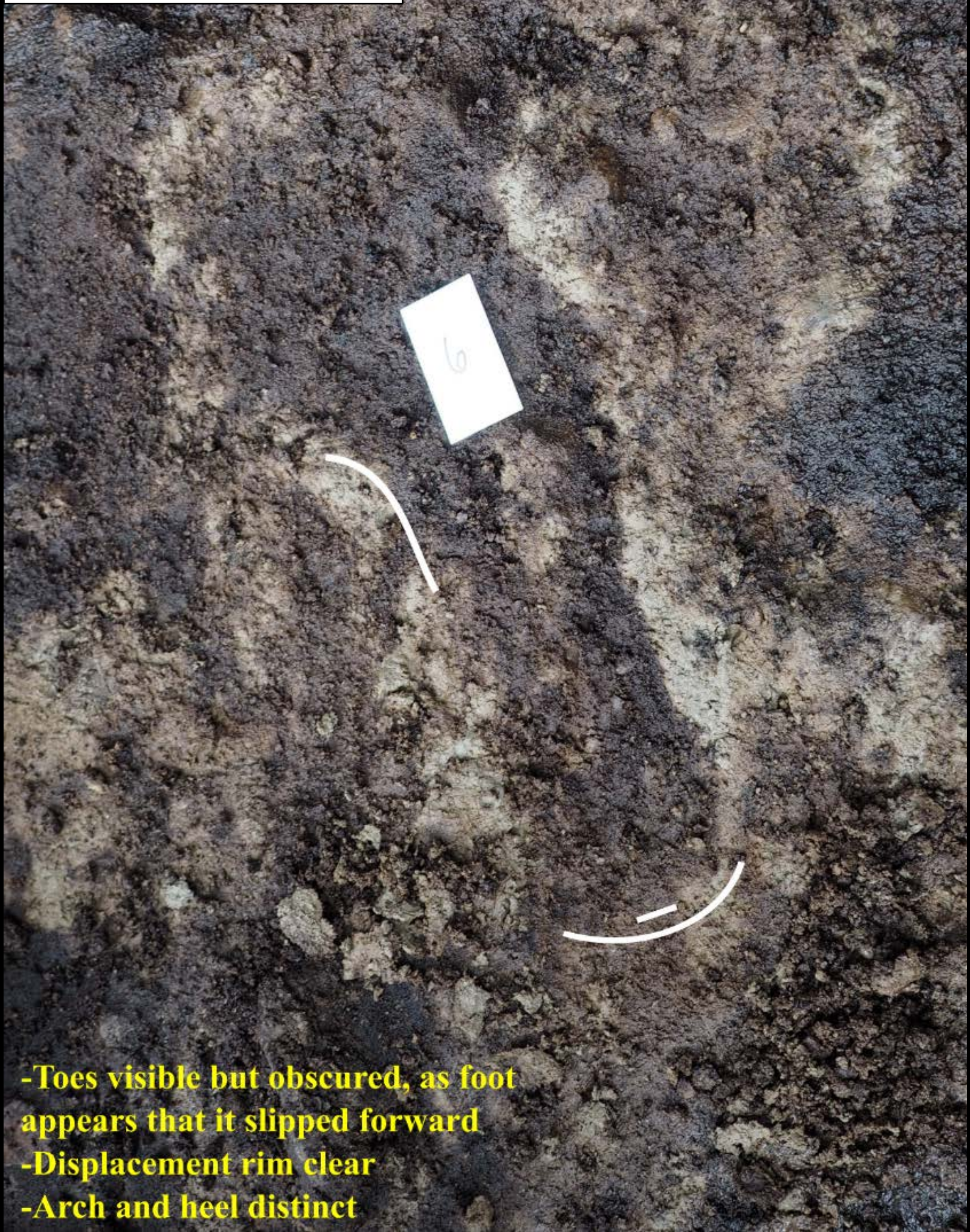

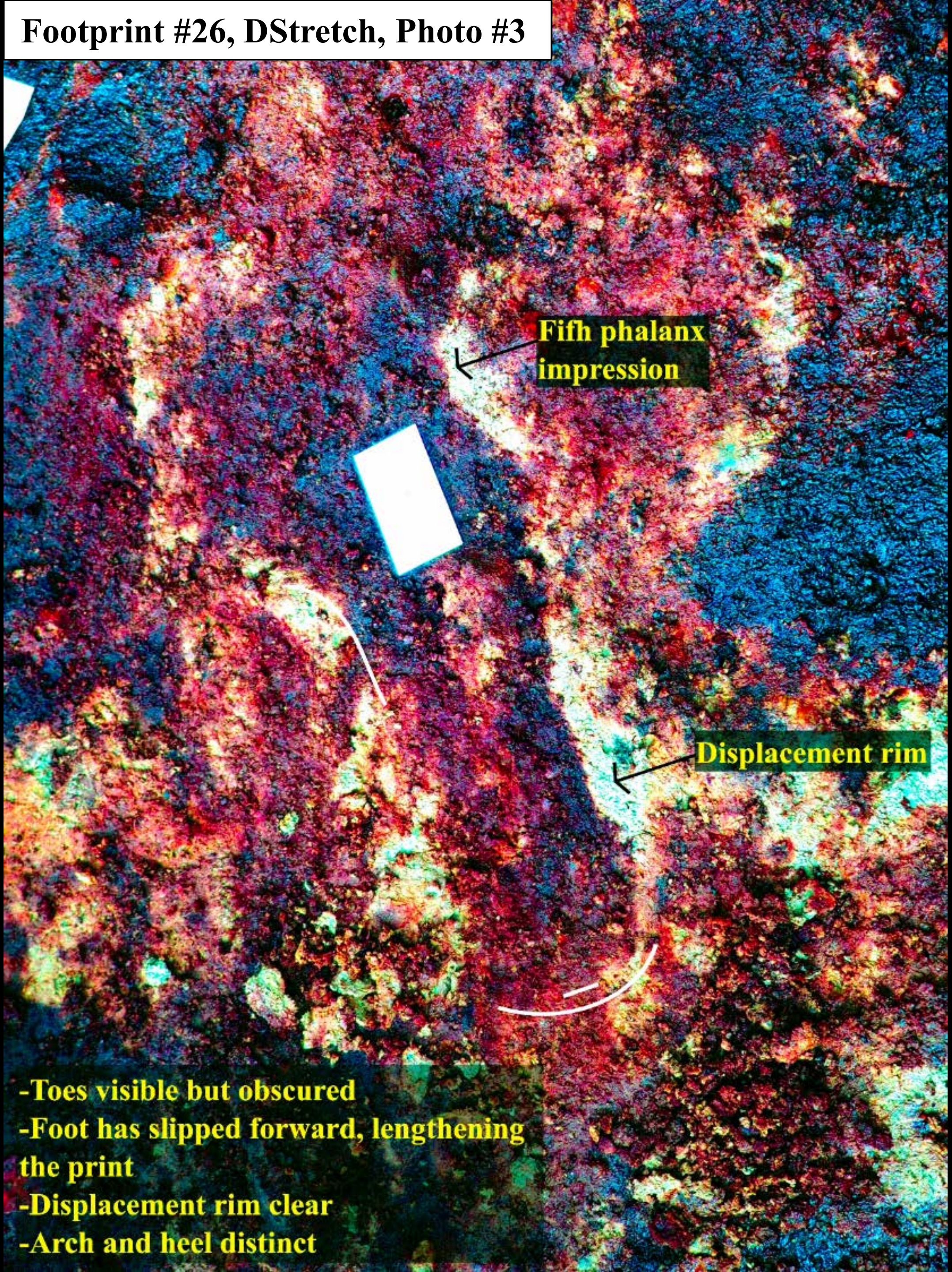
Footprint #26, DStretch, Photo #3
Fifh phalanx impression
Displacement rim
-Toes visible but obscured
-Foot has slipped forward, lengthening the print
-Displacement rim clear
-Arch and heel distinct

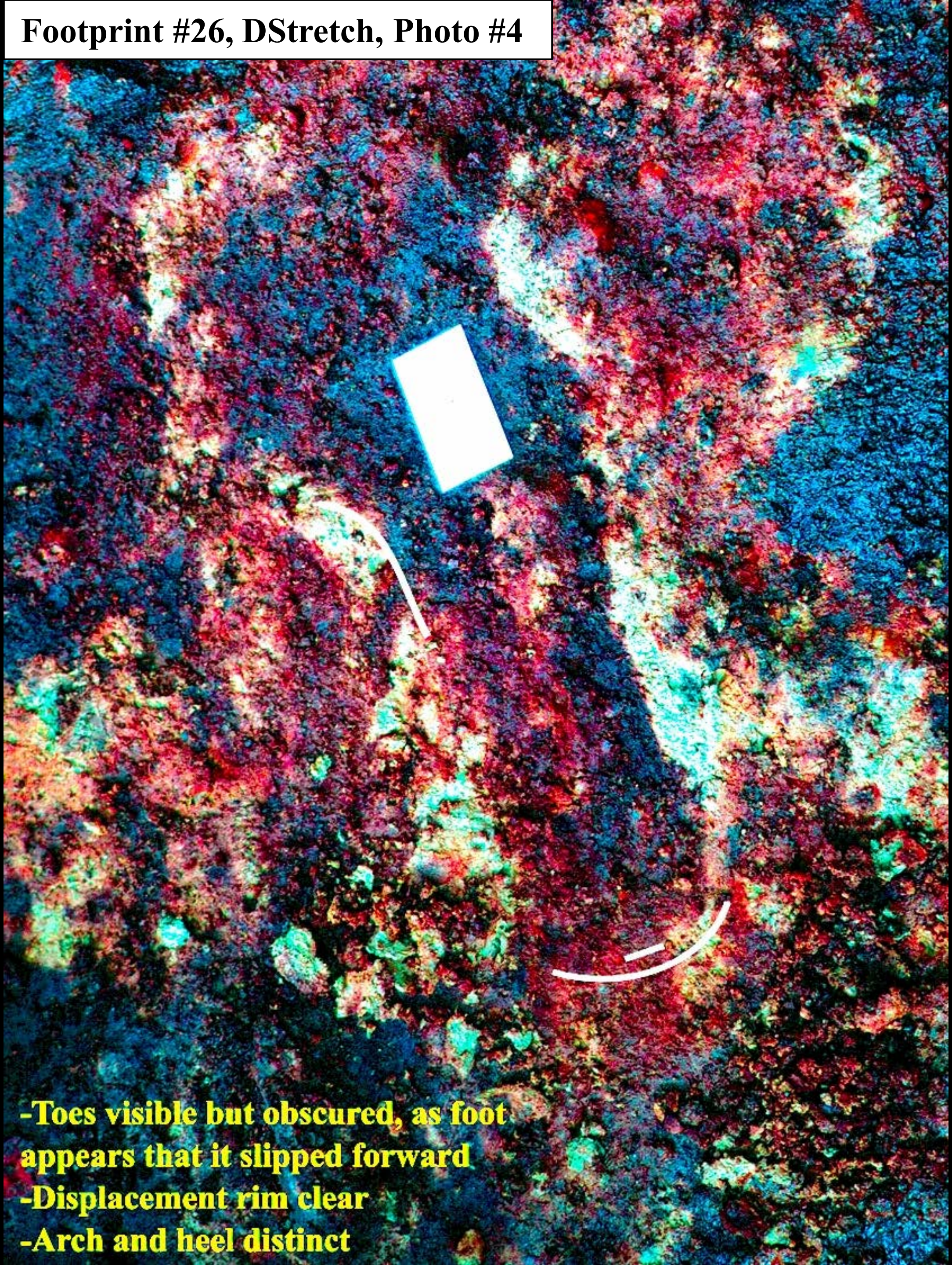
Footprint #26, DStretch, Photo #4
-Toes visible but obscured, as foot appears that it slipped forward
-Displacement rim clear
-Arch and heel distinct

Multiple Footprints 2015, Photo #1

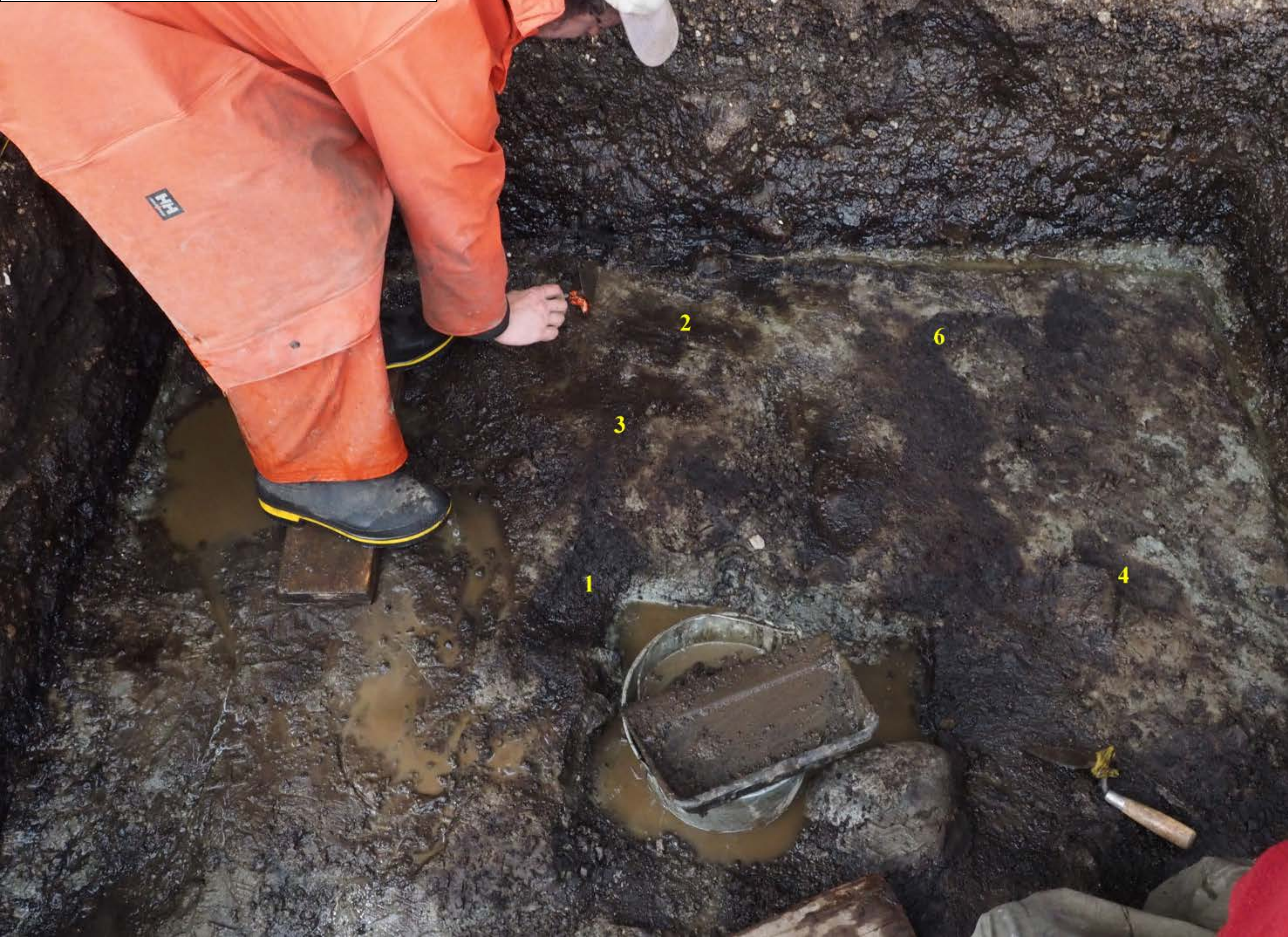

Multiple Footprints 2015, Photo #2

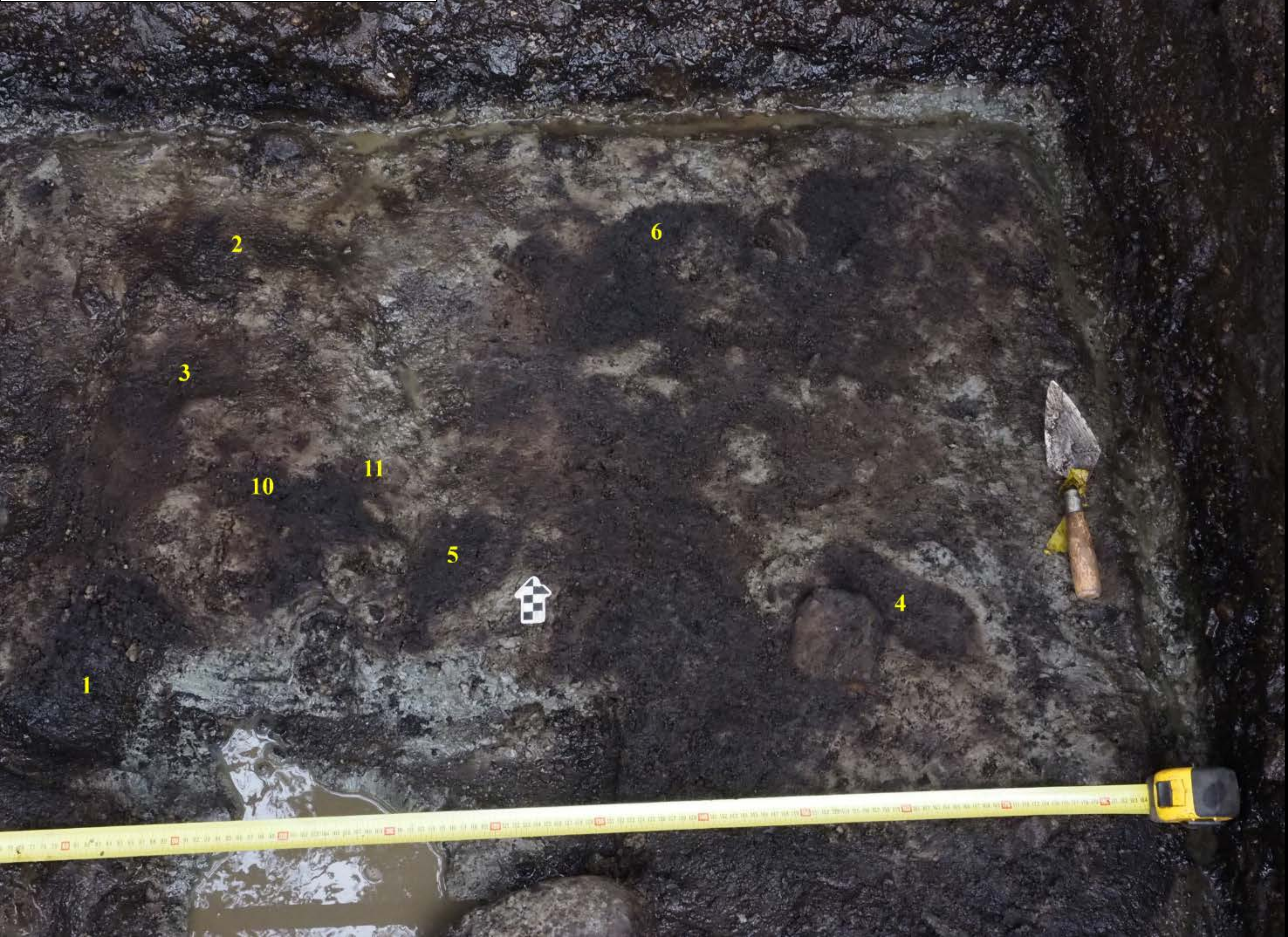

Multiple Footprints 2015, Photo #3

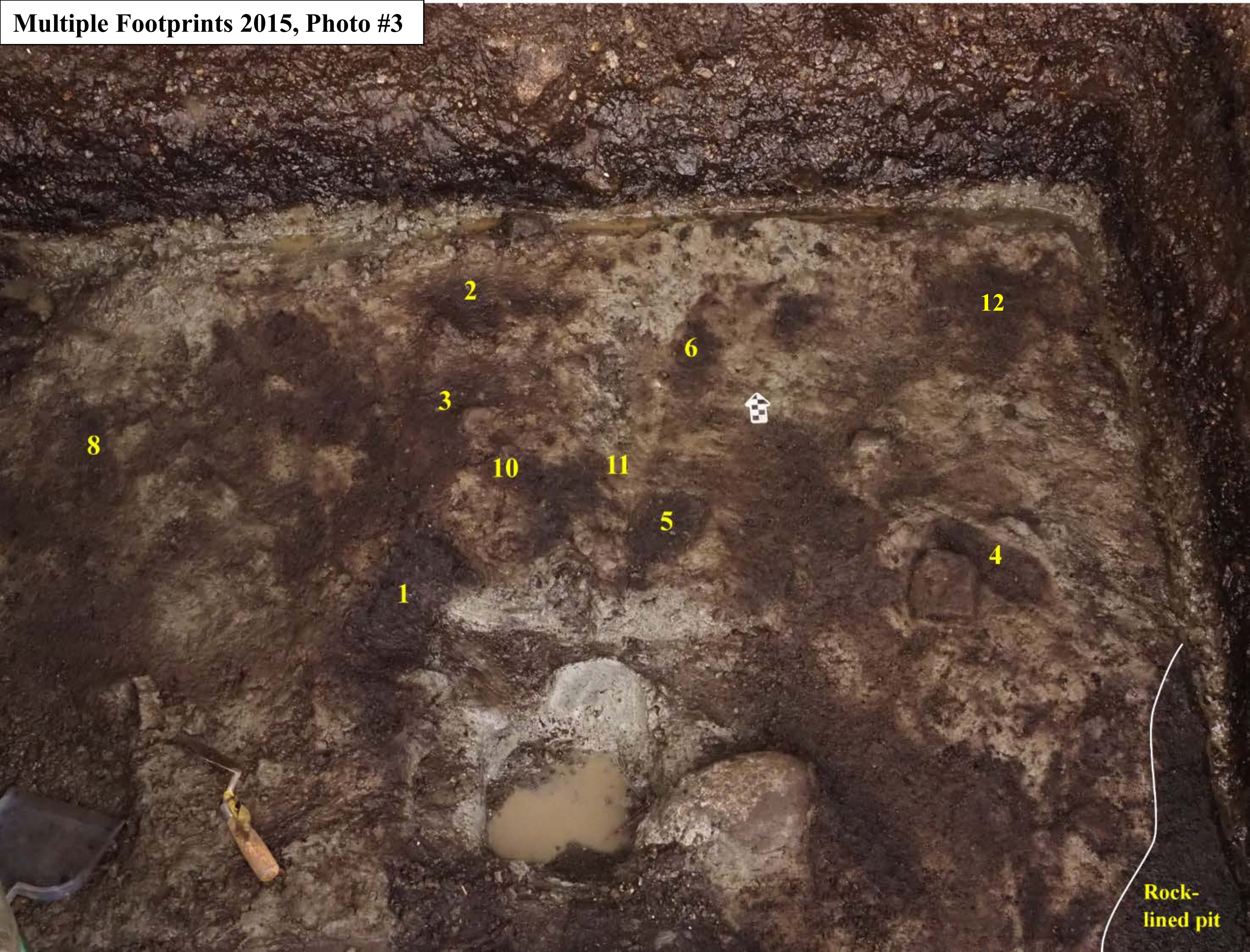

Multiple Footprints 2015, DStretch, Photo #4

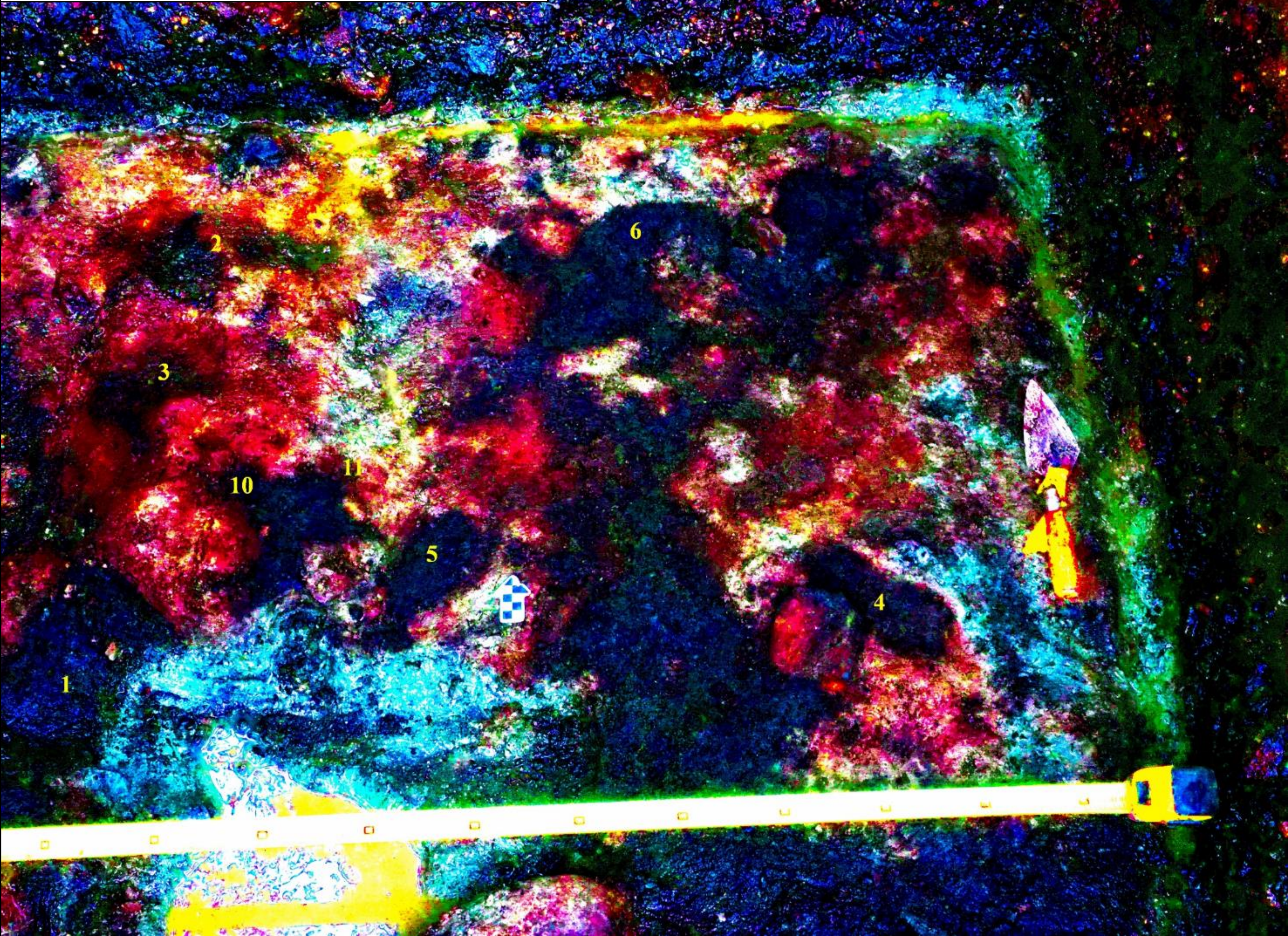

Multiple Footprints 2015, DStretch, Photo #5

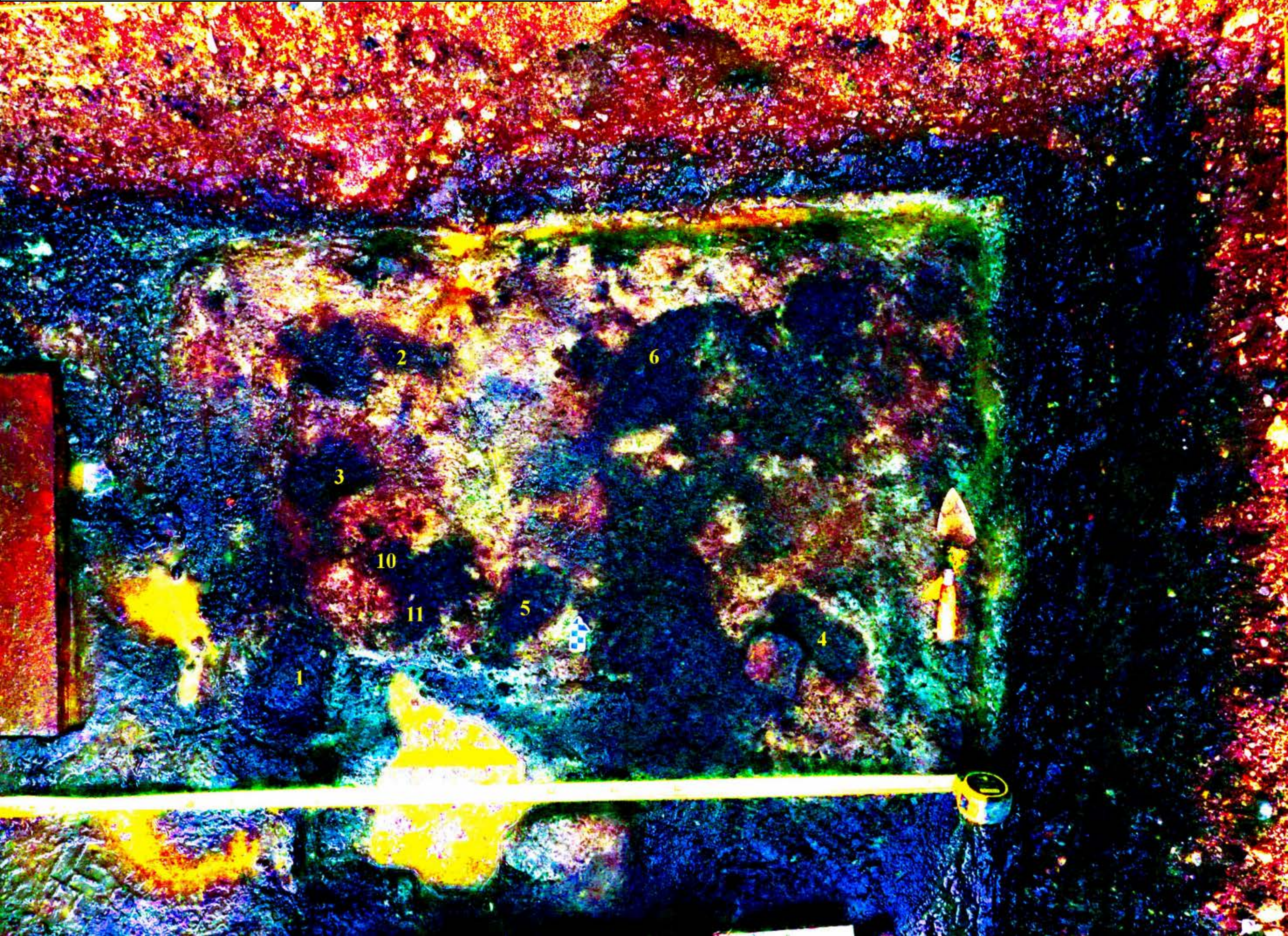

Multiple Footprints 2015, DStretch, Photo #6

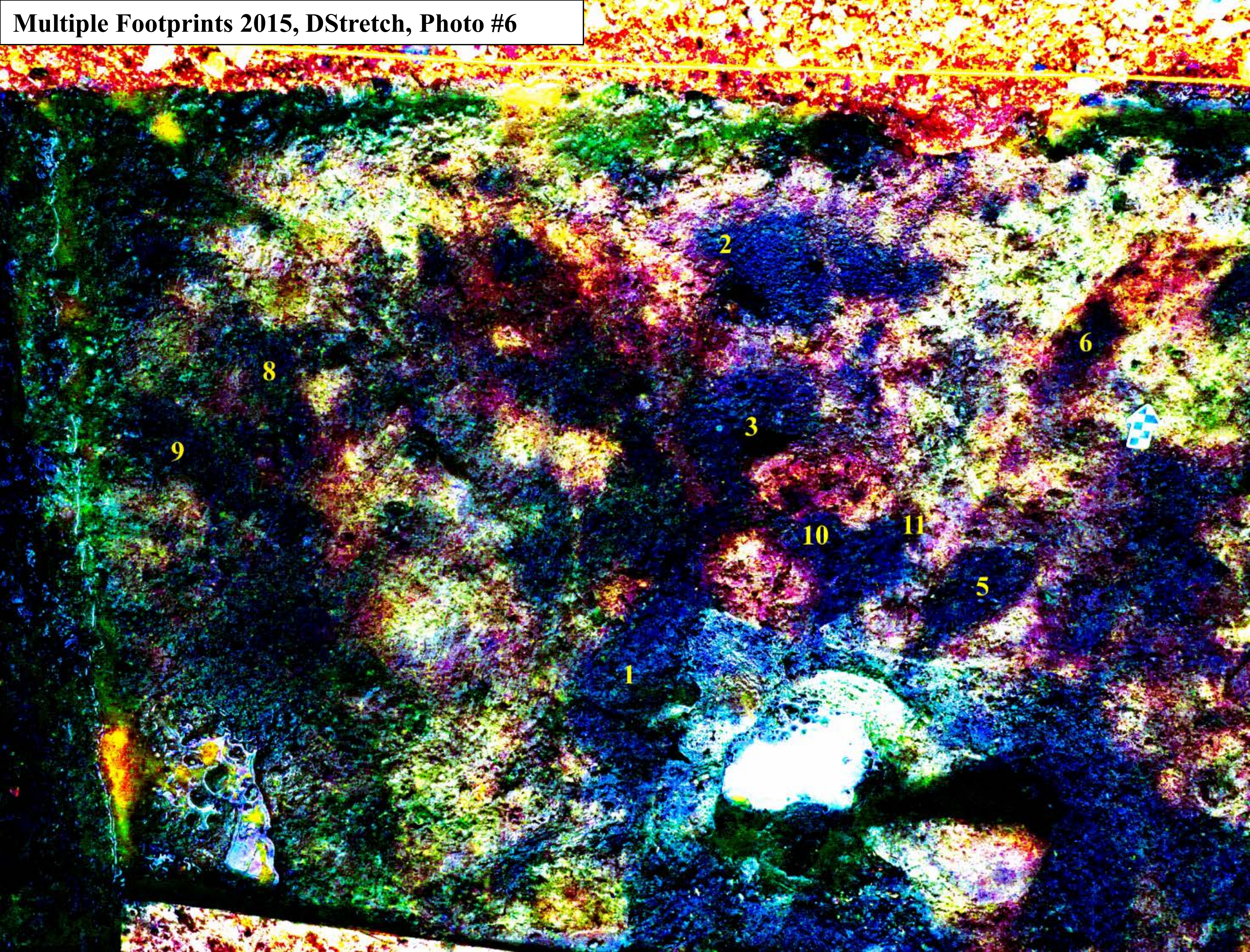

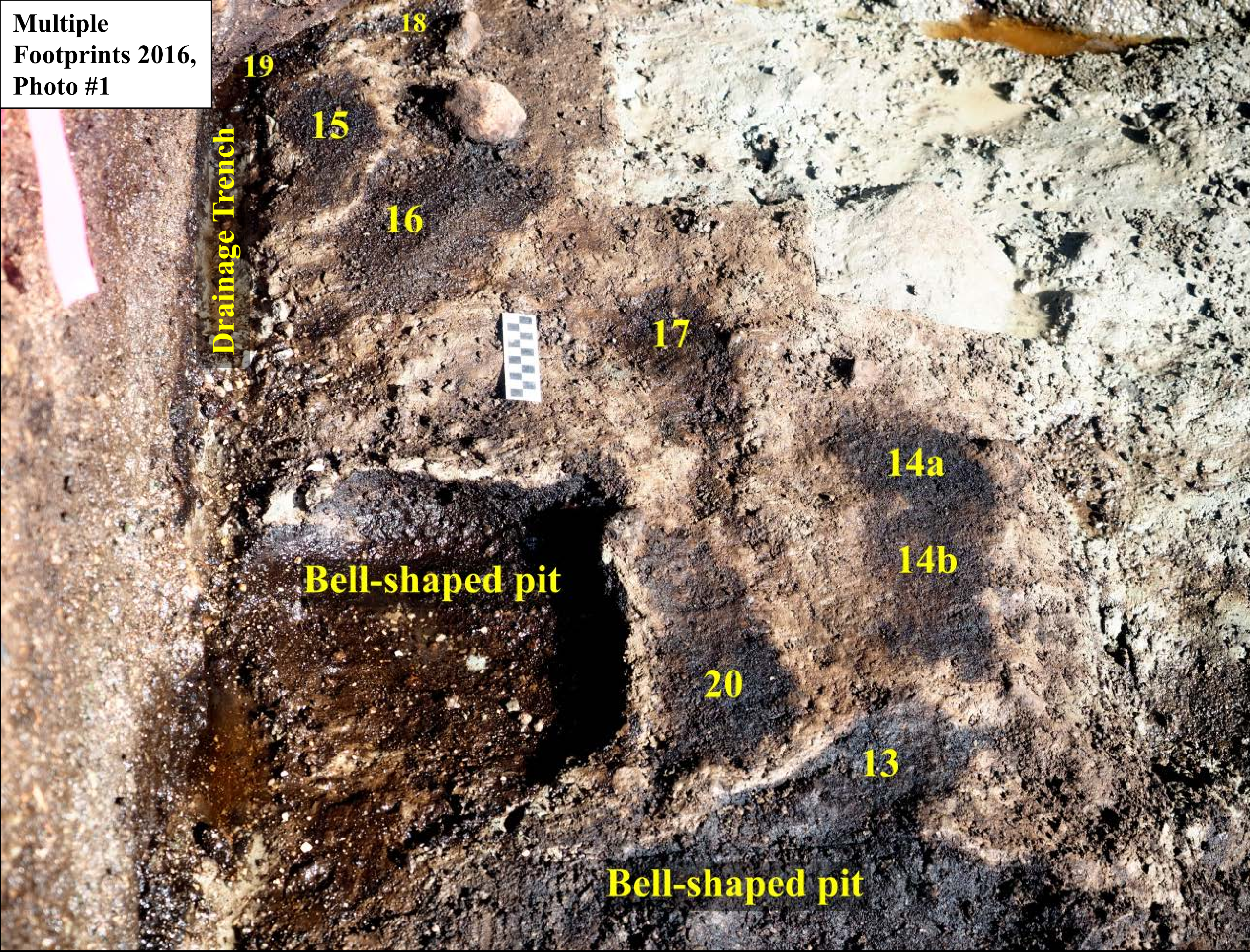
Multiple Footprints 2016, Photo #1
18
19
15
16
Drainage Trench
17
14a
14b
Bell-shaped pit
20
13
Bell-shaped pit

Multiple Footprints 2016, Photo #2

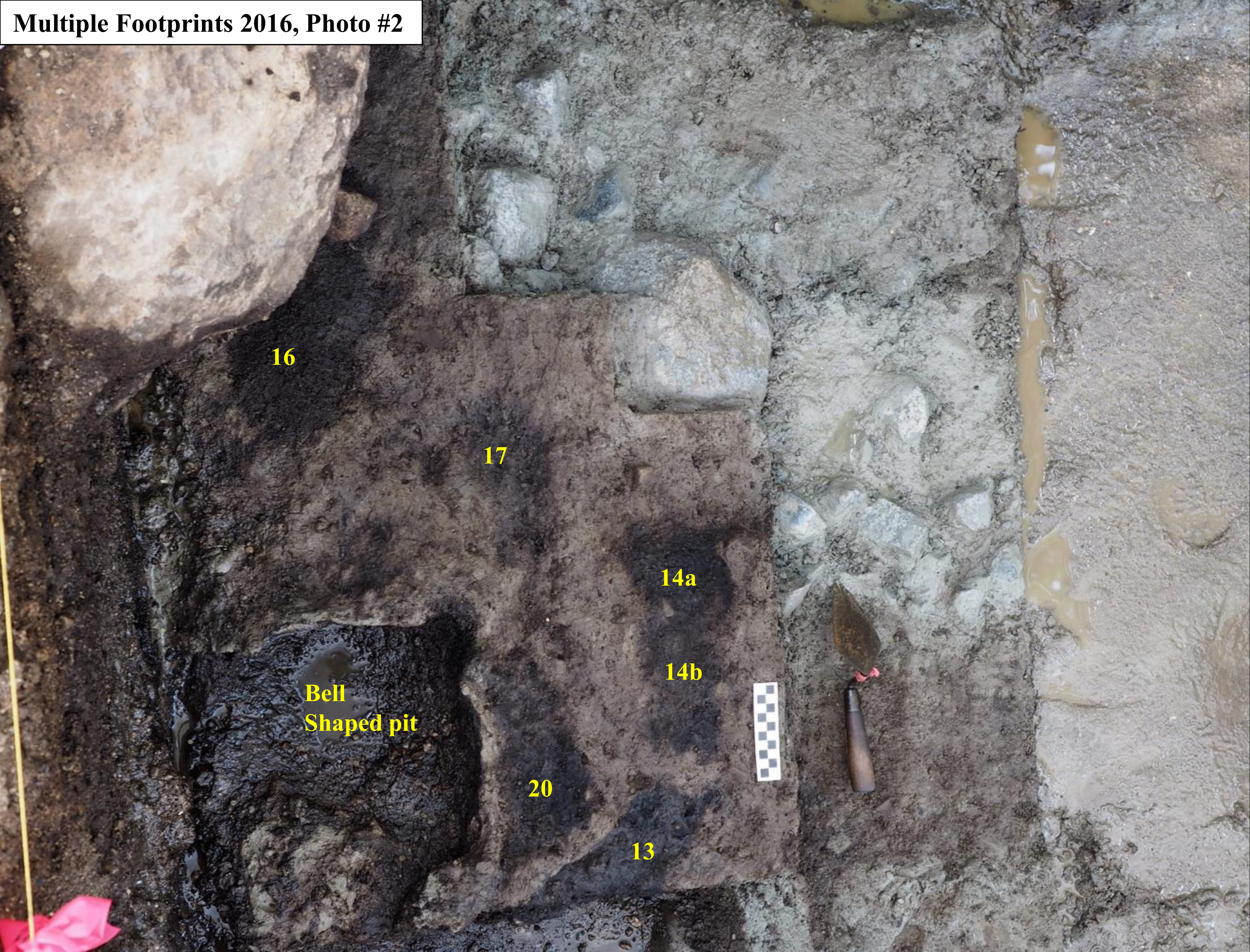

Multiple Footprints 2016, Photo #3

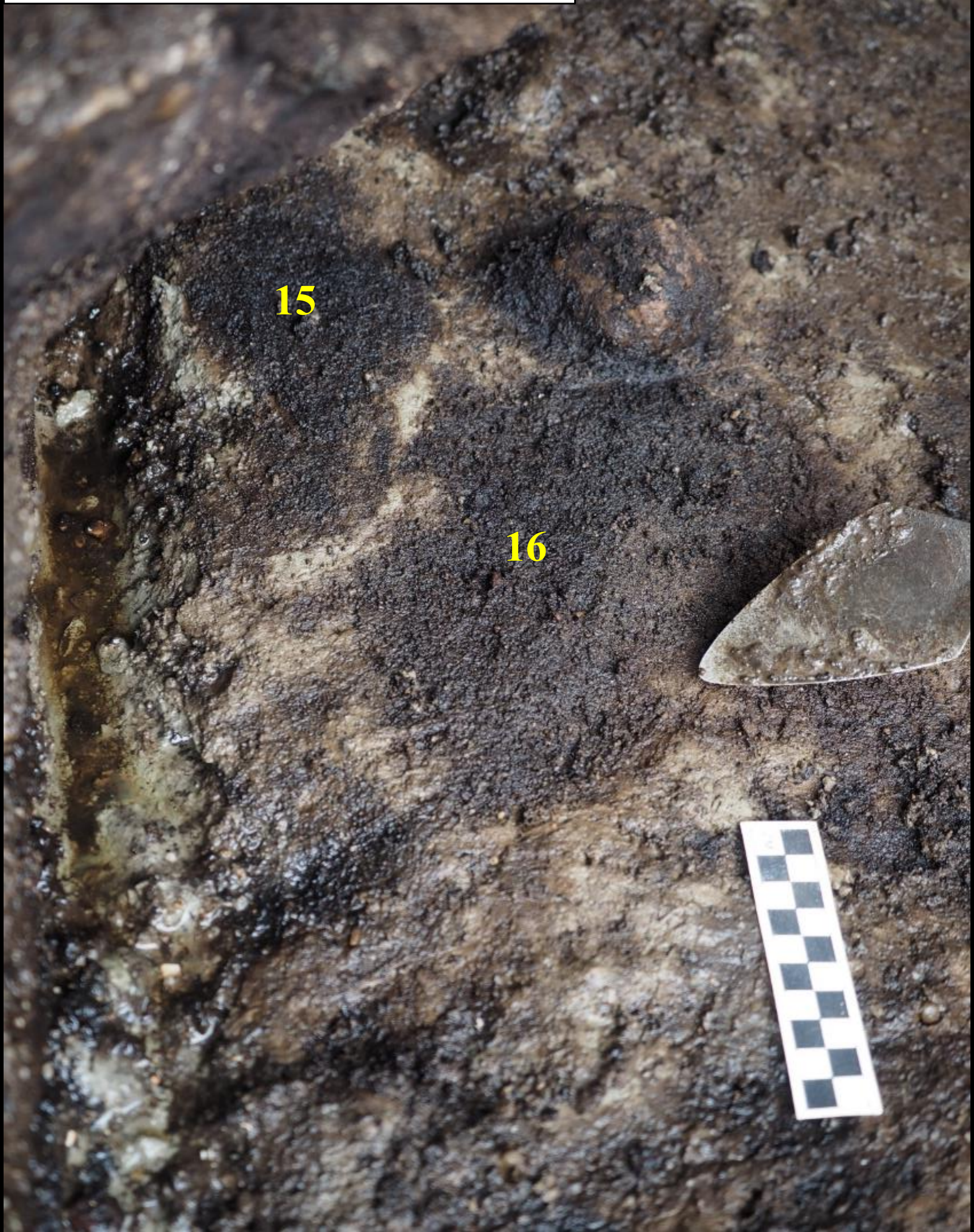

Multiple Footprints 2016, Photo #4

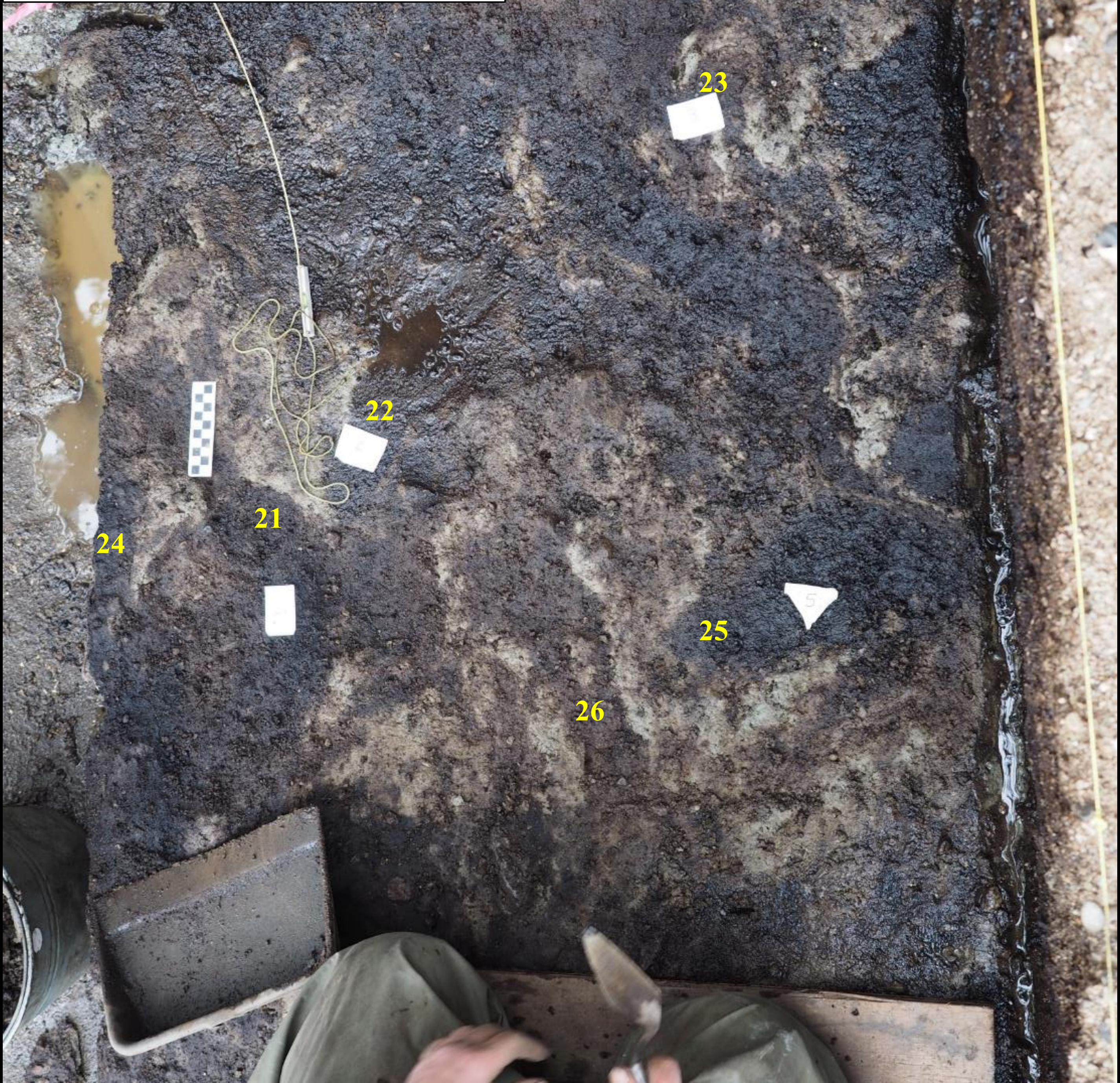

Multiple Footprints 2016, DStretch, Photo #5

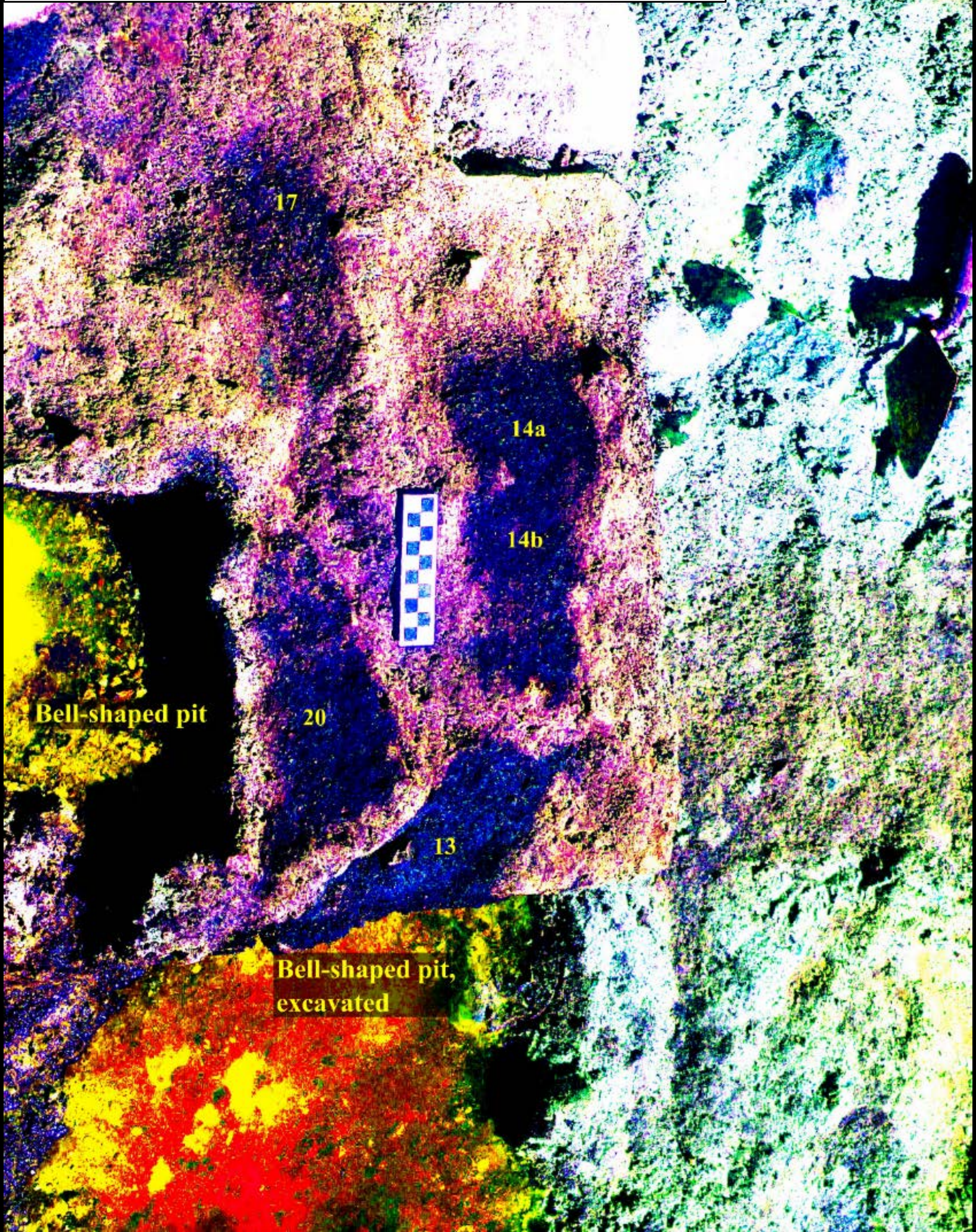

Multiple Footprints 2016, DStretch, Photo #6

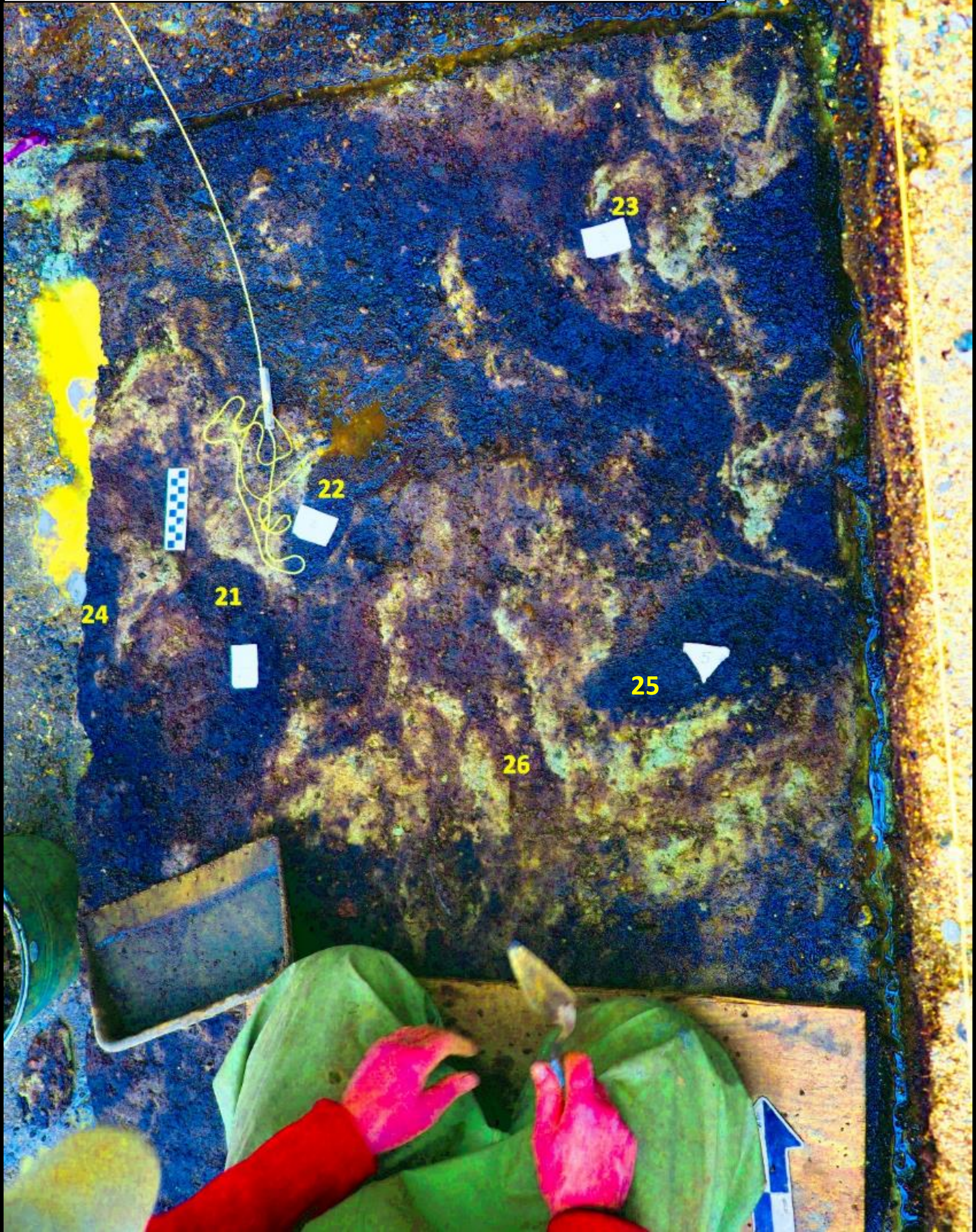

Modern Intertidal Footprint Analogues, Footprints left after one day.

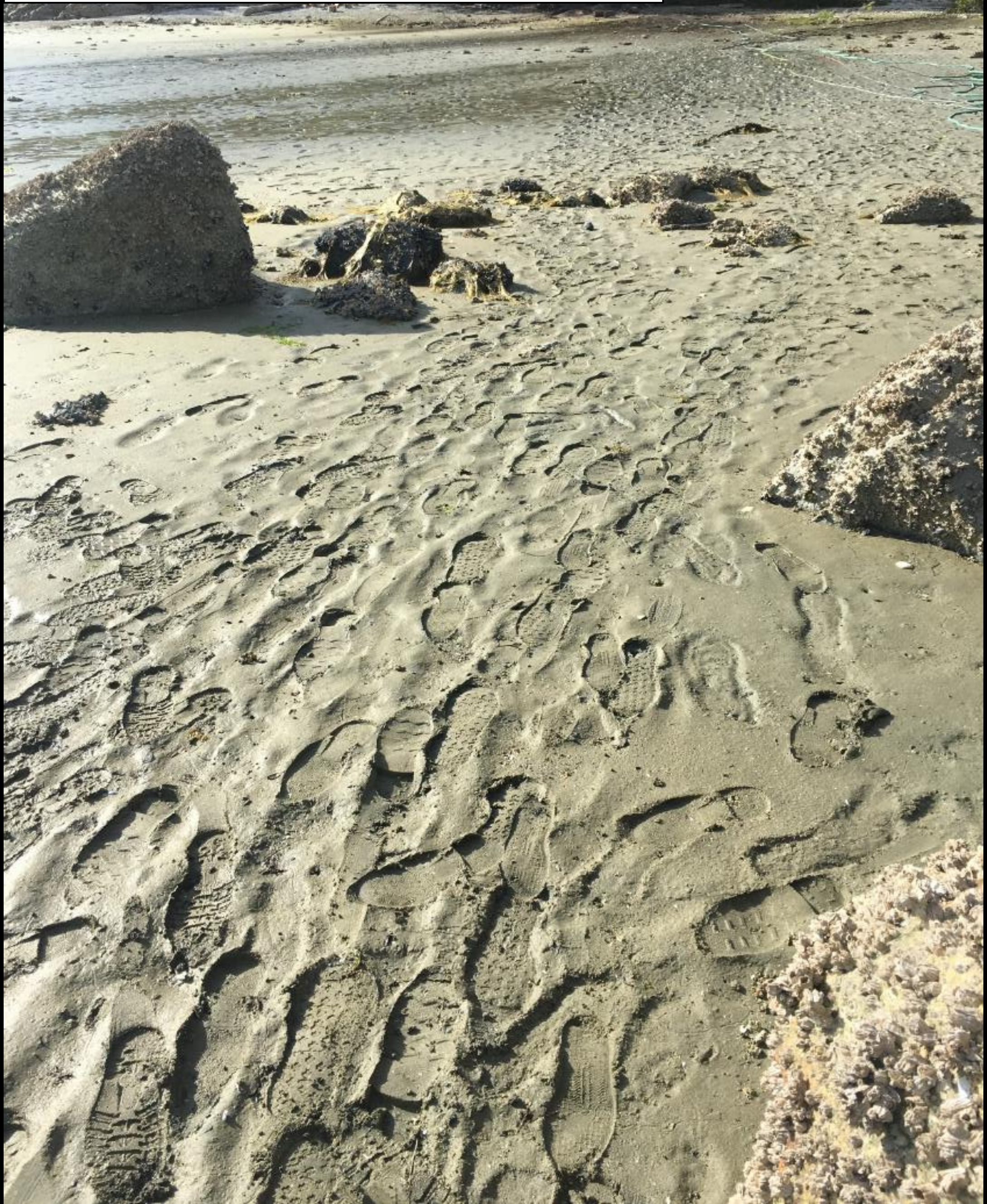

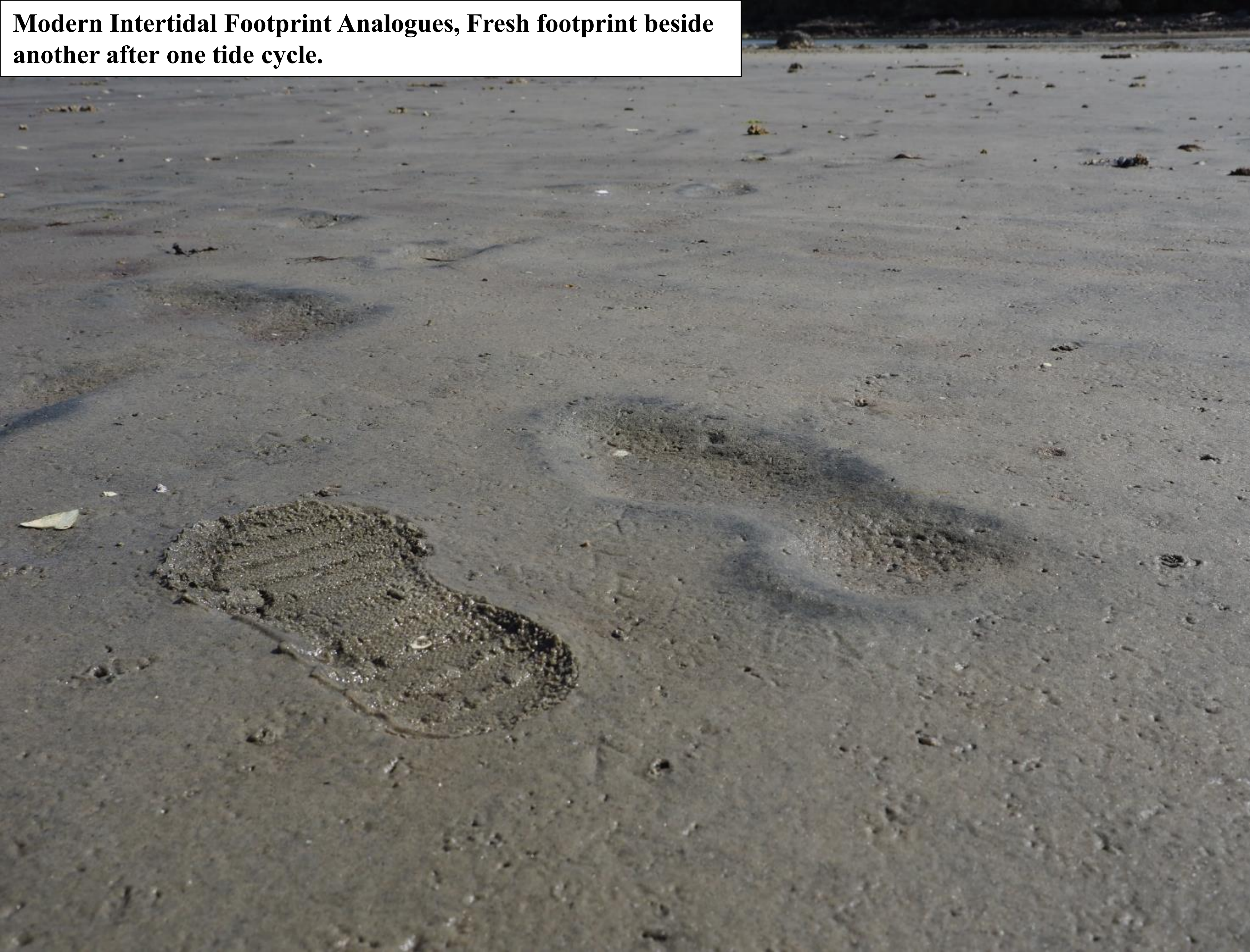

Modern Intertidal Footprint Analogues, Fresh footprint beside another after one tide cycle.

Modern Intertidal Footprint Analogues, Footprints after one tide cycle.

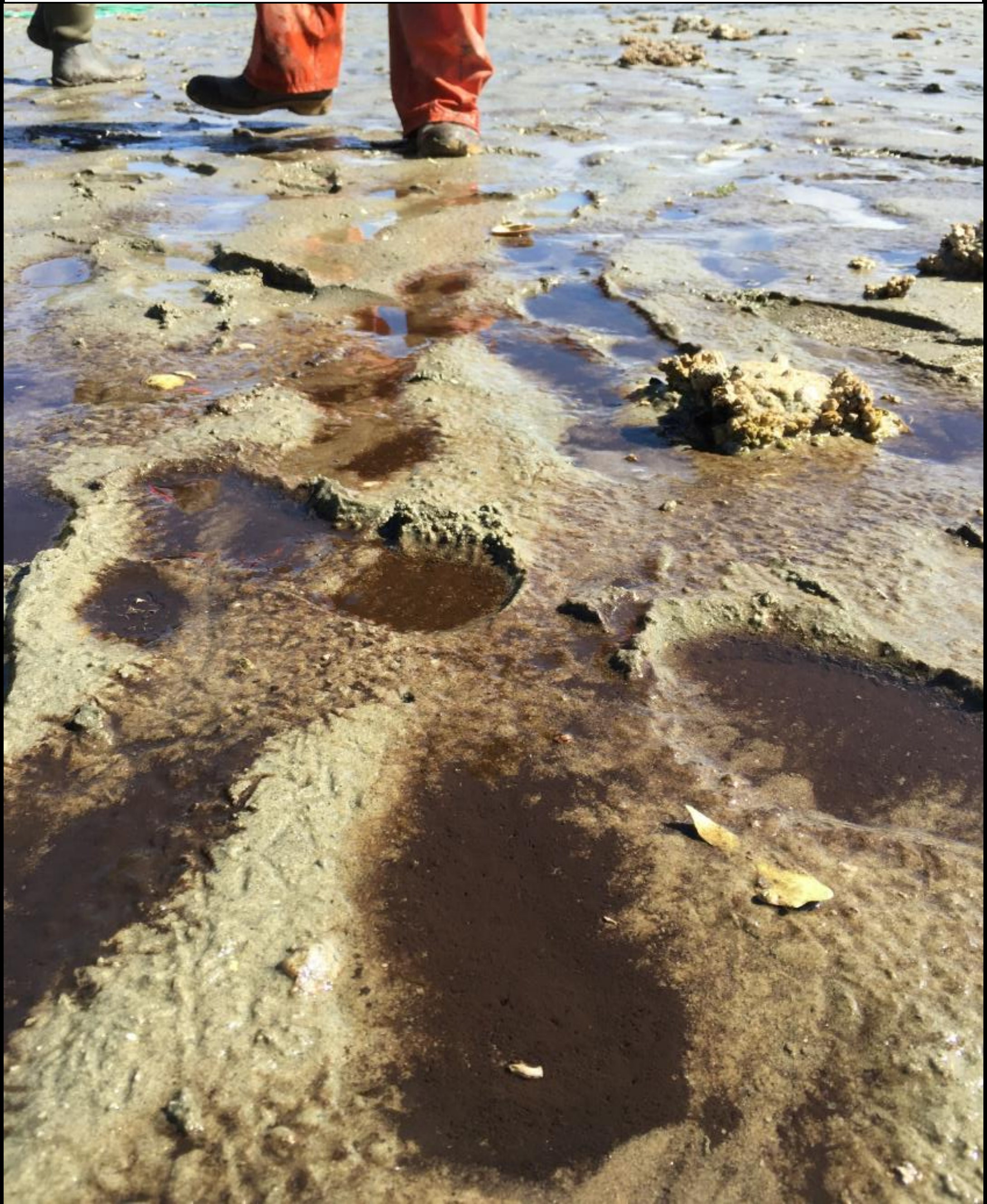

Supplement: S1 File — McLaren et al. 2018 S1 File.pdf. (PDF) [file pone.0193522.s001.pdf]
